# Synthesis of Seven- and Eight-Membered Rings by a Brønsted Acid Catalyzed Cationic Carbocyclization of Biphenyl Embedded Enynes

**DOI:** 10.1021/acs.orglett.4c00647

**Published:** 2024-04-11

**Authors:** Jaime Tostado, Ana Milián, Juan J. Vaquero, Manuel A. Fernández-Rodríguez

**Affiliations:** Universidad de Alcalá (IRYCIS). Departamento de Química Orgánica y Química Inorgánica, Instituto de Investigación Química “Andrés M. del Río” (IQAR), Autovía A-II, Km 33.1, 28805-Alcalá de Henares, Madrid, Spain

## Abstract

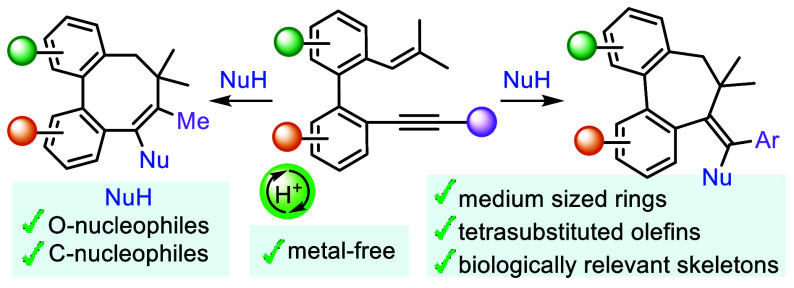

A Brønsted acid catalyzed cyclization of *o*-alkenyl-*o′*-alkynylbiaryls for the synthesis of biologically relevant dibenzo-fused medium-sized rings has been developed. The outcome of the cyclization is determined by the nature of the substituent at the alkyne, with arenes favoring seven-membered rings and alkyl substituents producing eight-membered rings. These reactions proceed via a vinyl cation, which is captured by water and, notably, by C-nucleophiles, such as electron-rich (hetero)arenes.

As carbocycles are essential motifs in bioactive compounds and materials, their synthesis from simple substrates is a central goal in chemistry. Compared to five- and six-membered rings, the construction of seven- and eight-membered rings is considerably more challenging due to unfavorable entropic and enthalpic factors.^1^ However, the prevalence of medium-sized carbocycles in bioactive molecules^2^ renders the development of new synthetic routes a key objective in organic synthesis. In particular, dibenzofused seven- and eight-membered rings are widely found in natural products and pharmaceutically relevant compounds such as allocolchicine alkaloids or dibenzocyclooctadiene lignans.^3,4^ Consequently, numerous methodologies for synthesizing these scaffolds have been devised, although their availability still remains somewhat limited.^5^

Cationic cyclization is a widely used strategy for the construction of carbo- and heterocycles from substrates bearing both electrophilic and nucleophilic moieties, via the initial activation of the electrophile, followed by intramolecular addition of the nucleophile (terminating group).^6^ This sequence generates cyclic cationic species that produce (hetero)cycles, mainly by elimination processes or nucleophilic additions. These cyclizations usually require the participation of heteroatoms to stabilize the initial cations or serve as a nucleophile. In contrast, the involvement of nonstabilized carbocations is less well-explored, and the use of carbon-centered terminating groups is primarily limited to alkenes and arenes.^7^ In this context, carbocyclization of *ortho*-alkynylbiaryls by acetylene activation, using a stoichiometric amount of a Brønsted acid, and subsequent intramolecular addition of an arene, is well established (Scheme 1a).^7−9^ Conversely, few examples of Brønsted acid catalyzed carbocyclizations of enynes in which the acetylene behaves as terminating group have been described.^10^ In these reactions, the carbocation generated by selective olefin protonation is captured by the alkyne, affording a vinyl carbocation. Both *exo* and *endo* cyclizations are possible depending on the relative stability of the carbocation, which is then typically captured by a nucleophile (Scheme 1b). Despite the significant progress achieved in these cyclizations, their applicability remains limited to the synthesis of five- and six-membered rings with no access to medium-size carbocycles. In addition, trapping of the alkenyl carbocation with external nucleophiles is restricted to halides, water, and other O-nucleophiles, and no examples employing C-nucleophiles have been reported.^11^

**Scheme 1:**
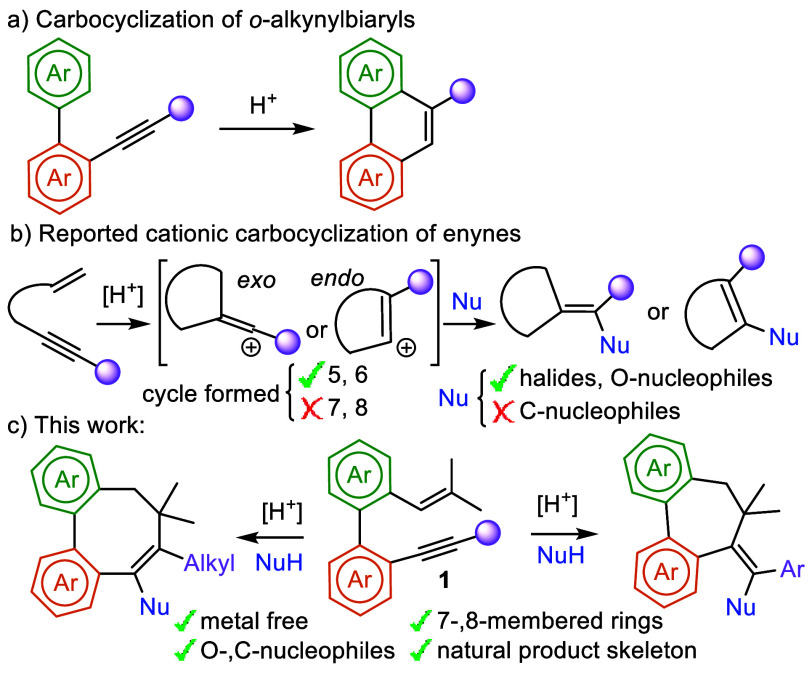
Acid-Mediated Carbocyclizations of Enynes

Herein, we present our results on the Brønsted acid catalyzed cationic carbocyclization of biphenyl embedded enynes **1**, which selectively yields dibenzofused seven- and eight-membered carbocycles. Remarkably, this new catalytic procedure enables the employment of C-nucleophiles (Scheme 1c).

As part of our ongoing research into electrophilic cycloisomerizations of *o*-alkenyl-*o′*-alkynylbiaryls **1**,^12^ we envisioned that a Brønsted acid would selectively react with these substrates via the trisubstitued olefin. The resulting carbocation would trigger a cyclization with the alkyne, thus leading to the formation of a medium-sized carbocycle, followed by the incorporation of a nucleophile. To test this hypothesis, we selected 2-(2-methylprop-1-en-1-yl)-2′-(phenylethynyl)-1,1′-biphenyl (**1a**), which comprises an aromatic-substituted acetylene, as a model. We then examined its reactivity with strong Brønsted acids in the presence of a stoichiometric amount of water as nucleophile. In the initial experiment using TfOH as catalyst (10 mol %) in 1,2-dichloroethane (DCE) at 60 °C for 12 h, we observed the selective formation in 26% yield of **2a**, which possesses an embedded seven-membered ring (Scheme 2). The structure of **2a** was determined based on NMR experiments and verified by X-ray diffraction analysis.^13^ The formation of **2a** can be explained by the proposed mechanism (Scheme 2).^10^ As anticipated, the reaction is initiated by exclusive activation of the alkene in **1a** by TfOH, thus generating the tertiary carbocation **Ia**. Subsequent intramolecular addition of the alkynyl moiety to this species would furnish cyclic vinyl carbocation **IIa** via a selective 7-*exo* carbocyclization. Finally, this intermediate would be intercepted by a water molecule, thus producing enol **IIIa**, which undergoes keto–enol tautomerization to yield ketone **2a**, along with the release of a proton for a new cycle.

**Scheme 2:**
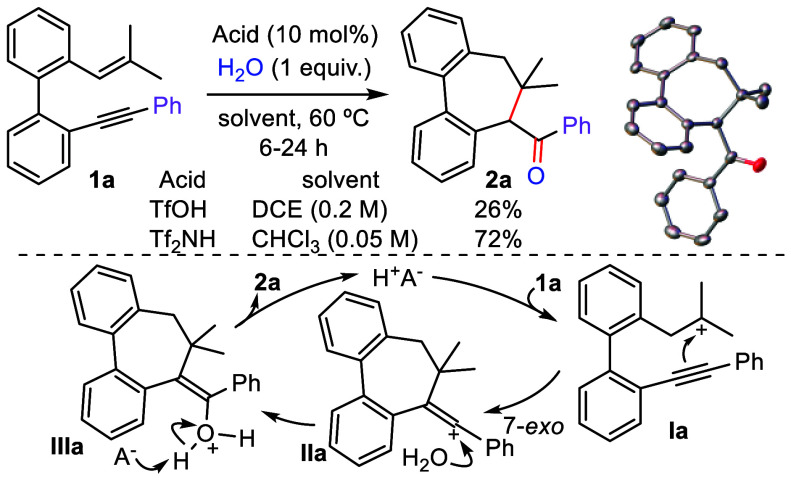
Brønsted Acid Catalyzed Cationic Carbocyclization of **1a** and Mechanistic Proposal (Thermal Ellipsoids Are Displayed at the 50% Probability Level)

In the optimization of this new reaction,^14^ we found that Tf_2_NH gave an improved 44% yield, while weaker acids did not promote any reaction. The solvent has a marked influence, and only experiments in 1,4-dioxane or chloroform gave **2a** in similar yields, with the latter being the optimal. Additionally, increasing the amount of water or varying the temperature did not have a significant impact on the cyclization. However, reducing the reaction concentration to 0.05 M afforded the tricyclic adduct **2a** in a remarkable 72% yield in less than 6 h (Scheme 2).^14^ Considering that these cyclizations selectively proceed via the more stable alkenyl carbocation intermediate,^15^ we envisaged that a suitable substituted enyne could change the reaction outcome. Thus, we proposed that an alkyl group would be unable to stabilize a carbocation at its adjacent carbon (**IIb-exo**), thereby inducing an alternative 8-*endo* cyclization that would produce a more stable **IIb-***endo* species (Scheme 3). Subsequent reaction with water would render compound **3** with an eight-membered ring. Therefore, enyne **1b**, which bears a methyl group on the triple bond, was prepared and subjected to the optimized conditions determined for **1a**. A complete inversion in the regioselectivity occurred, leading to the exclusive formation of cyclooctadienone **3b** in 64% yield. Given the relevance of selectively forming **3b** from enyne **1b**, we further optimized the reaction conditions by varying the acid, solvent, and temperature.^14^ TfOH was found to be the most efficient catalyst, providing a 95% yield of **3b**, and changing the solvent to DCE afforded **3b** quantitatively. Moreover, extending the reaction time to 48 h allowed the carbocyclization to occur in high yields either at room temperature or with sustainable solvents like EtOAc and dimethyl carbonate (DMC).

**Scheme 3:**
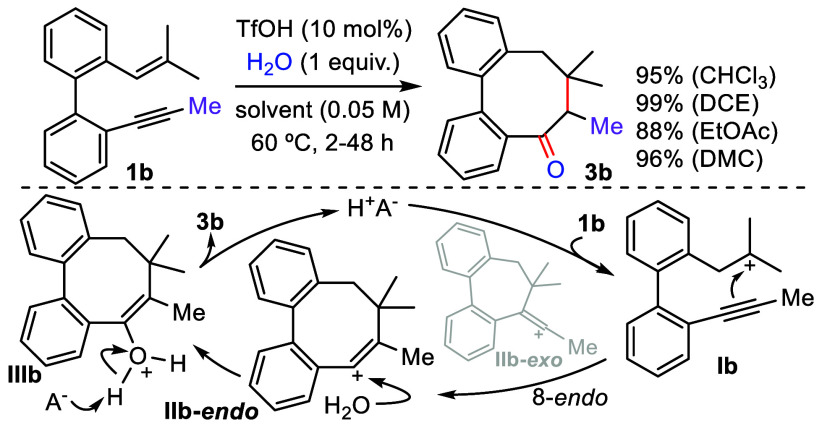
Cationic Carbocyclization of **1b**

After establishing the appropriate conditions for the intended selective synthesis of dibenzocycloheptadiene **2a** from enyne **1a**, we proceeded to explore the scope of this catalytic transformation (Scheme 4). First, a series of *o*-alkenyl-*o′*-alkynylbiaryls **1** were reacted in the presence of water (1.1 equiv) as an external nucleophile. As depicted in Scheme 4, the methodology developed is compatible with *p-* and *o-*electron-withdrawing-substituted arenes at the alkyne, leading to the corresponding dibenzocycloheptadienones **2c**–**e** in good to excellent yields. Substrates with electron-donating arenes and naphthyl groups at the same position also undergo carbocyclization, affording compounds **2f**,**h**,**i** in slightly lower yields. Only the reaction of **1g**, which bears a *p-*tolyl group, produced undesired phenanthrenes via the alternative alkyne activation, thus resulting in a significant decrease in the yield of the desired dibenzocycloheptadiene **2g**. Moreover, the biphenyl core of enynes **1** could be decorated with electron-donating and/or -withdrawing groups, providing ketones **2j**–**o** in good yields.^16^ To broaden the utility of the methodology, we investigated the possibility of trapping carbocation **IIa** (Scheme 2) with C-nucleophiles.^17^ For this purpose, reaction of **1a** with 1,2-dimethoxybenzene (1.5 equiv) was conducted under the optimal conditions determined for generating ketone **2a**. As a result, a new dibenzocycloheptadiene **4aa**, which incorporates dimethoxybenzene in its structure, was selectively obtained in 82% yield. Remarkably, this reaction represents the first reported example of the introduction of an external carbon-nucleophile in a Brønsted acid catalyzed enyne cationic carbocyclization. Furthermore, the formation of **4aa** entails the regio- and stereoselective creation of two new C–C bonds and a seven-membered ring. A brief optimization process was performed, finding that using triflimide (10 mol %), 1,2-dimethoxybenzene (1.1 equiv) in DCE (0.1 M) at 60 °C for 30 min afforded **4aa** in 91% yield (Scheme 4).^14^ Interestingly, this reaction could be conducted on a 1 mmol scale, with no significant impact on either the yield or the reaction time or at room temperature (79% yield). The scope of this novel transformation involving an external carbon-nucleophile was then examined. Reaction of selected substrates **1** bearing a *p-*chlorophenyl group at the alkyne, or electron-withdrawing or -donating substituents at the biphenyl core, selectively and efficiently yielded functionalized dibenzocycloheptadienes **4ca**, **4ja**, **4ka**, **4ma**, and **4na**. Next, we focused our attention on the carbon-nucleophilic counterpart, and the reaction of various electron-rich (hetero)aromatic compounds with substrate **1a** was analyzed. We found that bulky 1,3-dimethoxybenzene and 1,3,5-trimethoxybenzene are useful nucleophiles to build products **4ab** and **4ac** in moderate yields. As expected, substrate **1a** underwent a nonregioselective reaction with 1-methoxynaphthalene to furnish a mixture of compounds **4ad** and **4ad′** in a combined 88% yield. Despite their relatively lower nucleophilicity, anisole and mesitylene proved to be effective reagents for this cyclization process, giving rise to successful generation of the corresponding tricyclic products **4ae** and **4af**.^18^ Furthermore, the addition of π-excedent heteroarenes, like 2-methylthiophene and *N*-acetylated indoles, to enyne **1a** led to formation of 2-thiophenyl and 3-indolyl substituted dibenzocycloheptadienes **4ag**–**i** in good yields. Notably, cycloadducts **4** contain a triaryl-substituted alkene motif, the generation of which is of significant interest due to the complexity associated with their synthesis^19^ and their occurrence in natural products and drugs.^20^ The structural assignment for dibenzocycloheptadienes **4**, including the *Z* configuration of this newly formed all-carbon substituted double bond,^21^ was unambiguously confirmed by X-ray diffraction analysis of **4ca** and **4ae**.^13^

**Scheme 4:**
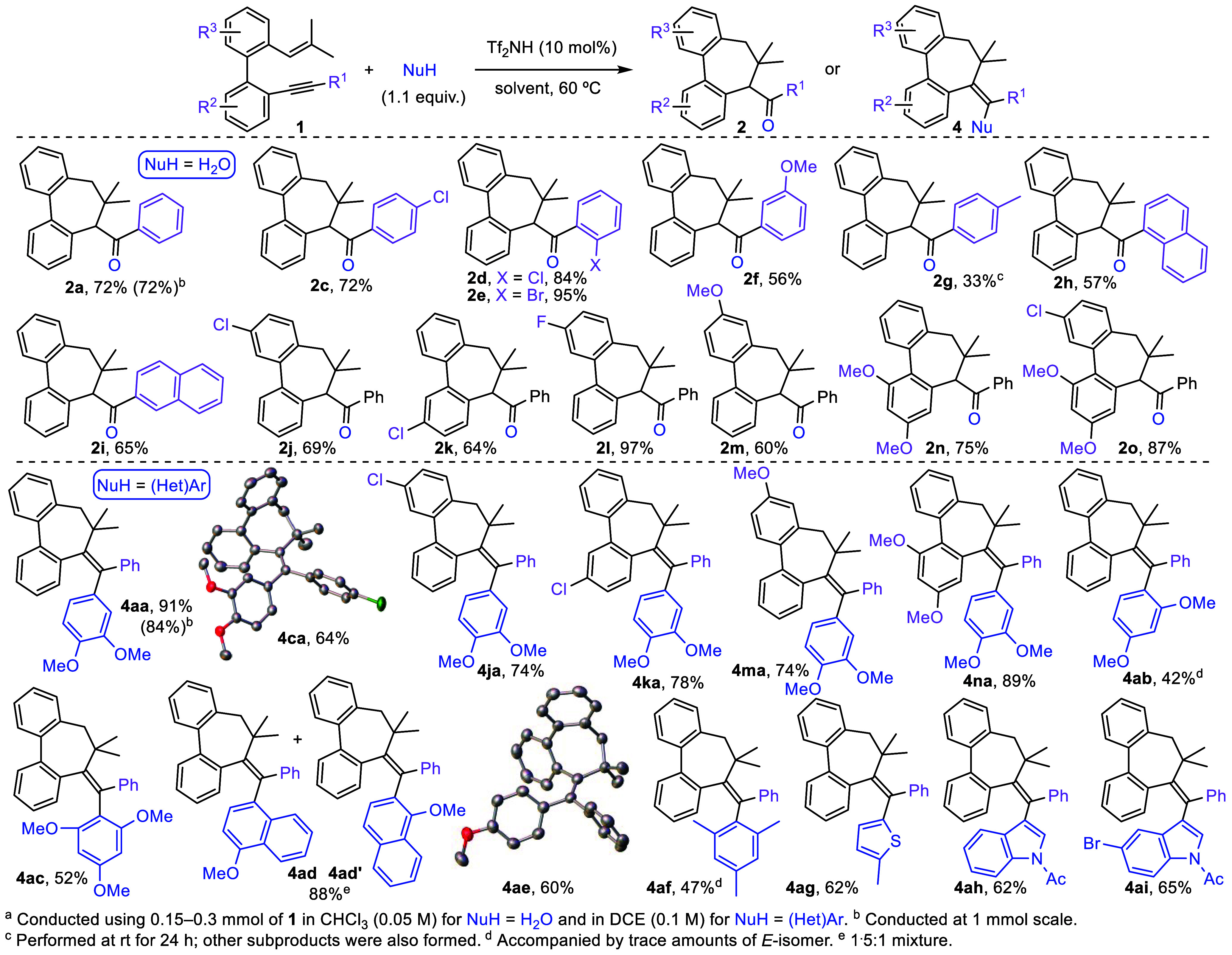
Synthesis of Dibenzocycloheptadienes **2** and **4**^*a*^ (Thermal Ellipsoids Are Displayed at the 50% Probability Level)

After assessing the applicability of the established method for synthesizing dibenzocycloheptadienes **2** and **4**, our next objective was to investigate the Brønsted acid catalyzed nucleophilic cyclization of alkyl-substituted enynes **1** for the synthesis of dibenzocyclooctatrienes **3**. As a proof of concept, reactions of selected enynes **1** with chlorine, methyl and/or methoxy groups at the biphenyl core were carried out under the optimized conditions, giving rise to ketones **3p**–**t** with total selectivity and high yields, with the exception of polysubstituted adduct **3t** that was obtained with a moderate yield (Scheme 5). The identity of biphenyl-embedded cyclooctanone was confirmed by X-ray single-crystal diffraction analysis of **3q**.^13^ We also examined the introduction of 1,2-dimethoxybenzene as a nucleophile. This reaction, which was conducted under slightly modified conditions, proceeded rapidly, and selectively delivered dibenzocyclooctatriene **6ba** in 58% yield.

**Scheme 5:**
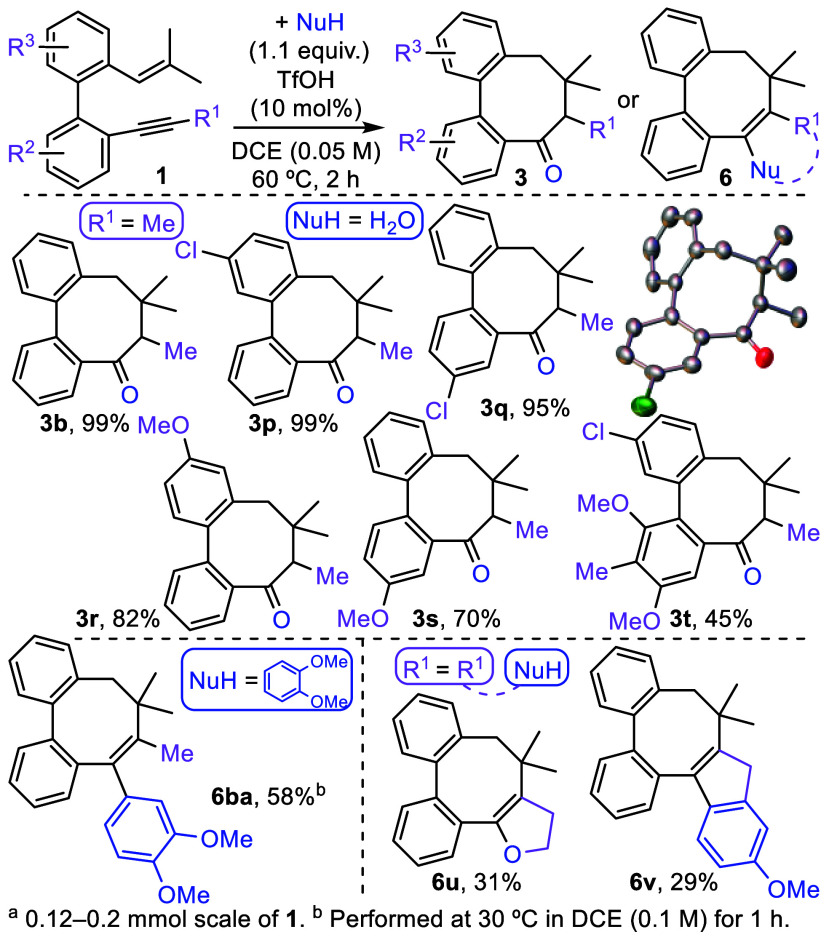
Synthesis of Dibenzocyclooctadienones **3** and **6** (Thermal Ellipsoids Are Displayed at the 50% Probability Level)

To prove the feasibility of an intramolecular version of the process, we prepared enynes **1u**–**v**. These substrates contain a hydroxyl or a *p*-methoxyphenyl group appropriately linked to the alkyne moiety to serve as an internal nucleophile to trap intermediate **IIb-endo** during the cyclization. The reactions thereof, under the optimized conditions for the synthesis of ketones **3**, allowed the selective production of tetra- and pentacyclic compounds **6u**–**v** in moderate yields (Scheme 5).

In conclusion, we have developed a novel Brønsted acid catalyzed cationic cyclization of biphenyl-embedded 1,7-enynes, in the presence of suitable O- and C-nucleophiles, to selectively produce dibenzofused seven- and eight-membered carbocycles. The use of water as a typical external nucleophile in Brønsted acid catalyzed reactions with enynes leads to the formation of tricyclic ketones. More remarkably, the employment of C-nucleophiles, such as electron-rich (hetero)arenes, which gives rise to the corresponding biaryl-embedded medium-sized all-carbon rings, has been reported for the first time in this type of metal-free transformations. Furthermore, the size of the central ring within the tricyclic system is controlled by stabilization of the vinyl cation generated upon addition of the alkyne to the carbocation species initially formed via selective alkene activation. Thus, enynes bearing arenes at the alkyne yield seven-membered rings, whereas substrates having alkyl groups at the same position afford eight-membered rings. The methodology developed herein has demonstrated its generality and compatibility with various functional groups, delivering over 30 functionalized polycycles in good yields. Notably, the carbocyclic scaffolds synthesized are present in bioactive compounds, including allocolchicine alkaloids and dibenzocyclooctadiene lignans.

## Data Availability

The data underlying this study are available in the published article and its Supporting Information.

## Supporting Information Available

Experimental details, NMR spectra for all new compounds and X-ray crystallographic data for **2a**, **3q**, **4ae**, and **4ca** (PDF)
