# Supplementary material for: Synthesis of Seven- and Eight-Membered Rings by a Brønsted Acid Catalyzed Cationic Carbocyclization of Biphenyl Embedded Enynes
Source: Org Lett. 2024 Apr 11;26(16):3343–8. doi: 10.1021/acs.orglett.4c00647 (PMC11059095; doi:10.1021/acs.orglett.4c00647)
Supplement: Supplementary file 1 — ol4c00647_si_001.pdf [file ol4c00647_si_001.pdf]

Supporting Information

Synthesis of Seven- and Eight-Membered Rings by a Brønsted Acid-Catalyzed Cationic Carbocyclization of Biphenyl Embedded Enynes

Jaime Tostado, Ana Milián, Juan J. Vaquero and Manuel A. Fernández-Rodríguez*

Universidad de Alcalá (IRYCIS). Departamento de Química Orgánica y Química Inorgánica, Instituto de Investigación Química "Andrés M. del Río" (IQAR). Campus Científico-Tecnológico, Facultad de Farmacia. Autovía A-II, Km 33.1, 28805-Alcalá de Henares, Madrid, Spain.

Table of Contents

General Experimental Details ---------- S2

Experimental Procedures and Data ---------- S3

Table S1. Optimization of the cyclization of **1a** with water as nucleophile ---------- S3

Table S2. Optimization of the cyclization of **1b** with water as nucleophile ---------- S3

Reaction of **1g** with water as nucleophile ---------- S4

Table S3. Optimization of the cyclization of **1a** with 1,2-dimethoxybenzene as nucleophile - S4

Table S4. Optimization of the cyclization of **1b** with 1,2-dimethoxybenzene as nucleophile - S4

General procedures for the synthesis of 2-alkenyl-2'-alkynyl-1,1'-biphenyls **1** ---------- S5

Synthesis of 2-(4-hydroxybut-1-yn-1-yl)-2'-(2-methylprop-1-en-1-yl)-1,1'-biphenyl (**1u**) ------ S6

Synthesis of 2-(3-(3-methoxyphenyl)prop-1-yn-1-yl)-2'-(2-methylprop-1-en-1-yl)-1,1'-biphenyl (**1v**) ---------- S7

Characterization data of unknown biphenyl embedded trienynes **1** and their precursors ---- S7

General procedure for the synthesis of dibenzocycloheptadienes **2** and **4** ---------- S14

Characterization data of dibenzocycloheptadienes **2** and **4** ---------- S14

General procedure for the synthesis of dibenzocyclooctadienones **3** ---------- S23

Characterization data of dibenzocyclooctadienones **3** ---------- S23

Synthesis of 8-(3,4-dimethoxyphenyl)-6,6,7-trimethyl-5,6-dihydrodibenzo[*a*,*c*]cyclooctatriene (**6ba**) ---------- S24

Synthesis of 8,8-dimethyl-6,7,8,9-tetrahydrodibenzo[5,6:7,8]cycloocta[1,2-b]furan (**6u**) --- S25

Synthesis of 13-methoxy-10,10-dimethyl-10,11-dihydro-9*H*-dibenzo[5,6:7,8]cycloocta[1,2-a]indene (**6v**) ---------- S25

Copies of ^{1}H and ^{13}C spectra for novel compounds and selected gCOSY, TOCSY, NOESY, gHSQC and gHMBC spectra ---------- S26

Crystallographic data for **2a**, **3q**, **4ae** and **4ca** ---------- S178

General Experimental Details

All reactions involving air sensitive compounds were carried out under inert atmosphere (Ar). Temperatures are reported as oil-bath temperatures. Dry solvents, where necessary, were dried by a MBRAUN MB-SPS-800 apparatus. Starting materials sourced from commercial suppliers were used as received unless otherwise stated. Substrates **1a-d,g,i-k,m-o,w-x** and their non-commercial precursors were previously described and were prepared as reported.[1] 1-acetylindole and 1-acetyl-5-bromoindole were prepared according to these previous literature procedures.[2] Reactions were monitored using analytical TLC plates (Merck; silica gel 60 F254, 0.25 mm), and compounds were visualized with UV radiation. Silica gel grade 60 (70-230 mesh, Merck) was used for column chromatography. All melting points were determined in open capillary tubes on a Stuart Scientific SMP3 melting point apparatus (uncorrected). ^{1}H and ^{13}C NMR spectra were recorded on either Varian Mercury VX-300, Varian Unity 300, Bruker Avance Neo 400 or Varian Unity 500 MHz spectrometer at room temperature. Chemical shifts are given in ppm (δ) downfield from tetramethylsilane, with calibration on the residual protio-solvent used (δ_H = 7.26 ppm and δ_C = 77.2 ppm for $CDCl_3$). Coupling constants (*J*) are in Hertz (Hz) and signals are described as follows: AB system; s, singlet; d, doublet; t, triplet; q, quadruplet; bs, broad singlet; dd, double doublet; dt, doublet of triplets; ddd, double doublet of doublets; td, triplet of doublets and m, multiplet. Structural assignments were made with additional information from gCOSY, gHSQC, and gHMBC experiments. High-resolution analyses (HRMS) were performed on an Agilent 6210 time of-flight LC/MS.

[1] a) Milián, A.; Fernández-Rodríguez, M. A.; Merino, E.; Vaquero, J. J.; García-García, P. *Angew. Chem. Int. Ed.* **2022**, *61*, e202205651. b) Milian, A.; García-García, P.; Pérez-Redondo, A.; Sanz, R., Vaquero, J.; Fernández-Rodríguez, M. A. *Org. Lett.* **2020**, *22*, 8464−8469.

[2] a) Zysman-Colman, E.; Arias, K.; Siegel, J. S. *Can. J. Chem.* **2009**, *87*, 440–447. b) Chen, X.; Zhou, X. *Synthesis.* **2019**, *51*, 516-521.

Experimental Procedures and Data

Table S1. Optimization of the cyclization of 1a with water as nucleophile:

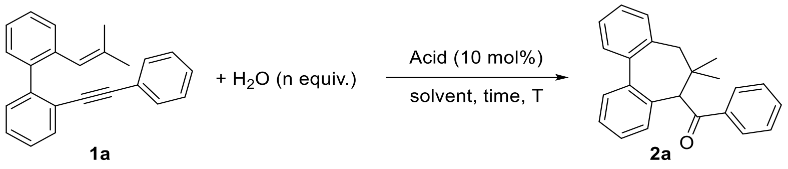

Entry	*[Acid]*	*Water (equiv.)*	*Solvent*	*[M]*	*T (ºC)*	*Time*	*Conversion (yield)*[a]
1	TfOH	1	DCE	0.20	60	24 h	84 (26%)
2	$HBF_4\cdot OEt_2$	1	DCE	0.20	60	24 h	–
3	$TsOH\cdot H_2O$	1	DCE	0.20	60	24 h	–
4	Tf_2NH	1	DCE	0.20	60	6 h	100 (44%)
5	Tf_2NH	1	CF_3Ph	0.20	60	6 h	100 (25%)[b]
6	Tf_2NH	1	DCM	0.20	60	1 h	100 (27%)[b]
7	Tf_2NH	1	Toluene	0.20	60	24 h	–
8	Tf_2NH	1	DMF	0.20	60	24 h	–
9	Tf_2NH	1	THF	0.20	60	24 h	–
10	Tf_2NH	1	$CHCl_3$	0.20	60	5 h	100 (53%)
11	Tf_2NH	1	1,4-dioxane	0.20	60	24 h	100 (50%)
12	Tf_2NH	2	$CHCl_3$	0.20	60	6 h	100 (52%)
13	Tf_2NH	4	$CHCl_3$	0.20	60	6 h	100 (49%)
14	Tf_2NH	1	$CHCl_3$	0.40	60	4 h	100 (32%)[b]
15	Tf_2NH	1	$CHCl_3$	0.15	60	4 h	100 (48%)[b]
16	Tf_2NH	1	$CHCl_3$	0.10	60	5 h	100 (67%)
17	Tf_2NH	1	$CHCl_3$	0.05	60	6 h	100 (72%)
18	Tf_2NH	1	DCE	0.10	60	6 h	100 (54%)[b]
19	Tf_2NH	1	$CHCl_3$	0.05	40	8 h	100 (12%)[b]

[a] Conversion estimated by ^1H NMR (300 MHz); isolated yield in brackets for an experiment conducted with 0.15 mmol of **1a**. [b] The yield was estimated by ^1H NMR spectroscopy using CH_2Br_2 as internal standard.

Table S2. Optimization of the cyclization of 1b with water as nucleophile:

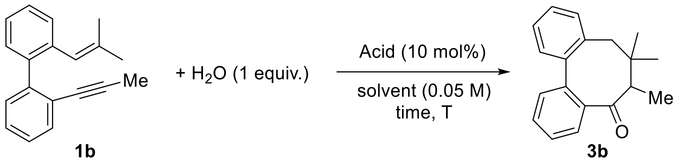

Entry	*[Acid]*	*Solvent*	*T (ºC)*	*Time*	*Conversion (yield)*[a]
1	Tf_2NH	$CHCl_3$	60	12 h	100 (64%)
2	TfOH	$CHCl_3$	60	12 h	100 (95%)
3	TfOH	DCM	60	12 h	100 (80%)
4	TfOH	DCE	60	12 h	100 (99%)
5	TfOH	DCE	40	24 h	100 (94%)
6	TfOH	DCE	RT	48 h	100 (86%)
7[b]	TfOH	DCE	60	12 h	100 (78%)
8	TfOH	DCE	60	2 h	100 (99%)
9	TfOH	EtOAc	60	48 h	100 (88%)
10	TfOH	DMC[c]	60	48 h	100 (96%)

[a] Conversion estimated by ^1H NMR (300 MHz); isolated yield in brackets for an experiment conducted with 0.15 mmol of **1a**. [b] Reaction conducted with 5 mol% of catalyst. [c] Dimethyl carbonate.

Reaction of 1g with water as nucleophile

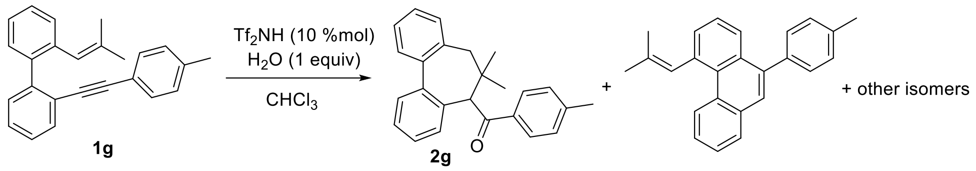

Under the optimized conditions, the reaction of substrate **1g** resulted in the competitive formation of other side products. These subproducts could not be isolated and, therefore, could not be appropriately characterized. We believe that the major byproduct is the phenanthrene depicted in the scheme, likely formed by an initial activation of the alkyne followed by cyclization with the arene. A copy of the ^{1}H-MNR of the mixture (**2g**, phenanthrene and other isomers) with a tentative assignment of key protons of the phenanthrene is included in the spectra appendix. Moreover, all the attempts performed to increase the selectivity to desired cycloadduct **2g** were unsuccessful.

Table S3. Optimization of the cyclization of 1a with 1,2-dimethoxybenzene as nucleophile

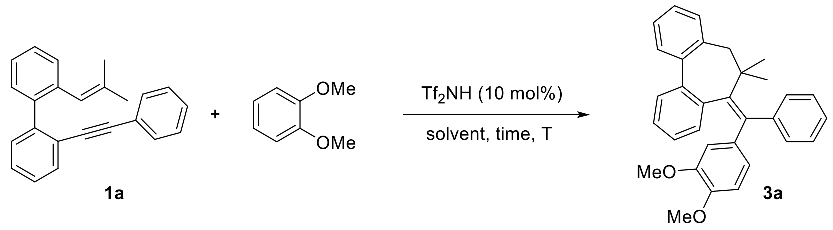

Entry	*Dimethoxybenzene (equiv)*	*Solvent*	*[M]*	*T (ºC)*	*Time*	*Conversion (yield)[a]*
1	1.5	$CHCl_3$	0.05	60	1 h	100 (82%)
2[b]	1.5	$CHCl_3$	0.05	60	1 h	100 (84%)
3	1.5	DCE	0.05	60	1 h	100 (82%)
4	1.5	DCE	0.1	60	30 min	100 (91%)
5	1.1	DCE	0.1	60	30 min	100 (91%)
6	1.1	DCE	0.1	40	30 min	100 (82%)
7	1.1	DCE	0.1	RT	5 h	100 (79%)

[a] Conversion estimated by ^{1}H NMR (300 Hz); isolated yield in brackets for an experiment conducted with 0.15 mmol of **1a**. [b] Conducted in the presence of molecular sieves.

Table S4. Optimization of the cyclization of 1b with 1,2-dimethoxybenzene as nucleophile

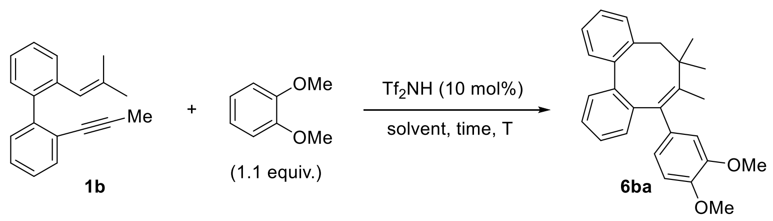

Entry	*Solvent*	*[M]*	*T (ºC)*	*Time*	*Conversion (yield)[a]*
1	DCE	0.05	60	1 h	100[b]
2	DCE	0.05	60	30 min	100 (40%)
3	DCE	0.1	30	1 h	100 (56%)
4	DCE	0.05	30	1 h 45 min	100 (40%)
5	DCE	0.5	30	1 h	100[b]
6	DMC[c]	0.05	30	1 h 45 min	-

[a] Conversion estimated by ^{1}H NMR (300 Hz); isolated yield in brackets for an experiment conducted with 0.12 mmol of **1b**. [b] **6ba** was formed accompanied with various unidentified products. [c] Dimethyl carbonate.

General procedures for the synthesis of 2-alkenyl-2'-alkynyl-1,1'-biphenyls 1:

The starting substrates **1** employed were prepared following two protocols (Methods A and B).

Method A: It involves a Sonogashira and Suzuki couplings and a Wittig reaction.

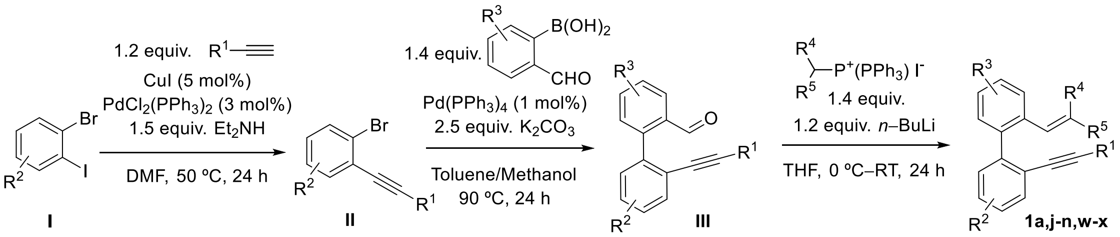

Step 1:[3] In a round bottom flask, the appropiate alkyne (1.2 equiv.) was added to a solution of the corresponding 1-bromo-2-iodobenzene **I** (1 equiv.), Et_2NH (1.5 equiv.), CuI (0.05 equiv.) and $PdCl_2(PPh_3)_2$ (0.3 equiv.) in anhydrous DMF (0.25 M). The resulting mixture was stirred under Ar atmosphere at 50 ºC until complete consumption of the starting iodobenzene **I**, as monitored by TLC. Water and CH_2Cl_2 were added to the cooler reaction mixture. The separated aqueous phase was extracted with CH_2Cl_2 (3 x 20 mL). The combined organic layers were dried over anhydrous Na_2SO_4, filtered and evaporated under reduced pressure. The residue was purified by flash chromatography using mixtures of hexane and ethyl acetate as eluent to obtain the corresponding o-alkynylbromobenzenes **II** (86-99% yields) which were used in the next step.

Step 2:[4] The appropriate boronic acid (1.4 equiv.), $Pd(PPh_3)_4$ (0.01 equiv.) and the corresponding *o*-alkynylbromobenzene **II** obtained in step 1 (1 equiv.) were suspended in toluene (0.5 M). Then methanol (5 mL) and K_2CO_3 (2.5 equiv.) were added and the mixture was stirred thoroughly under Ar atmosphere at 90 ºC. After completion of reaction, toluene was evaporated under reduced pressure and crude product was extracted with EtOAc (3 x 20 mL). The combined organic layers were dried over Na_2SO_4, and solvents were removed under reduced pressure. The residue was purified by flash chromatography using mixtures of hexane and EtOAc as eluents to obtain the biphenylcarbaldehydes **III** (60-99% yields) which were used in the next step.

Step 3:[5] *n*-BuLi (1.2 equiv., 1.6 M in hexanes) was added to a solution of the appropriate phosphonium halide (1.4 equiv.) in THF (0.25 M) at 0 ºC and the resulting mixture was stirred under argon atmosphere for 2 h at RT. The mixture was cooled to 0 ºC, the corresponding carbonyl **III** derivative obtained in step 2 (1 equiv.) was added and the reaction stirred at RT until the aldehyde **III** was consumed as determined by TLC. The resulting mixture was quenched with water and extracted with CH_2Cl_2 (3 x 20 mL). The combined organic layers were dried over anhydrous Na_2SO_4, filtered and evaporated under reduced pressure. The residue was purified by flash chromatography using mixtures of hexane and EtOAc as eluents to obtain the corresponding 2-alkenyl-2'-alkynyl-1,1'-biphenyls **1a,j-n,w-x** (50-99%).

---

[3] a) Guilarte, V.; Fernández-Rodríguez, M. A.; García-García, P.; Hernández, E.; Sanz, R. *Org. Lett.* **2011**, *13*, 5100−5103. b) Verma, A. K.; Kesharwani, T.; Singh, J.; Tandon, V.; Larock, R. C. *Angew. Chem. Int. Ed.* **2009**, *48,* 1138-1143.
[4] a) Bera, K.; Sarkar, S.; Jalal, S.; Jana, U. *J. Org. Chem.* **2012**, *77*, 8780-8786. b) Naveen, K.; Perumal, P. T.; Cho, D. *Org. Lett.* **2019**, *21,* 4350–4354.
[5] Guo, W.; Monge-Marcet, A.; Cattoen, X.; Shafir, A.; Pleixats, R. *Reactive & Functional Polymers.* **2013**, *73,* 192-199.

Method B: It involves a deprotection step and a Sonogashira coupling from silyl derivative **1-TIPS** obtained following Method A.

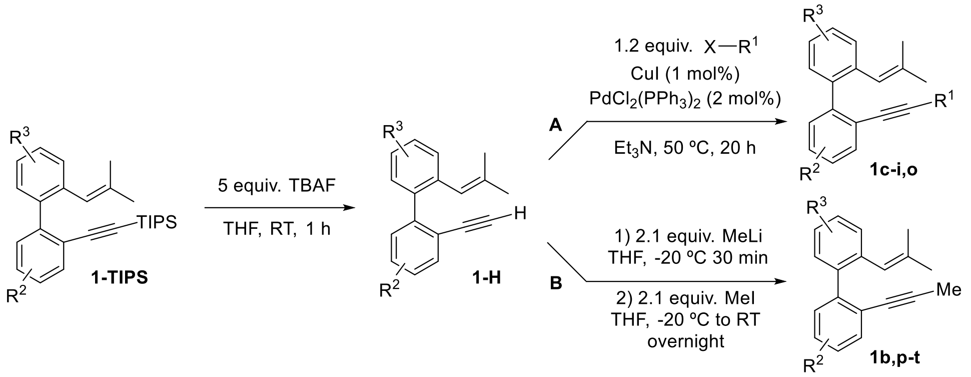

Step 1: The corresponding sylilated trienyne **1-TIPS** (1 equiv.) prepared following Method A was dissolved in THF (0.1 M) at 0 ºC and TBAF (5 equiv., 1.0 M in THF) was added. The reaction mixture was stirred for 1 hour at RT and quenched with water (10 mL/mmol substrate). The solution was concentrated, extracted with diethyl ether, and chromatographed on a silica column using hexane as eluent to give terminal trienynes **1-H**.

Step 2A: In a round bottom flask, the appropriate haloarene (1.2 equiv.) was added to a solution of **1-H** (1 equiv.), CuI (0.01 equiv., 1 mol%), $PdCl_2(PPh_3)_2$ (0.02 equiv., 2 mol%) in anhydrous Et_3N (0.2 M). The resulting mixture was stirred under Ar atmosphere at 50 ºC until complete consumption of the acetylene, as monitored by TLC. Water and CH_2Cl_2 were added to the cooler reaction mixture. The separated aqueous phase was extracted with CH_2Cl_2 (3 x 20 mL) and the combined organic layers were dried over anhydrous Na_2SO_4, filtered and evaporated under reduced pressure. The residue was purified by flash chromatography using mixtures of hexane and EtOAc as eluents to obtain the corresponding 2-alkenyl-2'-alkynyl-1,1'-biphenyls **1c-i,o** (84-99%).

Step 2B:[6] The appropriate terminal-alkyne derivative **1-H** was placed in a schlenk tube under Ar atmosphere and was dissolved in dry THF (0.14 M). The solution was cooled to –20 ºC and MeLi (2.1 equiv, 1.6 M in diethylether) was added dropwise. After 30 min, MeI (2.1 equiv.) was added dropwise and the resulting mixture was stirred and allowed to reach RT overnight until complete consumption of starting material. Water was added to the reaction mixture, the aqueous phase was extracted with hexane (3 x 20 mL) and the combined organic layers were dried over anhydrous Na_2SO_4, filtered and evaporated under reduced pressure. The residue was purified by flash chromatography using mixtures of hexane and EtOAc as eluents to obtain the corresponding 2-alkenyl-2'-alkynyl-1,1'-biphenyls **1b,p-t** (65-89%).

Synthesis of 2'-(4-Hydroxybut-1-yn-1-yl)-2-(2-methylprop-1-en-1-yl)-1,1'-biphenyl (1u): Over a solution of **1a-H** (100 mg, 0.43 mmol, 1.5 equiv.) in dry THF (0.1 mL) at –78 ºC, under Ar atmosphere in a Schlenk flask, was added BuLi (0.43 mmol, 1.6M, 1.5 equiv.) dropwise. The mixture was stirred 10 min before adding $BF_3 \cdot OEt_3$ (0.1 mL) and the stirring continued 10 min. Then, ethylene oxide (0.29 mmol, 2.5 M in THF, 1 equiv.) was added dropwise. The reaction was stirred 1.5 h, allowing to reach RT. After completion, it was quenched with ammonium chloride aqueous solution and the reaction crude was extracted with DCM (10 mL x 3). The organic fraction was dried over magnesium sulfate, the solvent was removed under reduced pressure and the crude reaction mixture was purified by flash chromatography on silica gel, using a mixture of Hexane/EtOAc 9:1 as eluent to give enyne **1u** as colorless oil (73.5 mg, 0.27 mmol, 68%).

---

[6] Weiss, H.; Touchette, K.; Angell, S.; Khan, J. *Org. Biomol. Chem.* **2003**, *1*, 2152-2156.

Synthesis of 2'-(3-(3-methoxyphenyl)prop-1-yn-1-yl)-2-(2-methylprop-1-en-1-yl)-1,1'-biphenyl (1v): In a Schlenk flask, after several vacuum-argon cycles, was added CuI (17.5 mg, 0.092 mmol, 0.1 equiv.), LiHMDS (184.1 mg, 1.10 mmol, 1.2 equiv.) and a solution of **1a-H** (213 mg, 0.92 mmol, 1.5 equiv.) in dry toluene (2.6 mL). Into this mixture was added dropwise 3-methoxybenzyl bromide (0.1 mL, 0.61 mmol, 1 equiv.) and the reaction was stirred 24 h at 90 ºC. After completion, the solvent was removed under reduced pressure and the crude reaction mixture was directly purified by flash chromatography on silica gel, using Hexane/EtOAc 40:1 as eluent to give enyne **1v** as yellow oil (72.9 mg, 0.21 mmol, 22%).

Characterization data of unknown biphenyl embedded trienynes 1 and their precursors:

1-Bromo-5-chloro-4-triisopropylsilylethynylbenzene (IIq)

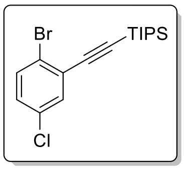

Obtained as colorless oil (670 mg, 1.80 mmol, 99%) from **Iq** (500 mg, 1.60 mmol) following method A. R_f = 0.60 (Hexane).

^{1}H-NMR (300 MHz, CDCl$_3$) δ (ppm) 7.50–7.44 (m, 2H), 7.12 (dd, *J* = 8.5, 2.5 Hz, 1H), 1.21 (bs, 18H), 1.14 (bs, 3H).

^{13}C-NMR (75 MHz, CDCl$_3$) δ (ppm) 133.4 (CH), 133.3 (CH), 132.9 (C), 129.5 (CH), 127.2 (C), 123.8 (C), 103.6 (C), 98.0 (C), 18.9 (4 x CH_3), 18.8 (2 x CH_3), 11.64 (2 x CH), 11.58 (CH).

HRMS (ESI-TOF) m/z: $[M+H]^+$ calculated for $C_{17}H_{25}BrClSi$ 371.0592. Found 371.0595.

1-Bromo-4-triisopropylsilylethynyl-3-methoxybenzene (IIs)

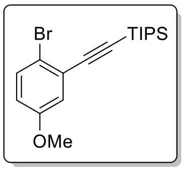

Obtained as colorless oil (448 mg, 1.22 mmol, 94%) from **Is** (400 mg, 1.30 mmol) following method A. R_f = 0.28 (Hexane).

^{1}H-NMR (300 MHz, CDCl$_3$) δ (ppm) 7.44 (d, *J* = 8.8 Hz, 1H), 7.05 (d, *J* = 3.1 Hz, 1H), 6.73 (dd, *J* = 8.8, 3.1 Hz, 1H), 3.79 (s, 3H), 1.21 (bs, 21H).

^{13}C-NMR (75 MHz, CDCl$_3$) δ (ppm) 158.3 (C), 133.0 (CH), 126.1 (C), 118.5 (CH), 116.5 (C), 116.3 (CH), 104.9 (C), 95.9 (C), 55.6 (CH_3), 18.9 (6 x CH_3), 11.6 (3 x CH).

HRMS (ESI-TOF) m/z: $[M+Na]^+$ calculated for $C_{18}H_{27}BrNaOSi$ 389.0907. Found 389.0914.

5-Fluoro-2’-ethynylphenyl-[1,1’-biphenyl]-2-carbaldehyde (IIIL)

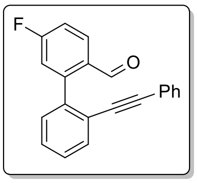

Obtained as yellow oil (258 mg, 0.85 mmol, 72%) from **IIL** (306 mg, 1.19 mmol) following method B. R_f = 0.33 (Hexane/EtOAc 40:1).

^{1}H-NMR (300 MHz, CDCl$_3$) δ (ppm) 9.89 (s, 1H), 8.16 (dd, *J* = 8.7, 6.0 Hz, 1H), 7.70–7.65 (m, 1H), 7.48–7.37 (m, 4H), 7.30–7.14 (m, 6H).

^{13}C-NMR (75 MHz, CDCl$_3$) δ (ppm) 190.1 (CH), 165.5 (d, $J_{C\text{-}F}$ = 256 Hz, C), 147.0 (C), 138.9 (d, $J_{C\text{-}F}$ = 1.9 Hz, C), 132.1 (CH), 131.3 (2 x CH), 130.9 (d, $J_{C\text{-}F}$ = 2.5 Hz, C), 130.0 (CH), 129.8 (d, $J_{C\text{-}F}$ = 10.2 Hz, CH), 128.7 (CH), 128.61 (CH), 128.58 (CH), 128.3 (2 x CH), 123.5 (C), 122.5 (C), 118.1 (d, $J_{C\text{-}F}$ = 22.5 Hz, CH), 115.6 (d, $J_{C\text{-}F}$ = 22.5 Hz, CH), 94.1 (C), 87.8 (C). **^{19}F-NMR (283 MHz, CDCl$_3$)** δ (ppm) –103.8.

HRMS (ESI-TOF) m/z: $[M+H]^+$ calculated for $C_{21}H_{14}FO$ 301.1023. Found 301.1017.

5-Chloro-2'-((triisopropylsilyl)ethynyl)-[1,1'-biphenyl]-2-carbaldehyde (IIIp)

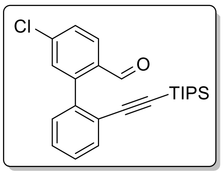

Obtained as colorless oil (346 mg, 0.87 mmol, 55%) from **IIp** (500 mg, 1.59 mmol) following method A. R_f = 0.22 (Hexane/Toluene 10:1).

^{1}H-NMR (300 MHz, CDCl$_3$) δ (ppm) 9.76 (s, 1H), 7.95 (d, *J* = 8.3 Hz, 1H), 7.63–7.60 (m, 1H), 7.47–7.39 (m, 4H), 7.30–7.27 (m, 1H), 0.95 (bs, 21H).

^{13}C-NMR (75 MHz, CDCl$_3$) δ (ppm) 190.3 (C=O), 146.0 (C), 139.7 (C), 139.1 (C), 132.9 (CH), 132.5 (C), 131.1 (CH), 130.0 (CH), 128.6 (CH), 128.5 (CH), 128.44 (CH), 128.37 (CH), 123.8 (C), 104.8 (C), 96.5 (C), 18.7 (6 x CH_3), 11.3 (3 x CH).

HRMS (ESI-TOF) m/z: $[M+H]^+$ calculated for $C_{24}H_{30}ClOSi$ 397.1749. Found 397.1755.

4'-Chloro-2'-((triisopropylsilyl)ethynyl)-[1,1'-biphenyl]-2-carbaldehyde (IIIq)

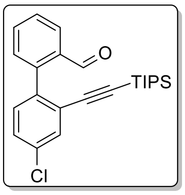

Obtained as colorless oil (313 mg, 0.79 mmol, 44%) from **IIq** (670 mg, 1.80 mmol) following method A. R_f = 0.25 (Hexane/EtOAc 40:1).

^{1}H-NMR (300 MHz, $CDCl_3$) δ (ppm) 9.83 (s, 1H), 8.00 (dd, *J* = 7.7, 1.5 Hz, 1H), 7.61–7.56 (m, 2H), 7.51–7.32 (m, 1H), 7.40–7.32 (m, 2H), 7.24 (d, *J* = 8.2 Hz, 1H), 0.92 (bs, 21H).

^{13}C-NMR (75 MHz, $CDCl_3$) δ (ppm) 191.0 (C=O), 143.2 (C), 139.2 (C), 134.0 (C), 133.9 (C), 133.5 (CH), 132.3 (CH), 131.2 (CH), 131.0 (CH), 128.5 (CH), 128.3 (CH), 127.4 (CH), 125.4 (C), 103.7 (C), 97.4 (C), 18.6 (6 x CH_3), 11.3 (3 x CH).

HRMS (ESI-TOF) m/z: $[M+H]^+$ calculated for $C_{24}H_{30}ClOSi$ 397.1749. Found 397.1761.

4-Methoxy-2'-((triisopropylsilyl)ethynyl)-[1,1'-biphenyl]-2-carbaldehyde (IIIr)

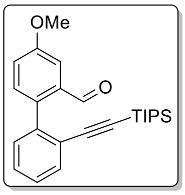

Obtained as yellow oil (220 mg, 0.56 mmol, 59%) from **IIr** (300 mg, 0.95 mmol) following method A. R_f = 0.32 (Hexane/EtOAc 40:1).

^{1}H-NMR (300 MHz, $CDCl_3$) δ (ppm) 9.80 (s, 1H), 7.61–7.57 (m, 1H), 7.51 (d, *J* = 2.8 Hz, 1H), 7.40–7.33 (m, 2H), 7.33–7.25 (m, 2H), 7.16 (dd, *J* = 8.5, 2.8 Hz, 1H), 3.88 (s, 3H), 0.92 (bs, 21H).

^{13}C-NMR (75 MHz, $CDCl_3$) δ (ppm) 191.8 (C=O), 159.5 (C), 140.5 (C), 137.8 (C), 135.1 (C), 132.8 (CH), 132.5 (CH), 130.6 (CH), 128.4 (CH), 127.9 (CH), 124.3 (C), 121.3 (CH), 109.6 (CH), 105.4 (C), 95.6 (C), 55.8 (CH_3), 18.6 (6 x CH_3), 11.3 (3 x CH).

HRMS (ESI-TOF) m/z: $[M+H]^+$ calculated for $C_{25}H_{33}O_2Si$ 393.2244. Found 393.2250.

4'-Methoxy-2'-((triisopropylsilyl)ethynyl)-[1,1'-biphenyl]-2-carbaldehyde (IIIs)

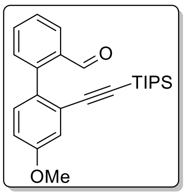

Obtained as yellow oil (335 mg, 0.85 mmol, 70%) from **IIs** (448 mg, 1.22 mmol) following method A. R_f = 0.30 (Hexane/EtOAc 40:1).

^{1}H-NMR (300 MHz, $CDCl_3$) δ (ppm) 9.86 (s, 1H), 7.98 (d, *J* = 7.4 Hz, 1H), 7.56 (td, *J* = 7.4, 1.5 Hz, 1H), 7.47–7.38 (m, 1H), 7.34 (d, *J* = 7.4 Hz, 1H), 7.19 (d, *J* = 8.5 Hz, 1H), 7.10 (d, *J* = 2.8 Hz, 1H), 6.93 (dd, *J* = 8.5, 2.8 Hz, 1H), 3.83 (s, 3H), 0.90 (s, 21H).

^{13}C-NMR (75 MHz, $CDCl_3$) δ (ppm) 191.8 (C=O), 159.1 (C), 144.4 (C), 134.3 (C), 133.4 (CH), 133.1 (C), 131.4 (CH), 131.3 (CH), 127.8 (CH), 127.0 (CH), 124.8 (C), 117.3 (CH), 114.9 (CH), 105.2 (C), 95.3 (C), 55.4 (CH_3), 18.5 (6 x CH_3), 11.1 (3 x CH).

HRMS (ESI-TOF) m/z: $[M+H]^+$ calculated for $C_{25}H_{33}O_2Si$ 393.2244. Found 393.2255.

2'-Triisopropylsilylethynyl-5-chloro-2-(2-methylprop-1-en-1-yl)-1,1'-biphenyl (1p-TIPS)

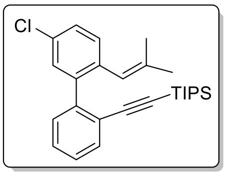

Obtained as colorless oil (200 mg, 0.47 mmol, 54%) from **IIIp** (346 mg, 0.87 mmol) following method A. R_f = 0.30 (Hexane).

^{1}H-NMR (300 MHz, $CDCl_3$) δ (ppm) 7.61–7.58 (m, 1H), 7.36 (d, *J* = 2.2 Hz, 1H), 7.32–7.25 (m, 3H), 7.22 (d, *J* = 8.2 Hz, 1H), 7.24–7.20 (m, 1H), 6.00 (bs, 1H), 1.73 (d, *J* = 1.5 Hz, 3H), 1.70 (d, *J* = 1.5 Hz, 3H), 1.03 (s, 21H).

^{13}C-NMR (75 MHz, $CDCl_3$) δ (ppm) 143.2 (C), 141.8 (C), 135.74 (C), 135.71 (C), 132.9 (CH), 131.4 (C), 130.9 (CH), 130.3 (CH), 130.1 (CH), 127.7 (CH), 127.08 (CH), 127.06 (CH), 123.5 (CH), 122.8 (C), 105.7 (C), 94.3 (C), 26.4 (CH_3), 19.7 (CH_3), 18.8 (6 x CH_3), 11.5 (3 x CH).

HRMS (ESI-TOF) m/z: $[M+H]^+$ calculated for $C_{27}H_{36}ClSi$ 423.2269. Found 423.2267.

2'-Triisopropylsilylethynyl-4'-chloro-2-(2-methylprop-1-en-1-yl)-1,1'-biphenyl (1q-TIPS)

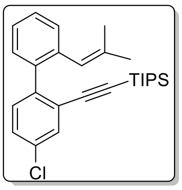

Obtained as colorless oil (266 mg, 0.63 mmol, 80%) from **IIIq** (313 mg, 0.79 mmol) following method A. R_f = 0.48 (Hexane).

¹H-NMR (300 MHz, CDCl₃) δ (ppm) 7.61–7.58 (m, 1H), 7.36–7.22 (m, 5H), 7.13 (d, *J* = 7.8 Hz, 1H), 6.10 (bs, 1H), 1.77 (s, 3H), 1.71 (s, 3H), 1.02 (bs, 21H).

^{13}C-NMR (75 MHz, $CDCl_3$) δ (ppm) 143.1 (C), 139.1 (C), 137.2 (C), 135.2 (C), 132.4 (CH), 132.3 (C), 131.6 (CH), 130.2 (CH), 129.7 (CH), 127.9 (CH), 127.3 (CH), 125.9 (CH), 124.6 (C), 124.3 (CH), 104.8 (C), 95.2 (C), 26.3 (CH_3), 19.6 (CH_3), 18.8 (6 x CH_3), 11.4 (3 x CH).

HRMS (ESI-TOF) m/z: $[M+H]^+$ calculated for $C_{27}H_{36}ClSi$ 423.2269. Found 423.2269.

2'-Triisopropylsilylethynyl-4-methoxy-2-(2-methylprop-1-en-1-yl)-1,1'-biphenyl (1r-TIPS)

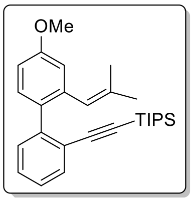

Obtained as colorless oil (196 mg, 0.47 mmol, 84%) from **IIIr** (220 mg, 0.56 mmol) following method A. R_f = 0.62 (Hexane/EtOAc 40:1).

^{1}H-NMR (300 MHz, $CDCl_3$) δ (ppm) 7.59–7.54 (m, 1H), 7.32–7.21 (m, 3H), 7.20–7.15 (m, 1H), 6.85 (dd, *J* = 2.8, 0.6 Hz, 1H), 6.83–6.76 (m, 1H), 6.09 (bs, 1H), 3.86 (s, 3H), 1.73 (bs, 3H), 1.70 (bs, 3H), 1.02 (bs, 21H).

^{13}C-NMR (75 MHz, $CDCl_3$) δ (ppm) 158.5 (C), 144.4 (C), 138.5 (C), 135.1 (C), 133.1 (C), 132.8 (CH), 131.4 (CH), 130.7 (CH), 127.6 (CH), 126.4 (CH), 124.6 (CH), 123.2 (C), 115.1 (CH), 111.4 (CH), 106.5 (C), 93.3 (C), 55.5 (CH_3), 26.3 (CH_3), 19.7 (CH_3), 18.8 (6 x CH_3), 11.5 (3 x CH).

HRMS (ESI-TOF) m/z: $[M+H]^+$ calculated for $C_{28}H_{39}OSi$ 419.2765. Found 419.2774.

2'-Triisopropylsilylethynyl-4'-methoxy-2-(2-methylprop-1-en-1-yl)-1,1'-biphenyl (1s-TIPS)

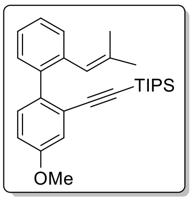

Obtained as yellow oil (299 mg, 0.72 mmol, 84%) from **IIIs** (335 mg, 0.85 mmol) following method A. R_f = 0.18 (Hexane).

^{1}H-NMR (300 MHz, $CDCl_3$) δ (ppm) 7.38 (d, *J* = 6.9 Hz, 1H), 7.35–7.21 (m, 3H), 7.19–7.10 (m, 2H), 6.92 (dt, *J* = 8.5, 2.4 Hz, 1H), 6.14 (s, 1H), 3.88 (s, 3H), 1.78 (s, 3H), 1.74 (s, 3H), 1.02 (bs, 21H).

^{13}C-NMR (75 MHz, $CDCl_3$) δ (ppm) 158.1 (C), 140.0 (C), 137.5 (2 x C), 134.7 (C), 131.6 (CH), 130.8 (CH), 129.6 (CH), 126.8 (CH), 125.8 (CH), 124.8 (CH), 123.7 (C), 117.1 (CH), 114.5 (CH), 106.3 (C), 93.3 (C), 55.4 (CH_3), 26.3 (CH_3), 19.5 (CH_3), 18.6 (6 x CH_3), 11.3 (3 x CH).

HRMS (ESI-TOF) m/z: $[M+H]^+$ calculated for $C_{28}H_{39}OSi$ 419.2765. Found 419.2778.

5-Chloro-2'-ethynyl-2-(2-methylprop-1-en-1-yl)-1,1'-biphenyl (1p-H)

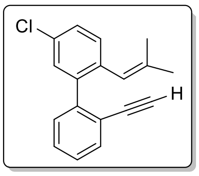

Obtained as yellow oil (113 mg, 0.42 mmol, 90%) from **1p-TIPS** (200 mg, 0.47 mmol) following method B. R_f = 0.28 (Hexane).

^{1}H-NMR (300 MHz, $CDCl_3$) δ (ppm) 7.58 (d, *J* = 7.9 Hz, 1H), 7.40–7.28 (m, 4H), 7.27–7.17 (m, 2H), 5.91 (s, 1H), 2.96 (s, 1H), 1.74 (s, 3H), 1.73 (s, 3H).

^{13}C-NMR (75 MHz, $CDCl_3$) δ (ppm) 143.3 (C), 141.4 (C), 136.1 (C), 136.0 (C), 133.0 (CH), 131.3 (C), 131.0 (CH), 130.0 (CH), 129.9 (CH), 128.4 (CH), 127.3 (CH), 127.2 (CH), 123.3 (CH), 121.7 (C), 82.6 (C), 80.3 (CH), 26.3 (CH_3), 19.5 (CH_3).

HRMS (ESI-TOF) m/z: $[M+H]^+$ calculated for $C_{18}H_{16}Cl$ 267.0935. Found 267.0937.

4'-Chloro-2'-ethynyl-2-(2-methylprop-1-en-1-yl)-1,1'-biphenyl (1q-H)

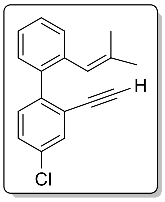

Obtained as colorless oil (174 mg, 0.65 mmol, 99%) from **1q-TIPS** (313 mg, 0.79 mmol) following method B. R_f = 0.40 (Hexane).

^{1}H-NMR (300 MHz, CDCl$_3$) δ (ppm) 7.62–7.60 (m, 1H), 7.42–7.29 (m, 5H), 7.20–7.16 (m, 1H) 6.00 (bs, 1H), 2.98 (s, 1H), 1.81 (s, 3H), 1.71 (s, 3H).

^{13}C-NMR (75 MHz, CDCl$_3$) δ (ppm) 143.1 (C), 138.7 (C), 137.5 (C), 135.6 (C), 132.6 (CH), 132.5 (C), 131.6 (CH), 129.9 (CH), 129.8 (CH), 128.5 (CH), 127.5 (CH), 125.9 (CH), 124.1 (CH), 123.4 (C), 81.7 (C), 80.9 (CH), 26.3 (CH$_3$), 19.5 (CH$_3$).

HRMS (ESI-TOF) m/z: [M+H]$^+$ calculated for $C_{18}H_{16}Cl$ 267.0935. Found 267.0939.

2'-Ethynyl-4-methoxy-2-(2-methylprop-1-en-1-yl)-1,1'-biphenyl (1r-H)

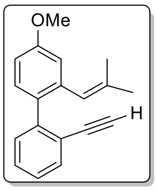

Obtained as colorless oil (102 mg, 0.38 mmol, 83%) from **1r-TIPS** (196.2 mg, 0.47 mmol) following method B. R_f = 0.58 (Hexane/EtOAc 40:1).

^{1}H-NMR (300 MHz, CDCl$_3$) δ (ppm) 7.58 (dd, J = 7.2, 1.2 Hz, 1H), 7.36–7.18 (m, 4H), 6.88–6.83 (m, 2H), 6.00 (bs, 1H), 3.87 (s, 3H), 2.95 (s, 1H), 1.75 (bs, 6H).

^{13}C-NMR (75 MHz, CDCl$_3$) δ (ppm) 158.5 (C), 144.4 (C), 138.7 (C), 135.4 (C), 133.0 (CH), 132.6 (C), 131.1 (CH), 130.7 (CH), 128.1 (CH), 126.5 (CH), 124.5 (CH), 121.9 (C), 115.1 (CH), 111.2 (CH), 83.3 (C), 79.7 (CH), 55.3 (CH$_3$), 26.3 (CH$_3$), 19.6 (CH$_3$).

HRMS (ESI-TOF) m/z: [M+H]$^+$ calculated for $C_{19}H_{19}O$ 263.1430. Found 263.1434.

2'-Ethynyl-4'-methoxy-2'-(2-methylprop-1-en-1-yl)-1,1'-biphenyl (1s-H)

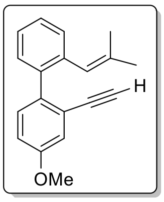

Obtained as white solid (160 mg, 0.61 mmol, 85%) from **1s-TIPS** (299 mg, 0.72 mmol) following method B. R_f = 0.23 (Hexane). M.p.: 108–110 ºC.

^{1}H-NMR (300 MHz, CDCl$_3$) δ (ppm) 7.36–7.24 (m, 4H), 7.14 (d, J = 6.0 MHz, 1H), 7.12 (s, 1H), 6.94 (dd, J = 8.8, 2.6 Hz, 1H), 6.00 (bs, 1H), 3.85 (s, 3H), 2.91 (s, 1H), 1.77 (s, 3H), 1.75 (s, 3H).

^{13}C-NMR (75 MHz, CDCl$_3$) δ (ppm) 158.1 (C), 139.6 (C), 137.8 (C), 137.4 (C), 135.1 (C), 131.6 (CH), 130.4 (CH), 129.8 (CH), 127.1 (CH), 125.8 (CH), 124.7 (CH), 122.5 (C), 117.3 (CH), 115.1 (CH), 83.0 (C), 79.5 (CH), 55.4 (CH$_3$), 26.3 (CH$_3$), 19.4 (CH$_3$).

HRMS (ESI-TOF) m/z: [M+H]$^+$ calculated for $C_{19}H_{19}O$ 263.1430. Found 263.1434.

2'-Ethynyl-4',6'-dimethoxy-2'-(2-(methylprop-1-en-1-yl)-5-chloro)-1,1'-biphenyl (1o-H)

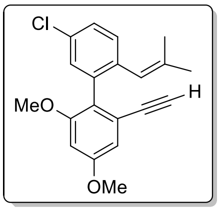

Obtained as yellow oil (148 mg, 0.45 mmol, 26%) from appropriated **1o-TIPS** precursor (800 mg, 1.75 mmol) following method B in a one-pot protocol. The compound could not be completely purified and was used in the next step. R_f = 0.23 (Hexane/EtOAc 40:1).

^{1}H-NMR (500 MHz, CDCl$_3$) δ (ppm) 7.29–7.27 (m, 1H), 7.23–7.18 (m, 2H), 6.71 (s, 1H), 6.53 (s, 1H), 5.83 (s, 1H), 3.85 (s, 3H), 3.67 (s, 3H), 2.88 (s, 1H), 1.71 (s, 3H), 1.69 (s, 3H).

^{13}C-NMR (125 MHz, CDCl$_3$) δ (ppm) 159.8 (C), 157.8 (C), 137.8 (C), 137.4 (C), 135.5 (C), 131.2 (C), 130.8 (CH), 130.7 (CH), 127.1 (CH), 125.7 (C), 123.4 (CH), 123.3 (C), 108.2 (CH), 100.3 (CH), 82.5 (C), 80.1 (CH), 55.8 (CH$_3$), 55.6 (CH$_3$), 26.2 (CH$_3$), 19.3 (CH$_3$).

HRMS (ESI-TOF) m/z: [M+H]$^+$ calculated for $C_{20}H_{19}ClO_2$ 327.1146. Found 327.1147.

2'-((2-Bromophenyl)ethynyl)-2-(2-methylprop-1-en-1-yl)-1,1'-biphenyl (1e)

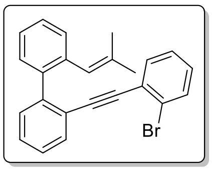

Obtained as colorless oil (141 mg, 0.36 mmol, 84%) from **1a-H** (100 mg, 0.43 mmol) following method B. R_f = 0.40 (Hexane).

^{1}H-NMR (300 MHz, $CDCl_3$) δ (ppm) 7.69–7.66 (m, 1H), 7.52–7.48 (m, 1H), 7.42–7.30 (m, 5H), 7.29–7.24 (m, 2H), 7.24–7.15 (m, 2H), 7.11–7.04 (m, 1H), 6.03 (bs, 1H), 1.74 (s, 3H), 1.69 (s, 3H).

^{13}C-NMR (75 MHz, $CDCl_3$) δ (ppm) 144.4 (C), 140.1 (C), 137.5 (C), 135.1 (C), 133.2 (CH), 132.4 (CH), 132.2 (CH), 130.3 (CH), 130.2 (CH), 129.7 (CH), 129.0 (CH), 128.1 (CH), 127.1 (CH), 126.83 (CH), 126.76 (CH), 125.8 (CH), 125.7 (C), 125.1 (C), 124.4 (CH), 122.5 (C), 93.8 (C), 90.6 (C), 26.4 (CH_3), 19.7 (CH_3).

HRMS (ESI-TOF) m/z: $[M+H]^+$ calculated for $C_{24}H_{20}Br$ 387.0743. Found 387.0741.

2-(2-Methylprop-1-en-1-yl)-2'-(*m*-methoxyethynyl)-1,1'-biphenyl (1f)

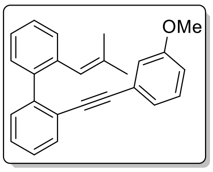

Obtained as colorless oil (147 mg, 0.43 mmol, 99%) from **1a-H** (100 mg, 0.43 mmol) following method B. R_f = 0.33 (Hexane/EtOAc 40:1).

^{1}H-NMR (300 MHz, $CDCl_3$) δ (ppm) 7.67–7.62 (m, 1H), 7.46–7.33 (m, 6H), 7.33–7.27 (m, 1H), 7.18 (t, J = 7.9 Hz, 1H), 6.86–6.82 (m, 2H), 6.72 (bs, 1H), 6.07 (bs, 1H), 3.77 (s, 3H), 1.75 (s, 3H), 1.72 (s, 3H).

^{13}C-NMR (75 MHz, $CDCl_3$) δ (ppm) 159.2 (C), 144.6 (C), 140.4 (C), 137.7 (C), 135.2 (C), 131.9 (CH), 130.4 (CH), 130.3 (CH), 129.7 (CH), 129.3 (CH), 127.9 (CH), 127.1 (CH), 126.9 (CH), 125.7 (CH), 124.6 (C), 124.5 (CH), 123.9 (CH), 122.8 (C), 116.2 (CH), 114.8 (CH), 92.4 (C), 89.3 (C), 55.3 (CH_3), 26.3 (CH_3), 19.6 (CH_3).

HRMS (ESI-TOF) m/z: $[M+H]^+$ calculated for $C_{25}H_{23}O$ 339.1743. Found 339.1734.

2'-(1-Naphtylethynyl)-2'-(2-methylprop-1-en-1-yl)-1,1'-biphenyl (1h)

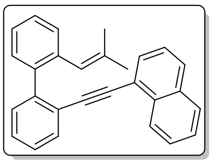

Obtained as white solid (280 mg, 0.78 mmol, 90%) from **1a-H** (200 mg, 0.86 mmol) following method B. R_f = 0.27 (Hexane). M.p.: 118–120 ºC.

^{1}H-NMR (300 MHz, $CDCl_3$) δ (ppm) 7.83–7.74 (m, 3H), 7.58–7.31 (m, 12H), 6.07 (s, 1H), 1.71 (s, 3H), 1.65 (s, 3H).

^{13}C-NMR (75 MHz, $CDCl_3$) δ (ppm) 144.6 (C), 140.7 (C), 137.8 (C), 135.5 (C), 133.3 (C), 133.1 (C), 132.2 (CH), 130.3 (CH), 130.2 (CH), 130.0 (CH), 129.9 (CH), 128.5 (CH), 128.04 (CH), 127.95 (CH), 127.3 (CH), 127.1 (CH), 126.7 (CH), 126.6 (CH), 126.3 (CH), 126.1 (CH), 125.2 (CH), 124.3 (CH), 123.2 (C), 121.3 (C), 94.2 (C), 90.6 (C), 26.3 (CH_3), 19.5 (CH_3).

HRMS (ESI-TOF) m/z: $[M+H]^+$ calculated for $C_{28}H_{23}$ 359.1794. Found 359.1788.

5-Fluoro-2'-Ethynylphenyl-2-(2-(methylprop-1-en-1-yl)-1,1'-biphenyl (1l)

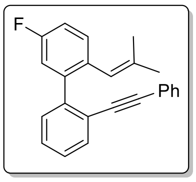

Obtained as yellow oil (211 mg, 0.64 mmol, 76%) from **IIIL** (258 mg, 0.85 mmol) following method A. R_f = 0.28 (Hexane).

^{1}H-NMR (300 MHz, $CDCl_3$) δ (ppm) 7.67–7.60 (m, 1H), 7.38–7.23 (m, 9H), 7.19 (dd, J = 9.6, 2.7 Hz, 1H), 7.09 (td, J = 8.5, 2.8 Hz, 1H), 6.00 (bs, 1H), 1.72 (s, 3H), 1.67 (s, 3H).

^{13}C-NMR (75 MHz, $CDCl_3$) δ (ppm) 160.9 (d, $J_{C\text{-}F}$ = 245.0 Hz, C), 143.2 (C), 142.1 (d, $J_{C\text{-}F}$ = 7.8 Hz, C), 135.4 (C), 133.8 (d, $J_{C\text{-}F}$ = 3.4 Hz, C), 132.1 (CH), 131.5 (2 x CH), 131.2 (d, $J_{C\text{-}F}$ = 7.8 Hz, CH), 130.1 (CH), 128.3 (2 x CH), 128.2 (CH), 127.9 (CH), 127.4 (CH), 123.5 (CH), 123.4 (C), 122.8 (C), 117.0 (d, $J_{C\text{-}F}$ = 21.4 Hz, CH), 114.0 (d, $J_{C\text{-}F}$ = 20.7 Hz, CH), 92.8 (C), 88.9 (C), 26.1 (CH_3), 19.5 (CH_3).

^{19}F-NMR (283 MHz, $CDCl_3$) δ (ppm) –117.2 (m).

HRMS (ESI-TOF) m/z: $[M+H]^+$ calculated for $C_{24}H_{20}F$ 327.1543. Found 327.1538.

2'-Ethynylphenyl-4',6'-dimethoxy-2'-(2-(methylprop-1-en-1-yl)-5-chloro)-1,1'-biphenyl (1o)

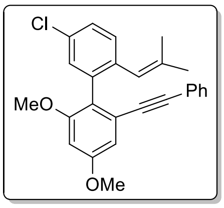

Obtained as yellow oil (75 mg, 0.18 mmol, 88%) from **1o-H** (70 mg, 0.21 mmol) following method B. R$_f$ = 0.28 (Hexane/EtOAc 40:1).

^{1}H-NMR (500 MHz, CDCl$_3$) δ (ppm) 7.36–7.33 (m, 2H), 7.29–7.24 (m, 4H), 7.22–7.19 (m, 2H), 6.77 (d, *J* = 2.4 Hz, 1H), 6.55 (d, *J* = 2.4 Hz, 1H), 5.91 (bs, 1H), 3.89 (s, 3H), 3.71 (s, 3H), 1.72 (s, 3H), 1.67 (s, 3H).

^{13}C-NMR (125 MHz, CDCl$_3$) δ (ppm) 159.9 (C), 157.8 (C), 138.3 (C), 137.6 (C), 135.4 (C), 131.5 (2 x CH), 131.1 (C), 131.0 (CH), 130.6 (CH), 128.3 (2 x CH), 128.2 (CH), 126.9 (CH), 125.3 (C), 124.5 (C), 123.4 (CH), 123.3 (C), 107.2 (CH), 99.9 (CH), 92.8 (C), 88.8 (C), 55.8 (CH$_3$), 55.6 (CH$_3$), 26.2 (CH$_3$), 19.4 (CH$_3$).

HRMS (ESI-TOF) m/z: [M+H]$^+$ calculated for C$_{26}$H$_{23}$ClO$_2$ 403.1459. Found 403.1472.

5-Chloro-2-(2-methylprop-1-en-1-yl)-2'-(prop-1-yn-1-yl)-1,1'-biphenyl (1p)

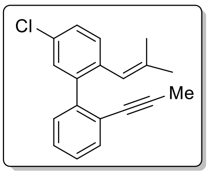

Obtained as colorless oil (99 mg, 0.35 mmol, 86%) from **1p-H** (110 mg, 0.41 mmol) following method B. R$_f$ = 0.29 (Hexane).

^{1}H-NMR (300 MHz, CDCl$_3$) δ (ppm) 7.49–7.45 (m, 1H), 7.33–7.30 (m, 1H), 7.29–7–7.19 (m, 4H), 7.17–7.13 (m, 1H), 5.88 (bs, 1H), 1.86 (s, 3H), 1.72 (s, 3H), 1.71 (s, 3H).

^{13}C-NMR (75 MHz, CDCl$_3$) δ (ppm) 142.5 (C), 142.1 (C), 136.2 (C), 135.7 (C), 132.4 (CH), 131.2 (C), 130.9 (CH), 130.1 (CH), 130.0 (CH), 127.3 (CH), 127.2 (CH), 127.0 (CH), 123.61 (C), 123.56 (CH), 89.1 (C), 78.8 (C), 26.3 (CH$_3$), 19.5 (CH$_3$), 4.5 (CH$_3$).

HRMS (ESI-TOF) m/z: [M+H]$^+$ calculated for C$_{19}$H$_{18}$Cl 281.1092. Found 281.1104.

4'-Chloro-2-(2-methylprop-1-en-1-yl)-2'-(prop-1-yn-1-yl)-1,1'-biphenyl (1q)

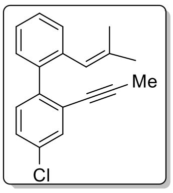

Obtained as colorless oil (118 mg, 0.42 mmol, 65%) from **1q-H** (174 mg, 0.65 mmol) following method B. R$_f$ = 0.29 (Hexane).

^{1}H-NMR (300 MHz, CDCl$_3$) δ (ppm) 7.48–7.46 (m, 1H), 7.36–7.23 (m, 5H), 7.11 (d, *J* = 8.5 Hz, 1H), 5.96 (bs, 1H), 1.84 (s, 3H), 1.77 (s, 3H), 1.74 (m, 3H).

^{13}C-NMR (75 MHz, CDCl$_3$) δ (ppm) 142.4 (C), 139.3 (C), 137.5 (C), 135.4 (C), 132.4 (C), 132.1 (CH), 131.6 (CH), 131.0 (CH), 129.8 (CH), 127.3 (2 x CH), 125.8 (CH), 125.3 (C), 124.3 (CH), 89.9 (C), 78.1 (C), 26.3 (CH$_3$), 19.5 (CH$_3$), 4.5 (CH$_3$).

HRMS (ESI-TOF) m/z: [M+H]$^+$ calculated for C$_{19}$H$_{18}$Cl 281.1092. Found 281.1094.

4-Methoxy-2-(2-methylprop-1-en-1-yl)-2'-(prop-1-yn-1-yl)-1,1'-biphenyl (1r)

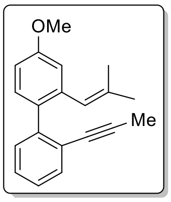

Obtained as colorless oil (34 mg, 0.12 mmol, 87%) from **1r-H** (36 mg, 0.14 mmol) following method B. R$_f$ = 0.18 (Hexane).

^{1}H-NMR (300 MHz, CDCl$_3$) δ (ppm) 7.467–7.44 (m, 1H), 7.27–7.21 (m, 3H), 7.17–7.13 (m, 1H), 6.86–6.81 (m, 2H), 5.96 (bs, 1H), 3.86 (s, 3H), 1.86 (s, 3H), 1.74 (s, 3H), 1.73 (s, 3H).

^{13}C-NMR (75 MHz, CDCl$_3$) δ (ppm) 158.3 (C), 143.5 (C), 138.7 (C), 135.1 (C), 133.2 (C), 132.3 (CH), 131.2 (CH), 130.6 (CH), 126.9 (CH), 126.5 (CH), 124.6 (CH),123.8 (C), 115.0 (CH), 111.1 (CH), 88.3 (C), 79.4 (C), 55.4 (CH$_3$), 26.4 (CH$_3$), 19.7 (CH$_3$), 4.7 (CH$_3$).

HRMS (ESI-TOF) m/z: [M+H]$^+$ calculated for C$_{20}$H$_{21}$O 277.1587. Found 277.1597.

4'-Methoxy-2-(2-methylprop-1-en-1-yl)-2'-(prop-1-yn-1-yl)-1,1'-biphenyl (1s)

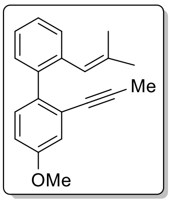

Obtained as colorless oil (136 mg, 0.49 mmol, 80%) from **1s-H** (160 mg, 0.61 mmol) following method B. R_f = 0.30 (Hexane/EtOAc 40:1).

^{1}H-NMR (300 MHz, $CDCl_3$) δ (ppm) 7.34–7.22 (m, 4H), 7.09 (d, *J* = 8.4 Hz, 1H), 7.02 (d, *J* = 2.7 Hz, 1H), 6.85 (dd, *J* = 8.4, 2.7 Hz, 1H), 5.97 (bs, 1H), 3.84 (s, 3H), 1.85 (s, 3H), 1.76 (d, *J* = 1.5 Hz, 3H), 1.75 (d, *J* = 1.4 Hz, 3H).

^{13}C-NMR (75 MHz, $CDCl_3$) δ (ppm) 158.0 (C), 140.1 (C), 137.7 (C), 136.5 (C), 134.6 (C), 131.4 (CH), 130.5 (CH), 129.6 (CH), 126.6 (CH), 125.6 (CH), 124.7 (CH), 124.4 (C), 116.6 (CH), 113.8 (CH), 88.3 (C), 79.3 (C), 55.4 (CH_3), 26.4 (CH_3), 19.7 (CH_3), 4.6 (CH_3).

HRMS (ESI-TOF) m/z: $[M+H]^+$ calculated for $C_{20}H_{21}O$ 277.1587. Found 277.1591.

4',6'-Dimethoxy-2-(2-methylprop-1-en-1-yl)-2'-(prop-1-yn-1-yl)-5'-chloro-1,1'-biphenyl (1t)

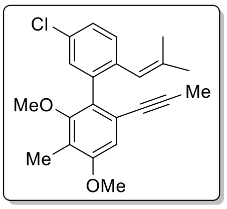

Obtained as colorless oil (52 mg, 0.15 mmol, 65%) from **1o-H** (75.6 mg, 0.23 mmol) following method B. Methylation occurred at both the alkyne terminus and the electron-rich arene. R_f = 0.26 (Hexane/EtOAc 40:1).

^{1}H-NMR (300 MHz, $CDCl_3$) δ (ppm) 7.30–7.20 (m, 3H), 6.75 (s, 1H), 5.91 (bs, 1H), 3.84 (s, 3H), 3.30 (s, 3H), 2.16 (s, 3H), 1.79 (s, 3H), 1.75 (s, 3H), 1.73 (s, 3H).

^{13}C-NMR (75 MHz, $CDCl_3$) δ (ppm) 157.7 (C), 156.5 (C), 138.8 (C), 137.0 (C), 135.6 (C), 131.0 (C), 130.7 (2 x CH), 129.0 (C), 126.8 (CH), 123.7 (CH), 122.3 (C), 120.3 (C), 109.5 (CH), 88.1 (C), 78.8 (C), 60.1 (CH_3), 55.7 (CH_3), 26.5 (CH_3), 19.6 (CH_3), 9.3 (CH_3), 4.4 (CH_3).

HRMS (ESI-TOF) m/z: $[M+H]^+$ calculated for $C_{22}H_{24}ClO_2$ 355.1459. Found 355.1459.

2'-(4-Hydroxybut-1-yn-1-yl)-2-(2-methylprop-1-en-1-yl)-1,1'-biphenyl (1u)

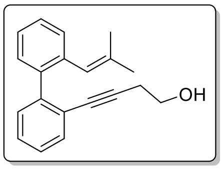

Obtained as colorless oil (74 mg, 0.27 mmol, 68%) from **1a-H** (100 mg, 0.43 mmol, 1.5 equiv.) following the method previously described. R_f = 0.30 (Hexane/EtOAc 9:1).

^{1}H-NMR (400 MHz, $CDCl_3$) δ (ppm) 7.40–7.38 (m, 1H), 7.30–7.24 (m, 2H), 7.22–7.16 (m, 4H), 7.11–7.09 (m, 1H), 5.87 (bs, 1H), 3.38 (q, *J* = 5.9 Hz, 2H), 2.36 (t, *J* = 5.9 Hz, 2H), 1.64 (d, *J* =1.4 Hz, 3H), 1.63 (d, *J* = 1.5 Hz, 3H).

^{13}C-NMR (100 MHz, $CDCl_3$) δ (ppm) 144.1 (C), 140.4 (C), 137.2 (C), 135.1 (C), 131.5 (CH), 129,7 (CH), 129.6 (CH), 129.5 (CH), 127.2 (CH), 127.0 (CH), 126.7 (CH), 125.5 (CH), 123.8 (CH), 122.6 (C), 89.2 (C), 82.0 (C), 60.7 (CH_2), 26.1 (CH_3), 23.8 (CH_2), 19.3 (CH_3).

HRMS (ESI-TOF) m/z: $[M+H]^+$ calculated for $C_{20}H_{21}O$ 277.1587. Found 277.1587.

2'-(3-(3-Methoxyphenyl)prop-1-yn-1-yl)-2-(2-methylprop-1-en-1-yl)-1,1'-biphenyl (1v)

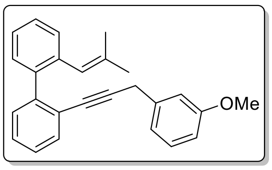

Obtained as yellow oil (73 mg, 0.21 mmol, 22%) from **1a-H** (213 mg, 0.92 mmol, 1.5 equiv.) following the method previously described. R_f = 0.45 (Hexane/EtOAc 40:1).

^{1}H-NMR (400 MHz, $CDCl_3$) δ (ppm) 7.48–7.46 (m, 1H), 7.29–7.12 (m, 7H), 7.06 (t, *J* = 7.8 Hz, 1H), 6.69–6.65 (m, 2H), 6.56 (d, *J* = 8.1, 1H), 5.94 (bs, 1H), 3.67 (s, 3H), 3.55 (s, 2H), 1.64 (s, 3H), 1.62 (s, 3H).

^{13}C-NMR (100 MHz, $CDCl_3$) δ (ppm) 159.7 (C), 144.2 (C), 140.5 (C), 138.3 (C), 137.5 (C), 135.0 (C), 132.3 (CH), 130.3 (CH), 130.2 (CH), 129.7 (CH), 129.5 (CH), 127.3 (CH), 127.0 (CH), 126.8 (CH), 125.8 (CH), 124.5 (CH), 123.3 (C), 120.3 (CH), 113.5 (CH), 112.0 (CH), 90.0 (C), 82.4 (C), 55.2 (CH_3), 26.3 (CH_3), 25.9 (CH_2), 19.5 (CH_3).

HRMS (ESI-TOF) m/z: $[M+H]^+$ calculated for $C_{26}H_{25}O$ 353.1900. Found 353.1910.

General procedure for the synthesis of dibenzocycloheptadienes 2 and 4:

In a sealed tube with bis(trifluoromethane)sulfonimide (10 mol%) under Ar atmosphere, a solution of the corresponding 2-alkenyl-2`-alkynyl-1,1`-biphenyl **1** (1 equiv., 0.15-0.30 mmol) and nucleophile (1.1 equiv., 0.17-0.33 mmol) in the appropriate solvent [$CHCl_3$ (0.05 M) for water and DCE (0.1 M) for carbon nucleophiles] was added. The resulted reaction mixture was stirred at 60 ºC until complete consumption of the starting enyne **1**, as monitored by TLC or GC/MS. Then, solvent was removed under reduced pressure and the crude reaction mixture was purified by flash chromatography on silica gel, using mixtures of hexane with EtOAc, DCM or Et_2O as eluents to give the corresponding dibenzocycloheptadienes **2** or **4**.

Characterization data of dibenzocycloheptadienes 2 and 4:

5-Benzoyl-6,6-dimethyl-6,7-dihydro-5*H*-dibenzo[*a*,*c*]cycloheptadiene (2a)

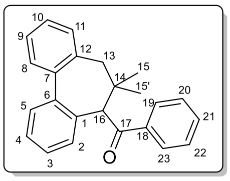

Obtained as white solid (234 mg, 0.72 mmol, 72%) from **1a** (308 mg, 1 mmol). R_f = 0.27 (Hexane/DCM 8:2). M.p.: 81–83 ºC.

^{1}H-NMR (300 MHz, $CDCl_3$) δ (ppm) 7.61 (d, *J* = 7.5 Hz, 1H, H-8), 7.53–7.50 (m, 3H, H-5,23), 7.47 (dt, *J* = 7.5, 1.2 Hz, 1H, H-9), 7.41–7.33 (m, 3H, H-4,10,21), 7.27 (d, *J* = 7.5 Hz, 1H, H-11), 7.25–7.19 (m, 3H, H-3,22), 7.17 (d, *J* = 7.6 Hz, 1H, H-2), 4.24 (s, 1H, H-16), 2.25 (AB system, *J* = 13 Hz, 2H, H-13), 1.39 (s, 3H, H-15), 1.17 (s, 3H, H-15’).

^{13}C-NMR (75 MHz, $CDCl_3$) δ (ppm) 200.1 (C, C-17), 140.8 (C, C-6), 140.3 (C, C-7), 138.3 (C, C-1), 138.0 (C, C-12), 136.1 (C, C-18), 132.5 (CH, C-4), 129.8 (CH, C-11), 128.7 (CH, C-21), 128.4 (4 x CH), 128.0 (CH, C-10), 127.8 (CH, C-8), 127.6 (CH, C-3), 127.5 (CH), 127.4 (CH, C-9), 127.3 (CH), 55.8 (CH, C-16), 48.7 (CH_2, C-13), 46.0 (C, C-14), 25.9 (CH_3, C-15’), 23.9 (CH_3, C-15).

HRMS (ESI-TOF) m/z: $[M+H]^+$ calculated for $C_{24}H_{23}O$ 327.1743. Found 327.1746.

5-(4-Chlorobenzoyl)-6,6-dimethyl-6,7-dihydro-5*H*-dibenzo[*a*,*c*]cycloheptadiene (2c)

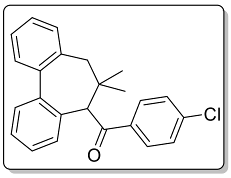

Obtained as white solid (76 mg, 0.21 mmol, 72%) from **1c** (100 mg, 0.29 mmol). R_f = 0.22 (Hexane/DCM 8:2). M.p.: 109–111 ºC.

^{1}H-NMR (300 MHz, $CDCl_3$) δ (ppm) 7.61 (dd, *J* = 7.5, 1.5 Hz, 1H), 7.53 (dd, *J* = 7.7, 1.2 Hz, 1H), 7.49 (dd, *J* = 7.5, 1.5 Hz, 1H), 7.43 (d, *J* = 8.7 Hz, 2H), 7.41–7.34 (m, 2H), 7.29–7.23 (m, 1H), 7.22–7.16 (m, 3H), 7.10 (dd, *J* = 7.7, 1.2 Hz, 1H), 4.15 (s, 1H), 2.25 (AB system, *J* = 13.4 Hz, 2H), 1.38 (s, 3H), 1.17 (s, 3H).

^{13}C-NMR (75 MHz, $CDCl_3$) δ (ppm) 198.8 (C=O), 140.8 (C), 140.2 (C), 138.8 (C), 138.0 (C), 136.5 (C), 135.8 (C), 129.9 (3 x CH), 128.8 (CH), 128.7 (2 x CH), 128.1 (CH), 127.7 (CH), 127.61 (2 x CH), 127.58 (CH), 127.4 (CH), 56.1 (CH), 48.7 (CH_2), 46.0 (C), 25.8 (CH_3), 23.8 (CH_3).

HRMS (ESI-TOF) m/z: $[M+H]^+$ calculated for $C_{24}H_{22}ClO$ 361.1354. Found 361.1348.

5-(2-Chlorobenzoyl)-6,6-dimethyl-6,7-dihydro-5*H*-dibenzo[*a*,*c*]cycloheptadiene (2d)

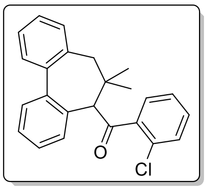

Obtained as colorless oil (72 mg, 0.20 mmol, 84%) from **1d** (84 mg, 0.24 mmol). R_f = 0.21 (Hexane/DCM 8:2).

^{1}H-NMR (300 MHz, $CDCl_3$) δ (ppm) 7.49–7.41 (m, 3H), 7.40–7.28 (m, 4H), 7.23–7.16 (m, 3H), 7.12–7.04 (m, 2H), 4.19 (s, 1H), 2.25 (d, *J* = 13.2 Hz, 1H), 2.17 (d, *J* = 13.2 Hz, 1H), 1.32 (s, 3H), 1.19 (s, 3H).

^{13}C-NMR (75 MHz, $CDCl_3$) δ (ppm) 202.5 (C=O), 141.2 (C), 140.6 (C), 140.4 (C), 137.7 (C), 135.0 (C), 131.5 (CH), 130.9 (CH), 129.6 (CH), 129.3 (CH), 128.7 (CH), 127.9 (CH), 127.8 (CH), 127.4 (2 x CH), 127.3 (CH), 127.2 (CH), 126.7 (CH), 59.1 (CH), 48.4 (CH_2), 47.0 (C), 26.1 (CH_3), 23.8 (CH_3). One quaternary carbon is not observed.

HRMS (ESI-TOF) m/z: $[M+H]^+$ calculated for $C_{24}H_{22}ClO$ 361.1354. Found 361.1363.

5-(2-Bromobenzoyl)-6,6-dimethyl-6,7-dihydro-5*H*-dibenzo[*a*,*c*]cycloheptadiene (2e)

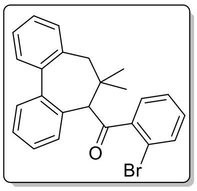

Obtained as colorless oil (50 mg, 0.12 mmol, 95%) from **1e** (50 mg, 0.13 mmol). R_f = 0.20 (Hexane/DCM 8:2).

^{1}H-NMR (300 MHz, $CDCl_3$) δ (ppm) 7.54–7.43 (m, 4H), 7.41–7.29 (m, 4H), 7.22 (dd, *J* = 7.3, 1.5 Hz, 1H), 7.14–7.02 (m, 3H), 4.19 (s, 1H), 2.25 (d, *J* = 13.1 Hz, 1H), 2.17 (d, *J* = 13.1 Hz, 1H), 1.32 (s, 3H), 1.19 (s, 3H).

^{13}C-NMR (75 MHz, $CDCl_3$) δ (ppm) 202.7 (C=O), 142.2 (C), 141.0 (C), 140.2 (C), 137.6 (C), 135.0 (C), 134.2 (CH), 131.5 (CH), 129.5 (CH), 129.3 (CH), 128.6 (CH), 127.8 (CH), 127.7 (CH), 127.4 (2 x CH), 127.3 (CH), 127.2 (2 x CH), 119.6 (C), 58.8 (CH), 48.5 (CH_2), 47.1 (C), 26.3 (CH_3), 23.8 (CH_3).

HRMS (ESI-TOF) m/z: $[M+H]^+$ calculated for $C_{24}H_{22}BrO$ 405.0849. Found 405.0849.

5-(3-Methoxybenzoyl)-6,6-dimethyl-6,7-dihydro-5*H*-dibenzo[*a*,*c*]cycloheptadiene (2f)

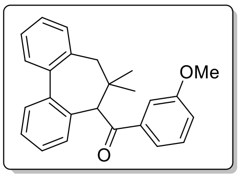

Obtained as white solid (30 mg, 0.08 mmol, 56%) from **1f** (50 mg, 0.15 mmol). R_f = 0.13 (Hexane/DCM 8:2). M.p.: 76–78 ºC.

^{1}H-NMR (300 MHz, $CDCl_3$) δ (ppm) 7.58 (dd, *J* = 7.4, 1.5 Hz, 1H), 7.52 (dd, *J* = 7.6, 1.4 Hz, 1H), 7.49–7.43 (m, 1H), 7.42–7.33 (m, 2H), 7.31–7.24 (m, 1H), 7.24–7.19 (m, 1H), 7.17–7.11 (m, 2H), 7.09 (dd, *J* = 7.9, 0.9 Hz, 1H), 7.01 (dd, *J* = 7.7, 1.4 Hz, 1H), 6.94–6.88 (m, 1H), 4.21 (s, 1H), 3.69 (s, 3H), 2.25 (AB system, *J* = 13.2 Hz, 2H), 1.38 (s, 3H), 1.17 (s, 3H).

^{13}C-NMR (75 MHz, $CDCl_3$) δ (ppm) 199.8 (C=O), 159.5 (C), 140.8 (C), 140.3 (C), 139.5 (C), 138.1 (C), 136.2 (C), 129.8 (CH), 129.4 (CH), 128.6 (CH), 128.0 (CH), 127.7 (CH), 127.6 (2 x CH), 127.5 (CH), 127.3 (CH), 121.1 (CH), 119.5 (CH), 112.4 (CH), 56.1 (CH), 55.2 (CH_3) 48.8 (CH_2), 46.0 (C), 25.8 (CH_3), 23.8 (CH_3).

HRMS (ESI-TOF) m/z: $[M+H]^+$ calculated for $C_{25}H_{25}O_2$ 357.1849. Found 357.1846.

5-(4-Methylbenzoyl)-6,6-dimethyl-6,7-dihydro-5*H*-dibenzo[*a*,*c*]cycloheptadiene (2g)

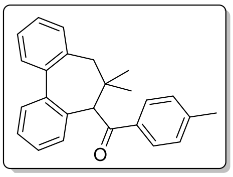

Obtained as white solid (18 mg, 0.05 mmol, 33%) from **1g** (50 mg, 0.16 mmol). R_f = 0.19 (Hexane/DCM 8:2)º. M.p.: 76–78 ºC

^{1}H-NMR (300 MHz, $CDCl_3$) δ (ppm) 7.59 (d, *J* = 7.4 Hz, 1H), 7.53–7.45 (m, 2H), 7.44–7.37 (m, 3H), 7.34 (d, *J* = 7.2 Hz, 1H), 7.29–7.22 (m, 1H), 7.22–7.13 (m, 2H), 7.02 (d, *J* = 8.3 Hz, 2H), 4.20 (s, 1H), 2.27 (s, 3H), 2.23 (AB system, *J* = 13.2 Hz, 2H), 1.37 (s, 3H), 1.15 (s, 3H).

^{13}C-NMR (75 MHz, $CDCl_3$) δ (ppm) 199.8 (C=O), 143.2 (C), 140.8 (C), 140.4 (C), 138.1 (C), 136.3 (C), 135.8 (C), 129.8 (CH), 129.1 (2 x CH), 128.6 (CH), 128.5 (2 x CH), 127.9 (CH), 127.7 (CH), 127.6 (CH), 127.5 (CH), 127.4 (CH), 127.3 (CH), 55.7 (CH), 48.8 (CH_2), 46.0 (C), 25.9 (CH_3), 23.9 (CH_3), 21.6 (CH_3).

HRMS (ESI-TOF) m/z: $[M+H]^+$ calculated for $C_{25}H_{25}O$ 341.1900. Found 341.1895.

5-(Naphthalen-1-oyl)-6,6-dimethyl-6,7-dihydro-5*H*-dibenzo[*a*,*c*]cycloheptadiene (2h)

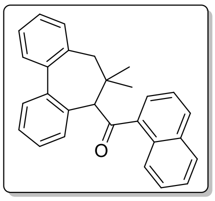

Obtained as white solid (30 mg, 0.08 mmol, 57%) from **1h** (50 mg, 0.14 mmol). R_f = 0.16 (Hexane/DCM 8:2). M.p.: 132–134 ºC.

^{1}H-NMR (300 MHz, $CDCl_3$) δ (ppm) 8.53 (d, *J* = 8.5 Hz, 1H), 7.79–7.74 (m, 2H), 7.57–7.30 (m, 8H), 7.27–7.15 (m, 4H), 4.34 (s, 1H), 2.31 (d, *J* = 13.1 Hz, 1H), 2.21 (d, *J* = 13.1 Hz, 1H), 1.43 (s, 3H), 1.19 (s, 3H).

^{13}C-NMR (75 MHz, $CDCl_3$) δ (ppm) 204.6 (C=O), 140.9 (C), 140.4 (C), 138.3 (C), 137.9 (C), 135.9 (C), 134.0 (C), 132.2 (CH), 130.3 (C), 129.7 (CH), 128.8 (CH), 128.6 (CH), 127.9 (2 x CH), 127.8 (2 x CH), 127.5 (CH), 127.4 (CH), 127.32 (CH), 127. 29 (CH), 127.2 (CH), 126.4 (CH), 125.7 (CH), 58.6 (CH), 48.6 (CH_2), 47.2 (C), 26.3 (CH_3), 23.9 (CH_3).

HRMS (ESI-TOF) m/z: $[M+H]^+$ calculated for $C_{28}H_{25}O$ 377.1900. Found 377.1897.

5-(Naphthalen-2-oyl)-6,6-dimethyl-6,7-dihydro-5*H*-dibenzo[*a*,*c*]cycloheptadiene (2i)

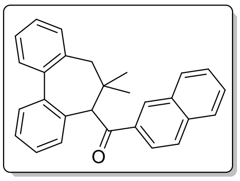

Obtained as colorless oil (56 mg, 0.15 mmol, 65%) from **1i** (81 mg, 0.23 mmol). R_f = 0.16 (Hexane/DCM 8:2).

^{1}H-NMR (400 MHz, $CDCl_3$) δ (ppm) 7.86 (s, 1H), 7.65 (d, *J* = 8.2 Hz, 1H), 7.61–7.55 (m, 4H), 7.47–7.38 (m, 3H), 7.36–7.30 (m, 2H), 7.28–7.17 (m, 2H), 7.13–7.10 (m, 2H), 4.30 (s, 1H), 2.22 (d, *J* = 13.1 Hz, 1H), 2.16 (d, *J* = 13.1 Hz, 1H), 1.34 (s, 3H), 1.12 (s, 3H).

^{13}C-NMR (100 MHz, $CDCl_3$) δ (ppm) 200.0 (C=O), 140.9 (C), 140.5 (C), 138.1 (C), 136.3 (C), 135.5 (C), 135.2 (C), 132.4 (C), 130.2 (CH), 129.9 (CH), 129.7 (CH), 128.7 (CH), 128.3 (CH), 128.2 (CH), 128.0 (CH), 127.69 (2 x CH), 127.65 (CH), 127.60 (CH), 127.5 (CH), 127.4 (CH), 126.6 (CH), 124.3 (CH), 56.0 (CH), 48.8 (CH_2), 46.1 (C), 25.9 (CH_3), 23.9 (CH_3).

HRMS (ESI-TOF) m/z: $[M+H]^+$ calculated for $C_{28}H_{25}O$ 377.1900 Found $[M+H]^+$: 377.1903.

7-Benzoyl-2-chloro-6,6-dimethyl-6,7-dihydro-5*H*-dibenzo[*a*,*c*]cycloheptadiene (2j)

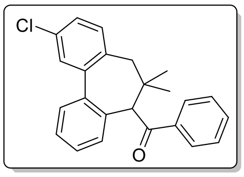

Obtained as white solid (72 mg, 0.20 mmol, 69%) from **1j** (100 mg, 0.29 mmol). R_f = 0.18 (Hexane/DCM 8:2). M.p.: 129–131 ºC.

^{1}H-NMR (300 MHz, $CDCl_3$) δ (ppm) 7.59 (d, *J* = 2.2 Hz, 1H), 7.52–7.47 (m, 3H), 7.41–7.31 (m, 3H), 7.28–7.22 (m, 3H), 7.22–7.13 (m, 2H), 4.18 (s, 1H), 2.20 (bs, 2H), 1.36 (s, 3H), 1.14 (s, 3H).

^{13}C-NMR (75 MHz, $CDCl_3$) δ (ppm) 199.8 (C=O), 142.0 (C), 139.6 (C), 138.2 (C), 136.5 (C), 136.1 (C), 132.9 (C), 132.6 (CH), 131.1 (CH), 128.7 (CH), 128.5 (2 x CH), 128.4 (2 x CH), 128.1 (CH), 127.9 (CH), 127.8 (CH), 127.7 (CH), 127.6 (CH), 55.7 (CH), 48.1 (CH_2), 46.1 (C), 25.8 (CH_3), 23.9 (CH_3).

HRMS (ESI-TOF) m/z: $[M+H]^+$ calculated for $C_{24}H_{22}OCl$ 361.1354. Found 361.1347.

5-Benzoyl-2-chloro-6,6-dimethyl-6,7-dihydro-5*H*-dibenzo[*a*,*c*]cycloheptadiene (2k)

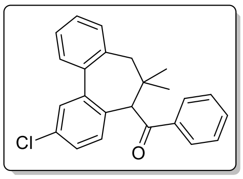

Obtained as white solid (34 mg, 0.09 mmol, 64%) from **1k** (50 mg, 0.14 mmol). R_f = 0.20 (Hexane/DCM 8:2). M.p.: 72–74 ºC.

^{1}H-NMR (300 MHz, $CDCl_3$) δ (ppm) 7.57 (dd, *J* = 7.4, 1.6 Hz, 1H), 7.50–7.46 (m, 3H), 7.43 (dd, *J* = 6.4, 1.6 Hz, 1H), 7.40–7.35 (m, 2H), 7.29–7.14 (m, 4H), 7.11 (d, *J* = 8.2 Hz, 1H), 4.19 (s, 1H), 2.22 (bs, 2H), 1.37 (s, 3H), 1.16 (s, 3H).

^{13}C-NMR (75 MHz, $CDCl_3$) δ (ppm) 199.6 (C=O), 142.5 (C), 139.0 (C), 138.0 (C), 137.9 (C), 134.5 (C), 133.1 (C), 132.6 (CH), 129.9 (CH), 128.8 (CH), 128.6 (CH), 128.4 (3 x CH), 128.3 (2 x CH), 127.6 (CH), 127.4 (2 x CH), 55.2 (CH), 48.6 (CH_2), 46.2 (C), 25.9 (CH_3), 23.9 (CH_3).

HRMS (ESI-TOF) m/z: $[M+H]^+$ calculated for $C_{24}H_{22}OCl$ 361.1354. Found 361.1362.

5-Benzoyl-11-fluoro-6,6-dimethyl-6,7-dihydro-5*H*-dibenzo[*a*,*c*]cycloheptadiene (2l)

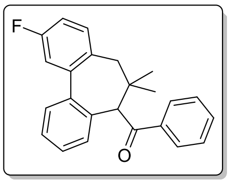

Obtained as white solid (103.9 mg, 0.30 mmol, 97%) from **1l** (100.0 mg, 0.31 mmol). R_f = 0.34 (Hexane/DCM 8:2). M.p.: 102–103 ºC.

^{1}H-NMR (300 MHz, $CDCl_3$) δ (ppm) 7.56–7.52 (m, 2H), 7.49 (dd, *J* = 7.5, 1.4 Hz, 1H), 7.42–7.31 (m, 3H), 7.29–7.18 (m, 5H), 7.08 (td, *J* = 8.5, 2.7 Hz, 1H), 4.22 (s, 1H), 2.21 (s, 2H), 1.38 (s, 3H), 1.16 (s, 3H).

^{13}C-NMR (75 MHz, $CDCl_3$) δ (ppm) 199.9 (C), 162.3 (d, $J_{C\text{-}F}$ = 244.9 Hz, C), 142.1 (d, $J_{C\text{-}F}$ = 7.6 Hz, C), 139.9 (d, $J_{C\text{-}F}$ = 2.4 Hz, C), 138.2 (C), 136.0 (C), 133.7 (d, $J_{C\text{-}F}$ = 3.2 Hz, C), 132.6 (CH), 131.1 (d, $J_{C\text{-}F}$ = 8.3 Hz, CH), 128.6 (CH), 128.5 (2 x CH), 128.4 (2 x CH), 128.0 (CH), 127.8 (CH), 127.6 (CH), 114.6 (d, $J_{C\text{-}F}$ = 21.5 Hz, CH), 114.4 (d, $J_{C\text{-}F}$ = 20.9 Hz, CH), 55.7 (CH), 47.9 (CH_2), 46.0 (C), 25.7 (CH_3), 23.8 (CH_3). **^{19}F-NMR (283 MHz, $CDCl_3$)** δ (ppm) –115.9 (m).

HRMS (ESI-TOF) m/z: $[M+H]^+$ calculated for $C_{24}H_{22}FO$ 345.1649. Found 345.1638.

7-Benzoyl-3-methoxy-6,6-dimethyl-6,7-dihydro-5*H*-dibenzo[*a*,*c*]cycloheptadiene (2m)

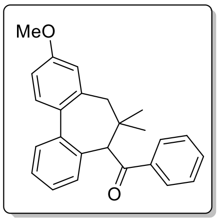

Obtained as yellow oil (32 mg, 0.09 mmol, 60%) from **1m** (50 mg, 0.15 mmol). R_f = 0.24 (Hexane/DCM 7:3).

^{1}H-NMR (300 MHz, CDCl$_3$) δ (ppm) 7.57–7.44 (m, 4H), 7.38–7.30 (m, 2H), 7.27–7.10 (m, 4H), 6.99 (dd, *J* = 8.4, 2.6 Hz, 1H), 6.83 (d, *J* = 2.7 Hz, 1H), 4.24 (s, 1H), 3.89 (s, 3H), 2.26 (d, *J* = 13.1 Hz, 1H), 2.16 (d, *J* = 13.1 Hz, 1H), 1.37 (s, 3H), 1.17 (s, 3H).

^{13}C-NMR (75 MHz, CDCl$_3$) δ (ppm) 200.2 (C=O), 159.4 (C), 140.6 (C), 139.5 (C), 138.3 (C), 135.9 (C), 132.9 (C), 132.5 (CH), 128.8 (CH), 128.5 (CH), 128.4 (4 x CH), 127.6 (CH), 127.3 (CH), 127.0 (CH), 115.6 (CH), 112.2 (CH), 55.9 (CH$_3$), 55.5 (CH), 49.1 (CH$_2$), 45.8 (C), 26.1 (CH$_3$), 23.9 (CH$_3$).

HRMS (ESI-TOF) m/z: [M+H]$^+$ calculated for $C_{25}H_{25}O_2$ 357.1849. Found 357.1847.

5-Benzoyl-1,3-dimethoxy-6,6-dimethyl-6,7-dihydro-5*H*-dibenzo[*a*,*c*]cycloheptadiene (2n)

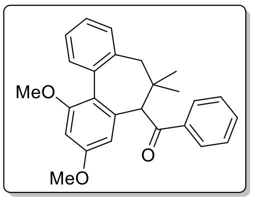

Obtained as white solid (32 mg, 0.08 mmol, 75%) from **1n** (42 mg, 0.11 mmol). R_f = 0.36 (Hexane/DCM 1:1). M.p.: 142–144 ºC.

^{1}H-NMR (300 MHz, CDCl$_3$) δ (ppm) 7.72 (dd, *J* = 7.6, 1.2 Hz, 1H), 7.55–7.51 (m, 2H), 7.43–7.30 (m, 3H), 7.27–7.21 (m, 3H), 6.50 (bs, 1H), 6.32 (bs, 1H), 4.06 (s, 1H), 3.79 (s, 3H), 3.71 (s, 3H), 2.28 (d, *J* = 12.7 Hz, 1H), 2.16 (d, *J* = 12.7 Hz, 1H),1.36 (s, 3H), 1.10 (s, 3H).

^{13}C-NMR (75 MHz, CDCl$_3$) δ (ppm) 200.2 (C=O), 159.6 (C), 157.4 (C), 138.3 (C), 138.4 (C), 138.3 (C), 135.5 (C), 132.2 (CH), 130.4 (CH), 129.7 (CH), 128.4 (2 x CH), 128.3 (2 x CH), 127.2 (CH), 126.1 (CH), 120.8 (C), 104.5 (CH), 97.7 (CH), 56.0 (CH), 55.7 (CH$_3$), 55.5 (CH$_3$), 48.7 (CH$_2$), 45.5 (C), 25.7 (CH$_3$), 24.2 (CH$_3$).

HRMS (ESI-TOF) m/z: [M+H]$^+$ calculated for $C_{26}H_{27}O_3$ 387.1955. Found 387.1949.

5-Benzoyl-11-chloro-1,3-dimethoxy-6,6-dimethyl-6,7-dihydro-5*H*-dibenzo[*a*,*c*]cycloheptadiene (2o)

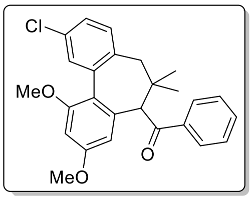

Obtained as white solid (66.2 mg, 0.15 mmol, 87%) from **1o** (74.5 mg, 0.18 mmol). R_f = 0.34 (Hexane/DCM 1:1). M.p.: 106–107 ºC.

^{1}H-NMR (500 MHz, CDCl$_3$) δ (ppm) 7.72 (d, *J* = 2.3 Hz, 1H), 7.56–7.52 (m, 2H), 7.42–7.37 (m, 1H), 7.30–7.25 (m, 3H), 7.17 (d, *J* = 8.0 Hz, 1H), 6.49 (d, *J* = 2.3 Hz, 1H), 6.32 (d, *J* = 2.3 Hz, 1H), 4.02 (s, 1H), 3.82 (s, 3H), 3.72 (s, 3H), 2.23 (d, *J* = 13.0 Hz, 1H), 2.15 (d, *J* = 13.0 Hz, 1H), 1.36 (s, 3H), 1.09 (s, 3H).

^{13}C-NMR (125 MHz, CDCl$_3$) δ (ppm) 199.9 (C), 160.1 (C), 157.4 (C), 138.8 (C), 138.3 (C), 137.3 (C), 136.9 (C), 132.6 (CH), 131.8 (C), 130.8 (CH), 130.3 (CH), 128.5 (2 x CH), 128.3 (2 x CH), 127.2 (CH), 119.5 (C), 104.7 (CH), 97.7 (CH), 55.9 (CH), 55.8 (CH$_3$), 55.5 (CH$_3$), 48.1 (CH$_2$), 45.5 (C), 25.6 (CH$_3$), 24.2 (CH$_3$).

HRMS (ESI-TOF) m/z: [M+H]$^+$ calculated for $C_{26}H_{25}ClO_3$ 421.1565. Found 421.1574.

(*Z*)-5-((3,4-Dimethoxyphenyl)(phenyl)methylene)-6,6-dimethyl-6,7-dihydro-5*H*-dibenzo[*a*,*c*]cycloheptadiene (4aa)

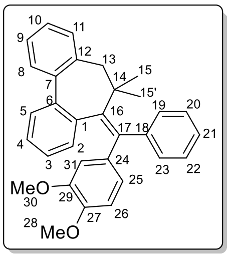

Obtained as white solid (376 mg, 0.84 mmol, 84%) from **1a** (308 mg, 1 mmol) and 1,2-dimethoxybenzene (152 mg, 1.1 mmol, 1.1 equiv.). R_f = 0.10 (Hexane/EtOAc 40:1). M.p.: 45–47 ºC.

^{1}H-NMR (500 MHz, CDCl$_3$) δ (ppm) 7.54 (dd, *J* = 7.5, 1.3 Hz, 1H, H-8), 7.50 (dt, *J* = 7.6, 1.6 Hz, 1H, H-19), 7.43 (td, *J* = 7.5, 1.3 Hz, 1H, H-9), 7.36 (td, *J* = 7.6, 1.6 Hz, 1H, H-20), 7.33–7.30 (m, 2H, H-5,10), 7.22–7.18 (m, 3H, H-2,11,21), 7.11 (td, *J* = 7.5, 1.4 Hz, 1H, H-22), 7.01–6.94 (m, 2H,

H-3,4), 6.70 (dt, J = 7.8, 1.6 Hz, 1H, H-23), 6.43 (d, J = 8.3 Hz, 1H, H-26), 6.19 (dd, J = 8.3, 2.0 Hz, 1H, H-25), 6.05 (d, J = 2.0 Hz, 1H, H-31), 3.69 (s, 3H, H-28), 3.60 (s, 3H, H-30), 2.75 (d, J = 12.7 Hz, 1H, H-13), 2.12 (d, J = 12.7 Hz, 1H, H-13), 1.39 (s, 3H, H-15'), 0.73 (s, 3H, H-15).

^{13}C-NMR (75 MHz, CDCl$_3$) δ (ppm) 147.3 (C, C-29), 146.4 (C, C-27), 145.9 (C, C-16), 144.3 (C, C-18), 142.4 (C, C-1), 141.7 (C, C-17), 141.4 (C,C-6), 140.9 (C, C-7), 138.5 (C, C-12), 137.9 (C, C-24), 132.5 (CH, C-4), 130.0 (CH, C-23), 129.5 (CH, C-11), 128.8 (CH, C-19), 128.1 (CH, C-22), 127.3 (CH, C-20), 127.1 (CH, C-3), 127.01 (4 x CH, C-2,5,9,10), 126.6 (CH, C-8), 126.4 (CH, C-21), 122.1 (CH, C-25), 112.9 (CH, C-31), 109.9 (CH, C-26), 55.7 (CH$_3$, C-30), 55.6 (CH$_3$, C-28), 50.2 (CH$_2$, C-13), 48.5 (C, C-14), 30.3 (CH$_3$, C-15), 27.2 (CH$_3$, C-15').

HRMS (ESI-TOF) m/z: [M+H]$^+$ calculated for $C_{32}H_{31}O_2$ 447.2319. Found 447.2311.

(*Z*)-5-((3,4-Dimethoxyphenyl)(4-chlorophenyl)methylene)-6,6-dimethyl-6,7-dihydro-5*H*-dibenzo[*a*,*c*]cycloheptadiene (4ca)

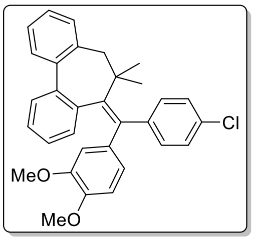

Obtained as white solid (43 mg, 0.09 mmol, 64%) from **1c** (50 mg, 0.14 mmol) and 1,2-dimethoxybenzene (22 mg, 0.15 mmol, 1.1 equiv.). R_f = 0.24 (Hexane/EtOAc 9:1). M.p.: 182–184 ºC.

^{1}H-NMR (300 MHz, CDCl$_3$) δ (ppm) 7.53 (dd, J = 7.6, 1.4 Hz, 1H), 7.47–7.40 (m, 2H), 7.37–7.29 (m, 3H), 7.26–7.17 (m, 2H), 7.09 (dd, J = 8.3, 2.3 Hz, 1H), 7.02–6.90 (m, 2H), 6.64 (dd, J = 8.3, 2.1 Hz, 1H), 6.44 (d, J = 8.3 Hz, 1H), 6.14 (dd, J = 8.3, 2.0 Hz, 1H), 6.02 (d, J = 2.0 Hz, 1H), 3.70 (s, 3H), 3.60 (s, 3H), 2.75 (d, J = 12.9 Hz, 1H), 2.12 (d, J = 12.9 Hz, 1H), 1.38 (s, 3H), 0.76 (s, 3H).

^{13}C-NMR (75 MHz, CDCl$_3$) δ (ppm) 147.6 (C), 146.9 (C), 146.7 (C), 143.0 (C), 142.3 (C), 141.5 (C), 141.0 (C), 140.6 (C), 138.5 (C), 137.6 (C), 132.53 (CH), 132.45 (C), 131.6 (CH), 130.2 (CH), 129.6 (CH), 128.7 (CH), 127.6 (CH), 127.32 (CH), 127.29 (CH), 127.23 (3 x CH), 126.7 (CH), 122.2 (CH), 112.8 (CH), 110.0 (CH), 55.7 (CH$_3$), 55.6 (CH$_3$), 50.1 (CH$_2$), 48.5 (C), 30.3 (CH$_3$), 27.4 (CH$_3$).

HRMS (ESI-TOF) m/z: [M+H]$^+$ calculated for $C_{32}H_{30}O_2Cl$ 481.1929. Found 481.1928.

(*Z*)-5-((3,4-Dimethoxyphenyl)(phenyl)methylene)-10-chloro-6,6-dimethyl-6,7-dihydro-5*H*-dibenzo[*a*,*c*]cycloheptadiene (4ja)

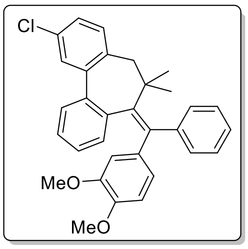

Obtained as white solid (50 mg, 0.10 mmol, 74%) from **1j** (50 mg, 0.14 mmol) and 1,2-dimethoxybenzene (22 mg, 0.15 mmol, 1.1 equiv.). R_f = 0.14 (Hexane/EtOAc 10:1). M.p.: 140–142 ºC.

^{1}H-NMR (300 MHz, CDCl$_3$) δ (ppm) 7.54–7.45 (m, 2H), 7.39–7.36 (m, 1H), 7.31–7.26 (m, 2H), 7.22–7.10 (m, 3H), 7.04–6.92 (m, 2H), 6.73 (d, J = 7.6 Hz, 1H), 6.43 (d, J = 8.3 Hz, 1H), 6.14 (dd, J = 8.3, 2.0 Hz, 1H), 6.02 (d, J = 2.1 Hz, 1H), 3.69 (s, 3H), 3.61 (s, 3H), 2.68 (d, J = 12.9 Hz, 1H), 2.09 (d, J = 12.9 Hz, 1H), 1.38 (s, 3H), 0.71 (s, 3H).

^{13}C-NMR (75 MHz, CDCl$_3$) δ (ppm) 147.6 (C), 146.6 (C), 145.5 (C), 144.3 (C), 142.8 (C), 142.6 (C), 142.4 (C), 140.3 (C), 137.9 (C), 137.2 (C), 132.8 (CH), 132.7 (C), 130.9 (CH), 130.1 (CH), 128.9 (CH), 128.4 (CH), 127.7 (CH), 127.5 (CH), 127.3 (CH), 127.1 (CH), 127.0 (CH), 126.7 (CH), 126.6 (CH), 122.1 (CH), 112.7 (CH), 110.0 (CH), 55.7 (CH$_3$), 55.6 (CH$_3$), 49.5 (CH$_2$), 48.4 (C), 30.2 (CH$_3$), 27.0 (CH$_3$).

HRMS (ESI-TOF) m/z: [M+H]$^+$ calculated for $C_{32}H_{30}O_2Cl$ 481.1929. Found 481.1924.

(*Z*)-5-((3,4-Dimethoxyphenyl)(phenyl)methylene)-2-chloro-6,6-dimethyl-6,7-dihydro-5*H*-dibenzo[*a*,*c*]cycloheptadiene (4ka)

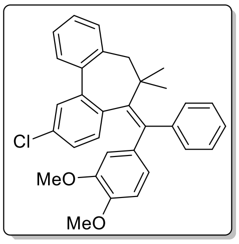

Obtained as white solid (56 mg, 0.11 mmol, 78%) from **1k** (50 mg, 0.14 mmol) and 1,2-dimethoxybenzene (22 mg, 0.15 mmol, 1.1 equiv.). R_f = 0.20 (Hexane/EtOAc 40:1). M.p.: 99–101 ºC.

^{1}H-NMR (300 MHz, CDCl$_3$) δ (ppm) 7.55–7.42 (m, 3H), 7.41–7.31 (m, 3H), 7.23–7.17 (m, 2H), 7.11 (td, *J* = 7.5, 1.6 Hz, 1H), 6.97 (dd, *J* = 8.5, 2.6 Hz, 1H), 6.91 (d, *J* = 6.9 Hz, 1H), 6.71–6.68 (m, 1H), 6.46 (d, *J* = 8.4 Hz, 1H), 6.18 (dd, *J* = 8.4, 2.0 Hz, 1H), 6.06 (d, *J* = 2.0 Hz, 1H), 3.72 (s, 3H), 3.63 (s, 3H), 2.72 (d, *J* = 12.8 Hz, 1H), 2.13 (d, *J* = 12.8 Hz, 1H), 1.38 (s, 3H), 0.73 (s, 3H).

^{13}C-NMR (75 MHz, CDCl$_3$) δ (ppm) 147.6 (C), 146.7 (C), 145.0 (C), 144.1 (C), 143.2 (C), 142.4 (C), 141.1 (C), 139.9 (C), 138.6 (C), 137.7 (C), 133.9 (CH), 132.8 (C), 130.0 (CH), 129.7 (CH), 128.8 (CH), 128.3 (CH), 127.8 (CH), 127.5 (CH), 127.3 (CH), 127.13 (CH), 127.07 (CH), 126.7 (CH), 126.6 (CH), 122.1 (CH), 112.7 (CH), 110.1 (CH), 55.8 (CH$_3$), 55.6 (CH$_3$), 50.0 (CH$_2$), 48.4 (C), 30.2 (CH$_3$), 27.1 (CH$_3$).

HRMS (ESI-TOF) m/z: $[M+H]^+$ calculated for $C_{32}H_{30}O_2Cl$ 481.1929. Found 481.1924.

(*Z*)-5-((3,4-Dimethoxyphenyl)(phenyl)methylene)-9-methoxy-6,6-dimethyl-6,7-dihydro-5*H*-dibenzo[*a*,*c*]cycloheptadiene (4ma)

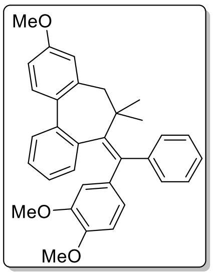

Obtained as white solid (53 mg, 0.11 mmol, 74%) from **1m** (50 mg, 0.15 mmol) and 1,2-dimethoxybenzene (23 mg, 0.17 mmol, 1.1 equiv.). R_f = 0.20 (Hexane/EtOAc 9:1). M.p.: 160–162 ºC.

^{1}H-NMR (300 MHz, CDCl$_3$) δ (ppm) 7.54–7.42 (m, 2H), 7.35 (t, *J* = 7.5 Hz, 1H), 7.30–7.08 (m, 4H), 7.00–6.92 (m, 3H), 6.77 (d, *J* = 3.0 Hz, 1H), 6.73 (d, *J* = 7.4 Hz, 1H), 6.43 (d, *J* = 8.4 Hz, 1H), 6.18 (dd, *J* = 8.4, 2.0 Hz, 1H), 6.05 (d, *J* = 2.0 Hz, 1H), 3.87 (s, 3H), 3.69 (s, 3H), 3.60 (s, 3H), 2.75 (d, *J* = 12.3 Hz, 1H), 2.08 (d, *J* = 12.3 Hz, 1H), 1.39 (s, 3H), 0.76 (s, 3H).

^{13}C-NMR (75 MHz, CDCl$_3$) δ (ppm) 158.8 (C), 147.5 (C), 146.5 (C), 146.2 (C), 144.5 (C), 142.4 (C), 141.7 (C), 141.3 (C), 140.1 (C), 138.2 (C), 133.9 (C), 132.7 (CH), 130.3 (CH), 129.0 (CH), 128.3 (CH), 127.7 (CH), 127.4 (CH), 127.2 (CH), 126.9 (CH), 126.7 (CH), 126.5 (CH), 122.2 (CH), 115.3 (CH), 112.9 (CH), 112.2 (CH), 109.9 (CH), 55.7 (CH$_3$), 55.6 (CH$_3$), 55.4 (CH$_3$), 50.5 (CH$_2$), 48.3 (C), 30.2 (CH$_3$), 27.3 (CH$_3$).

HRMS (ESI-TOF) m/z: $[M+H]^+$ calculated for $C_{33}H_{33}O_3$ 477.2424. Found 477.2420.

(*Z*)-5-((3,4-Dimethoxyphenyl)(phenyl)methylene)-1,3-dimethoxy-6,6-dimethyl-6,7-dihydro-5*H*-dibenzo[*a*,*c*]cycloheptadiene (4na)

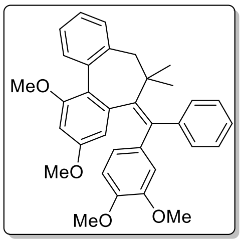

Obtained as white solid (46 mg, 0.10 mmol, 89%) from **1n** (34.7 mg, 0.10 mmol) and 1,2-dimethoxybenzene (15 mg, 0.11 mmol, 1.1 equiv.). R_f = 0.23 (Hexane/EtOAc 9:1). M.p.: 138–140 ºC.

^{1}H-NMR (300 MHz, CDCl$_3$) δ (ppm) 7.69 (d, *J* = 7.6 Hz, 1H), 7.48 (d, *J* = 7.6 Hz, 1H), 7.41–7.32 (m, 2H), 7.29–7.16 (m, 3H), 7.11 (td, *J* = 7.2, 1.3 Hz, 1H), 6.72–6.63 (m, 1H), 6.49 (d, *J* = 8.1 Hz, 1H), 6.37 (d, *J* = 2.5 Hz, 1H), 6.32–6.21 (m, 2H), 6.16 (d, *J* = 2.5 Hz, 1H), 3.75 (s, 3H), 3.73 (s, 3H), 3.69 (s, 3H), 3.62 (s, 3H), 2.77 (d, *J* = 12.5 Hz, 1H), 2.09 (d, *J* = 12.5 Hz, 1H), 1.37 (s, 3H), 0.69 (s, 3H).

^{13}C-NMR (75 MHz, CDCl$_3$) δ (ppm) 159.0 (C), 156.2 (C), 147.6 (C), 146.7 (C), 146.3 (C), 145.0 (C), 144.3 (C), 141.1 (C), 139.0 (C), 138.2 (C), 136.2 (C), 130.1 (CH), 129.6 (CH), 129.5 (CH), 128.8 (CH), 128.2 (CH), 127.4 (CH), 126.5 (CH), 126.4 (CH), 125.8 (CH), 122.2 (C), 121.9 (CH), 112.5 (CH), 110.0 (CH), 109.5 (CH), 97.7 (CH), 55.9 (CH$_3$), 55.73 (CH$_3$), 55.65 (CH$_3$), 55.4 (CH$_3$), 50.0 (CH$_2$), 48.0 (C), 30.4 (CH$_3$), 27.3 (CH$_3$).

HRMS (ESI-TOF) m/z: $[M+H]^+$ calculated for $C_{34}H_{35}O_4$ 507.2530. Found 507.2539.

(*Z*)-5-((2,4-Methoxyphenyl)(phenyl)methylene)-6,6-dimethyl-6,7-dihydro-5*H*-dibenzo[*a*,*c*]cycloheptadiene (4ab)

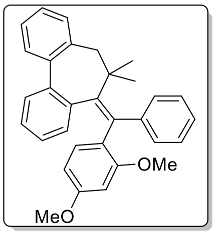

Obtained as white solid (30.3 mg, 0.07 mmol, 42%) from **1a** (50 mg, 0.16 mmol) and 1,3-dimethoxybenzene (17 mg, 0.18 mmol, 1.1 equiv.). R_f = 0.17 (Hexane). M.p.:150–152 °C. The compound is slightly contaminated with other regioisomer formed in the reaction that could not be isolated.

^{1}H-NMR (300 MHz, $CDCl_3$) δ (ppm) 7.60 (d, *J* = 7.7 Hz, 1H), 7.51 (dd, *J* = 7.6, 1.4 Hz, 1H), 7.44 (td, *J* = 7.5, 1.4 Hz, 1H), 7.30 (td, *J* = 7.5, 1.4 Hz, 1H), 7.26 (t, *J* = 7.5 Hz, 1H), 7.23–7.17 (m, 3H), 7.15–7.08 (m, 2H), 7.02 (td, *J* = 7.5, 1.4 Hz, 1H), 6.97 (td, *J* = 7.5, 1.4 Hz, 1H), 6.65 (d, *J* = 7.7 Hz, 1H), 6.25 (d, *J* = 8.3 Hz, 1H), 6.10 (d, *J* = 2.4 Hz, 1H), 6.01 (dd, *J* = 8.3, 2.4 Hz, 1H), 3.61 (2 x s, 6H), 2.69 (d, *J* = 12.7 Hz, 1H), 2.08 (d, *J* = 12.7 Hz, 1H), 1.41 (s, 3H), 0.70 (s, 3H).

^{13}C-NMR (75 MHz, $CDCl_3$) δ (ppm) 159.2 (C), 157.4 (C), 146.8 (C), 143.2 (C), 142.9 (C), 141.4 (C), 140.4 (C), 139.4 (C), 138.6 (C), 130.4 (CH), 129.9 (CH), 129.43 (CH), 129.42 (CH), 129.38 (CH), 128.4 (C), 127.2 (CH), 127.1 (CH), 127.04 (CH), 127.01 (CH), 127.00 (CH), 126.9 (CH), 126.69 (CH), 126.68 (CH), 125.9 (CH), 103.8 (CH), 98.4 (CH), 55.3 (CH_3), 55.2 (CH_3), 50.0 (CH_2), 48.2 (C), 30.1 (CH_3), 27.2 (CH_3).

HRMS (ESI-TOF) m/z: $[M+H]^+$ calculated for $C_{32}H_{30}O_2$ 447.2318. Found 447.2312.

(*Z*)-5-((2,4,6-Trimethoxyphenyl)(phenyl)methylene)-6,6-dimethyl-6,7-dihydro-5*H*-dibenzo[*a*,*c*]cycloheptadiene (4ac)

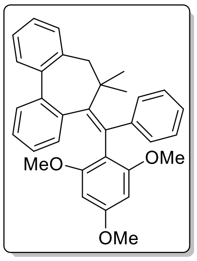

Obtained as white solid (40 mg, 0.08 mmol, 64%) from **1a** (50 mg, 0.16 mmol) and 1,3,5-trimethoxybenzene (30 mg, 0.18 mmol, 1.1 equiv.). R_f = 0.19 (Hexane/EtOAc 40:1). M.p.: 135–137 ºC.

H-NMR (300 MHz, $CDCl_3$) δ (ppm) 7.57 (dt, *J* = 7.7, 1.4 Hz, 1H), 7.49–7.46 (m, 1H), 7.39 (dd, *J* = 7.3, 1.3 Hz, 1H), 7.36–7.31 (m, 1H), 7.29–7.18 (m, 3H), 7.17–7.06 (m, 3H), 7.05–6.99 (m, 1H), 6.98–6.91 (m, 2H), 5.82 (d, *J* = 2.2 Hz, 1H), 5.68 (d, *J* = 2.2 Hz, 1H), 3.78 (s, 3H), 3.64 (s, 3H), 3.35 (s, 3H), 2.68 (d, *J* = 12.6 Hz, 1H), 2.06 (d, *J* = 12.6 Hz, 1H), 1.42 (s, 3H), 0.73 (s, 3H).

^{13}C-NMR (75 MHz, $CDCl_3$) δ (ppm) 159.5 (C), 157.9 (C), 155.6 (C), 147.5 (C), 144.4 (C), 142.3 (C), 141.5 (C), 139.6 (C), 138.3 (C), 132.6 (C), 130.1 (CH), 129.3 (CH), 129.2 (CH), 128.9 (CH), 127.5 (CH), 127.4 (CH), 127.3 (CH), 126.8 (CH), 126.7 (CH), 126.5 (CH), 126.1 (CH), 125.8 (CH), 125.7 (CH), 116.4 (C), 89.8 (CH), 89.6 (CH), 55.4 (CH_3), 55.2 (2 x CH_3), 50.2 (CH_2), 48.6 (C), 30.6 (CH_3), 27.3 (CH_3).

HRMS (ESI-TOF) m/z: $[M+H]^+$ calculated for $C_{33}H_{33}O_3$ 477.2424. Found 477.2421.

Mixture of (*Z*)-5-((4-methoxynaphthalen-1-yl)(phenyl)methylene)-6,6-dimethyl-6,7-dihydro-5*H*-dibenzo[*a*,*c*]cycloheptadiene (4ad) and (*Z*)-5-((1-methoxynaphthalen-2-yl)(phenyl)methylene)-6,6-dimethyl-6,7-dihydro-5*H*-dibenzo[*a*,*c*]cycloheptadiene (4ad')

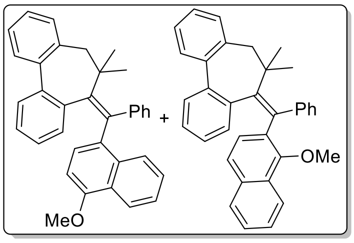

Obtained as white solid (66 mg, 0.14 mmol, 88%) from **1a** (50 mg, 0.16 mmol) and 1-methoxynaphthalene (28 mg, 0.17 mmol, 1.1 equiv.) as an inseparable 1.5:1 (maj/min) mixture of **4ad**/**4ad'** regioisomers. R_f = 0.31 (Hexane/DCM 8:2).

^{1}H-NMR (300 MHz, $CDCl_3$) δ (ppm) 8.05–7.99 (m, 2H, min), 7.96–7.93 (m, 1H, maj), 7.58 (dd, *J* = 7.7, 1.7 Hz, 1H, maj), 7.54 (d, *J* = 7.6 Hz, 1H, min), 7.50–7.42 (m, 2H, maj + min), 7.40–6.89 (m, 23H), 6.84 (td, *J* = 7.4, 1.4 Hz, 1H, maj), 6.78– 6.72 (m, 2H, maj), 6.65 (dd, *J* = 7.8, 1.7 Hz, 1H, min), 6.63–6.57 (m, 1H, maj), 6.53–6.44 (m, 2H, maj + min), 6.33 (d, *J* = 7.9 Hz, 1H, min), 3.78 (s, 3H, maj), 3.77 (s, 3H, min), 2.71 (d, *J* = 12.8 Hz, 2H, maj + min), 2.12 (dd, *J* = 12.7, 1.8 Hz, 2H, maj + min), 1.54 (s, 3H, min), 1.47 (s, 3H, maj), 0.87 (s, 3H, maj), 0.81 (s, 3H, min).

^{13}C-NMR (75 MHz, $CDCl_3$) δ (ppm) 154.0 (C, min), 153.3 (C, maj), 148.7 (C, min), 148.4 (C, maj), 145.4 (C, maj), 143.1 (C, min), 142.9 (C, min), 141.6 (C, min), 141.1 (C, min), 140.9 (C, maj), 140.8 (C, maj), 140.3 (C, min), 140.1 (C, maj), 138.5 (C, maj), 138.4 (C maj + C min), 135.7 (C, min), 135.3 (C, maj), 133.0 (CH min + C maj), 132.6 (C, min), 130.7 (C, maj), 130.2 (CH, min), 129.7 (CH, min), 129.5 (CH, min), 129.3 (CH, maj), 129.1 (CH, min), 129.0 (CH, maj), 128.1 (CH, maj), 128.0 (CH, maj), 127.91 (CH, maj), 127.86 (CH, maj), 127.8 (CH, maj), 127.7 (CH, maj), 127.6 (CH, min), 127.2 (CH, min), 127.1 (2 x CH, maj), 127.03 (2 x CH, min), 127.0 (CH, maj), 126.96 (CH, min), 126.8 (CH, min), 126.6 (CH, maj), 126.5 (CH, min), 126.3 (4 x CH, maj + min), 126.0 (CH, maj), 125.83 (CH, min), 125.77 (CH, min), 125.5 (CH, maj), 125.47 (C, min), 125.1 (C, maj), 124.5 (CH, maj), 124.4 (CH, min), 122.0 (CH, min), 121.3 (CH, maj), 103.1 (CH, min), 103.0 (CH, maj), 55.34 (CH_3, maj), 55.29 (CH_3, min), 50.2 (CH_2, maj), 50.1 (CH_2, min), 49.4 (C, maj), 48.6 (C, min), 30.8 (CH_3, maj), 30.5 (CH_3, min), 27.5 (CH_3, maj), 27.0 (CH_3, min).

HRMS (APCI) m/z: $[M+H]^+$ calculated for $C_{35}H_{31}O$ 467.2369. Found 467.2373.

(*Z*)-5-((4-Methoxyphenyl)(phenyl)methylene)-6,6-dimethyl-6,7-dihydro-5*H*-dibenzo[*a*,*c*]cycloheptadiene (4ae)

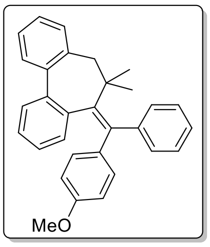

Obtained as white solid (40 mg, 0.09 mmol, 60%) from **1a** (50 mg, 0.16 mmol) and anisole (19 mg, 0.18 mmol, 1.1 equiv.). R_f = 0.10 (Hexane/DCM 9:1). M.p.: 121–123 ºC.

^{1}H-NMR (300 MHz, $CDCl_3$) δ (ppm) 7.59 (dd, *J* = 7.4, 1.4 Hz, 1H), 7.50 (d, *J* = 7.5 Hz, 1H), 7.45 (dd, *J* = 7.6, 1.4 Hz, 1H), 7.38–7.28 (m, 3H), 7.22–7.14 (m, 3H), 7.09 (td, *J* = 7.2, 1.3 Hz, 1H), 7.01–6.91 (m, 2H), 6.66 (d, *J* = 7.5 Hz, 1H), 6.50 (d, *J* = 9.0 Hz, 2H), 6.41 (d, *J* = 9.0 Hz, 2H), 3.62 (s, 3H), 2.75 (d, *J* = 12.7 Hz, 1H), 2.13 (d, *J* = 12.7 Hz, 1H), 1.41 (s, 3H), 0.75 (s, 3H).

^{13}C-NMR (75 MHz, $CDCl_3$) δ (ppm) 157.1 (C), 146.2 (C), 144.6 (C), 142.6 (C), 141.8 (C), 141.7 (C), 141.3 (C), 138.6 (C), 138.1 (C), 132.5 (CH), 130.4 (2 x CH), 130.2 (CH), 129.5 (CH), 129.0 (CH), 128.2 (CH), 127.5 (CH), 127.2 (CH), 127.1 (3 x CH), 127.0 (CH), 126.9 (CH), 126.5 (CH), 112.7 (2 x CH), 55.1 (CH_3), 50.1 (CH_2), 48.4 (C), 30.2 (CH_3), 27.2 (CH_3).

HRMS (APCI) m/z: $[M+H]^+$ calculated for $C_{31}H_{29}O$ 417.2213. Found 417.2222.

(*Z*)-5-((2,4,6-Trimethylphenyl)(phenyl)methylene)-6,6-dimethyl-6,7-dihydro-5*H*-dibenzo[*a*,*c*]cycloheptadiene (4af)

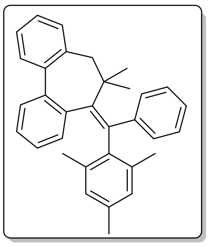

Obtained as colorless solid (32 mg, 0.07 mmol, 47%) from **1a** (50 mg, 0.16 mmol) and mesitylene (30 mg, 0.18 mmol, 1.1 equiv.). R_f = 0.26 (Hexane/DCM 9:1). M.p.: 143–144 °C.

^{1}H-NMR (300 MHz, $CDCl_3$) δ (ppm) 7.76 (dt, *J* = 7.6, 1.7 Hz, 1H), 7.54 (dd, *J* = 7.6, 1.4 Hz, 1H), 7.37 (td, *J* = 7.5, 1.4 Hz, 1H), 7.33–7.20 (m, 3H), 7.17–7.08 (m, 4H), 7.05 (td, *J* = 7.4, 1.5 Hz, 1H), 6.98–6.90 (m, 1H), 6.84–6.74 (m, 1H), 6.53 (bs, 1H), 6.48 (bs, 1H), 2.73 (d, *J* = 12.7 Hz, 1H), 2.36 (s, 3H), 2.09 (d, *J* = 12.7 Hz, 1H), 2.04 (s, 3H), 1.80 (s, 3H), 1.55 (s, 3H), 0.80 (s, 3H).

^{13}C-NMR (75 MHz, $CDCl_3$) δ (ppm) 147.3 (C), 143.3 (C), 141.7 (C), 141.1 (C), 141.0 (C), 139.5 (C), 138.9 (C), 138.2 (C), 135.9 (C), 134.8 (C), 133.9 (C), 130.22 (CH), 130.18 (CH), 129.4 (CH), 128.8 (CH), 128.4 (CH), 128.3 (CH), 128.2 (CH), 128.1 (CH), 128.0 (CH), 127.0 (CH), 126.9 (CH), 126.73 (CH), 126.67 (CH), 126.3 (CH), 126.2 (CH), 50.7 (CH_2), 49.1 (C), 31.6 (CH_3), 26.8 (CH_3), 22.3 (CH_3), 21.6 (CH_3), 20.9 (CH_3).

HRMS (ESI-TOF) m/z: $[M+H]^+$ calculated for $C_{33}H_{33}$ 429.2577. Found 429.2593.

(*Z*)-5-((5-Methylthien-2-yl)(phenyl)methylene)-6,6-dimethyl-6,7-dihydro-5*H*-dibenzo[*a*,*c*]cycloheptadiene (4ag)

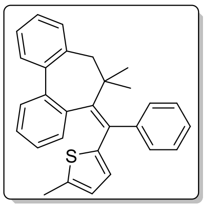

Obtained as white solid (40 mg, 0.09 mmol, 62%) from **1a** (50 mg, 0.16 mmol) and 5-methylthiophene (17 mg, 0.18 mmol, 1.1 equiv.). R_f = 0.17 (Hexane). M.p.: 150–152 ºC.

^{1}H-NMR (300 MHz, $CDCl_3$) δ (ppm) 7.61 (d, *J* = 7.5 Hz, 1H), 7.46–7.31 (m, 5H), 7.29 (d, *J* = 6.7 Hz, 1H), 7.25 (d, *J* = 7.2 Hz, 1H), 7.22–7.13 (m, 4H), 6.81 (d, *J* = 7.6 Hz, 1H), 6.17 (d, *J* = 2.7 Hz, 1H), 5.70 (d, *J* = 3.4 Hz, 1H), 2.71 (d, *J* = 12.8 Hz, 1H), 2.18 (s, 3H), 2.08 (d, *J* = 12.8 Hz, 1H), 1.34 (s, 3H), 0.65 (s, 3H).

^{13}C-NMR (75 MHz, $CDCl_3$) δ (ppm) 146.0 (C), 145.4 (C), 144.0 (C), 142.2 (C), 142.1 (C), 140.8 (C), 140.3 (C), 138.4 (C), 135.1 (C), 131.7 (CH), 131.1 (CH), 129.4 (CH), 129.2 (CH), 128.1 (CH), 127.9 (CH), 127.79 (CH), 127.77 (CH), 127.6 (CH), 127.5 (CH), 127.3 (CH), 127.13 (CH), 127.07 (CH), 127.0 (CH), 123.9 (CH), 50.2 (CH_2), 49.1 (C), 29.6 (CH_3), 27.3 (CH_3), 15.2 (CH_3).

HRMS (ESI-TOF) m/z: $[M+H]^+$ calculated for $C_{29}H_{27}S$ 407.1828. Found 407.1831.

(*Z*)-5-((*N*-Acetylindol-3-yl)(phenyl)methylene)-6,6-dimethyl-6,7-dihydro-5*H*-dibenzo[*a*,*c*]cycloheptadiene (4ah)

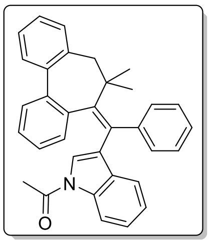

Obtained as white solid (45 mg, 0.10 mmol, 62%) from **1a** (50 mg, 0.16 mmol) and *N*-acetylindole (28 mg, 0.18 mmol, 1.1 equiv.). R_f = 0.60 (Hexane/EtOAc 8:2). M.p.: 79–81 ºC.

^{1}H-NMR (500 MHz, $CDCl_3$) δ (ppm) 8.18 (d, *J* = 8.3 Hz, 1H), 7.72 (d, *J* = 7.6 Hz, 1H), 7.54 (dd, *J* = 7.6, 1.4 Hz, 1H), 7.49 (td, *J* = 7.5, 1.3 Hz, 1H), 7.40 (td, *J* = 7.6, 1.4 Hz, 1H), 7.36 (dd, *J* = 7.4, 1.5 Hz, 1H), 7.31 (dd, *J* = 7.7, 1.3 Hz, 1H), 7.24 (dd, *J* = 7.4, 1.3 Hz, 1H), 7.23–7.16 (m, 1H), 7.14 (ddd, *J* = 8.3, 6.2, 2.3 Hz, 1H), 7.07 (ddd, *J* = 7.5, 6.1, 1.4 Hz, 2H), 7.04–6.97 (m, 2H), 6.92 (td, *J* = 7.5, 1.3 Hz, 1H), 6.80–6.73 (m, 1H), 6.49 (s, 1H), 2.78 (d, *J* = 12.7 Hz, 1H), 2.37 (s, 3H), 2.17 (d, *J* = 12.7 Hz, 1H), 1.49 (s, 3H), 0.81 (s, 3H).

^{13}C-NMR (500 MHz, $CDCl_3$) δ (ppm) 168.3 (C=O), 149.5 (C), 142.9 (C), 142.6 (C), 141.1 (C), 141.0 (C), 138.6 (C), 135.4 (C), 133.7 (C), 130.7 (CH), 130.3 (C), 129.9 (CH), 129.8 (CH), 129.6 (CH), 128.3 (CH), 127.57 (CH), 127.55 (CH), 127.4 (CH), 127.30 (CH), 127.28 (CH), 127.1 (CH), 126.9 (CH), 126.8 (CH), 126.4 (C), 124.6 (CH), 123.4 (CH), 123.3 (CH), 120.4 (CH), 116.1 (CH), 50.3 (CH_2), 48.9 (C), 30.2 (CH_3), 27.0 (CH_3), 23.9 (CH_3).

HRMS (ESI-TOF) m/z: $[M+H]^+$ calculated for $C_{34}H_{30}NO$ 468.2322. Found 468.2330.

(*Z*)-5-((*N*-Acetyl-5-bromoindol-3-yl)(phenyl)methylene)-6,6-dimethyl-6,7-dihydro-5*H*-dibenzo[*a*,*c*]cycloheptadiene (4ai)

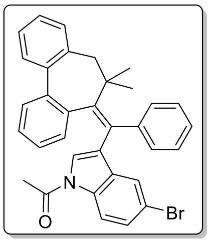

Obtained as white solid (57 mg, 0.10 mmol, 65%) from **1a** (50 mg, 0.16 mmol) and *N*-acetyl-5-bromoindole (42 mg, 0.18 mmol, 1.1 equiv.). R_f = 0.40 (Hexane/EtOAc 10:1). M.p.: 81–83 ºC.

^{1}H-NMR (500 MHz, $CDCl_3$) δ (ppm) 8.06 (d, *J* = 8.8 Hz, 1H), 7.69 (d, *J* = 7.6 Hz, 1H), 7.60 (dd, *J* = 7.6, 1.5 Hz, 1H), 7.51 (td, *J* = 7.4, 1.4 Hz, 1H), 7.42 (td, *J* = 7.5, 1.4 Hz, 1H), 7.36 (td, *J* = 7.4, 1.5 Hz, 1H), 7.30 (dd, *J* = 7.6, 1.4 Hz, 1H), 7.28–7.21 (m, 3H), 7.17 (dd, *J* = 7.5, 1.4 Hz, 1H), 7.13–7.06 (m, 3H), 6.94 (td, *J* = 77.5, 1.4 Hz, 1H), 6.75 (d, *J* = 7.7 Hz, 2H), 6.53 (s, 1H), 2.77 (d, *J* = 12.7 Hz, 1H), 2.3 (s, 3H), 2.16 (d, *J* = 12.7 Hz, 1H), 1.48 (s, 3H), 0.81 (s, 3H).

^{13}C-NMR (500 MHz, $CDCl_3$) δ (ppm) 168.2 (C=O), 150.2 (C), 142.5 (C), 142.3 (C), 141.0 (C), 140.7 (C), 138.4 (C), 134.0 (C), 132.7 (C), 131.7 (C), 130.9 (CH), 129.8 (CH), 129.6 (CH), 129.1 (CH), 128.4 (CH), 127.8 (CH), 127.7 (CH), 127.6 (CH), 127.51 (CH), 127.45 (CH), 127.3 (CH), 127.1 (3 x CH), 125.8 (C), 124.5 (CH), 122.8 (CH), 117.6 (CH), 116.8 (C), 50.1 (CH_2), 49.0 (C), 30.2 (CH_3), 26.9 (CH_3), 23.8 (CH_3).

HRMS (ESI-TOF) m/z: $[M+H]^+$ calculated for $C_{34}H_{29}BrNO$ 546.1427. Found 546.1429.

General procedure for the synthesis of dibenzocyclooctadienones 3:

To a solution of the corresponding 2-alkenyl-2`-alkynyl-1,1`-biphenyl **1** (1 equiv., 0.12-0.20 mmol) in DCE (0.1 M), in a sealed tube under argon atmosphere, was added distilled water (1.1 equiv., 0.13-0.22 mmol) and trifluoromethanesulfonic acid (10 mol%). The resulted reaction mixture was stirred at 60 ºC until complete consumption of the starting enyne **1**, as monitored by TLC or GC/MS. Then, solvent was removed under reduced pressure and the crude reaction mixture was purified directly by flash chromatography on silica gel, using mixtures of hexane and DCM as eluents to give the corresponding dibenzocyclooctadienone **3**.

Characterization data of dibenzocyclooctadienones 3:

6,7,7-Trimethyl-7,8-dihydrodibenzo[*a*,*c*]cyclooctadienone (3b)

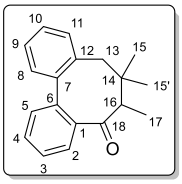

Obtained as colorless oil (32 mg, 0.12 mmol, 99%) from **1b** (30 mg, 0,12 mmol). R_f = 0.27 (Hexane/DCM 8:2).

^{1}H-NMR (500 MHz, $CDCl_3$) δ (ppm) 8.38 (ddd, *J* = 8.0, 1.5, 0.4 Hz, 1H, H-2), 7.56 (ddd, *J* = 7.6, 7.2, 1.5 MHz, 1H, H-4), 7.53–7.48 (m, 1H, H-8), 7.46 (ddd, *J* = 8.0, 7.2, 1.4 Hz, 1H, H-3), 7.42-7.36 (m, 2H, H-9,10), 7.28 (dd, *J* = 7.6, 1.3 Hz, 1H, H-5), 7.19 (dd, *J* = 7.2, 1.7 Hz, 1H, H-11), 2,72 (q, *J* = 6.6 Hz, 1H, H-16), 2.69 (d, *J* = 13.1 Hz, 1H, H-13), 2.42 (d, *J* = 13.1 Hz, 1H, H-13), 0.97 (s, 3H, H-15'), 0.94 (d, *J* = 6.6 Hz, 3H, H-17), 0.94 (s, 3H, H-15).

^{13}C-NMR (125 MHz, $CDCl_3$) δ (ppm) 203.2 (C=O, C-18), 142.4 (C, C-7), 141.1 (C, C-6), 136.9 (C, C-12), 136.8 (C, C-1), 134.4 (CH, C-5), 132.7 (CH, C-4), 130.1 (CH, C-8), 130.0 (CH, C-11), 129.8 (CH, C-2), 127.7 (CH, C-10), 127.5 (CH, C-3), 127.0 (CH, C-9), 47.9 (CH, C-16), 46.6 (CH_2, C-13), 39.6 (C, C-14), 26.6 (CH_3, C-15), 23.8 (CH_3, C-15'), 11.4 (CH_3, C-17).

HRMS (ESI-TOF) m/z: $[M+H]^+$ calculated for $C_{19}H_{21}O$ 265.1587. Found 265.1591.

11-Chloro-6,7,7-trimethyl-7,8-dihydrodibenzo[*a*,*c*]cyclooctadienone (3p)

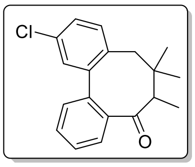

Obtained as colorless oil (43 mg, 0.14 mmol, 99%) from **1p** (40 mg, 0,14 mmol). R_f = 0.28 (Hexane/DCM 8:2).

^{1}H-NMR (300 MHz, $CDCl_3$) δ (ppm) 8.37 (dd, *J* = 8.0, 1.6 Hz, 1H), 7.58 (td, *J* = 7.4, 1.6 Hz, 1H), 7.51–7.44 (m, 2H), 7.35 (dd, *J* = 8.1, 2.2 Hz, 1H), 7.26 (dd, *J* = 7.7, 1.4 Hz, 1H), 7.11 (d, *J* = 8.1 Hz, 1H), 2.69 (q, *J* = 6.5 Hz, 1H), 2.64 (d, *J* = 13.2 Hz, 2H), 2.41 (d, *J* = 13.2 Hz, 2H), 0.98 (s, 3H), 0.97 (d, *J* = 6.5 Hz, 3H), 0.94 (s, 3H).

^{13}C-NMR (75 MHz, $CDCl_3$) δ (ppm) 202.4 (C=O), 143.9 (C), 139.6 (C), 136.6 (C), 135.3 (C), 134.0 (CH), 132.7 (CH), 132.4 (C), 131.2 (CH), 129.9 (CH), 129.8 (CH), 128.0 (CH), 127.6 (CH), 47.9 (CH), 46.1 (CH_2), 39.6 (C), 26.6 (CH_3), 23.8 (CH_3), 11.5 (CH_3).

HRMS (ESI-TOF) m/z: $[M+H]^+$ calculated for $C_{19}H_{20}ClO$ 299.1197. Found 299.1208.

3-Chloro-6,7,7-trimethyl-7,8-dihydrodibenzo[*a*,*c*]cyclooctadienone (3q)

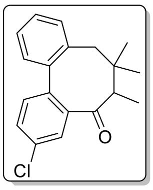

Obtained as white solid (40 mg, 0.13 mmol, 95%) from **1q** (40 mg, 0.14 mmol). R_f = 0.27 (Hexane/DCM 8:2). M.p.: 59–60 ºC.

^{1}H-NMR (300 MHz, $CDCl_3$) δ (ppm) 8.34 (d, *J* = 2.5 Hz, 1H), 7.51 (dd, *J* = 8.5, 2.5 Hz, 1H), 7.48–7.37 (m, 3H), 7.26–7.17 (m, 2H), 2.69 (q, *J* = 6.9 Hz, 1H), 2.63 (d, *J* = 13.2 Hz, 1H), 2.43 (d, *J* = 13.2 Hz, 1H), 0.96 (s, 3H), 0.93 (d, *J* = 6.9 Hz, 3H), 0.93 (s, 3H).

^{13}C-NMR (75 MHz, $CDCl_3$) δ (ppm) 201.9 (C=O), 141.1 (C), 139.4 (C), 137.8 (C), 136.8 (C), 135.8 (CH), 134.2 (C), 132.4 (CH), 130.2 (CH), 130.0 (CH), 129.7 (CH), 128.1 (CH), 127.2 (CH), 47.8 (CH), 46.5 (CH_2), 39.8 (C), 26.6 (CH_3), 23.8 (CH_3), 11.4 (CH_3).

HRMS (ESI-TOF) m/z: $[M+H]^+$ calculated for $C_{19}H_{20}ClO$ 299.1197. Found 299.1204.

10-Methoxy-6,7,7-trimethyl-7,8-dihydrodibenzo[*a*,*c*]cyclooctadienone (3r)

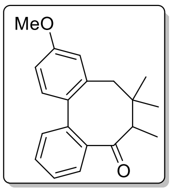

Obtained as pale grey oil (30 mg, 0.10 mmol, 82%) from **1r** (34 mg, 0,12 mmol). R_f = 0.18 (Hexane/DCM 8:2).

^{1}H-NMR (300 MHz, $CDCl_3$) δ (ppm) 8.36 (dd, *J* = 8.0, 1.6 Hz, 1H), 7.54 (ddd, *J* = 7.8, 7.2, 1.6 Hz, 1H), 7.47–7.39 (m, 2H), 7.26 (dd, *J* = 7.7, 1.4 Hz, 1H), 6.95 (dd, *J* = 8.6, 2.8 Hz, 1H), 6.73 (d, *J* = 2.8 Hz, 1H), 3.88 (s, 3H), 2.77 (q, *J* = 6.5 Hz, 1H), 2.66 (d, *J* = 13.1 Hz, 1H), 2.37 (d, *J* = 13.1 Hz, 1H), 0.97 (s, 3H), 0-96 (s, 3H), 0.95 (d, *J* = 6.5 Hz, 3H).

^{13}C-NMR (75 MHz, $CDCl_3$) δ (ppm) 203.3 (C=O), 159.2 (C), 141.0 (C), 138.4 (C), 136.6 (C), 134.9 (C), 134.3 (CH), 132.6 (CH), 131.3 (CH), 129.9 (CH), 127.1 (CH), 115.6 (CH), 112.3 (CH), 55.5 (CH_3), 47.8 (CH), 46.9 (CH_2), 39.6 (C), 26.8 (CH_3), 23.9 (CH_3), 11.5 (CH_3).

HRMS (ESI-TOF) m/z: $[M+H]^+$ calculated for $C_{20}H_{23}O_2$ 295.1693. Found 295.1702.

3-Methoxy-6,7,7-trimethyl-7,8-dihydrodibenzo[*a*,*c*]cyclooctadienone (3s)

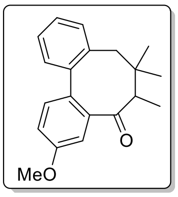

Obtained as yellow oil (36 mg, 0.13 mmol, 70%) from **1s** (50 mg, 0,18 mmol). R_f = 0.16 (Hexane/DCM 7:3).

^{1}H-NMR (300 MHz, $CDCl_3$) δ (ppm) 7.90 (d, *J* = 2.8 Hz, 1H), 7.47 (dd, *J* = 7.2, 1.8 Hz, 1H), 7.41–7.31 (m, 2H), 7.27–7.11 (m, 3H), 3.92 (s, 3H), 2.75 (q, *J* = 6.5 Hz, 1H), 2.69 (d, *J* = 13.0 Hz, 1H), 2.42 (d, *J* = 13.0 Hz, 1H), 0.99 (s, 3H), 0.98 (d, *J* = 6.5 Hz, 3H), 0.95 (s, 3H).

^{13}C-NMR (75 MHz, $CDCl_3$) δ (ppm) 202.8 (C=O), 158.8 (C), 141.9 (C), 137.6 (C), 136.7 (C), 135.8 (CH), 133.8 (C), 130.0 (CH), 129.9 (CH), 127.3 (CH), 126.9 (CH), 120.1 (CH), 112.4 (CH), 55.6 (CH_3), 47.6 (CH), 46.7 (CH_2), 40.0 (C), 26.6 (CH_3), 24.0 (CH_3), 11.6 (CH_3).

HRMS (ESI-TOF) m/z: $[M+H]^+$ calculated for $C_{20}H_{23}O_2$ 295.1693. Found 295.1697.

11-Chloro-1,3-dimethoxy-6,7,7-trimethyl-7,8-dihydrodibenzo[*a*,*c*]cyclooctadienone (3t)

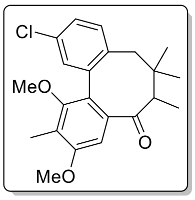

Obtained as colorless oil (24 mg, 0.07 mmol, 45%) from **1t** (52 mg, 0.15 mmol). R_f = 0.22 (Hexane/DCM 7:3).

^{1}H-NMR (300 MHz, $CDCl_3$) δ (ppm) 7.68 (s, 1H), 7.51 (d, *J* = 2.3 Hz, 1H), 7.33 (dd, *J* = 8.2, 2.3 Hz, 1H), 7.13 (d, *J* = 8.2 Hz, 1H), 3.93 (s, 3H), 3.00 (s, 3H), 2.66 (d, *J* = 13.1 Hz, 1H), 2.50 (q, *J* = 6.7 Hz, 1H), 2.39 (d, *J* = 13.1 Hz, 1H), 2.21 (s, 3H), 0.93 (s, 3H), 0.92 (d, *J* = 6.7 Hz, 3H), 0.89 (s, 3H).

^{13}C-NMR (75 MHz, $CDCl_3$) δ (ppm) 202.0 (C), 158.1 (C), 157.0 (C), 137.8 (C), 136.5 (C), 136.3 (C), 132.0 (CH), 131.4 (C), 130.2 (CH), 127.5 (CH), 126.6 (C), 126.1 (C), 106.5 (CH), 59.4 (CH_3), 55.7 (CH_3), 48.3 (CH), 46.0 (CH_2), 39.0 (C), 27.0 (CH_3), 23.9 (CH_3), 11.6 (CH_3), 9.9 (CH_3).

HRMS (ESI-TOF) m/z: $[M+H]^+$ calculated for $C_{22}H_{26}ClO_3$ 373.1565. Found 373.1573.

Synthesis of 8-(3,4-dimethoxyphenyl)-6,6,7-trimethyl-5,6-dihydrodibenzo[*a*,*c*] cyclooctatriene (6ba):

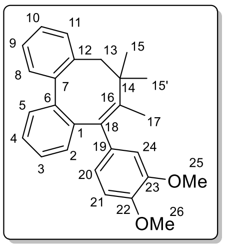

To a solution of biphenyl **1b** (1 equiv., 0.12 mmol, 30 mg) in DCE (0.1 M), in a sealed tube under Ar atmosphere, was added 1,2-dimethoxybenzene (1.1 equiv., 0.13 mmol, 18 mg) and trifluoromethanesulfonic acid (10 mol%, 1 μL). The resulted reaction mixture was stirred at 30 ºC for 1 h. Then, solvent was removed under reduced pressure and the crude reaction mixture was directly purified by flash chromatography on silica gel, using a mixture of Hexane/EtOAc 9:1 as eluent to give dibenzocyclooctatriene **6ba** (yellow oil, 36 mg, 0.07 mmol, 58%). R_f = 0.35 (Hexane/EtOAc 9:1).

^{1}H-NMR (400 MHz, $CDCl_3$) δ (ppm) 7.35–7.33 (m, 1H), 7.25–7.21 (m, 2H), 7.19–7.06 (m, 5H), 6.57 (d, *J* = 8.1 Hz, 1H, H-21), 6.32 (d, *J* = 8.1 Hz, 1H, H-20), 6.15 (s, 1H, H-24), 3.69 (s, 3H, H-

26), 3.63 (s, 3H, H-25), 2.62 (d, *J* = 13.4 Hz, 1H, H-13), 2.29 (d, *J* = 13.4 Hz, 1H, H-13), 1.36 (s, 3H, H-17), 1.25 (s, 3H, H-15'), 1.13 (s, 3H, H-15).

^{13}C-NMR (100 MHz, CDCl$_3$) δ (ppm) 148.5 (C, C-23), 146.9 (C, C-22), 144.4 (C), 143.3 (C, C-7), 140.9 (C, C-16), 140.2 (C), 140.0 (C), 139.8 (C, C-19), 136.0 (C, C-18), 129.3 (CH), 128.73 (CH, C-11), 128.67 (CH), 127.3 (CH), 127.2 (CH), 127.0 (CH), 126.2 (2 x CH), 120.5 (CH, C-20), 112.1 (CH, C-24), 111.0 (CH, C-21), 55.84 (CH$_3$, C-26), 55.79 (CH$_3$, C-25), 48.0 (CH$_2$, C-13), 40.6 (C, C-14), 30.4 (CH$_3$, C-15), 29.7 (CH$_3$, C-15'), 19.5 (CH$_3$, C-17).

HRMS (ESI-TOF) m/z: [M+H]$^+$ calculated for $C_{27}H_{29}O_2$ 385.2162. Found 385.2162.

Synthesis of 8,8-dimethyl-6,7,8,9-tetrahydrodibenzo[5,6:7,8]cycloocta[1,2-*b*]furan (6u)

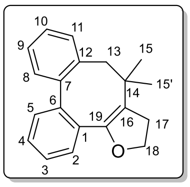

To a solution of **1u** (48 mg, 0.17 mmol, 1 equiv.) in DCE (0.05 M), in a sealed tube under Ar atmosphere, was added trifluoromethanesulfonic acid (10 mol%, 1.5 μL). The mixture was stirred overnight at 60 ºC. After completion, the solvent was removed under reduced pressure and the crude reaction mixture was purified directly by flash chromatography on silica gel, using a mixture of Hexane/DCM 8:2 as eluent to give dibenzocyclooctatriene **6u** as yellow oil (15 mg, 0.05 mmol, 31%). R_f = 0.41 (Hexane/DCM 8:2).

^{1}H-NMR (500 MHz, CDCl$_3$) δ (ppm) 7.54–7.49 (m, 1H, H-2), 7.39–7.33 (m, 2H, H-3,4), 7.33–7.26 (m, 3H, H-8,9,10), 7.24–7.17 (m, 2H, H-4,H-11), 4.23 (ddd, *J* = 10.8, 9.1, 8.5 Hz, 1H, H-18), 4.13 (ddd, *J* = 10.8, 8.5, 7.3 Hz, 1H, H-18), 2.92 (d, *J* = 13.2 Hz, 1H, H-13), 2.77 (ddd, *J* = 14.7, 10.8, 7.3 Hz, 1H, H-17), 2.63 (ddd, *J* = 14.7, 10.8, 9.1 Hz, 1H, H-17), 2.37 (d, *J* = 13.2 Hz, 1H, H-13), 1.20 (s, 3H, H-15), 1.19 (s, 3H, H-15').

^{13}C-NMR (125 MHz, CDCl$_3$) δ (ppm) 145.5 (C, C-19), 142.1 (C, C-7), 140.0 (C, C-6), 138.4 (C, C-12), 133.1 (C, C-1), 130.4 (CH, C-5), 129.9 (CH, C-11), 129.4 (CH, C-8), 129.1 (CH, C-2), 128.2 (CH, C-3), 127.2 (CH, C-4), 126.9 (CH, C-10), 126.2 (CH, C-9), 116.8 (C, C-16), 67.3 (CH$_2$, C-18), 45.4 (CH$_2$, C-13), 36.4 (C, C-14), 33.5 (CH$_2$, C-17), 31.0 (CH$_3$, C-15'), 27.6 (CH$_3$, C-15).

HRMS (ESI-TOF) m/z: [M+H]$^+$ calculated for $C_{20}H_{21}O$ 277.1587. Found 2771591.

Synthesis of 13-methoxy-10,10-dimethyl-10,11-dihydro-9*H*-dibenzo[5,6:7,8]cycloocta[1,2-a]indene (6v)

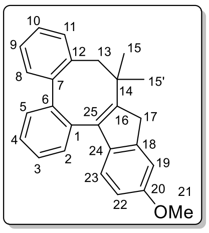

To a solution of **1v** (50 mg, 0.14 mmol, 1 equiv.) in DCE (0.05 M), in a sealed tube under Ar atmosphere, was added trifluoromethanesulfonic acid (10 mol%, 1.2 μL). The mixture was stirred overnight at 60 ºC. After completion, the solvent was removed under reduced pressure and the crude reaction mixture was purified directly by flash chromatography on silica gel, using a mixture of Hexane/DCM 8:2 as eluent to give dibenzocyclooctatriene **6v** as white solid (13 mg, 0.04 mmol, 29%). R_f = 0.38 (Hexane/DCM 8:2). M.p.: 136–138 ºC.

^{1}H-NMR (400 MHz, CDCl$_3$) δ (ppm) 7.39–7.29 (m, 3H, H-2,3,4), 7.26–7.23 (m, 1H, H-5), 7.22–7.17 (m, 1H, H-8), 7.16–7.09 (m, 3H, H-9,10,11), 6.85 (s, 1H, H-19), 6.71 (d, *J* = 8.5 Hz, 1H, H-23), 6.61 (d, *J* = 8.5 Hz, 1H, H-22), 3.68 (s, 3H, H-21), 3.38 (d, *J* = 22.4 Hz, 1H, H-17), 3.09 (d, *J* = 22.4 Hz, 1H, H-17'), 2.99 (d, *J* = 13.3 Hz, 1H, H-13), 2.34 (d, *J* = 13.3 Hz, 1H, H-13'), 1.35 (s, 3H, H-15), 1.15 (s, 3H, H-15').

^{13}C-NMR (100 MHz, CDCl$_3$) δ (ppm) 157.5 (C, C-20), 148.7 (C, C-16), 143.5 (C, C-25), 142.6 (C, C-7), 141.6 (C, C-24), 140.9 (C, C-6), 138.8 (C, C-12), 136.3 (C, C-1), 135.1 (C, C-18), 130.4 (CH, C-5), 129.3 (2 x CH, C-2,11), 128.6 (CH, C-8), 127.1 (2 x CH, C-3,4), 126.8 (CH, C-10), 126.2 (CH, C-9), 120.2 (CH, C-23), 111.7 (CH, C-22), 109.7 (CH, C-19), 55.8 (CH$_3$, C-21), 46.3 (CH$_2$, C-13), 39.7 (CH$_2$, C-17), 39.0 (C, C-14), 32.2 (CH$_3$, C-15'), 28.6 (CH$_3$, C-15).

HRMS (ESI-TOF) m/z: [M+H]$^+$ calculated for $C_{26}H_{25}O$ 353.1900. Found 353.1918.

Copies of ^1H and ^{13}C spectra for novel compounds and selected GCOSY, TOCSY, NOESY, GHSQC and GHMBC spectra

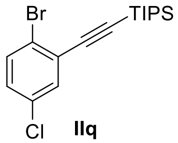

IIq

^{1}H-NMR (300 MHz, $CDCl_3$)

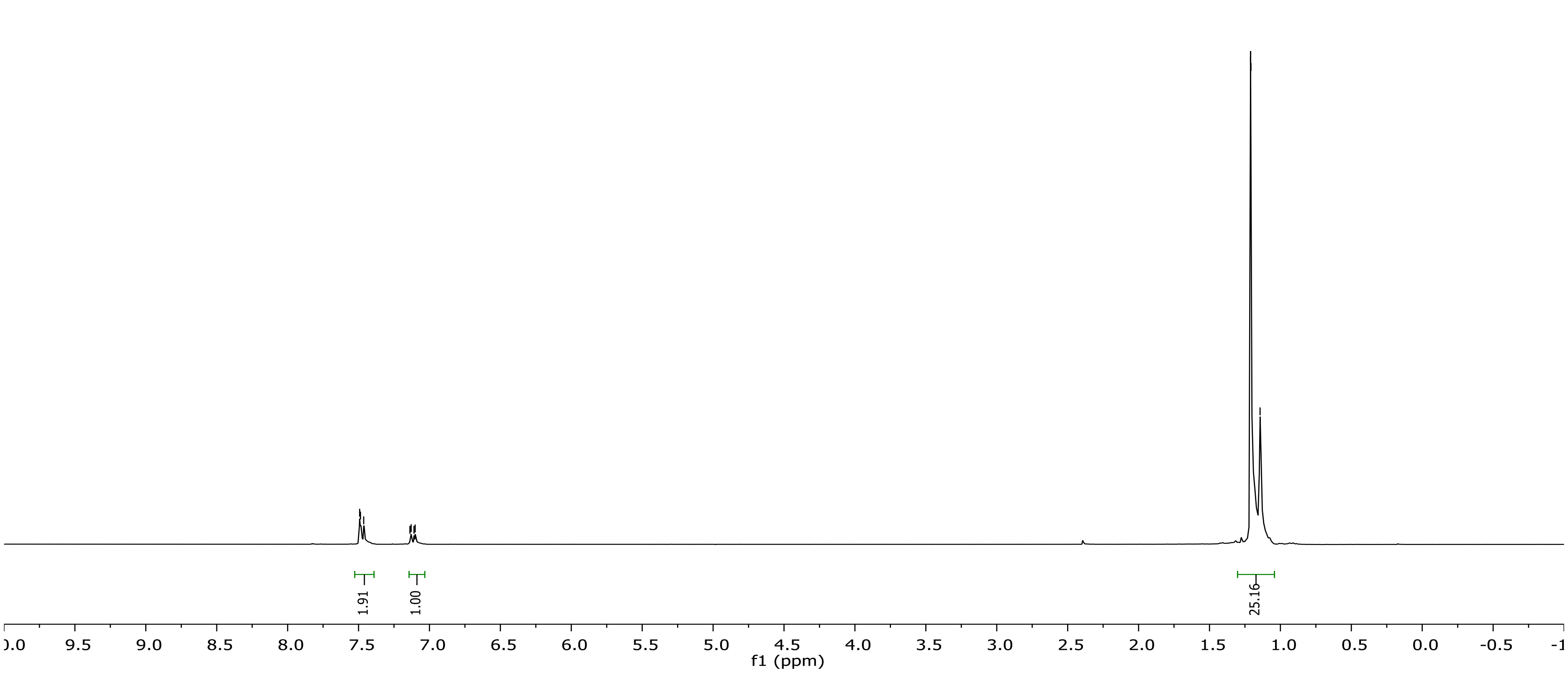

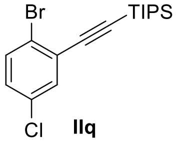

IIq

^{13}C-NMR (75 MHz, CDCl$_3$)

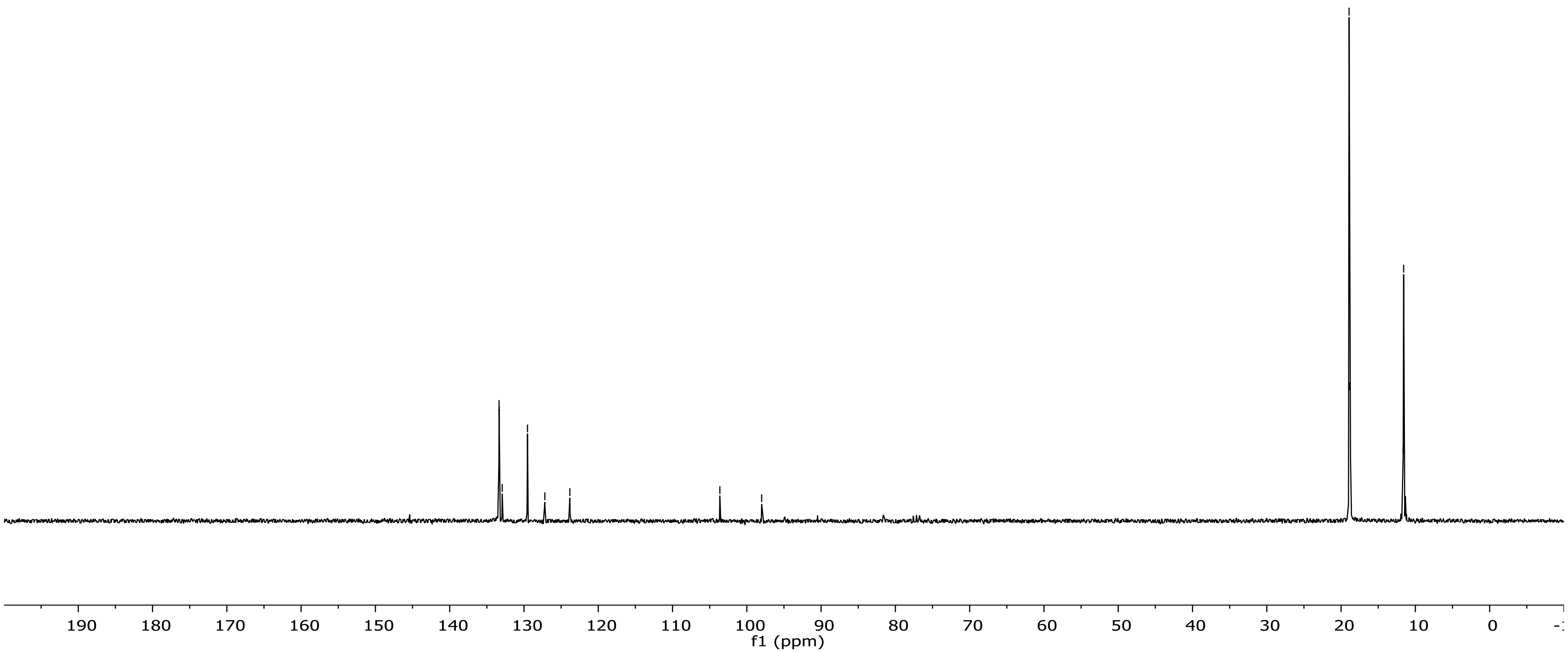

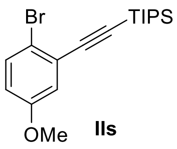

IIs

^{1}H-NMR (300 MHz, $CDCl_3$)

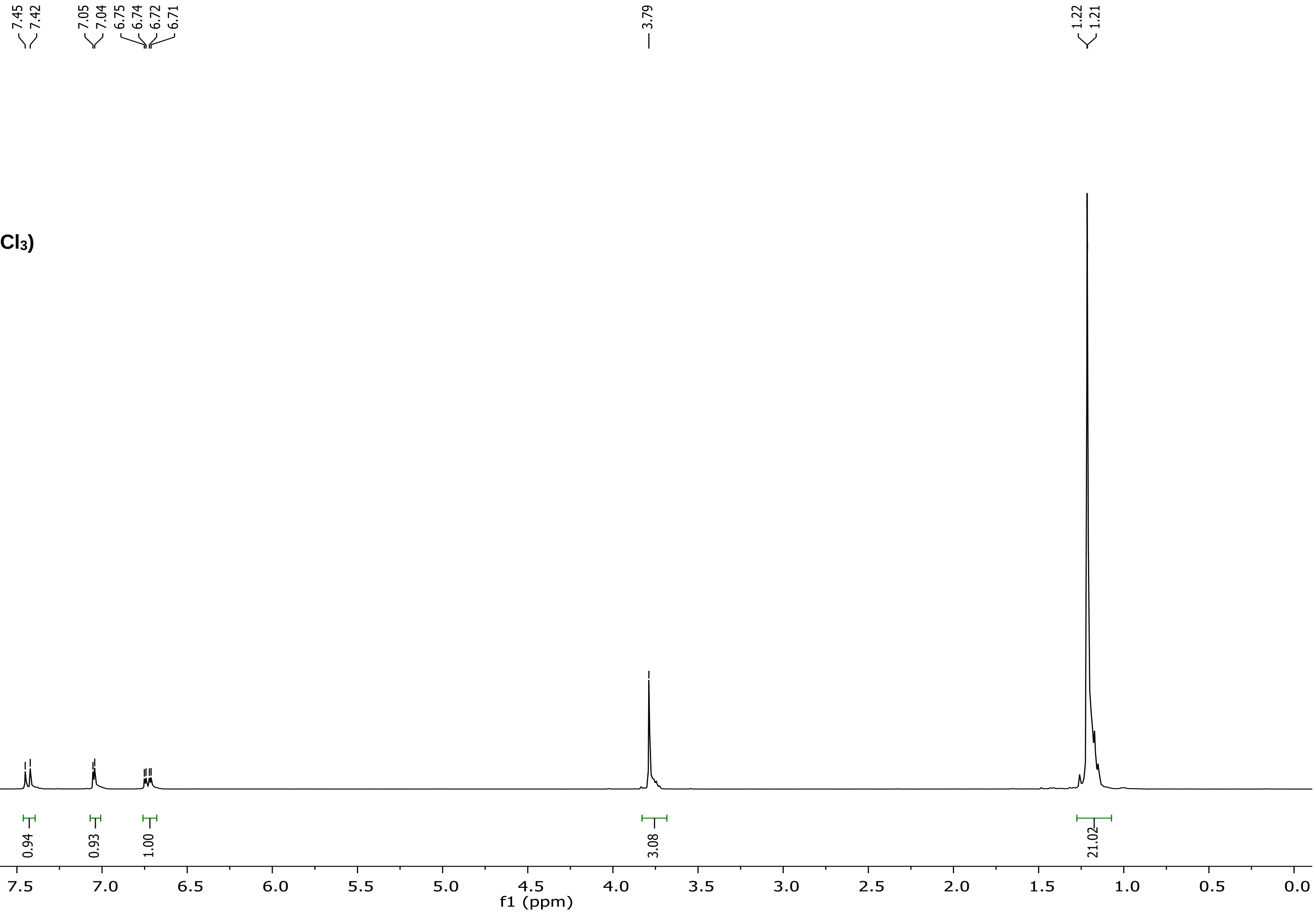

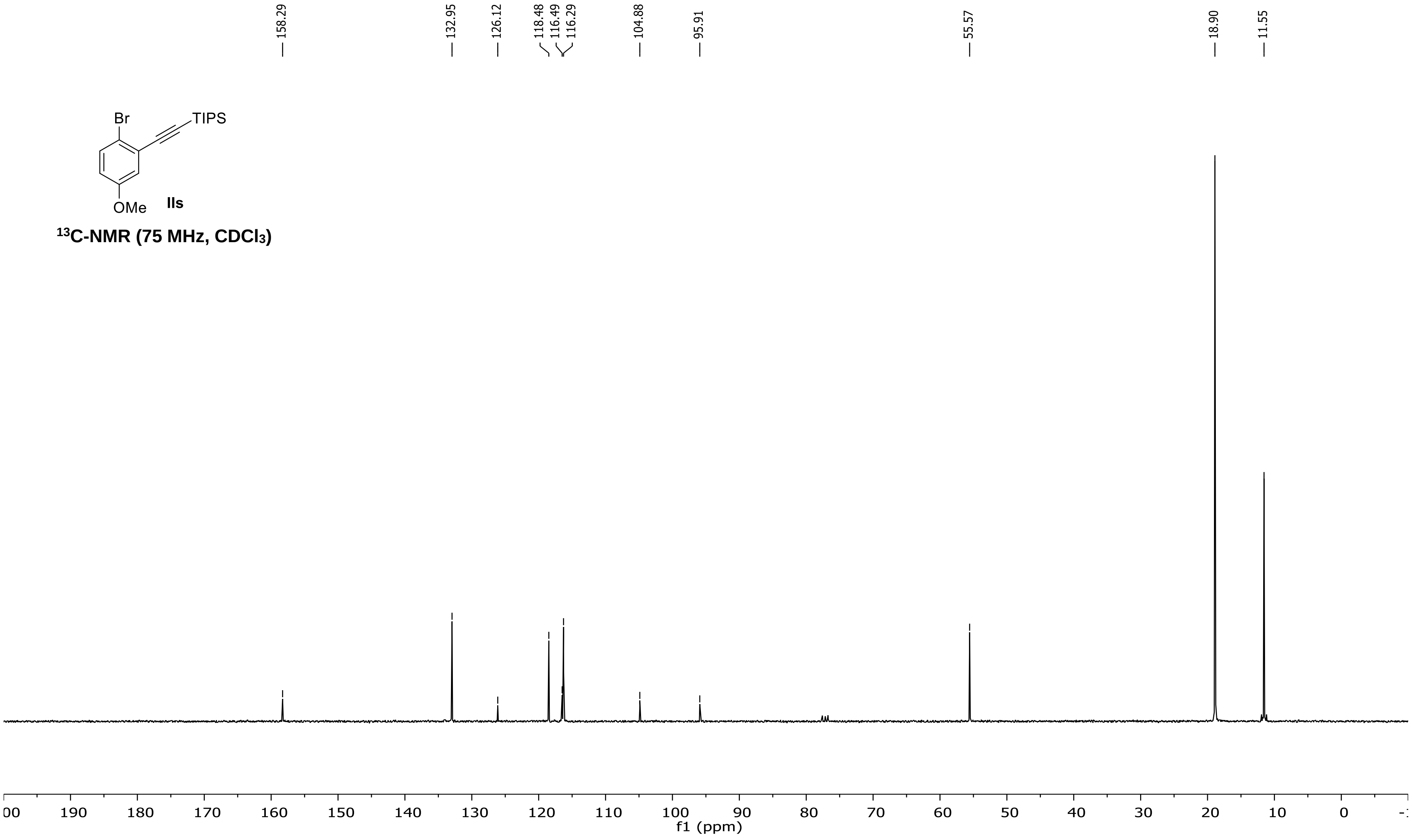

^{13}C-NMR (75 MHz, $CDCl_3$)

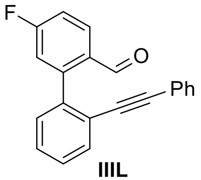

IIIL

^{1}H-NMR (300 MHz, $CDCl_3$)

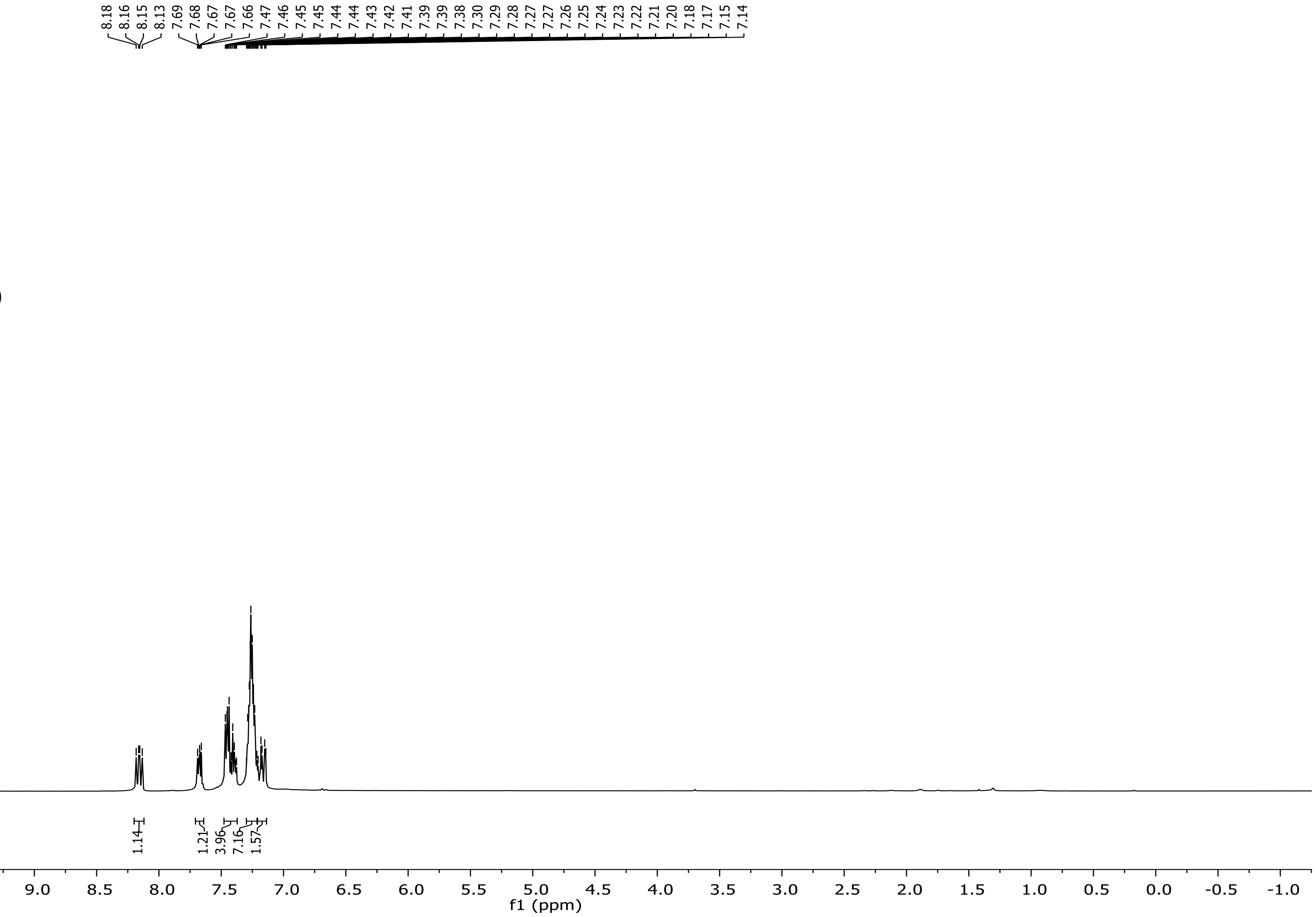

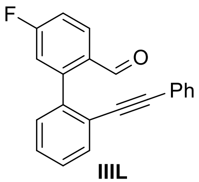

IIIL

^{13}C-NMR (75 MHz, CDCl₃)

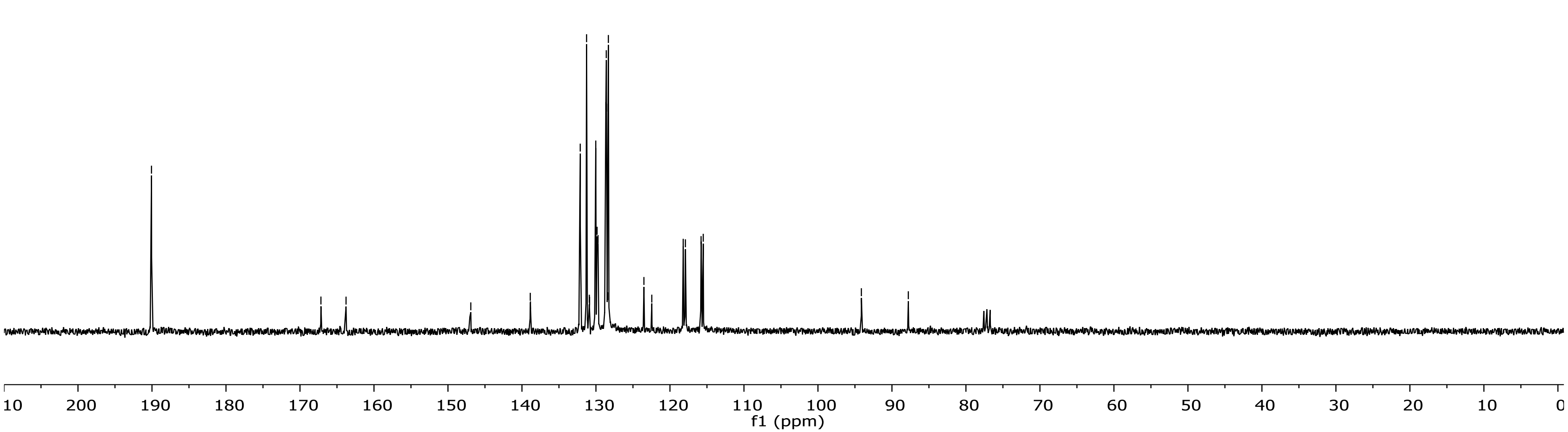

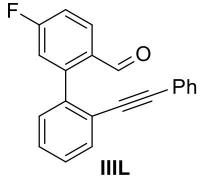

IIIL

^{19}F-NMR (283 MHz, $CDCl_3$)

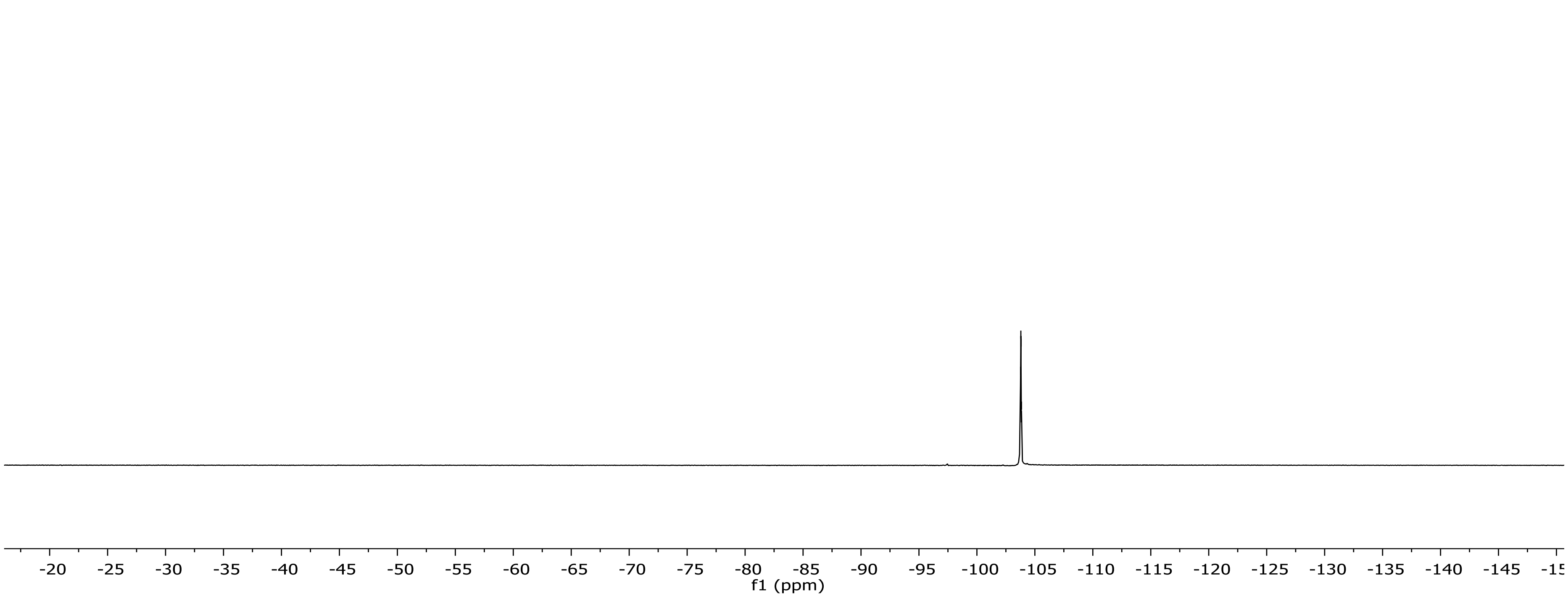

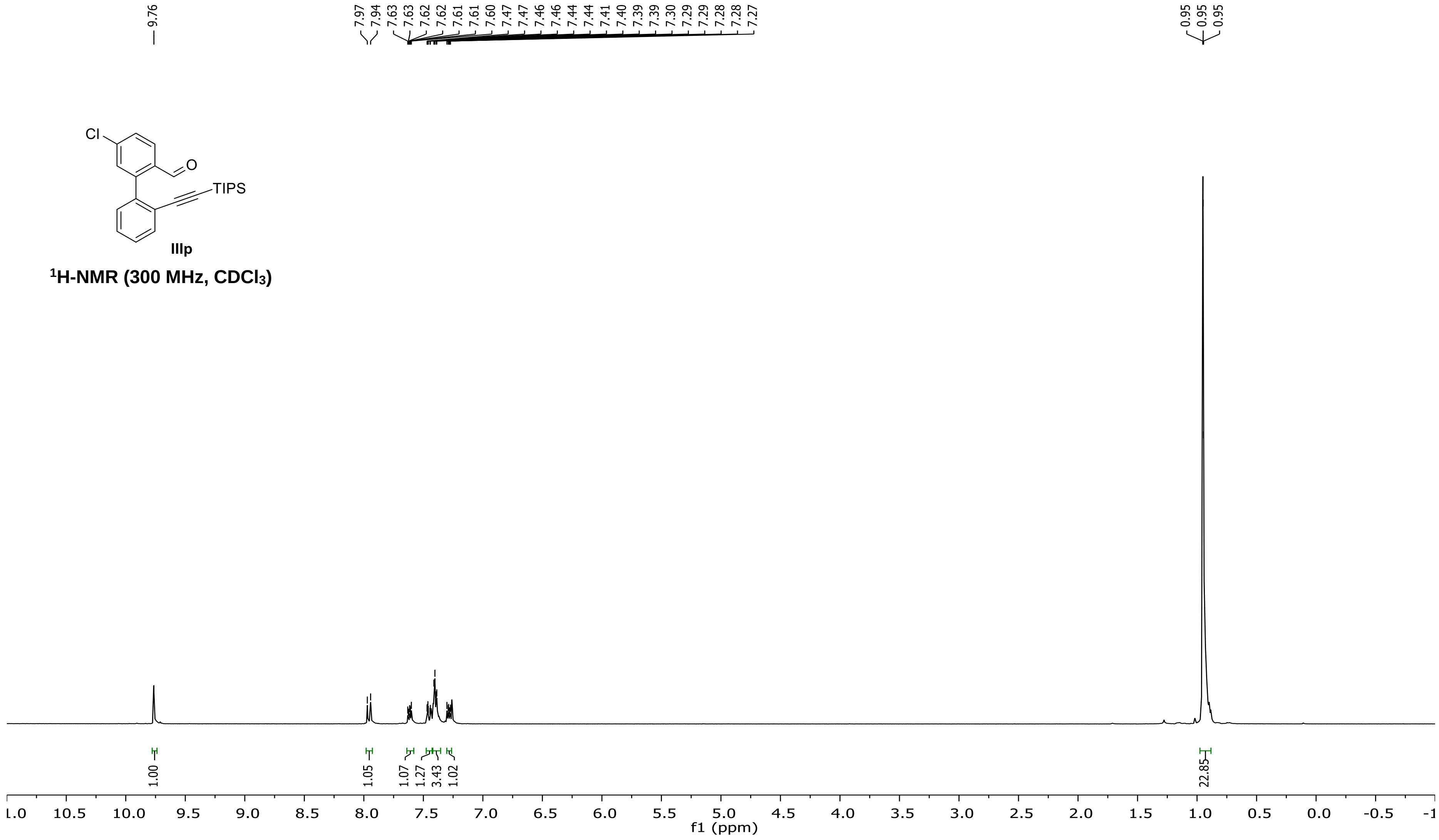

IIIp

^{1}H-NMR (300 MHz, CDCl$_3$)

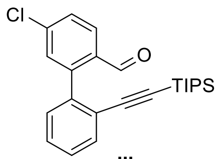

IIIp

^{13}C-NMR (75 MHz, $CDCl_3$)

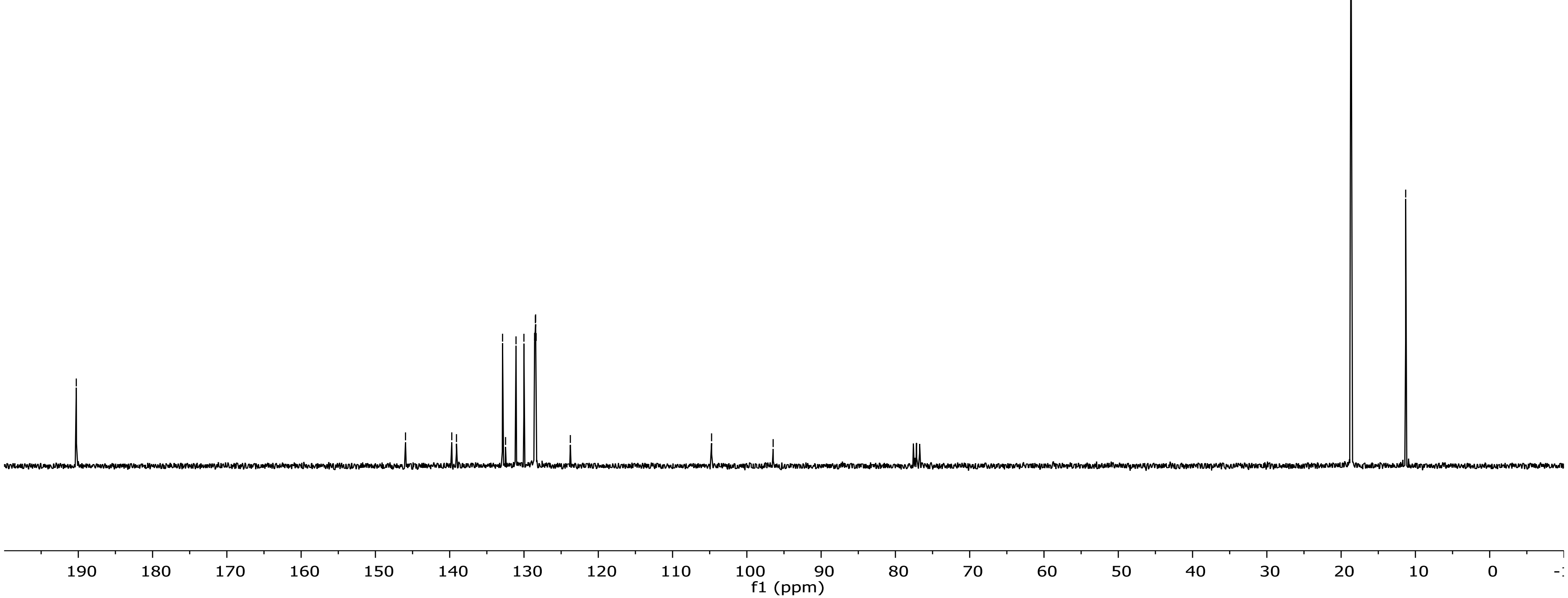

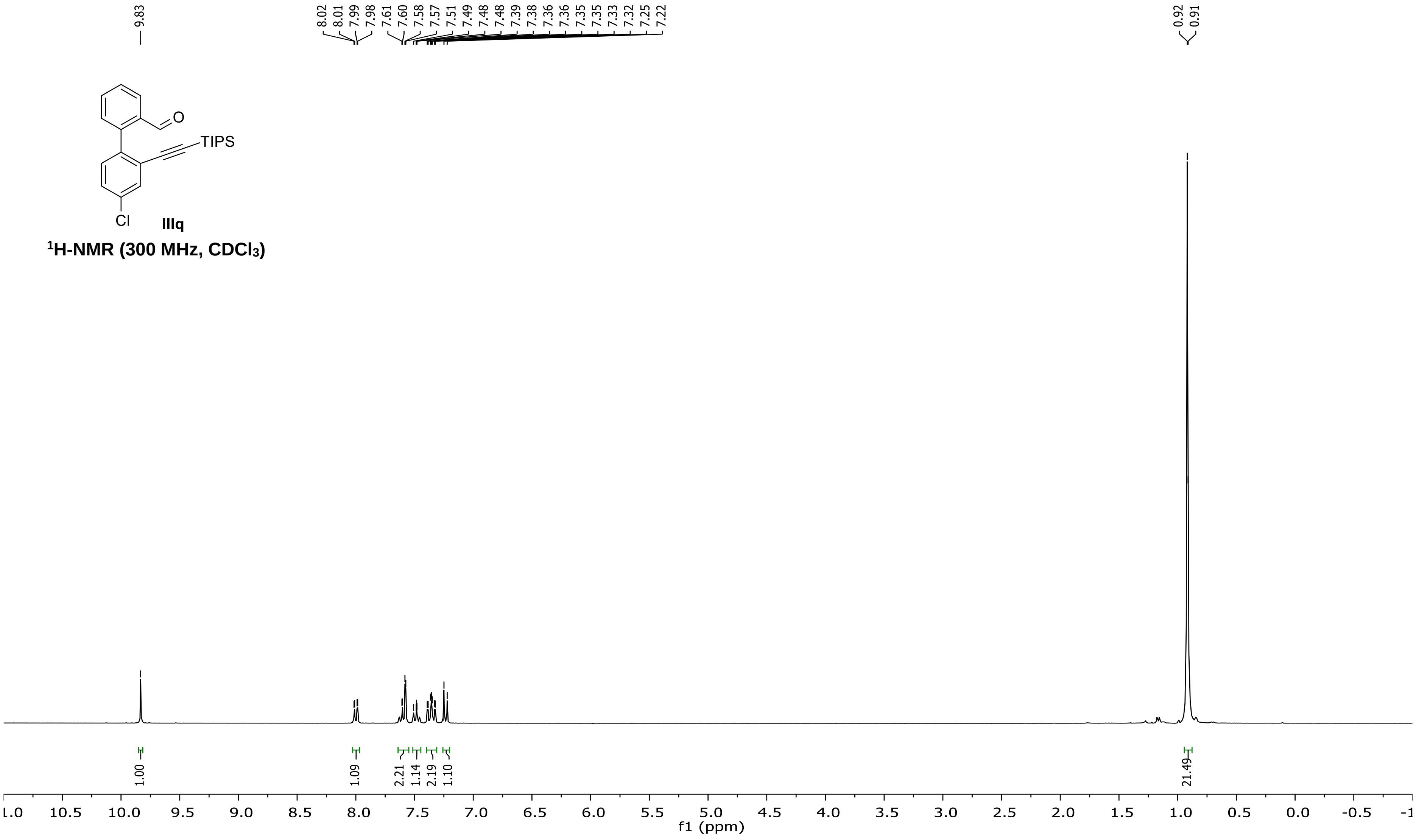
9.83
8.02
8.01
7.99
7.98
7.61
7.60
7.58
7.57
7.51
7.49
7.48
7.48
7.39
7.38
7.36
7.36
7.35
7.35
7.33
7.32
7.25
7.22
0.92
0.91
O
TIPS
Cl
IIIq
1H-NMR (300 MHz, CDCl3)
1.00
1.09
2.21
1.14
2.19
1.10
21.49
10.5
10.0
9.5
9.0
8.5
8.0
7.5
7.0
6.5
6.0
5.5
5.0
4.5
4.0
3.5
3.0
2.5
2.0
1.5
1.0
0.5
0.0
-0.5
f1 (ppm)

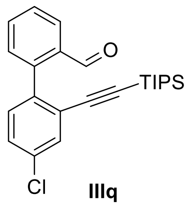

IIIq

^{13}C-NMR (75 MHz, CDCl$_3$)

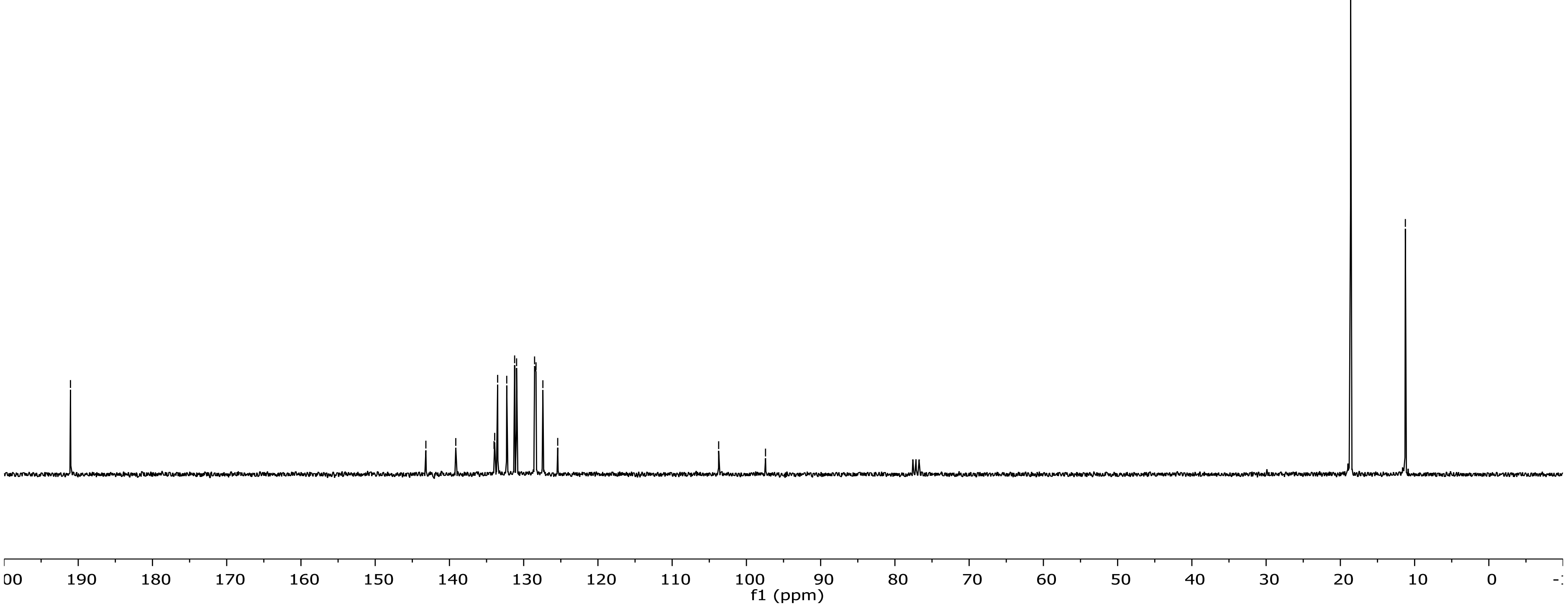

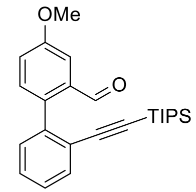

IIIr

^{1}H-NMR (300 MHz, CDCl$_3$)

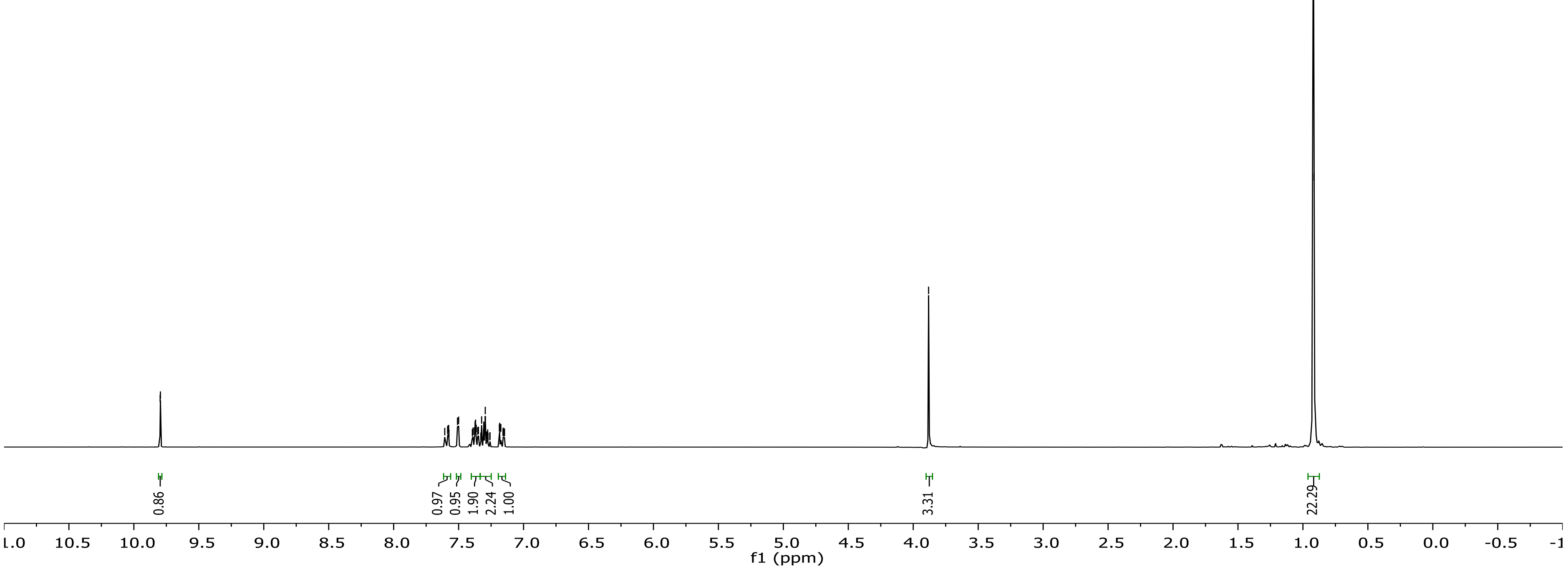

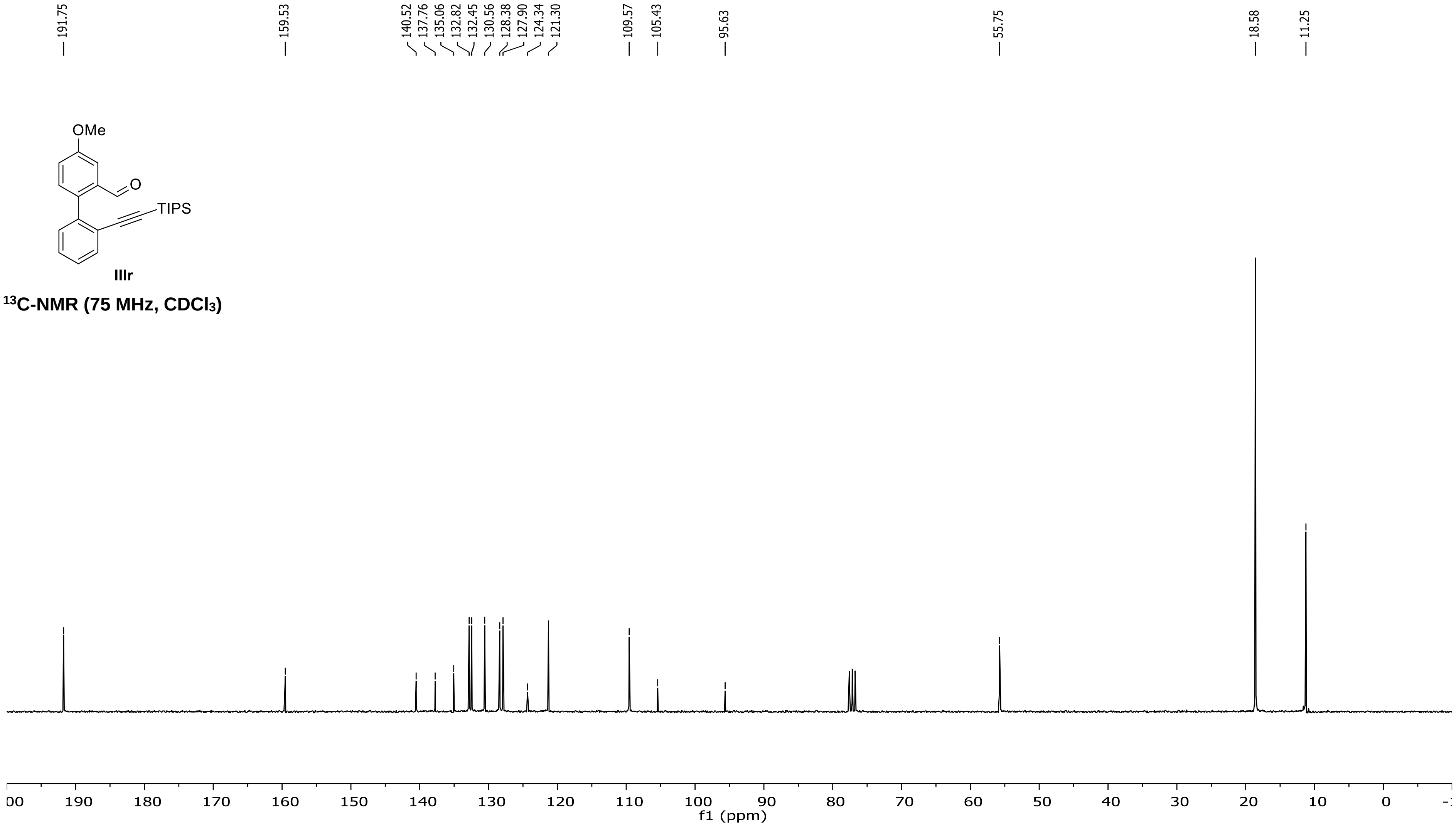

191.75
159.53
140.52
137.76
135.06
132.82
132.45
130.56
128.38
127.90
124.34
121.30
109.57
105.43
95.63
55.75
18.58
11.25
OMe
O
TIPS
IIIr
^{13}C-NMR (75 MHz, $CDCl_3$)
00
190
180
170
160
150
140
130
120
110
100
90
80
70
60
50
40
30
20
10
0
f1 (ppm)

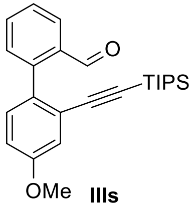

IIIs

^{1}H-NMR (300 MHz, CDCl$_3$)

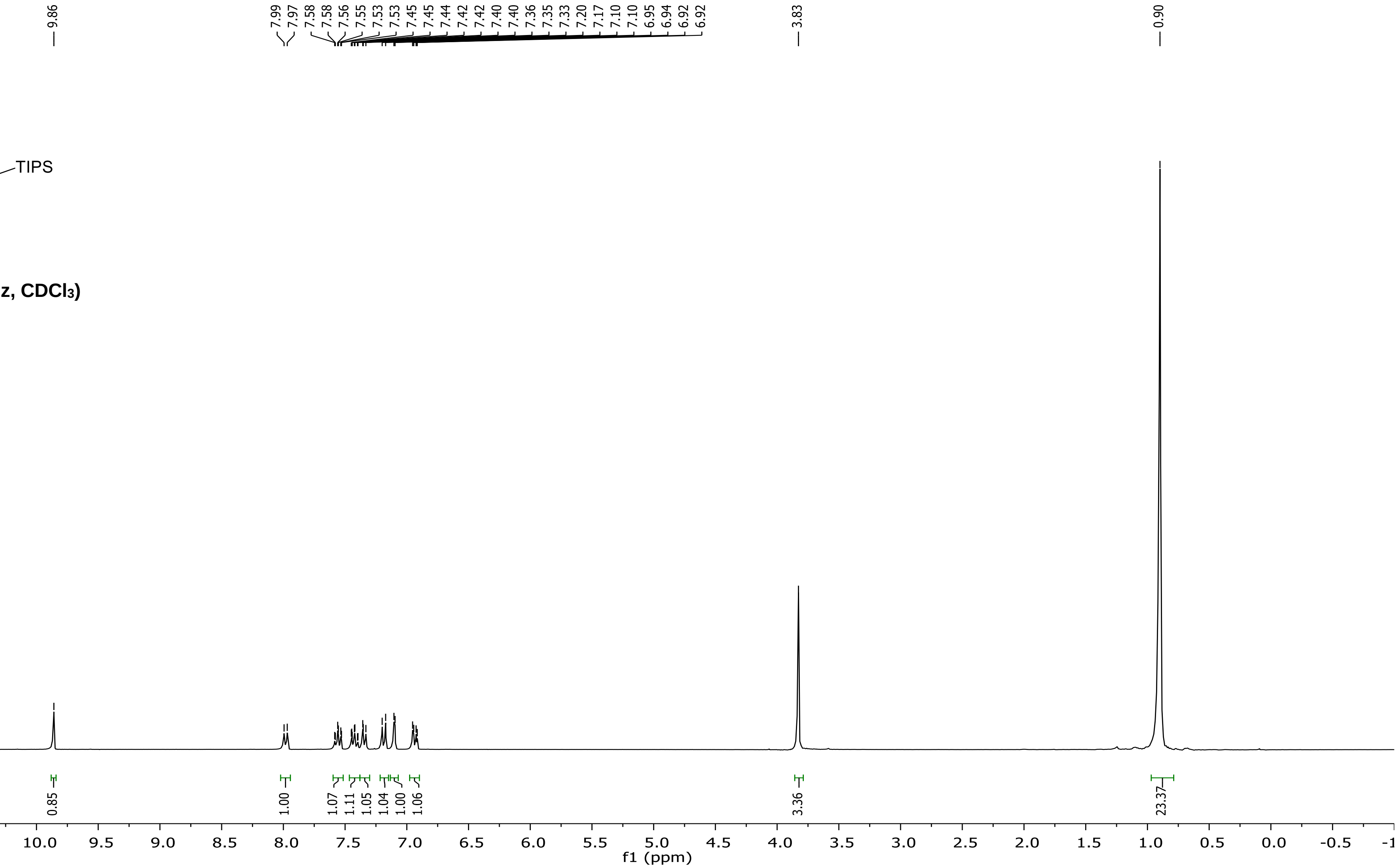

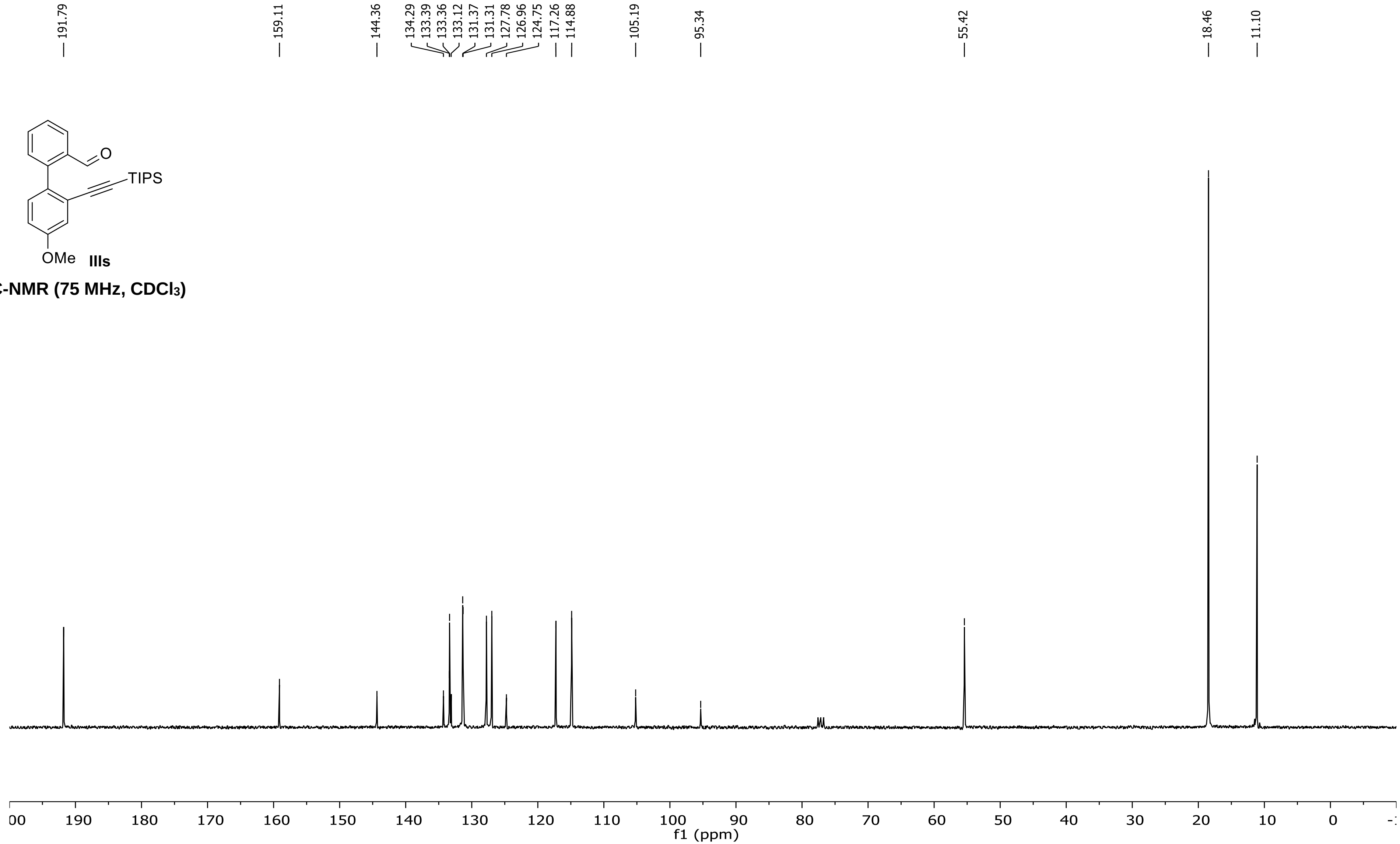

^{13}C-NMR (75 MHz, $CDCl_3$)

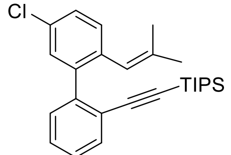

1p-TIPS

^{1}H-NMR (300 MHz, $CDCl_3$)

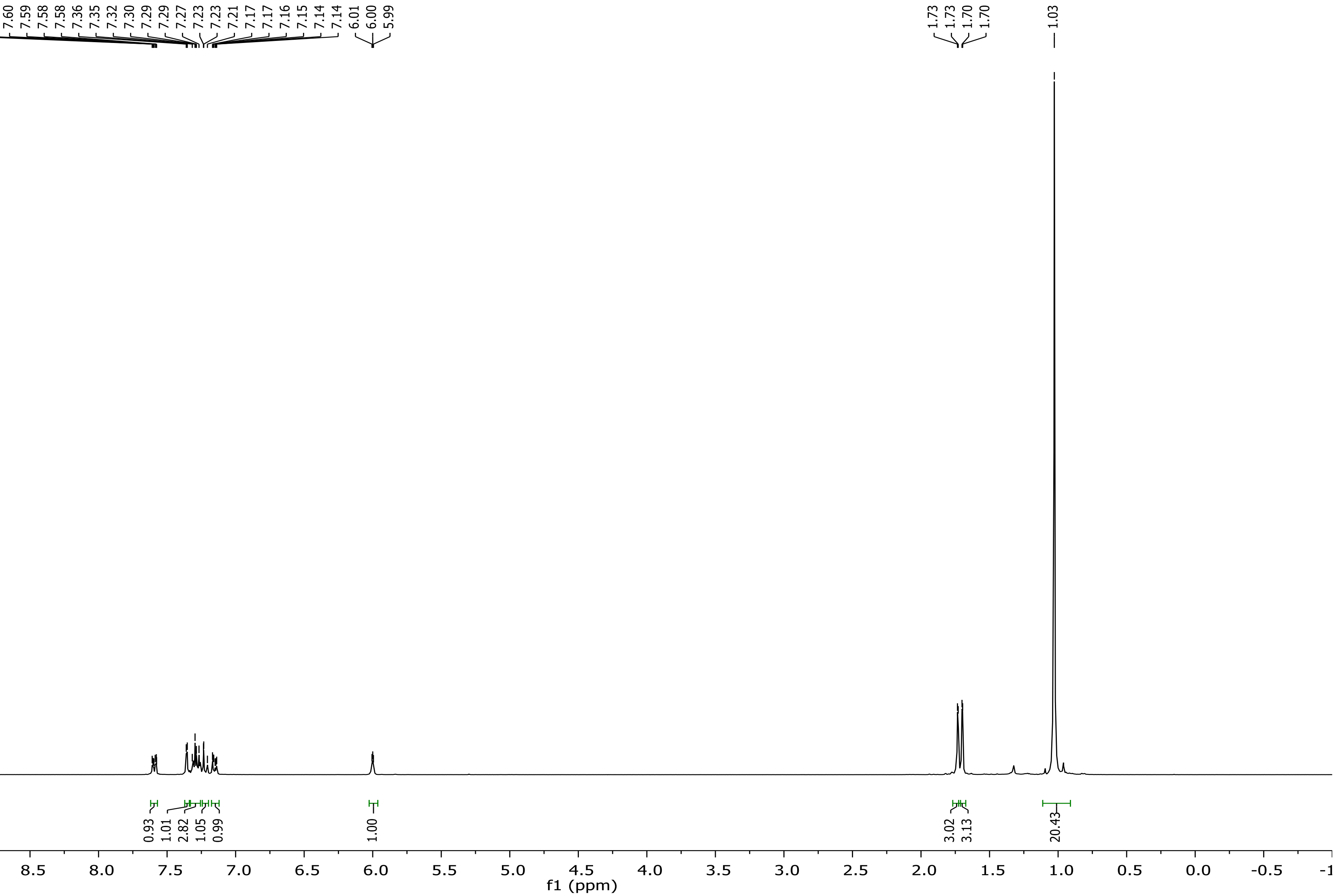

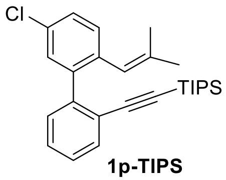

1p-TIPS

^{13}C-NMR (75 MHz, CDCl$_3$)

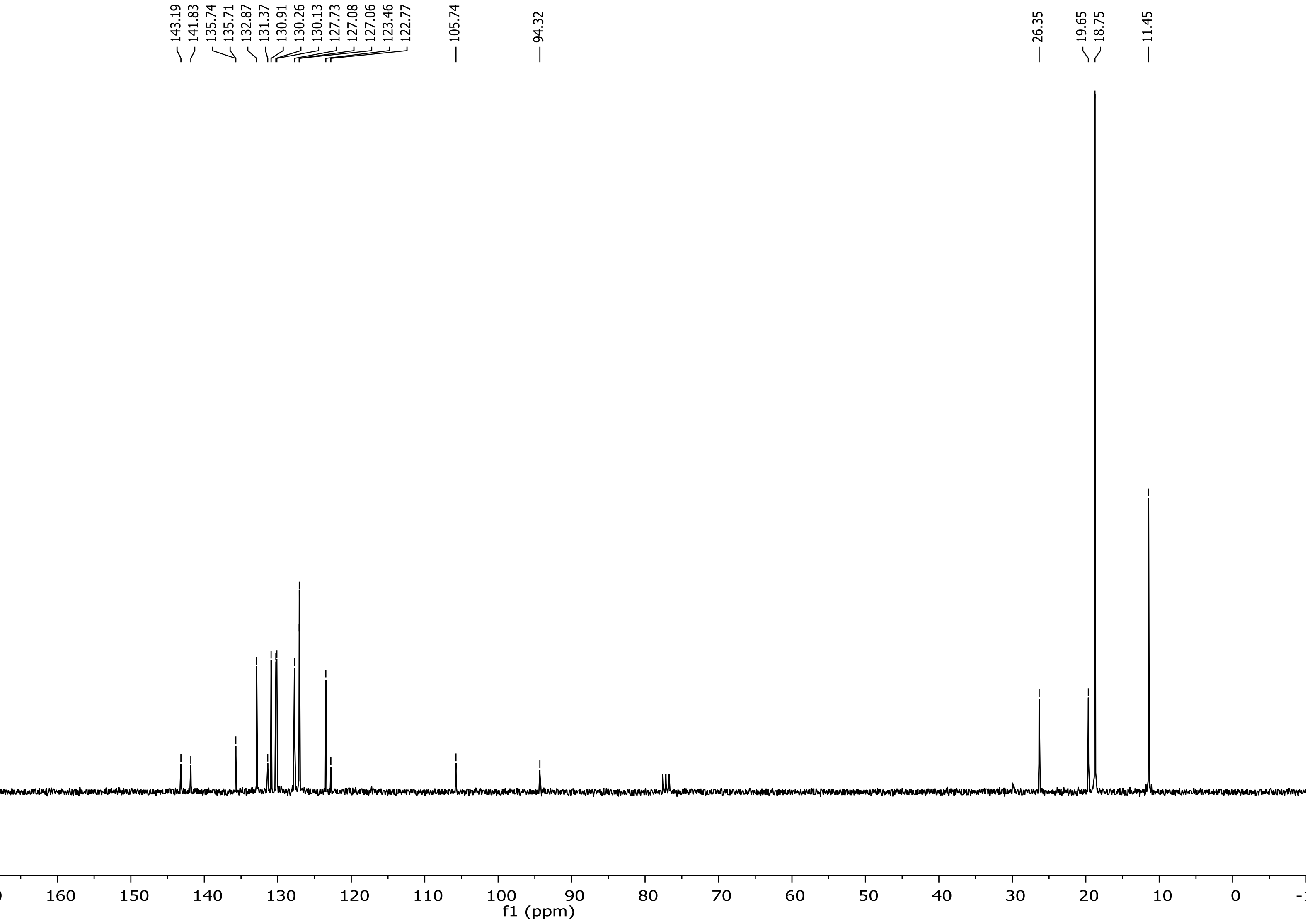

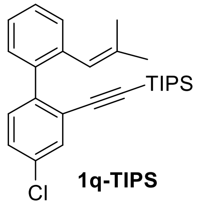

1q-TIPS

^{1}H-NMR (300 MHz, $CDCl_3$)

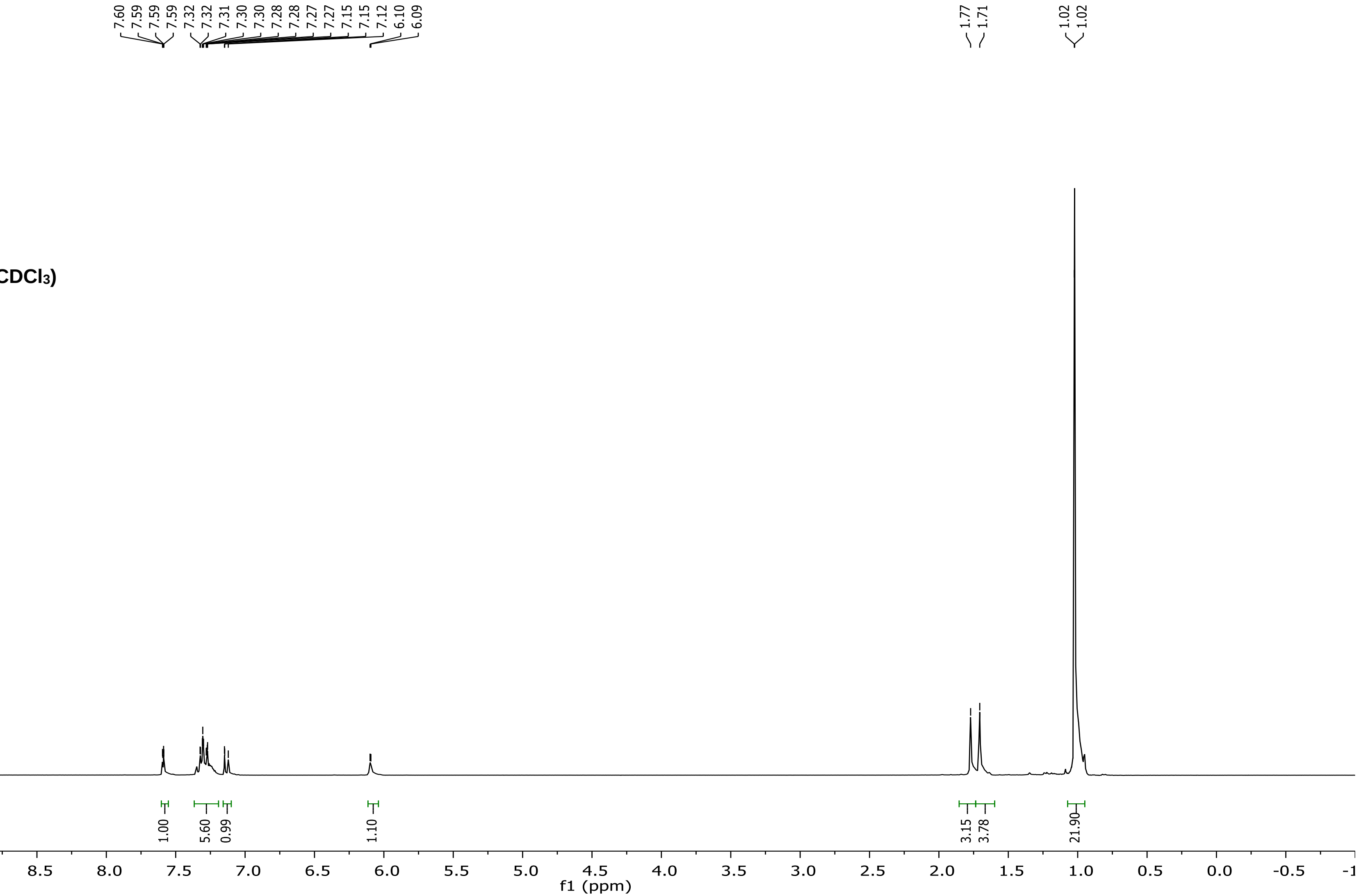

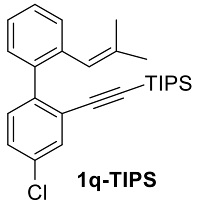

1q-TIPS

^{13}C-NMR (75 MHz, $CDCl_3$)

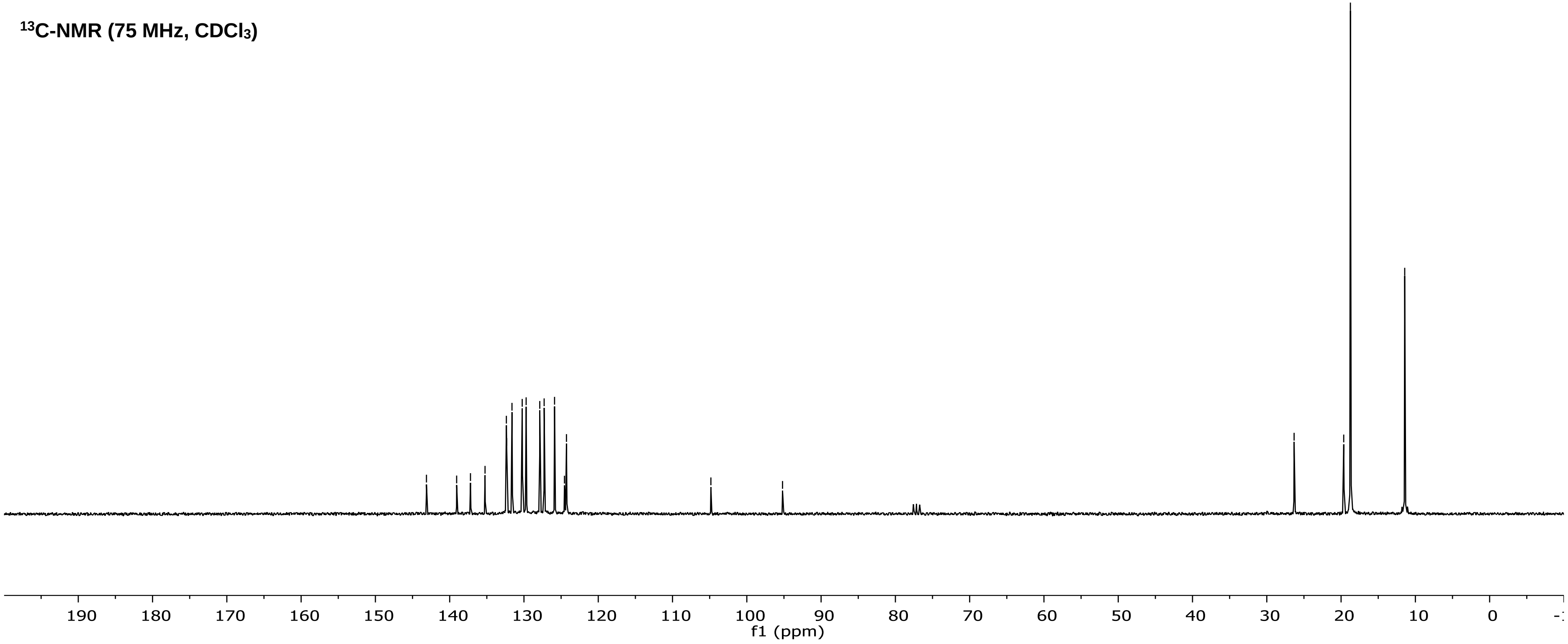

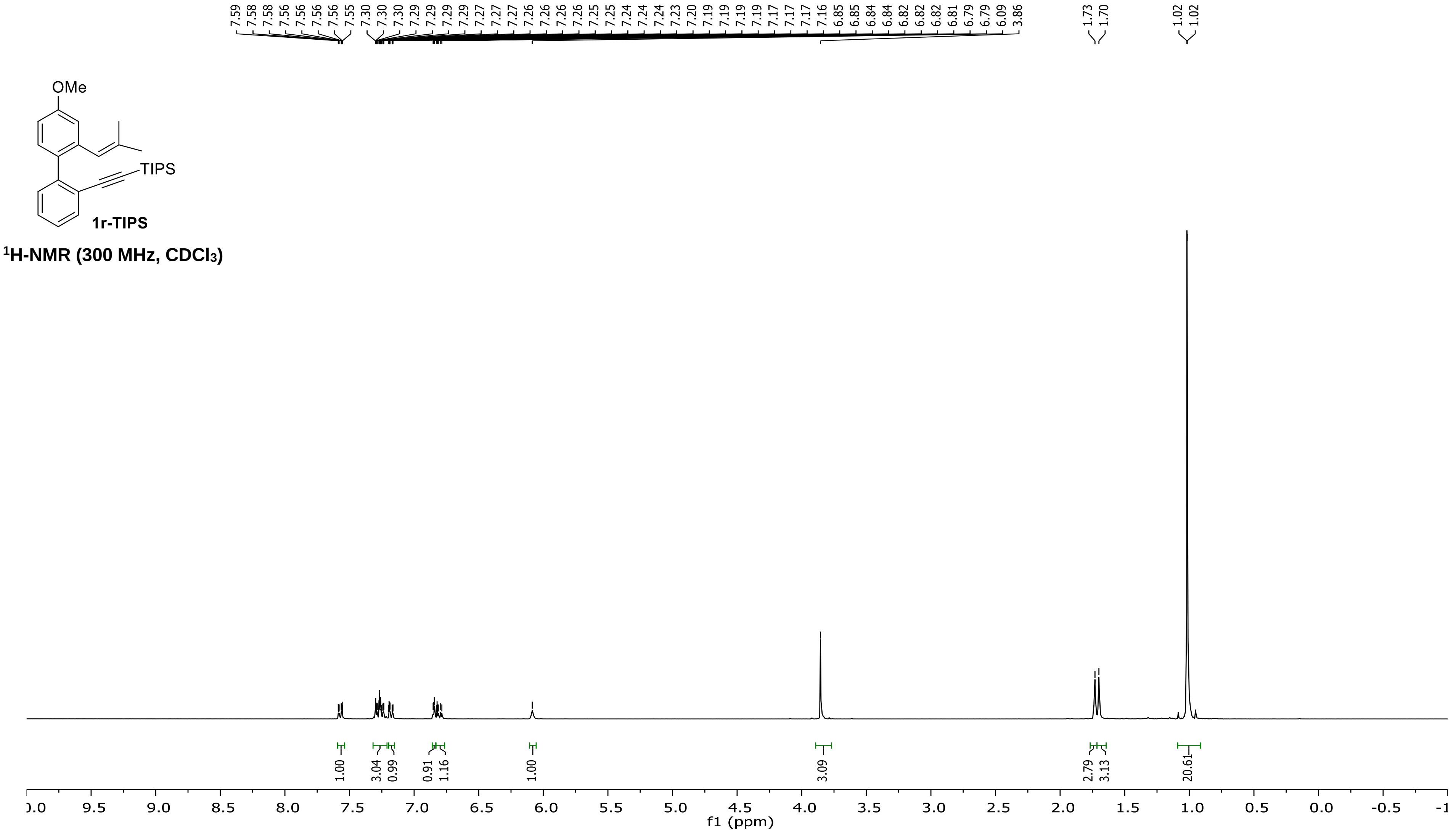

^{1}H-NMR (300 MHz, CDCl$_3$)

1r-TIPS

^{13}C-NMR (75 MHz, CDCl$_3$)

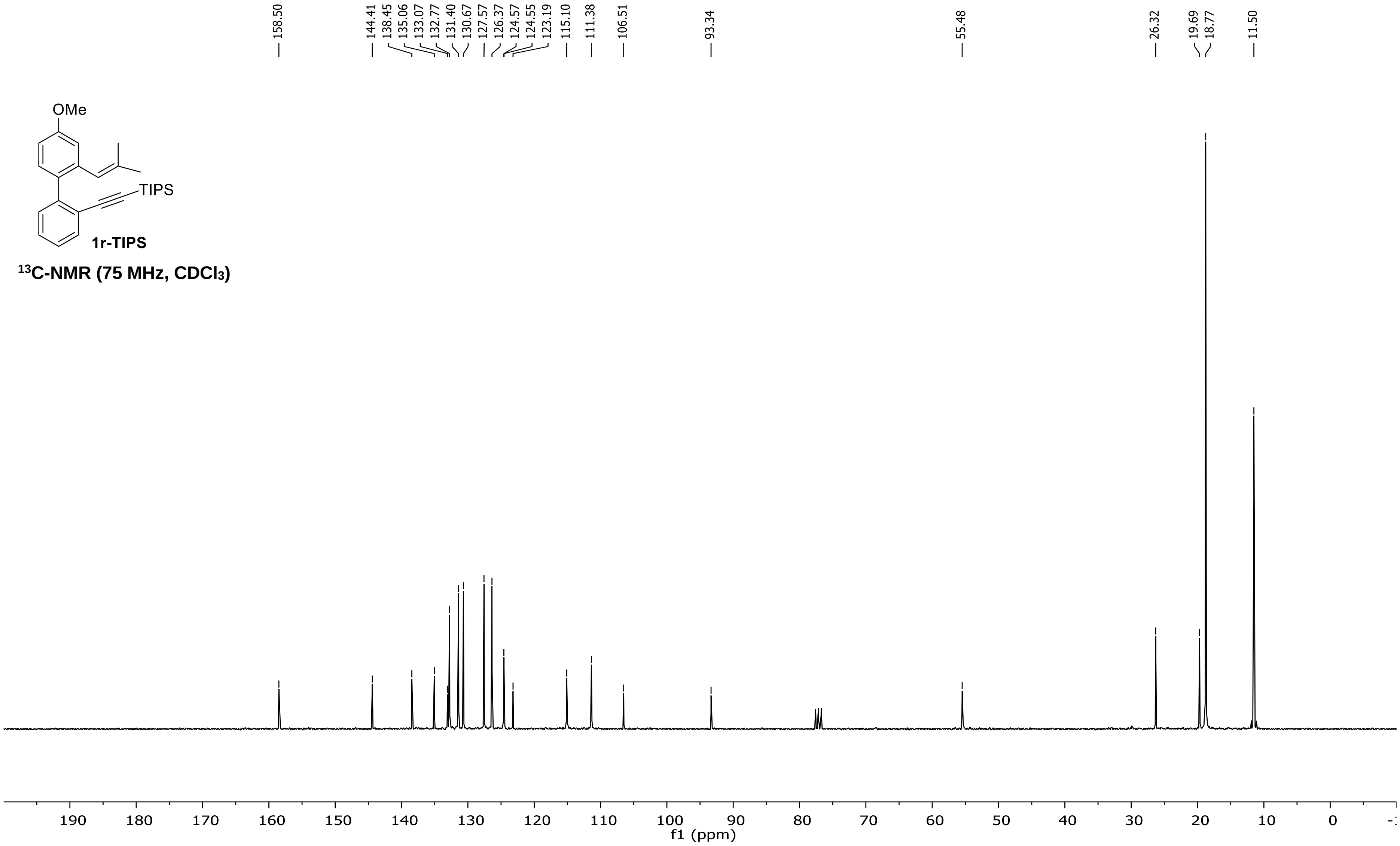

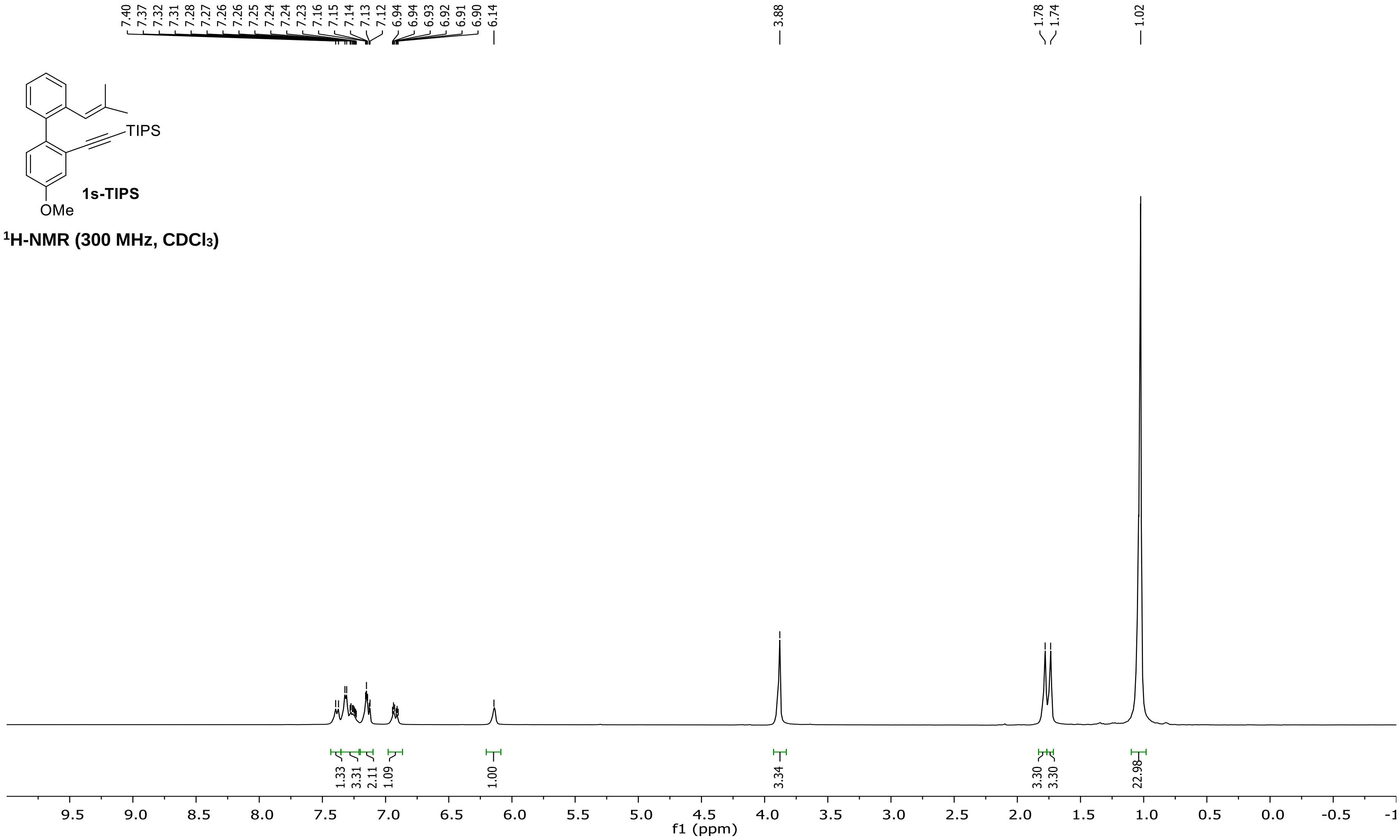
7.40 7.37 7.32 7.31 7.28 7.27 7.26 7.26 7.25 7.24 7.24 7.23 7.16 7.15 7.14 7.13 7.12 6.94 6.94 6.93 6.92 6.91 6.90 6.14
3.88
1.78 1.74
1.02
TIPS
OMe
1s-TIPS
1H-NMR (300 MHz, CDCl3)
1.33 3.31 2.11 1.09
1.00
3.34
3.30 3.30
22.98
9.5 9.0 8.5 8.0 7.5 7.0 6.5 6.0 5.5 5.0 4.5 4.0 3.5 3.0 2.5 2.0 1.5 1.0 0.5 0.0 -0.5 -1
f1 (ppm)

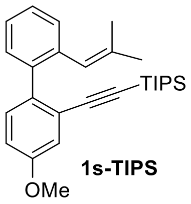

1s-TIPS

^{13}C-NMR (75 MHz, CDCl3)

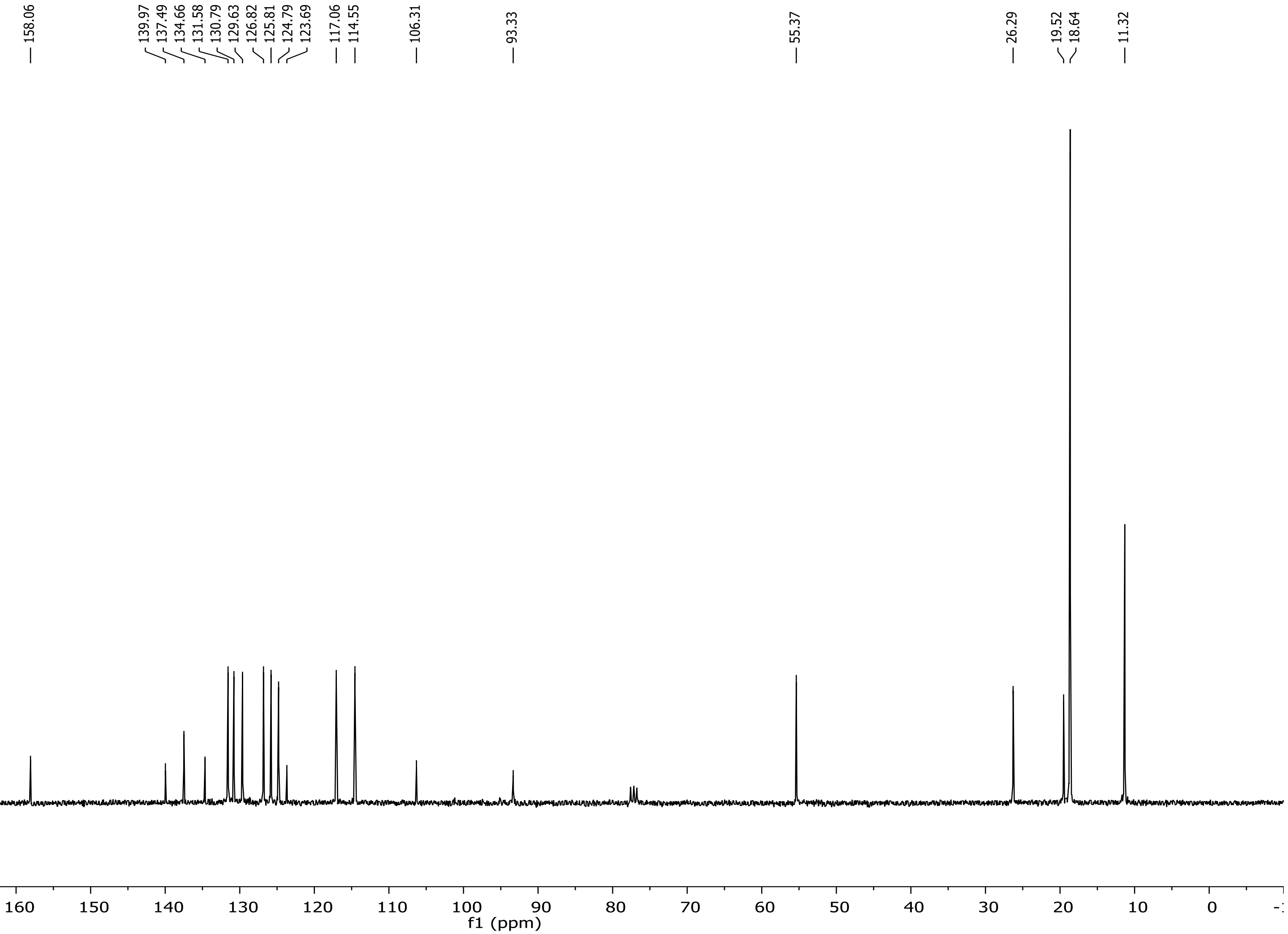

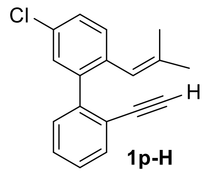

1p-H

^{1}H-NMR (300 MHz, CDCl$_3$)

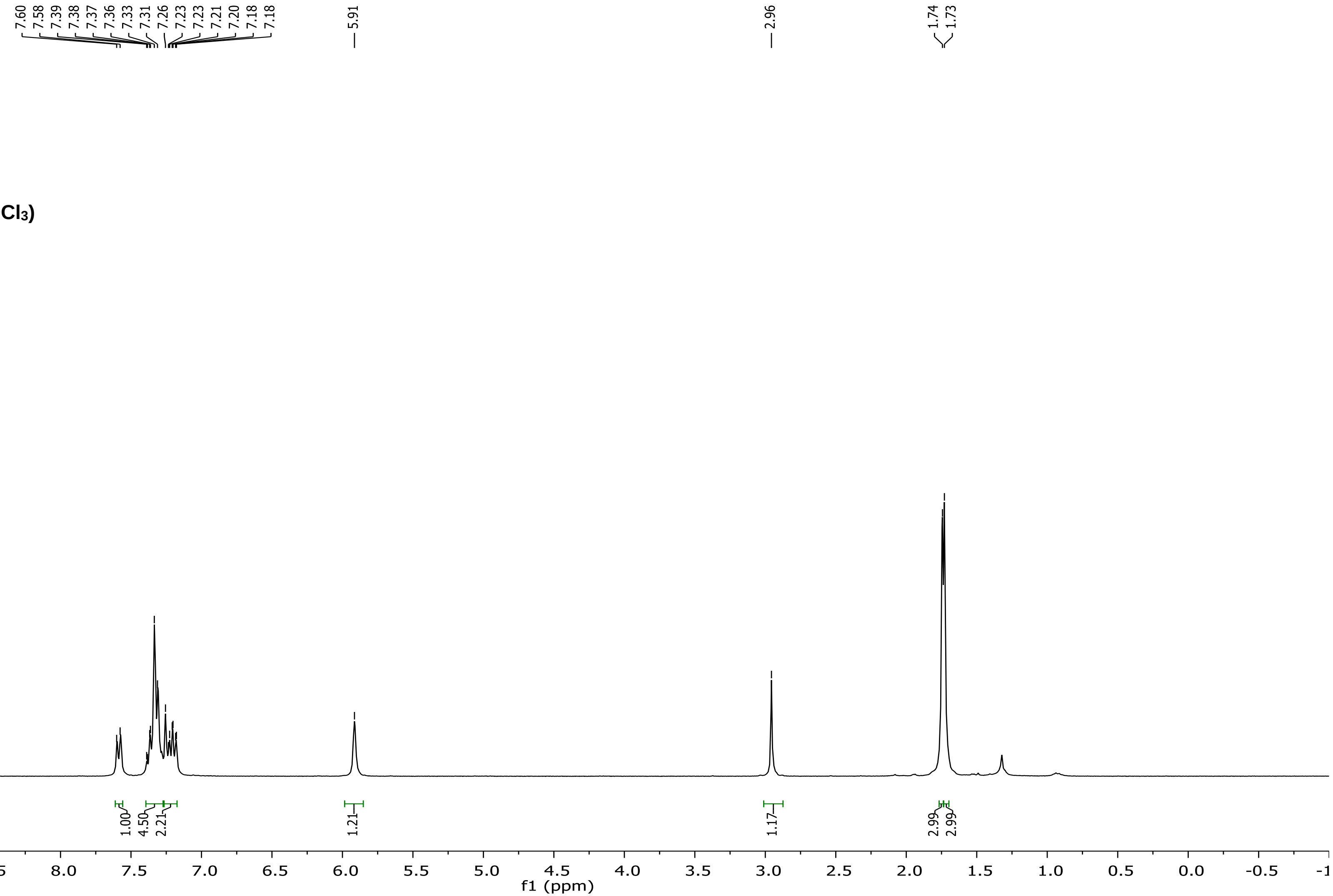

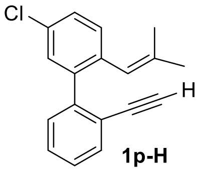

1p-H

^{13}C-NMR (75 MHz, $CDCl_3$)

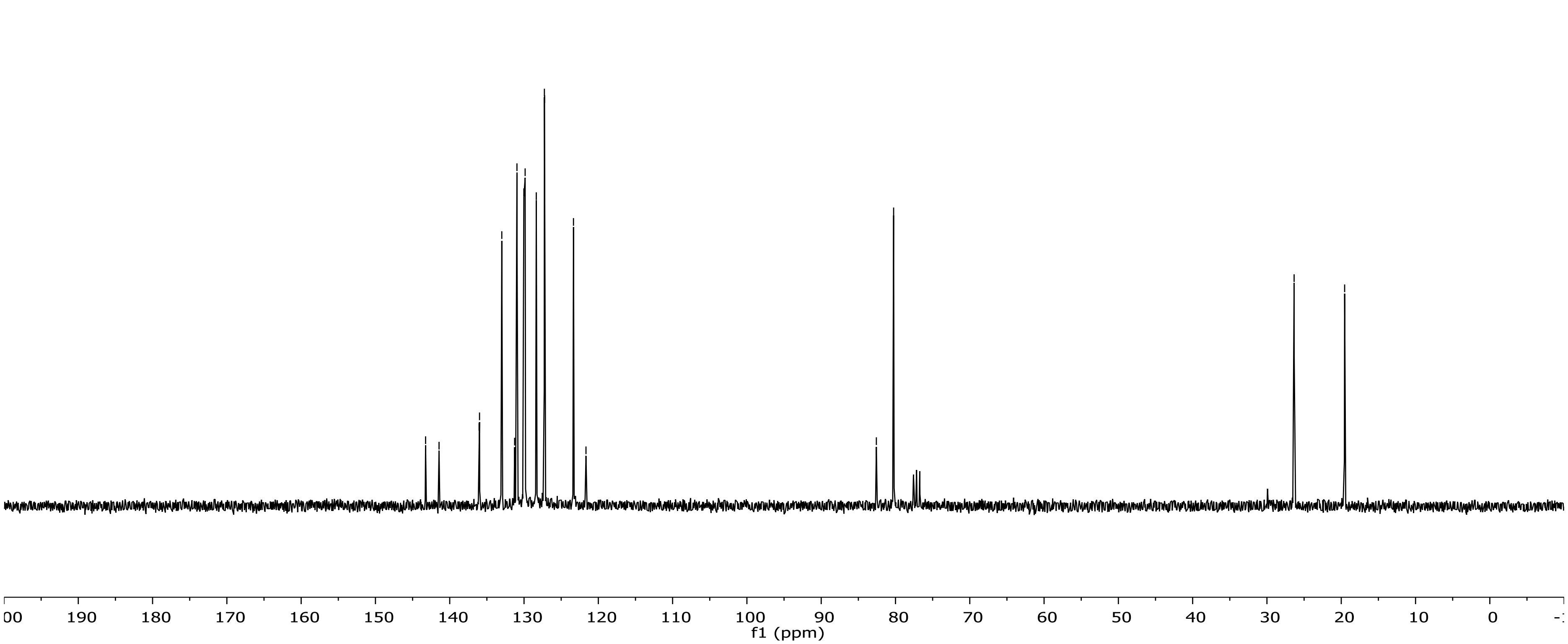

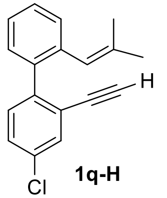

1q-H

^{1}H-NMR (300 MHz, $CDCl_3$)

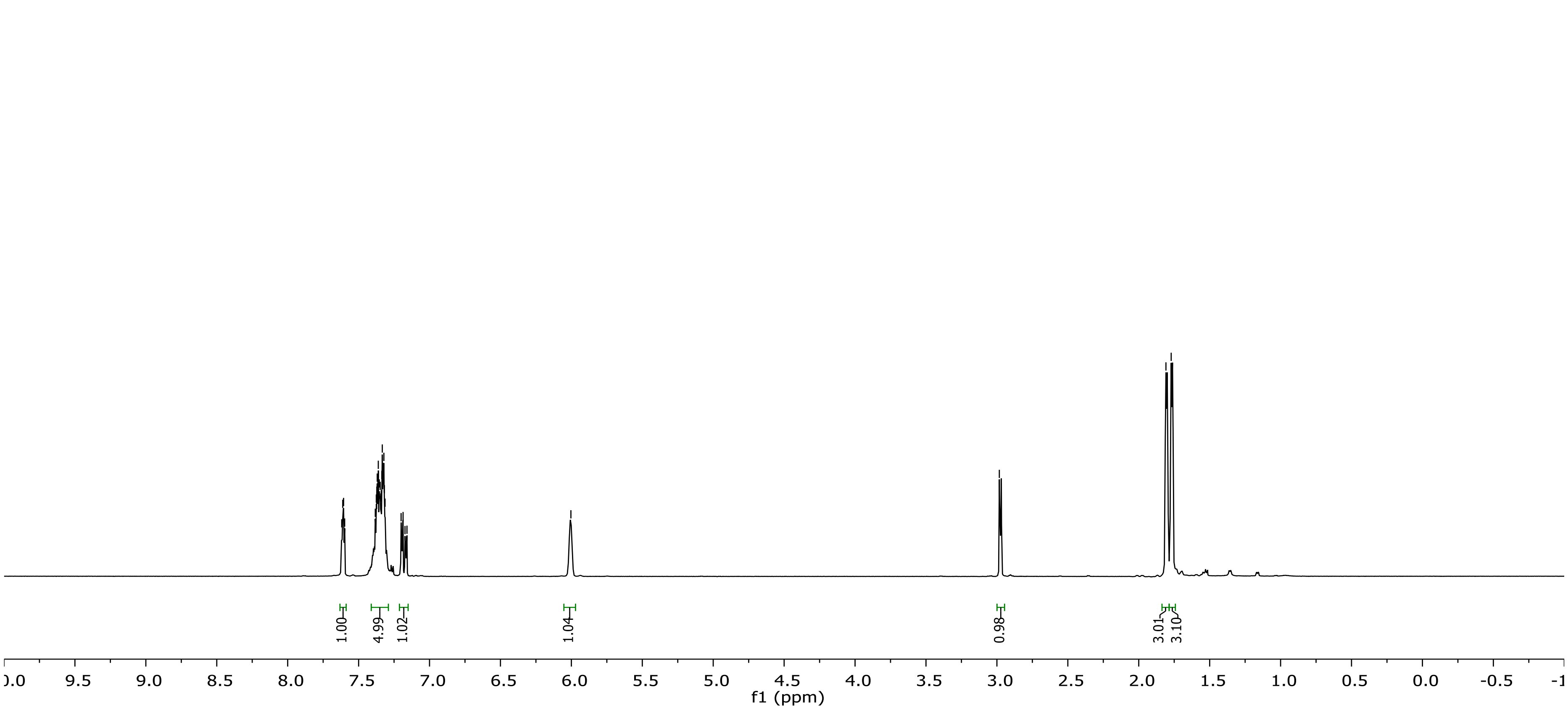

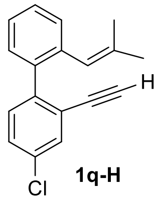

1q-H

^{13}C-NMR (75 MHz, $CDCl_3$)

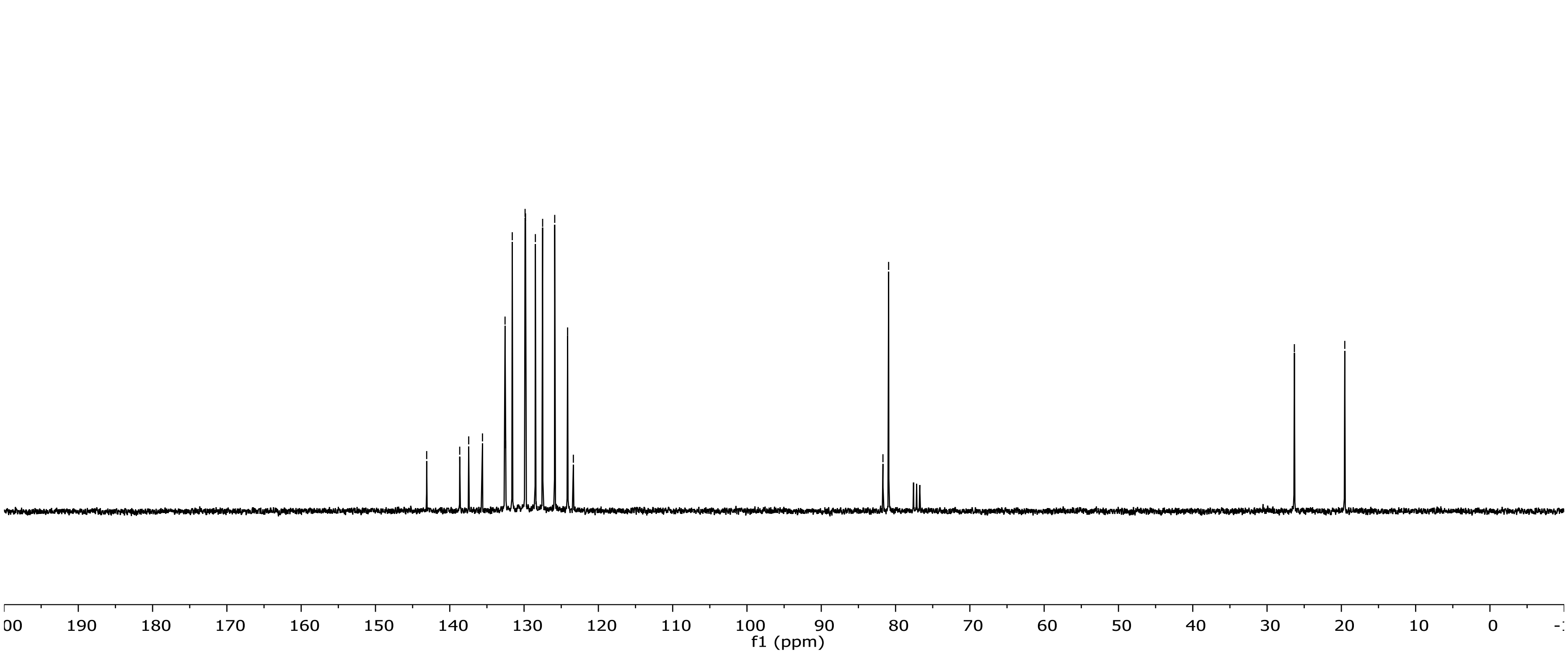

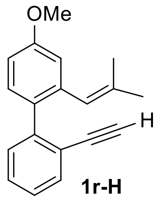

1r-H

^{1}H-NMR (300 MHz, CDCl$_3$)

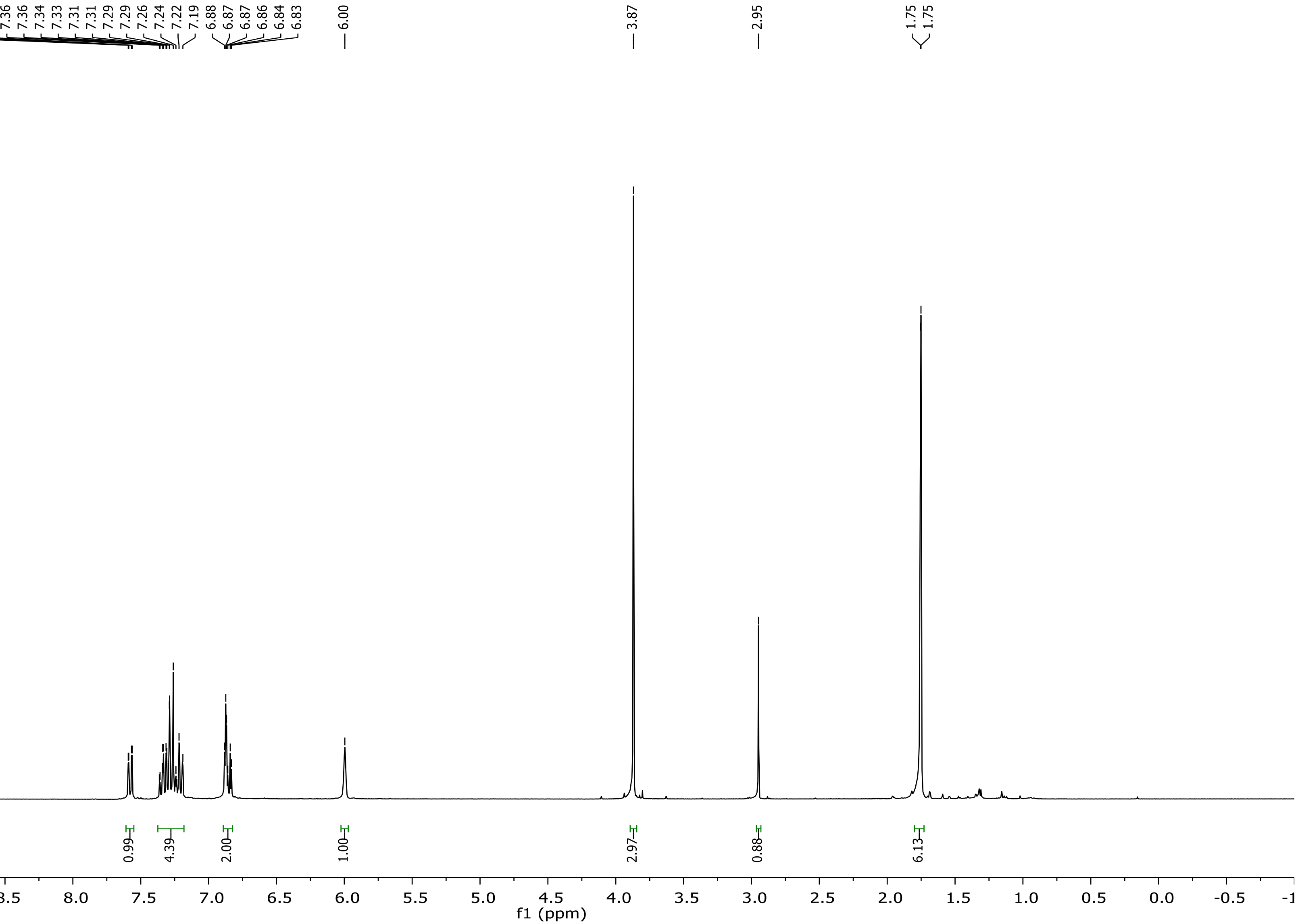

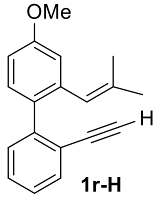

1r-H

^{13}C-NMR (75 MHz, $CDCl_3$)

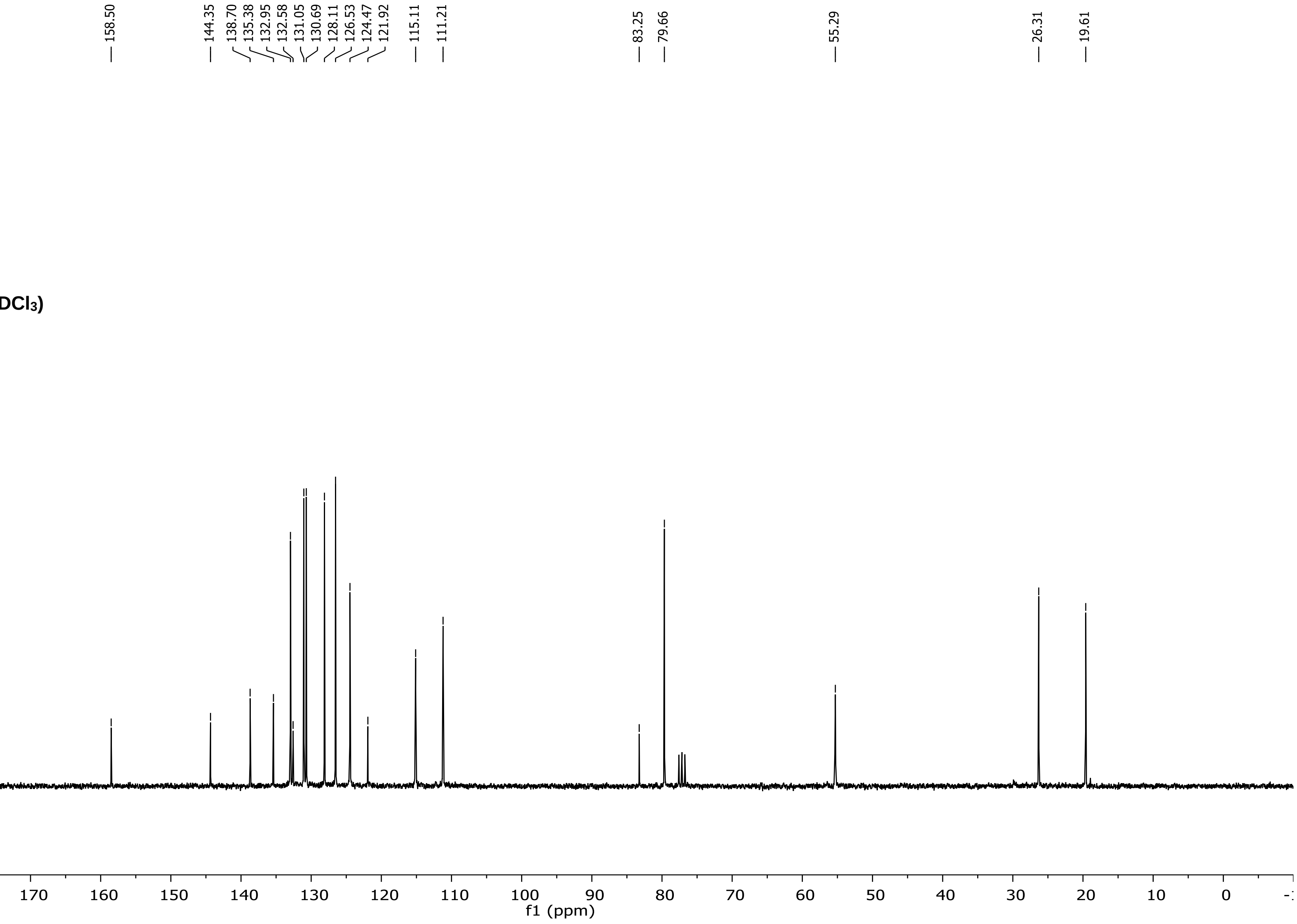

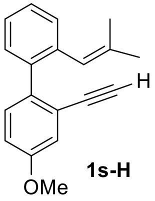

1s-H

^{1}H-NMR (300 MHz, $CDCl_3$)

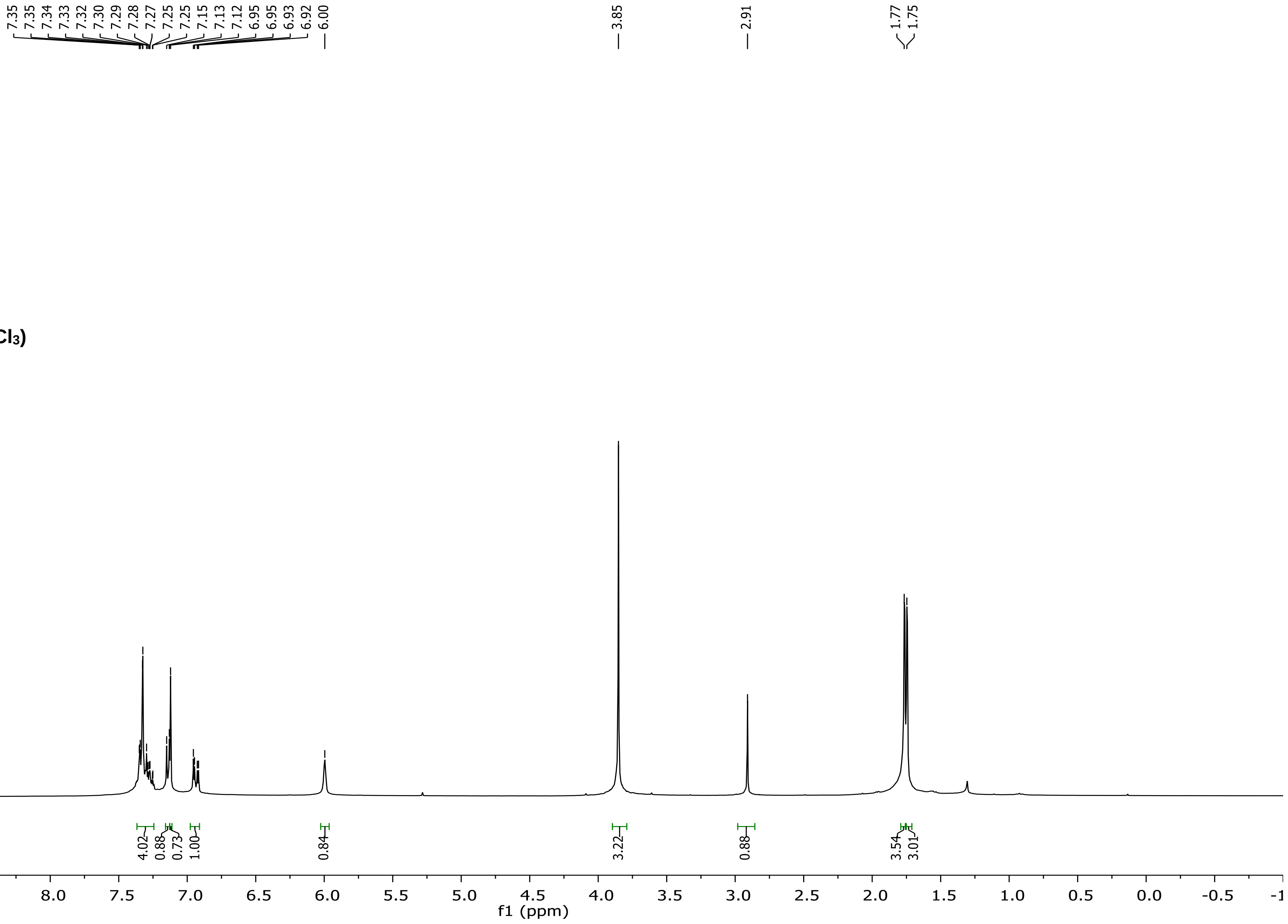

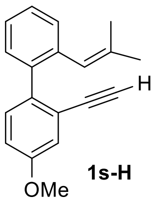

1s-H

^{13}C-NMR (75 MHz, $CDCl_3$)

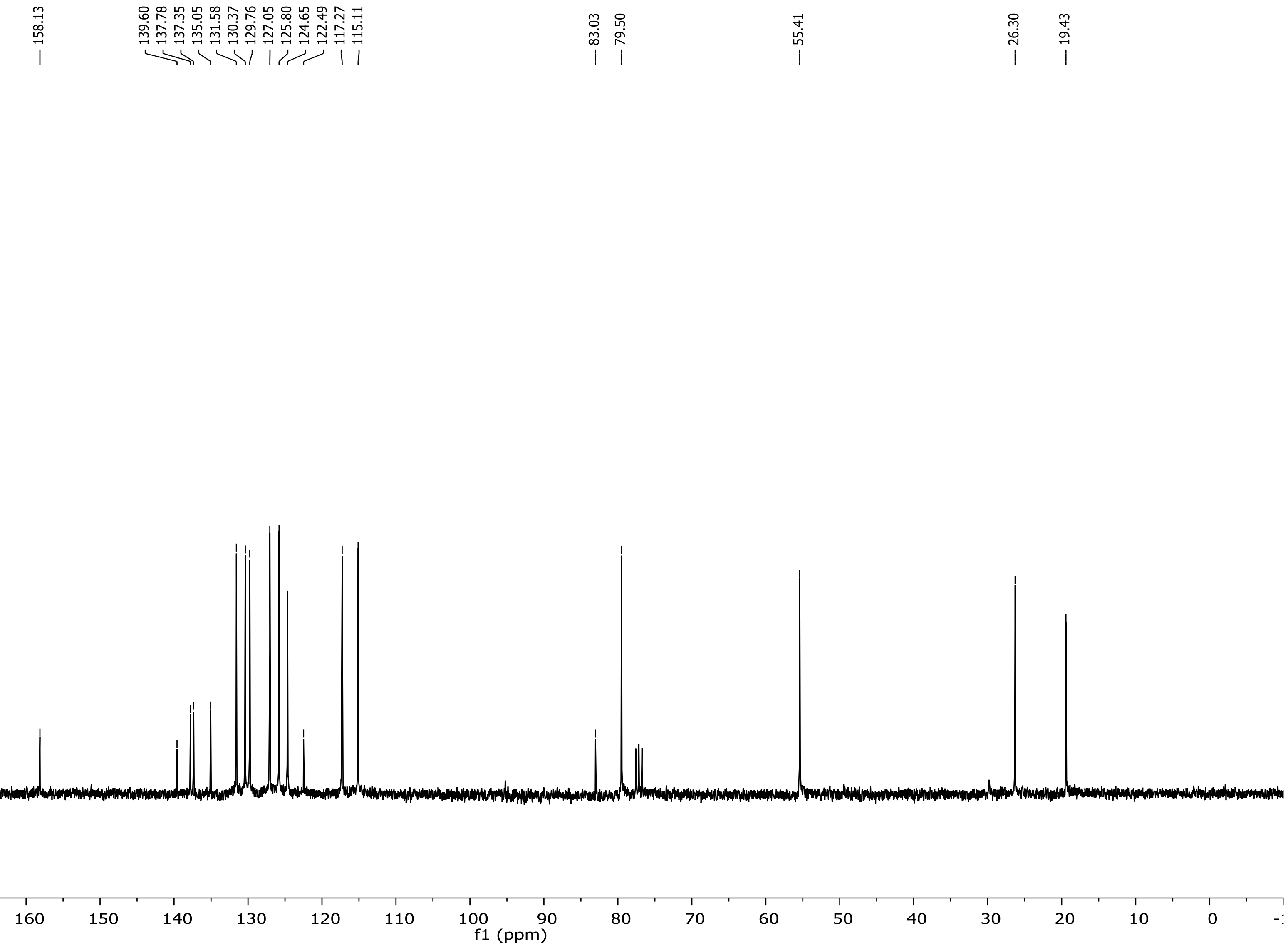

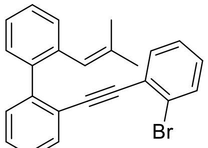

1e

^{1}H-NMR (300 MHz, $CDCl_3$)

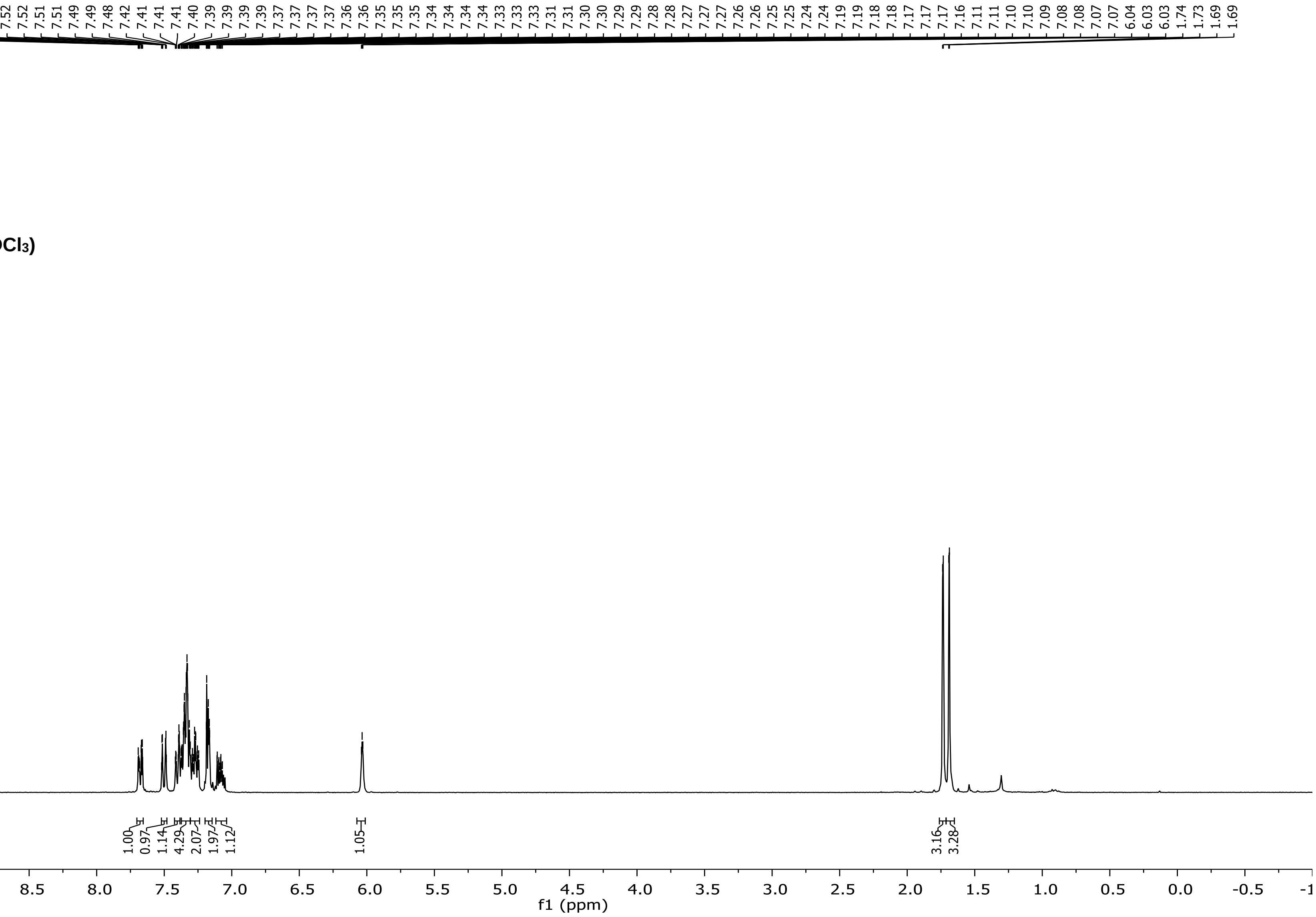

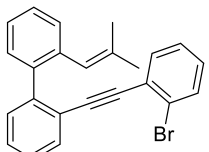

1e

^{13}C-NMR (75 MHz, $CDCl_3$)

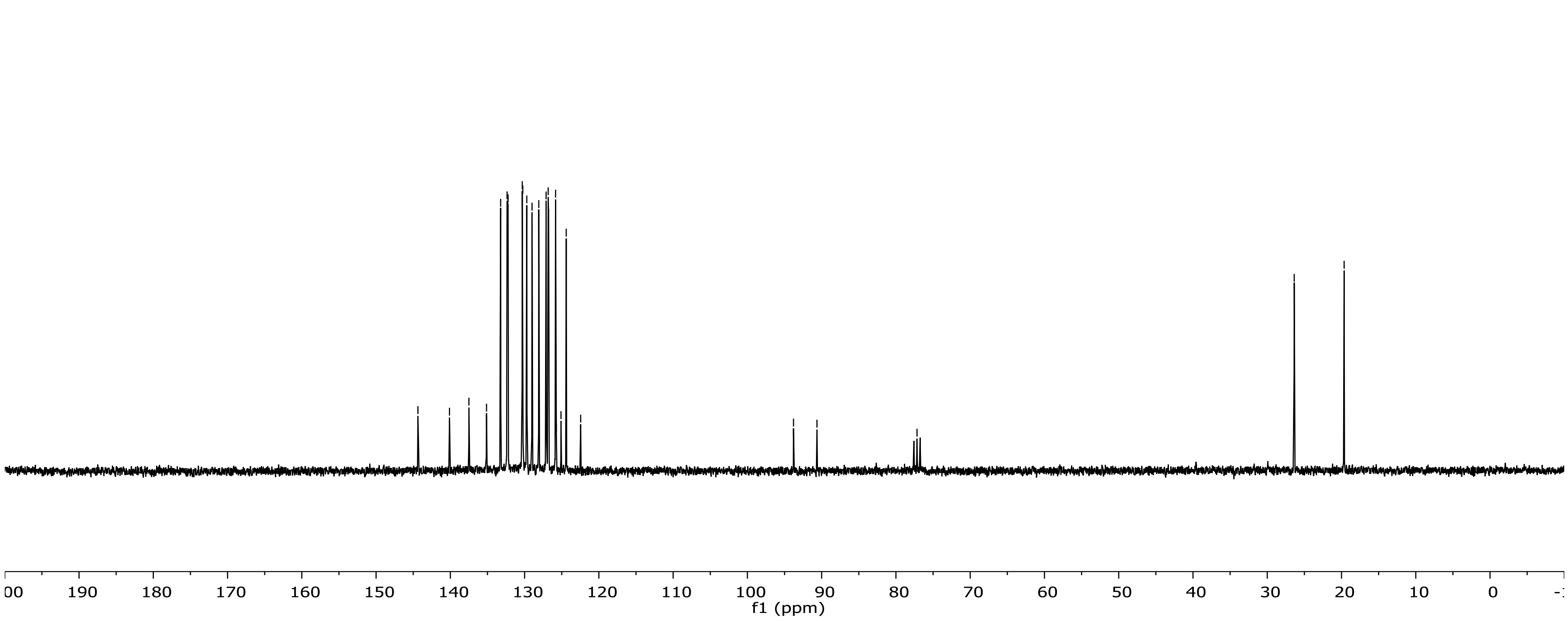

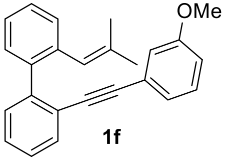

1f

^{1}H-NMR (300 MHz, CDCl$_3$)

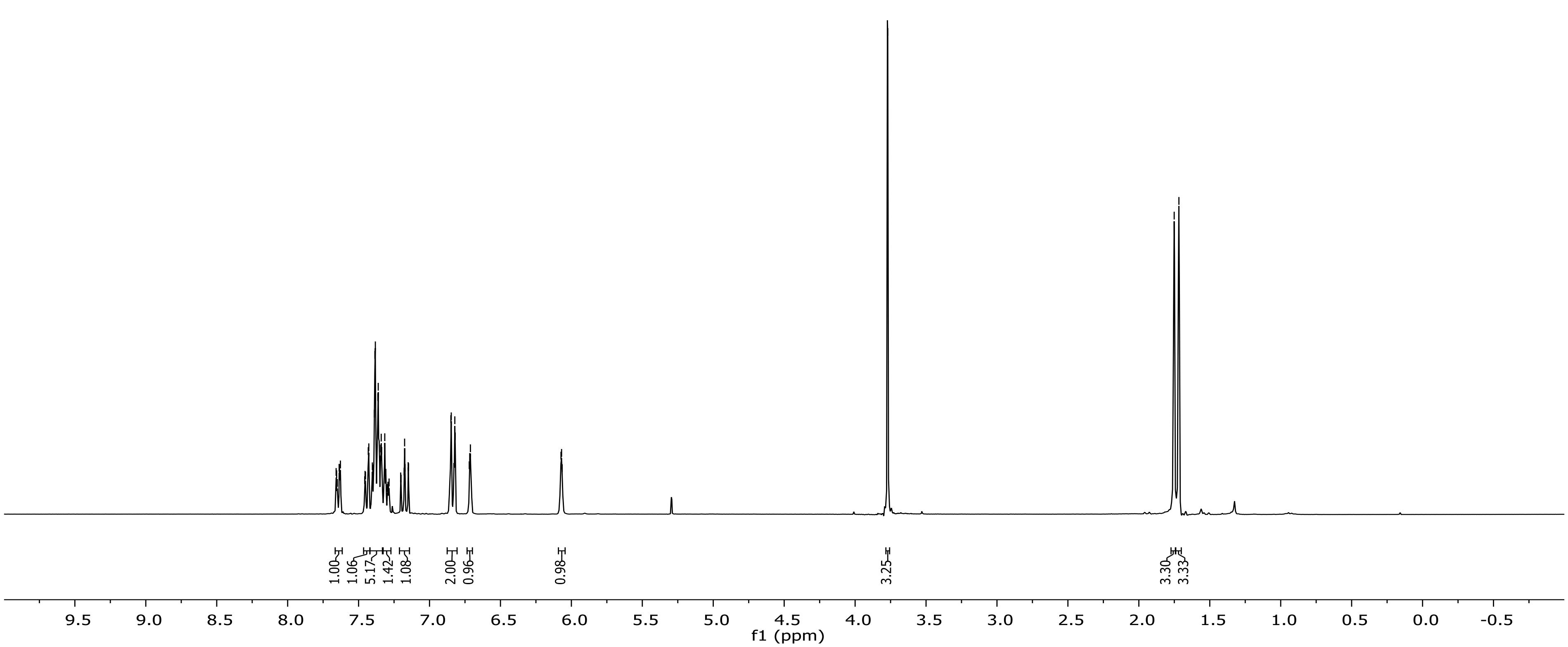

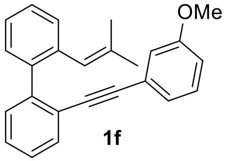

1f

^{13}C-NMR (75 MHz, $CDCl_3$)

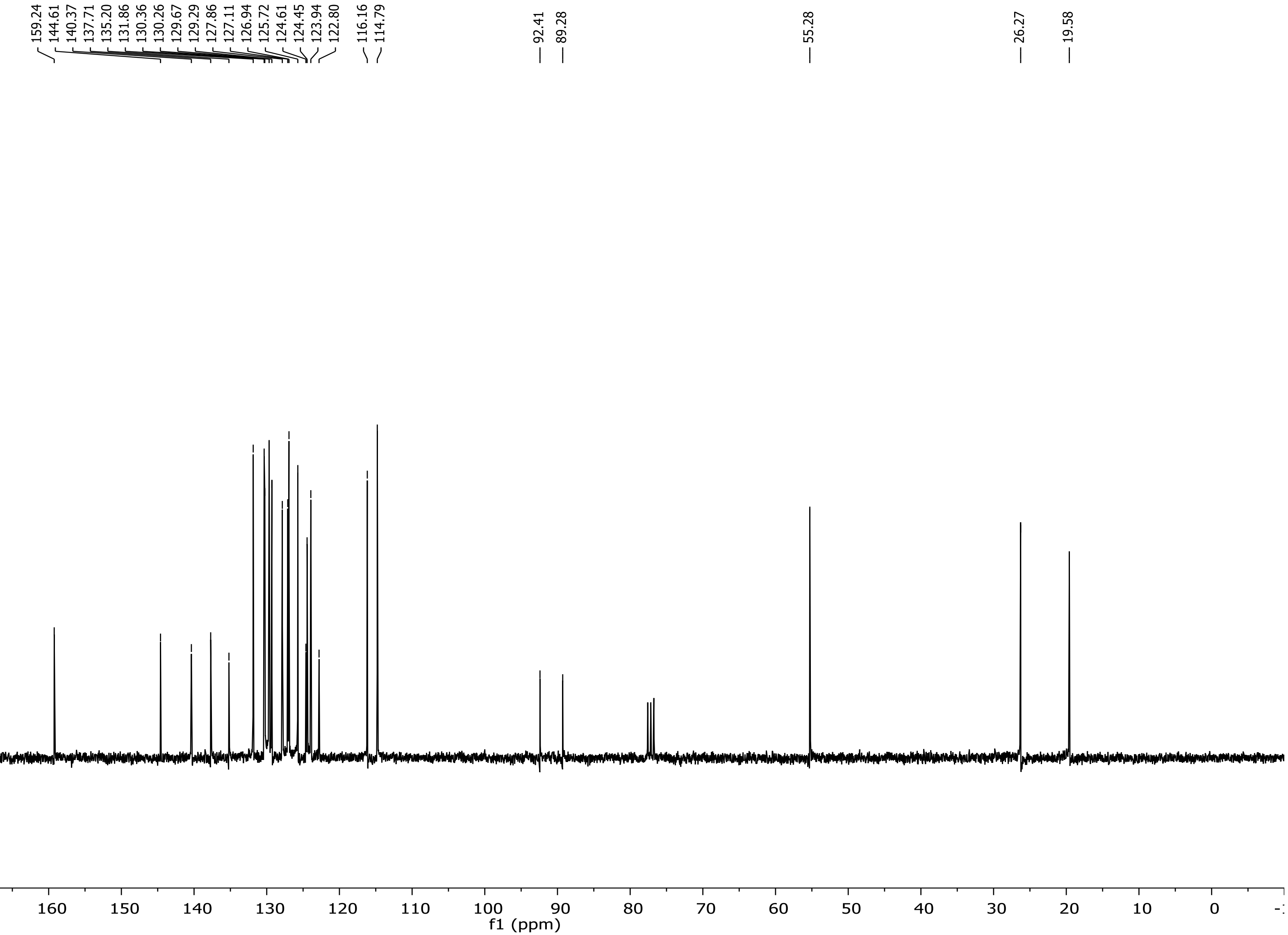

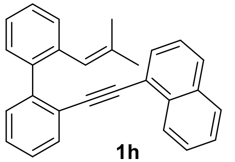

1h

^{1}H-NMR (300 MHz, $CDCl_3$)

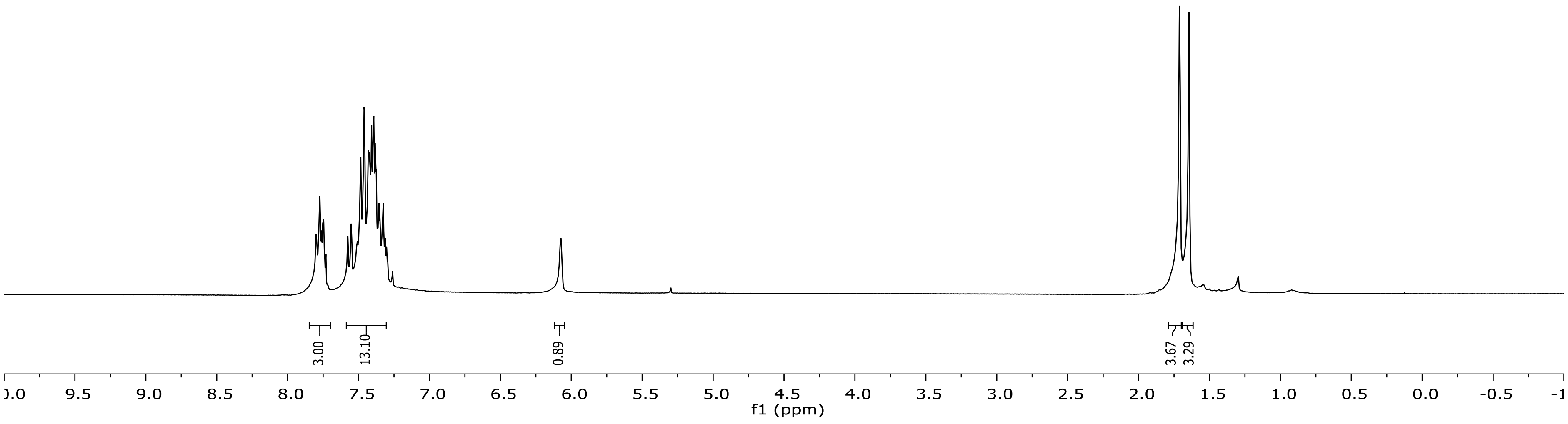

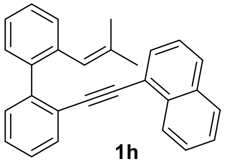

1h

^{13}C-NMR (75 MHz) $CDCl_3$)

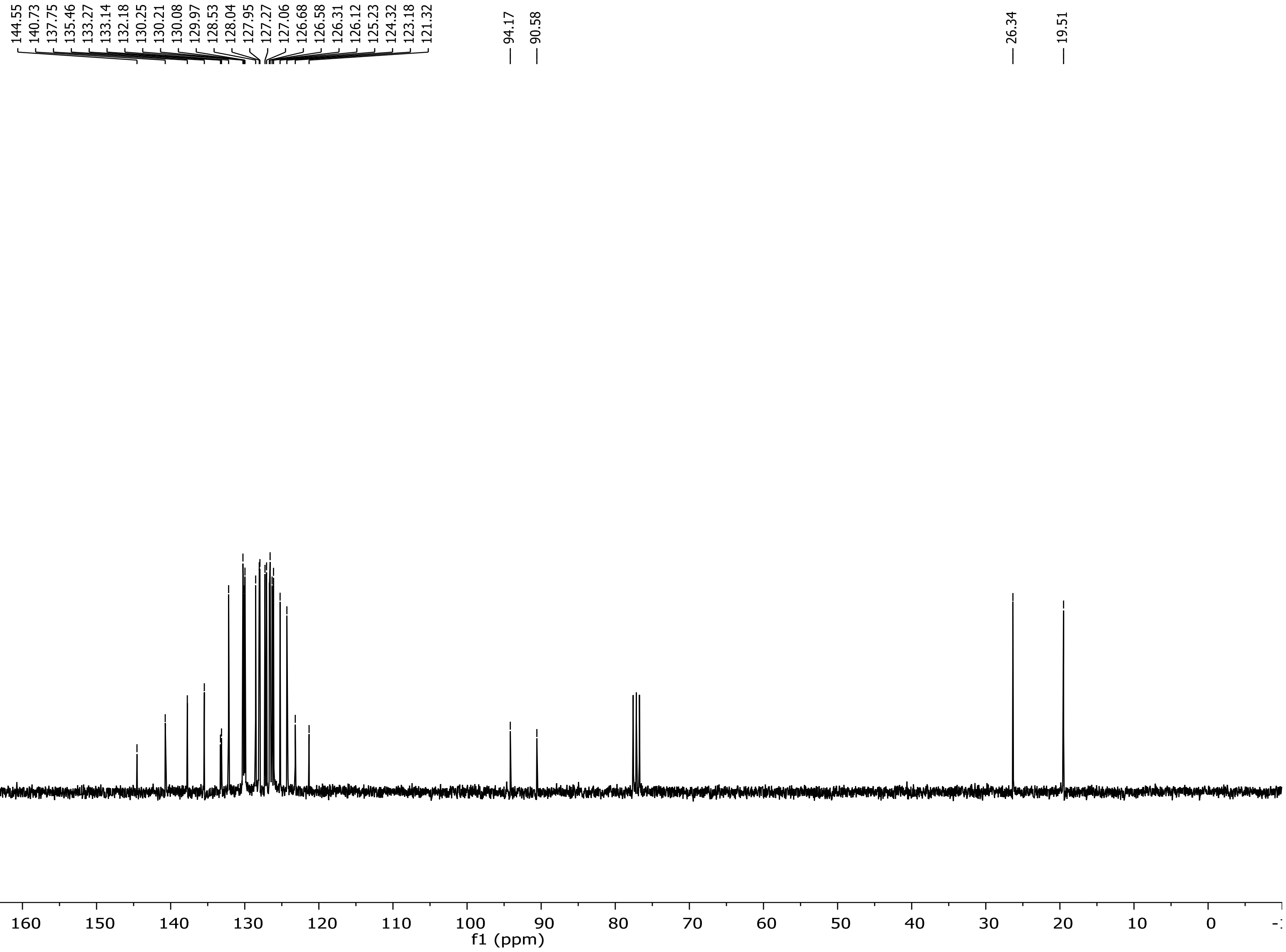

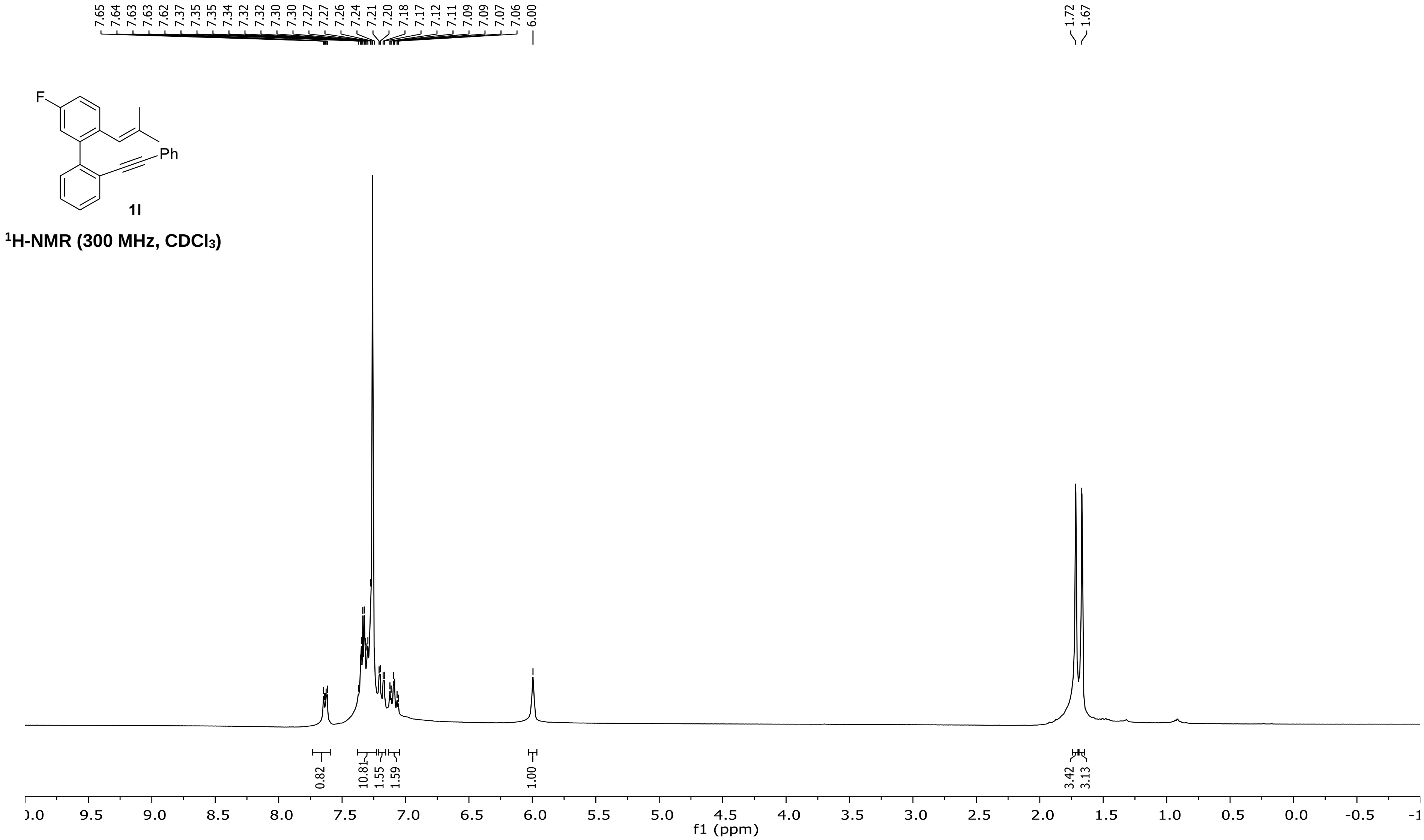

7.65 7.64 7.63 7.63 7.62 7.37 7.35 7.35 7.34 7.32 7.32 7.30 7.30 7.27 7.27 7.26 7.24 7.21 7.20 7.18 7.17 7.12 7.11 7.09 7.09 7.07 7.06
6.00
1.72 1.67
F
Ph
1l
¹H-NMR (300 MHz, CDCl₃)
0.82
10.81
1.55
1.59
1.00
3.42
3.13
f1 (ppm)

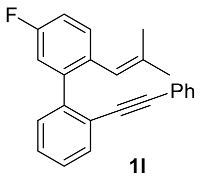

1l

^{13}C-NMR (75 MHz, CDCl$_3$)

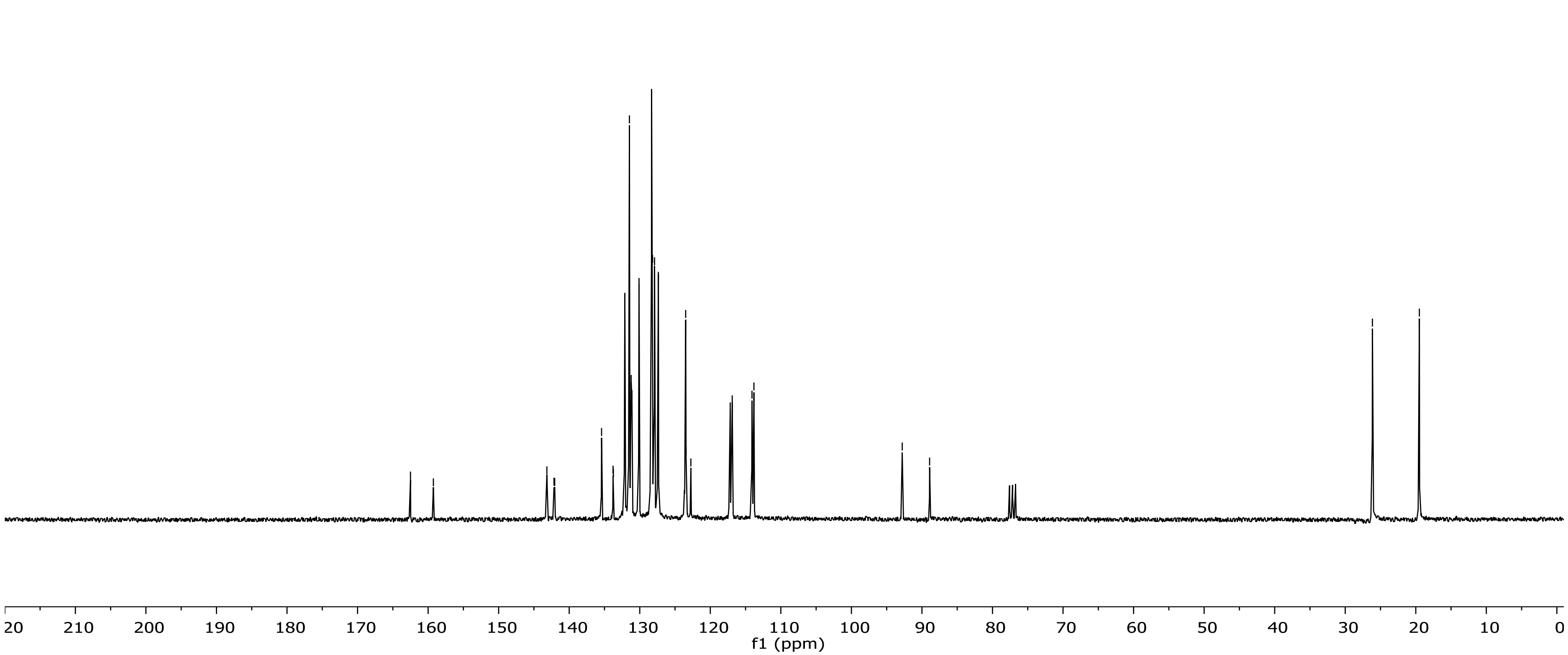

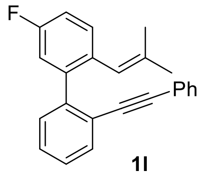

1l

^{19}F-NMR (283 MHz, $CDCl_3$)

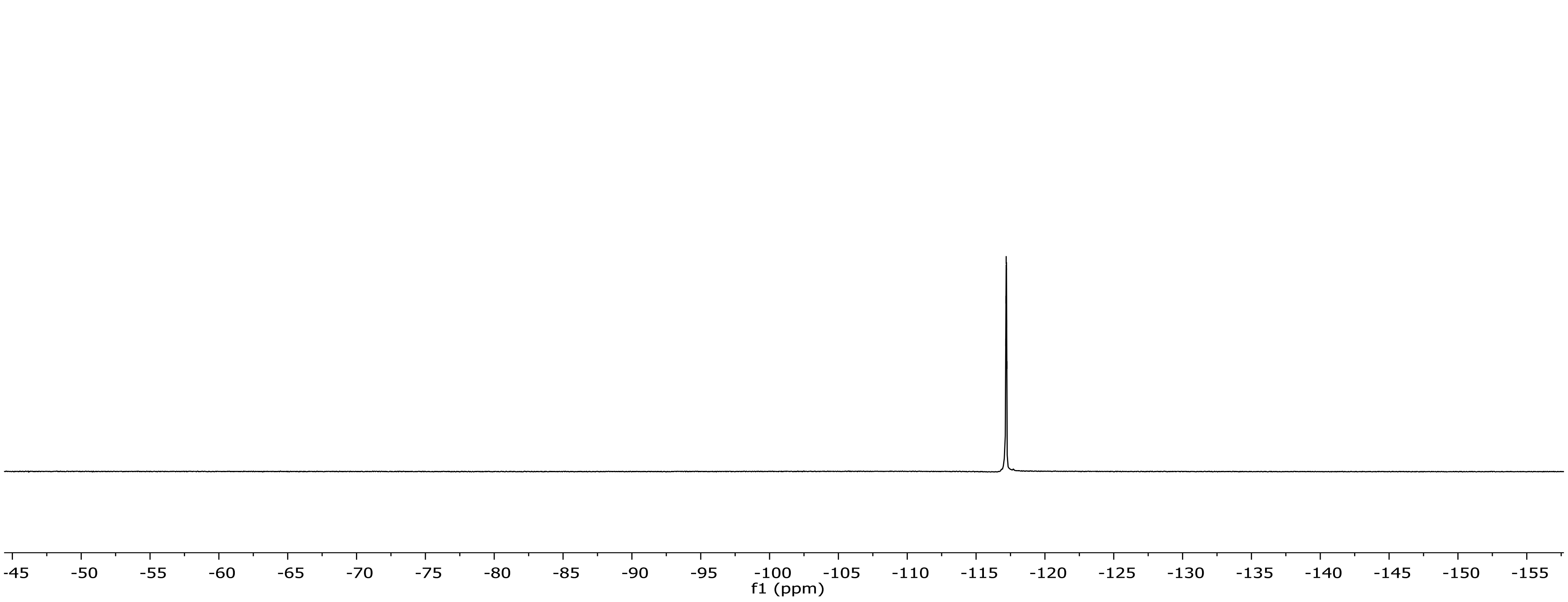

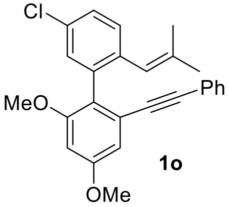

1o

^{1}H-NMR (500 MHz, $CDCl_3$)

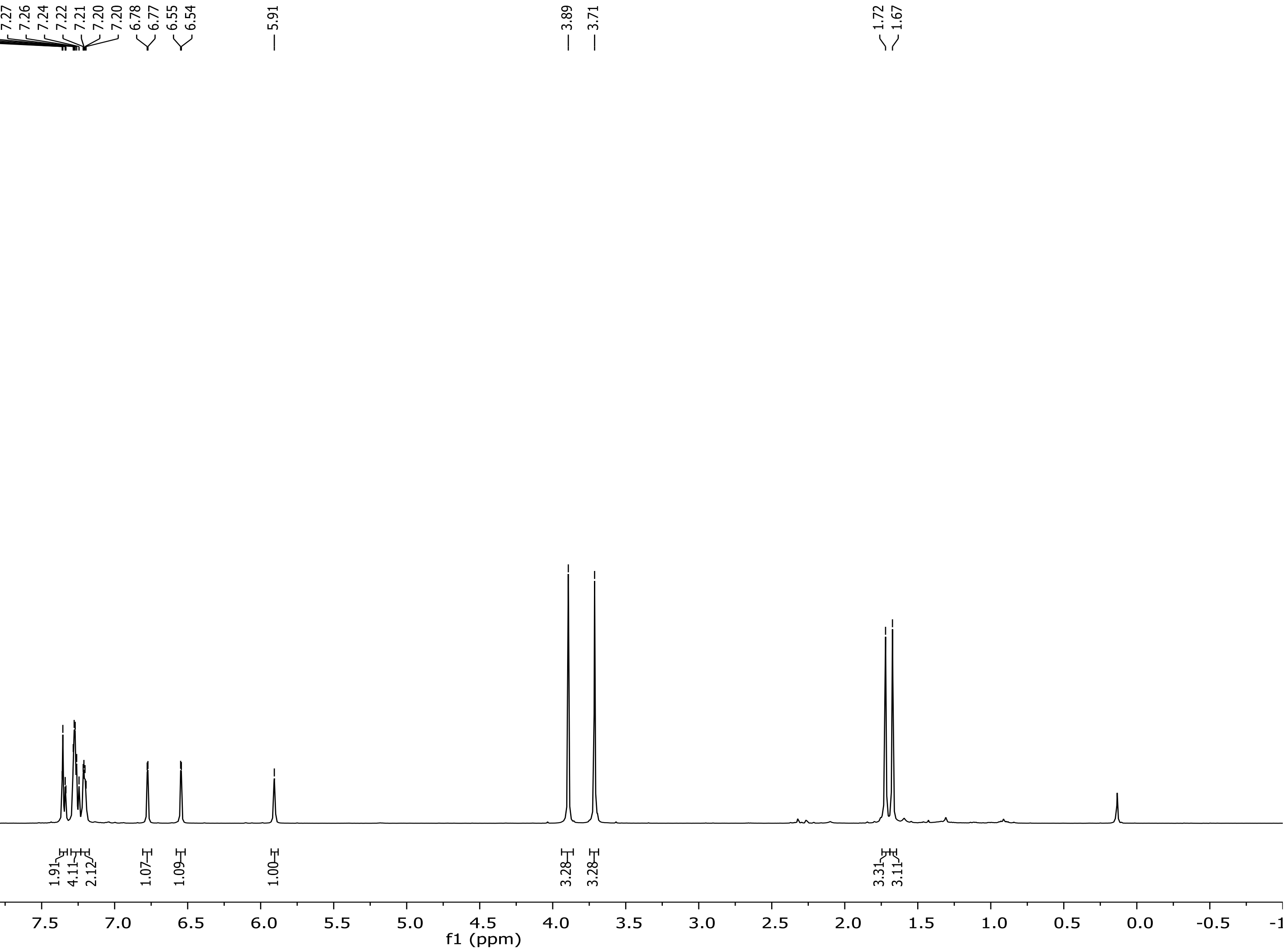

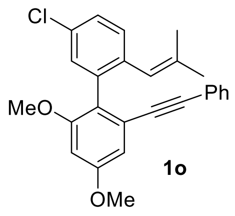

1o

^{13}C-NMR (125 MHz, CDCl3)

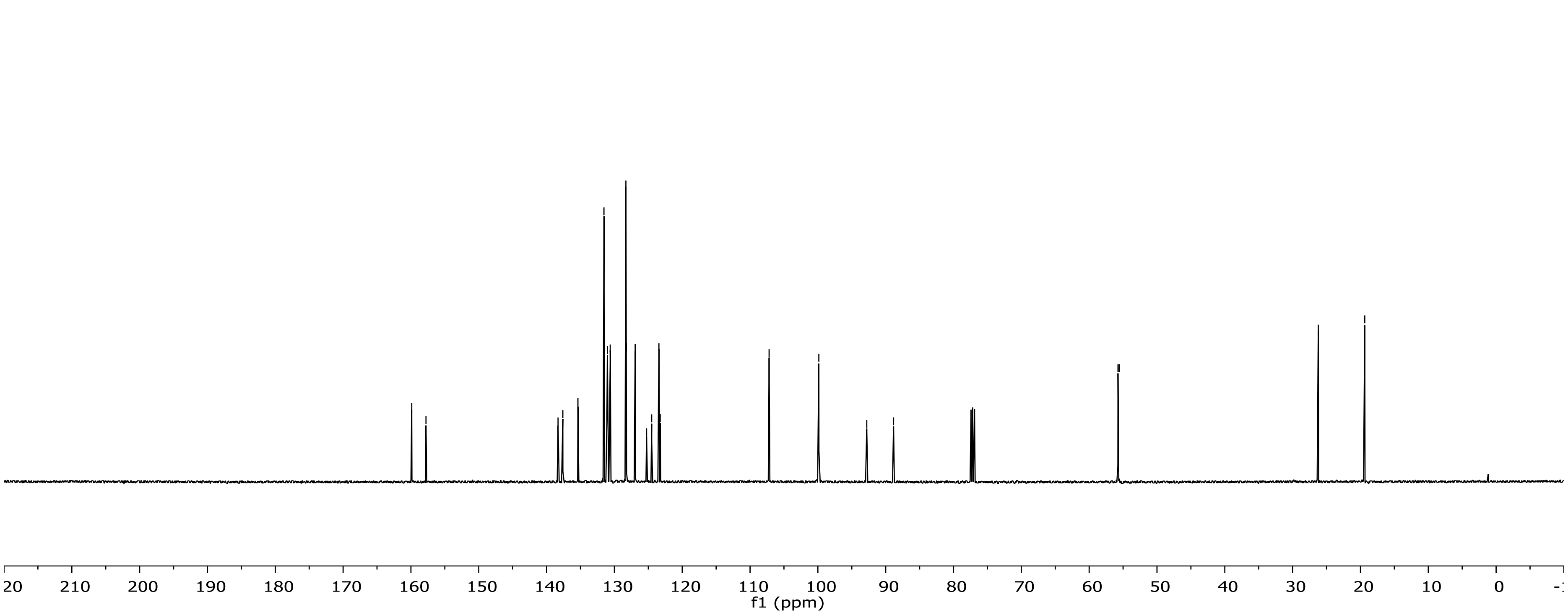

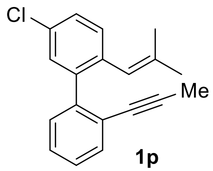

1p

^{1}H-NMR (300 MHz, $CDCl_3$)

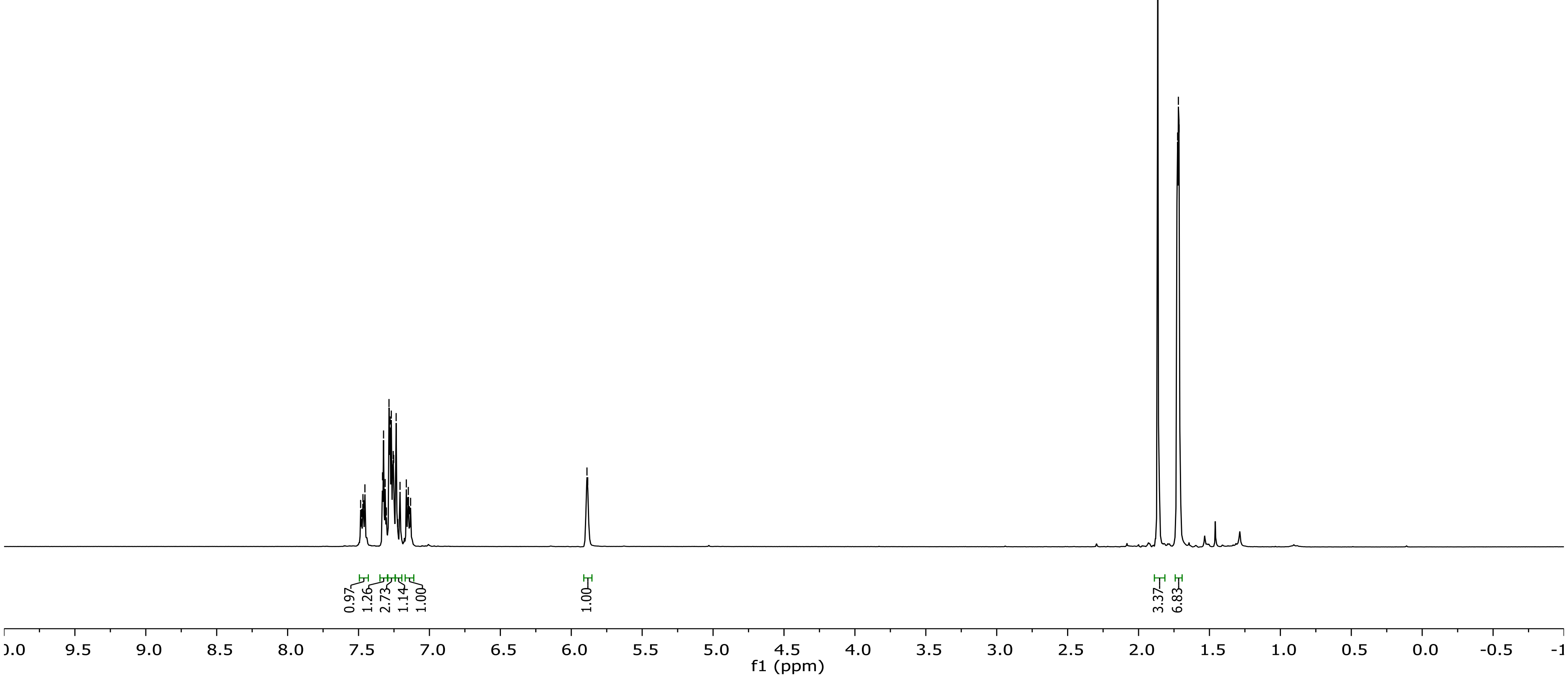

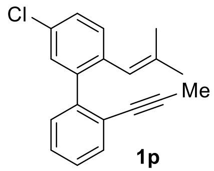

1p

^{13}C-NMR (75 MHz, CDCl$_3$)

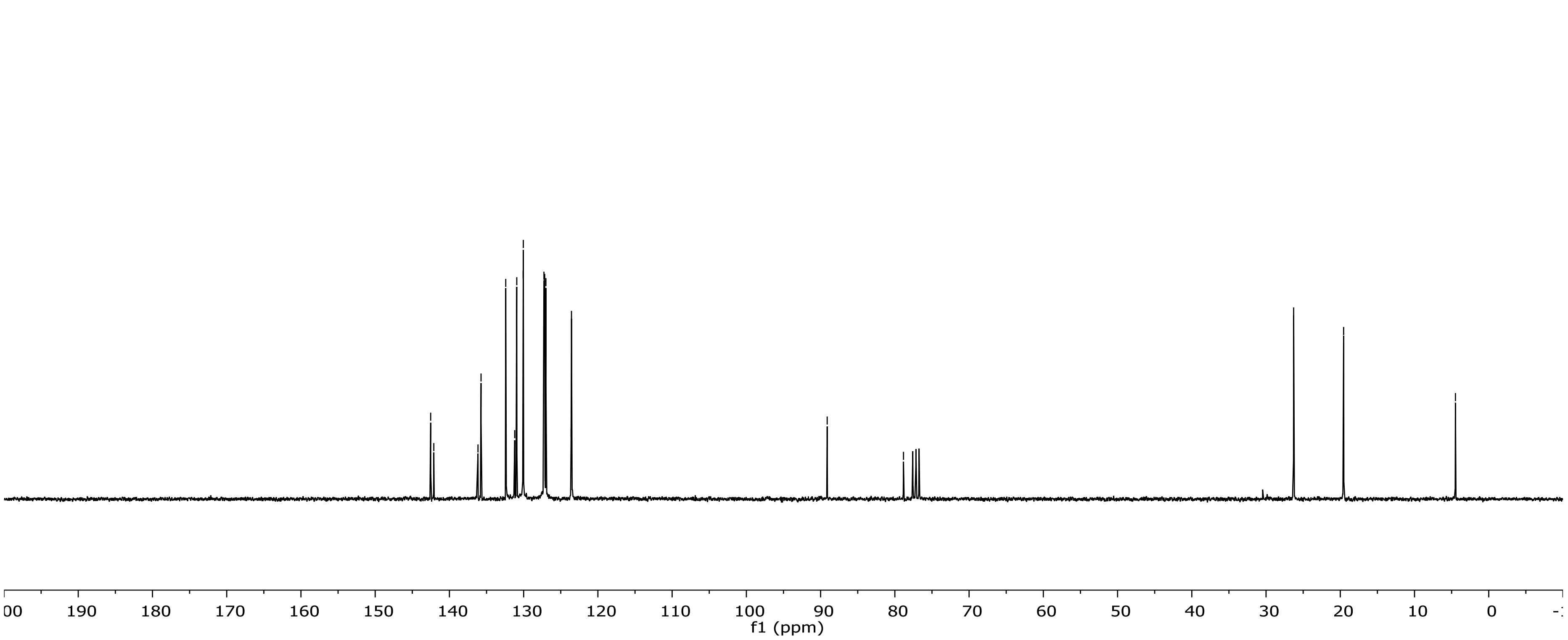

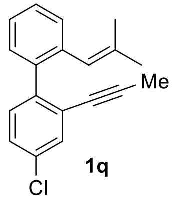

1q

^{1}H-NMR (300 MHz, $CDCl_3$)

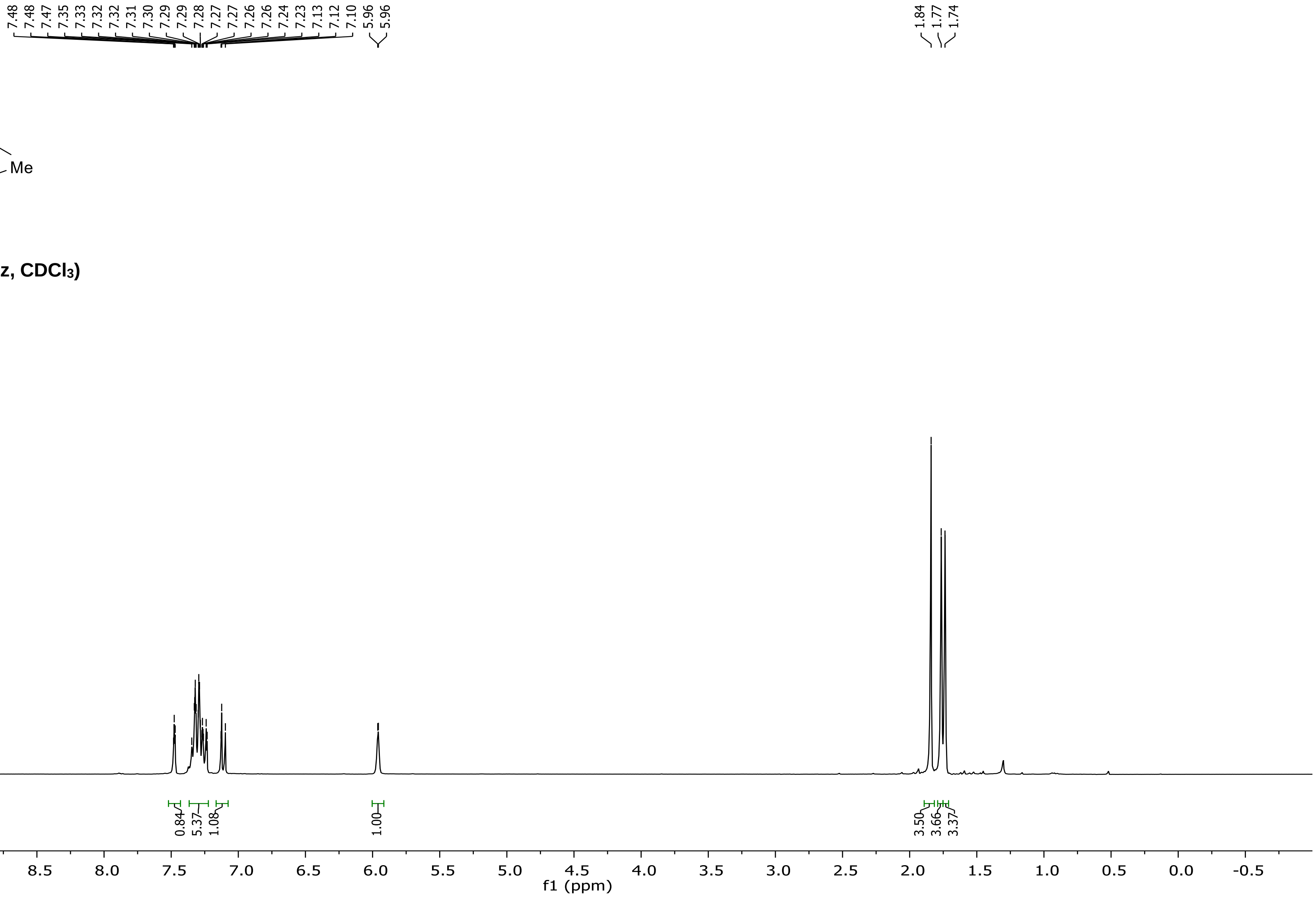

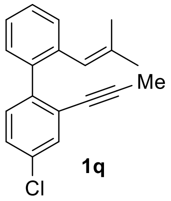

1q

^{13}C-NMR (75 MHz, $CDCl_3$)

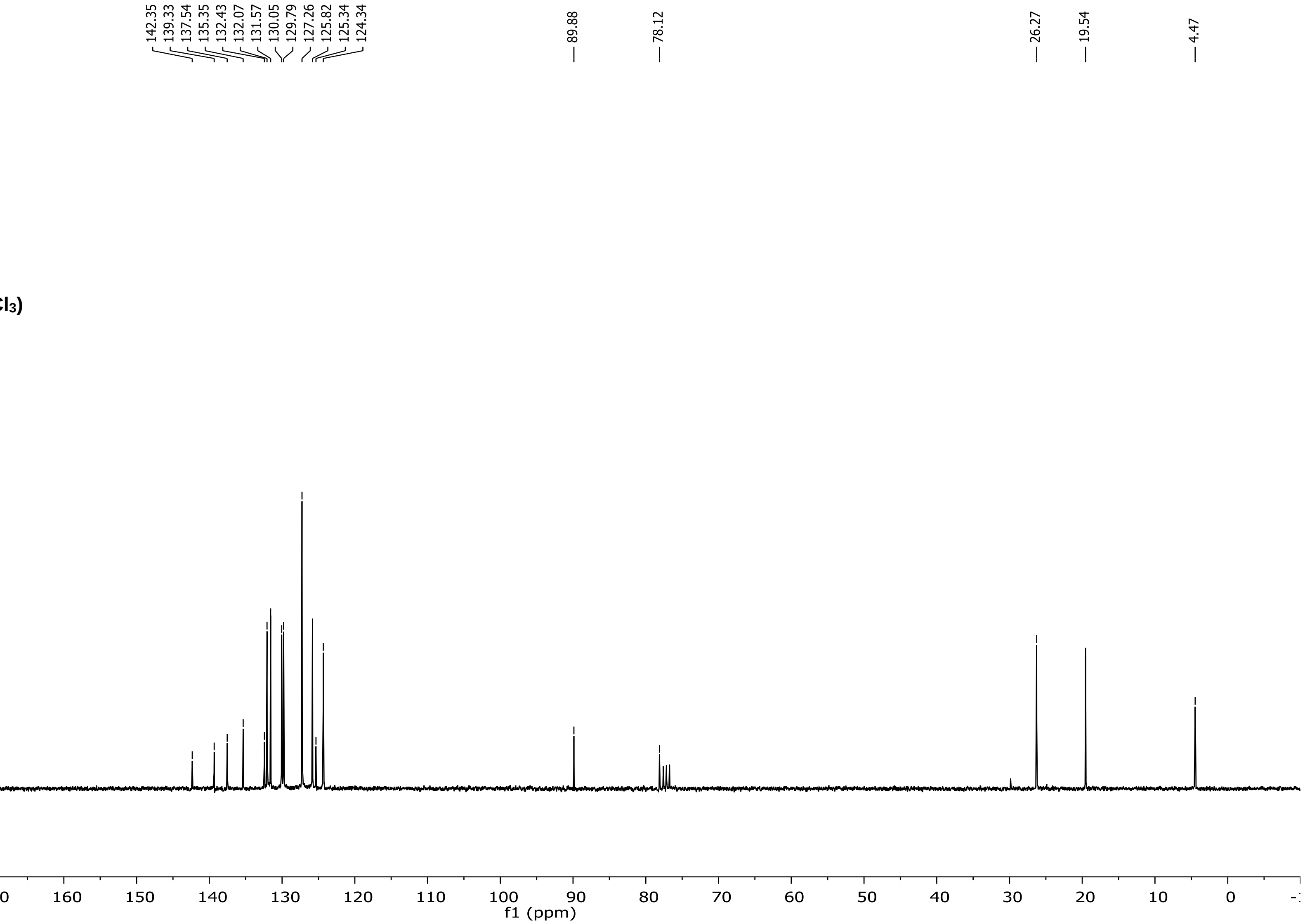

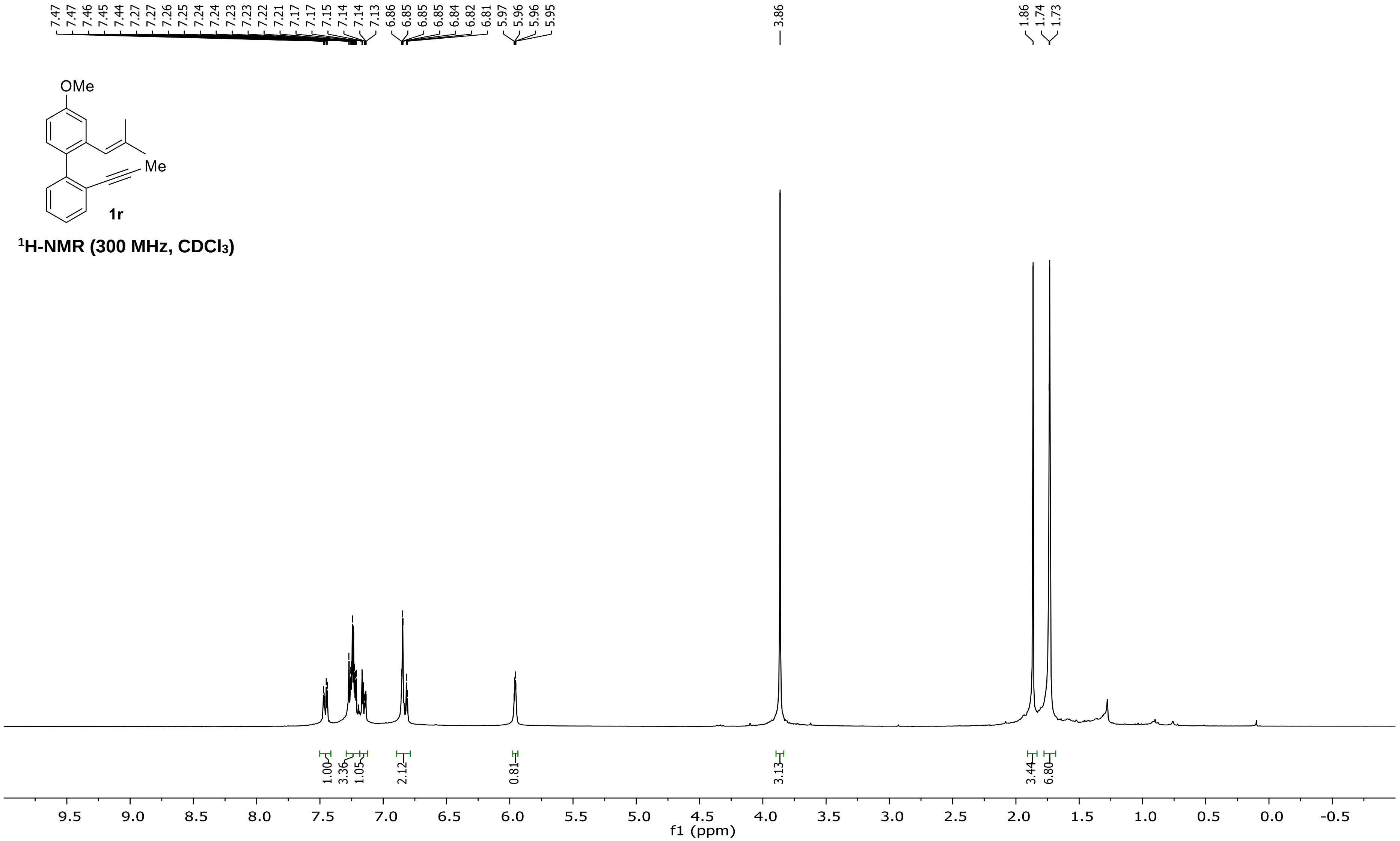
7.47 7.47 7.46 7.45 7.44 7.27 7.27 7.26 7.25 7.24 7.24 7.23 7.23 7.22 7.21 7.17 7.17 7.15 7.14 7.14 7.13 6.86 6.85 6.85 6.85 6.84 6.82 6.81 5.97 5.96 5.96 5.95
3.86
1.86 1.74 1.73
OMe
Me
1r
1H-NMR (300 MHz, CDCl3)
1.00 3.36 1.05 2.12 0.81 3.13 3.44 6.80
9.5 9.0 8.5 8.0 7.5 7.0 6.5 6.0 5.5 5.0 4.5 4.0 3.5 3.0 2.5 2.0 1.5 1.0 0.5 0.0 -0.5
f1 (ppm)

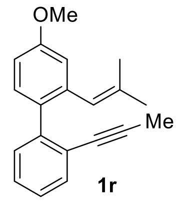

1r

^{13}C-NMR (75 MHz, CDCl$_3$)

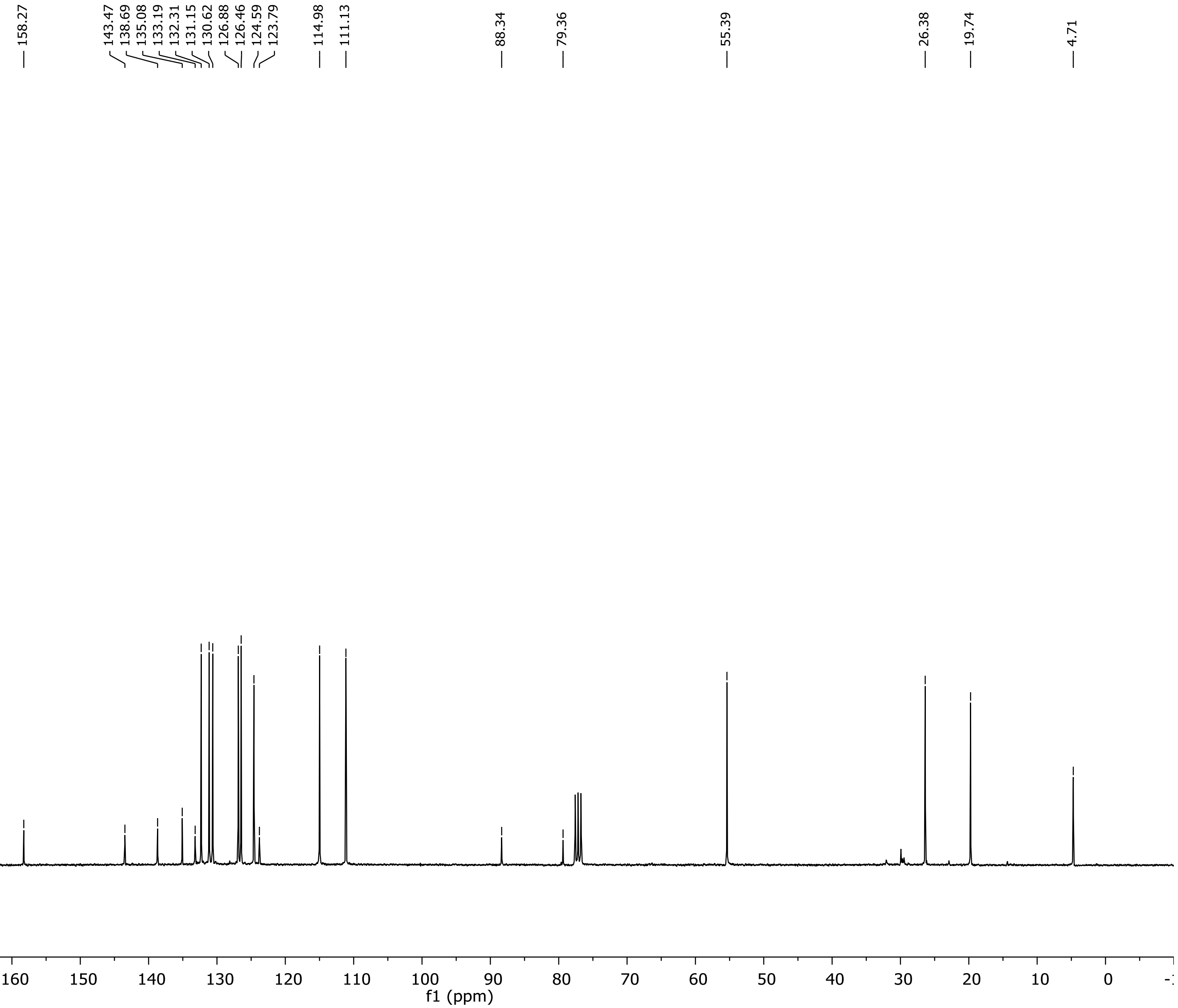

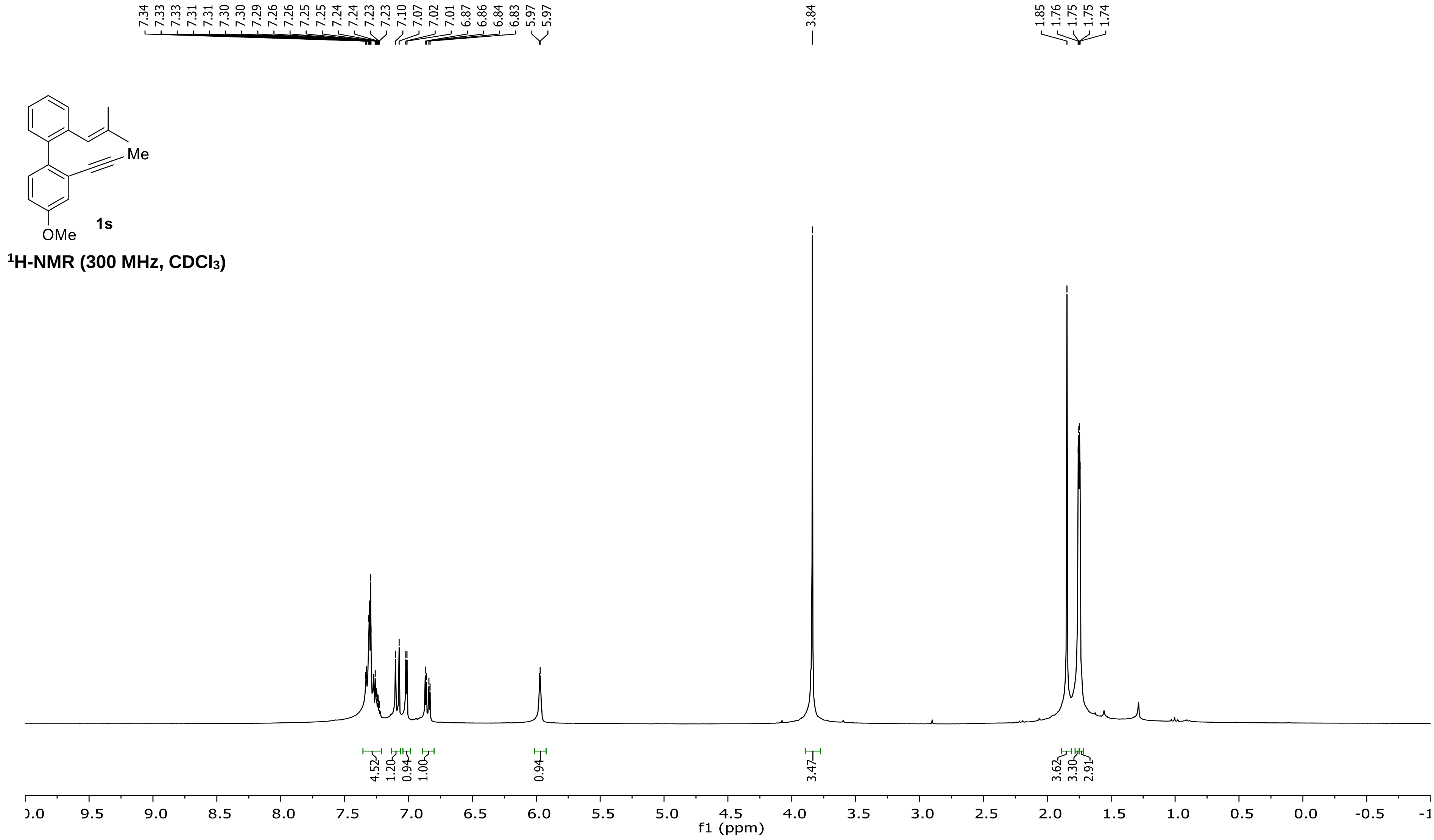

^{1}H-NMR (300 MHz, $CDCl_3$)

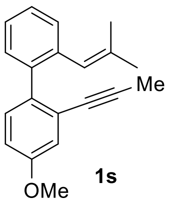

1s

^{13}C-NMR (75 MHz, $CDCl_3$)

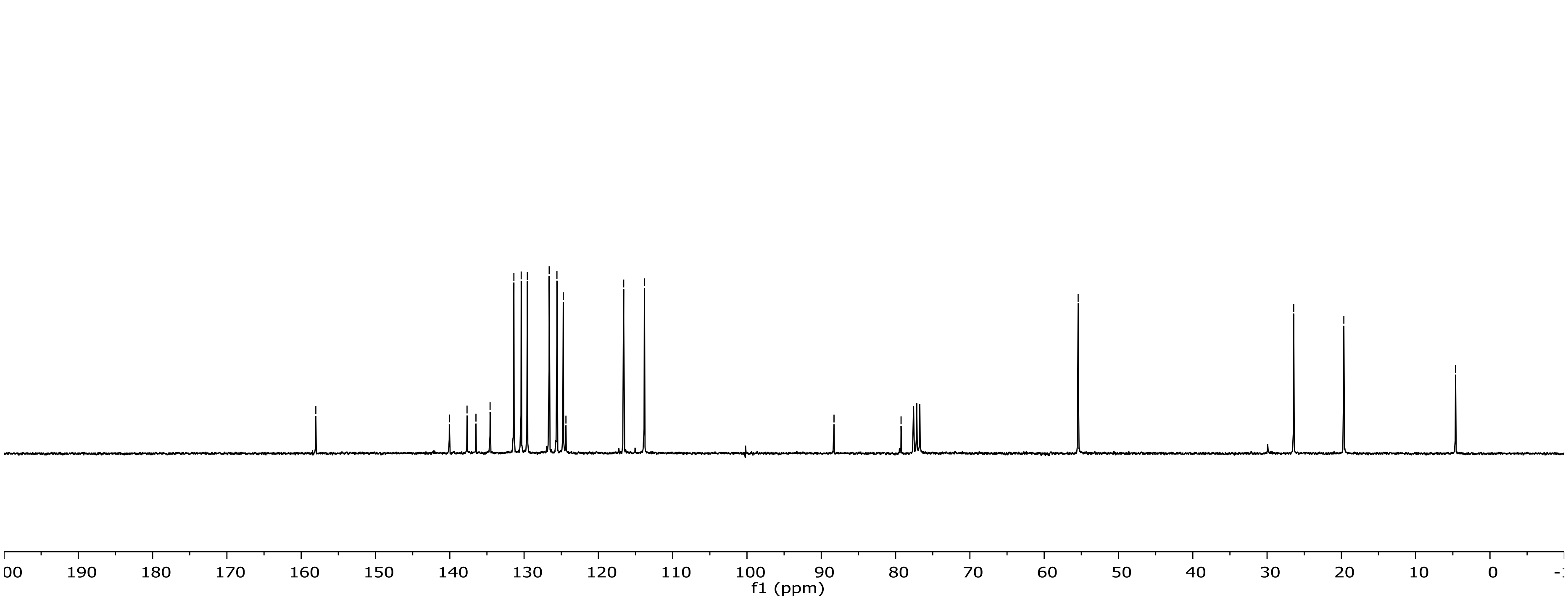

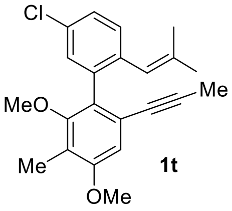

1t

^{1}H-NMR (300 MHz, $CDCl_3$)

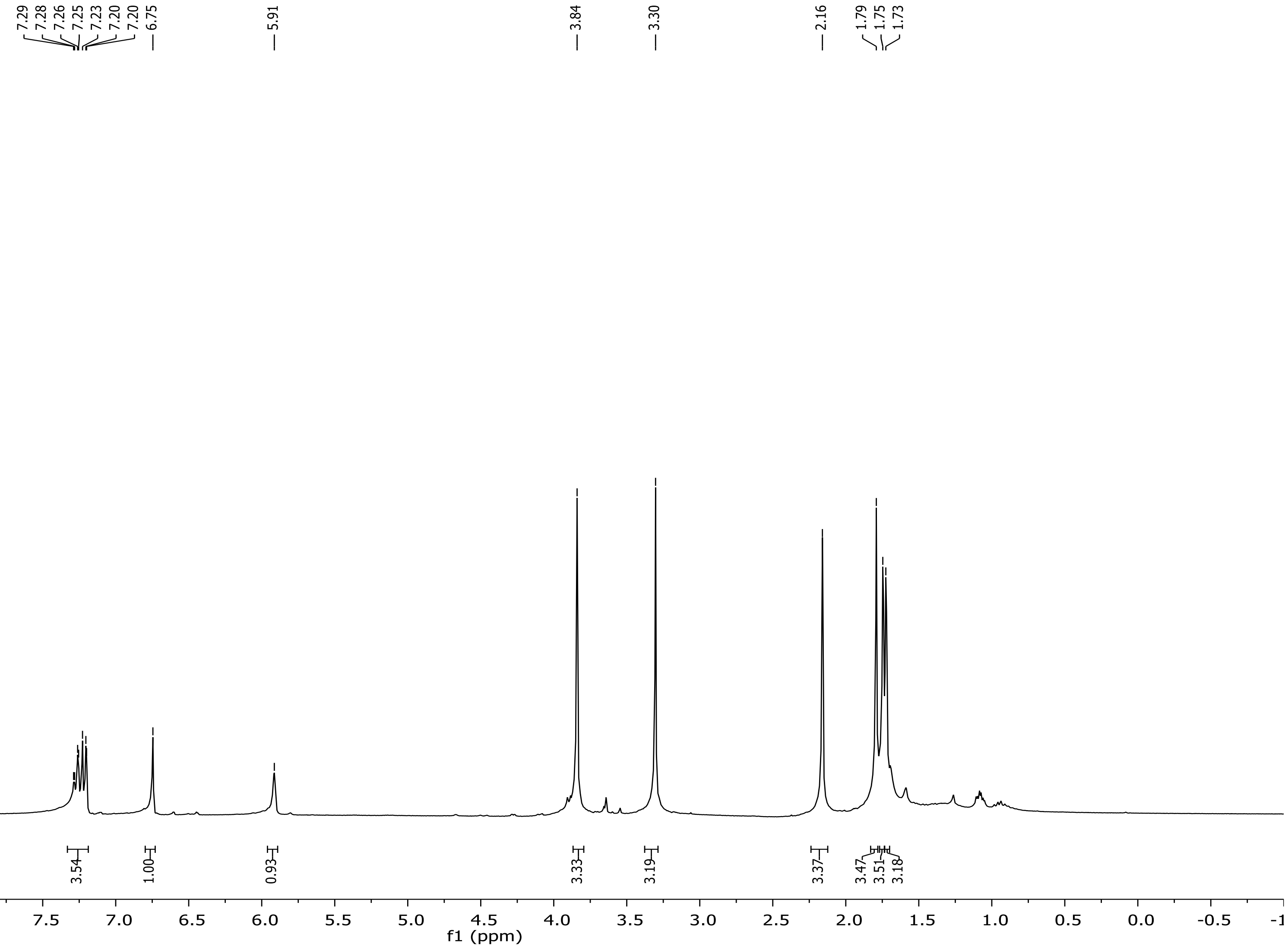

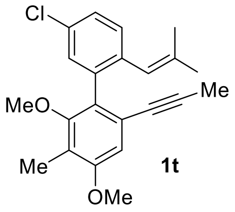

1t

^{13}C-NMR (75 MHz, $CDCl_3$)

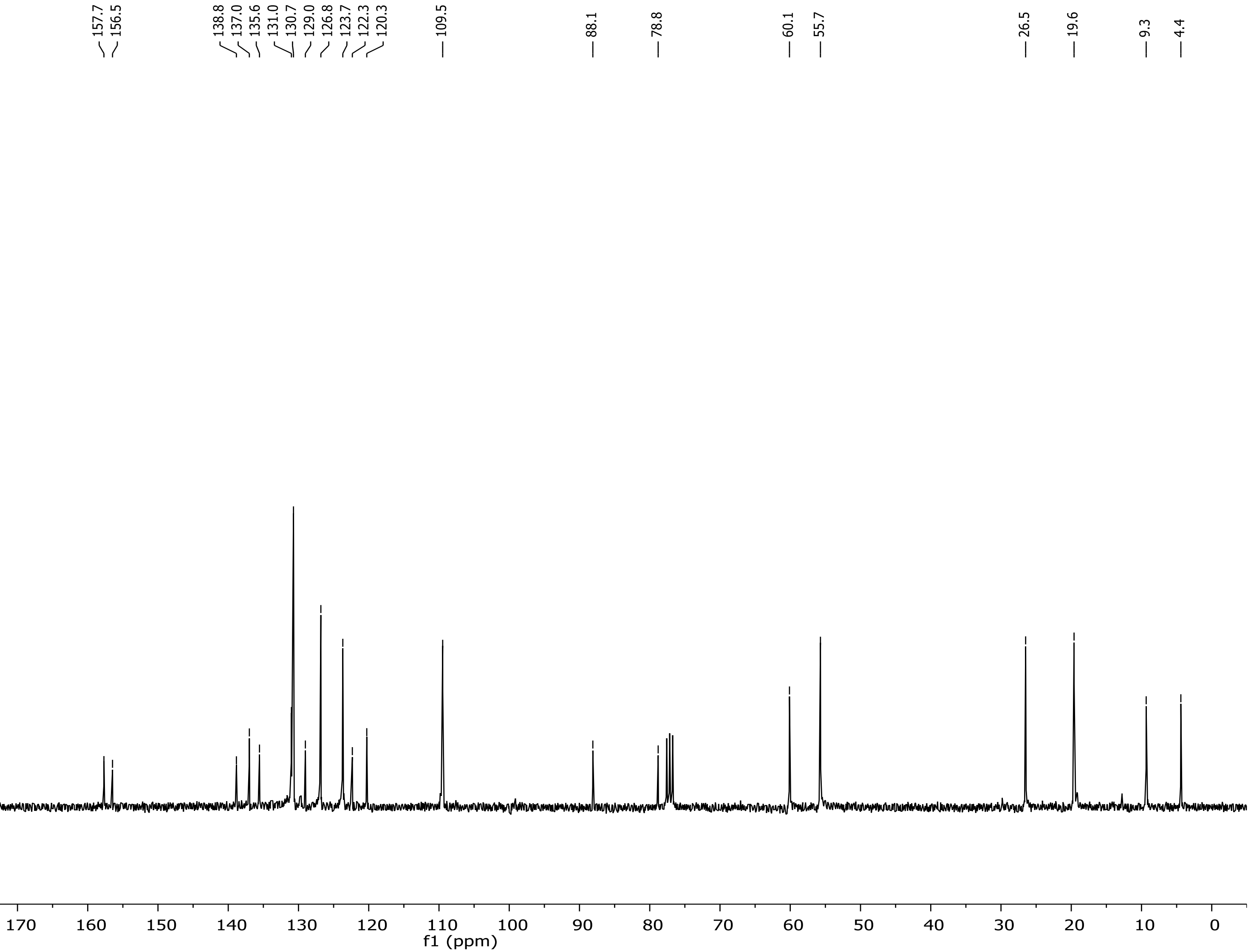

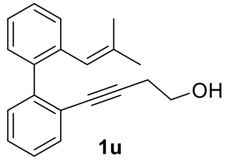

1u

^{1}H-NMR (400 MHz, $CDCl_3$)

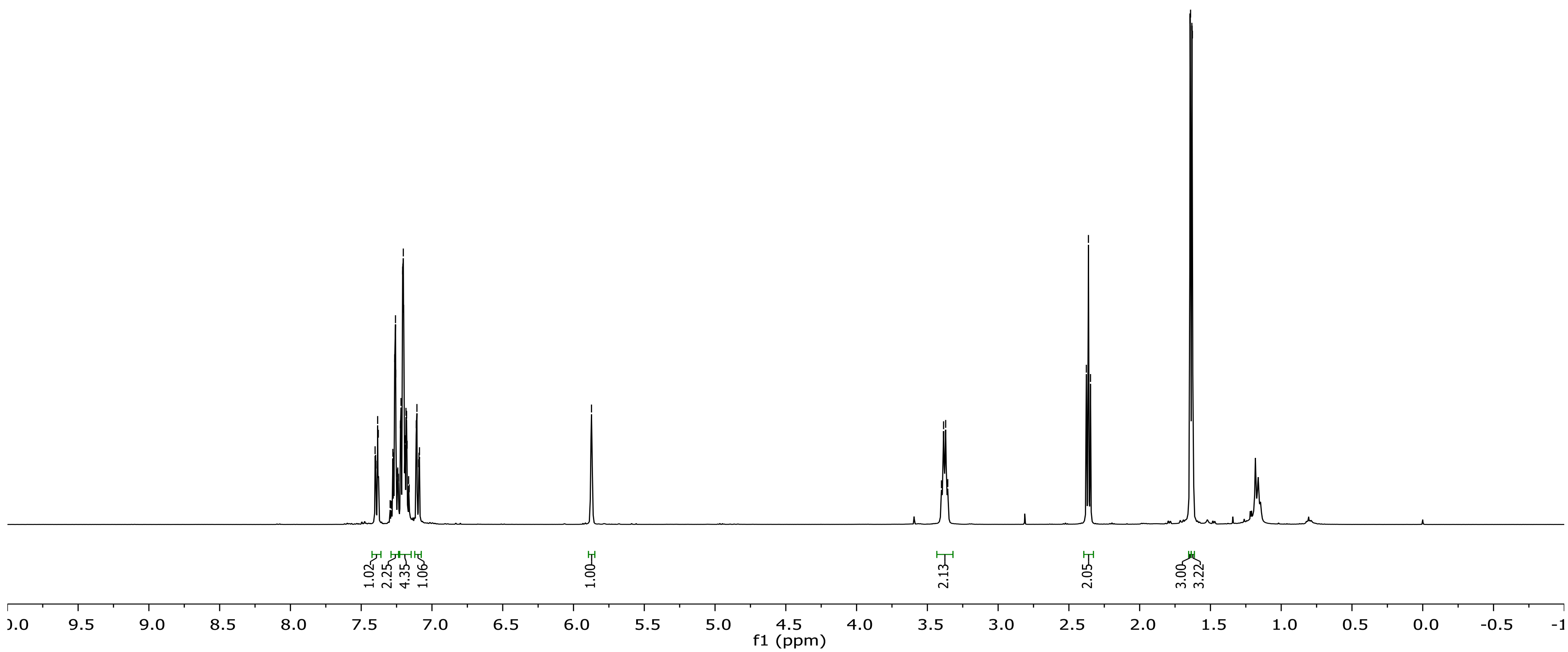

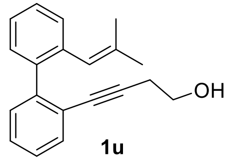

1u

^{13}C-NMR (100 MHz, CDCl$_3$)

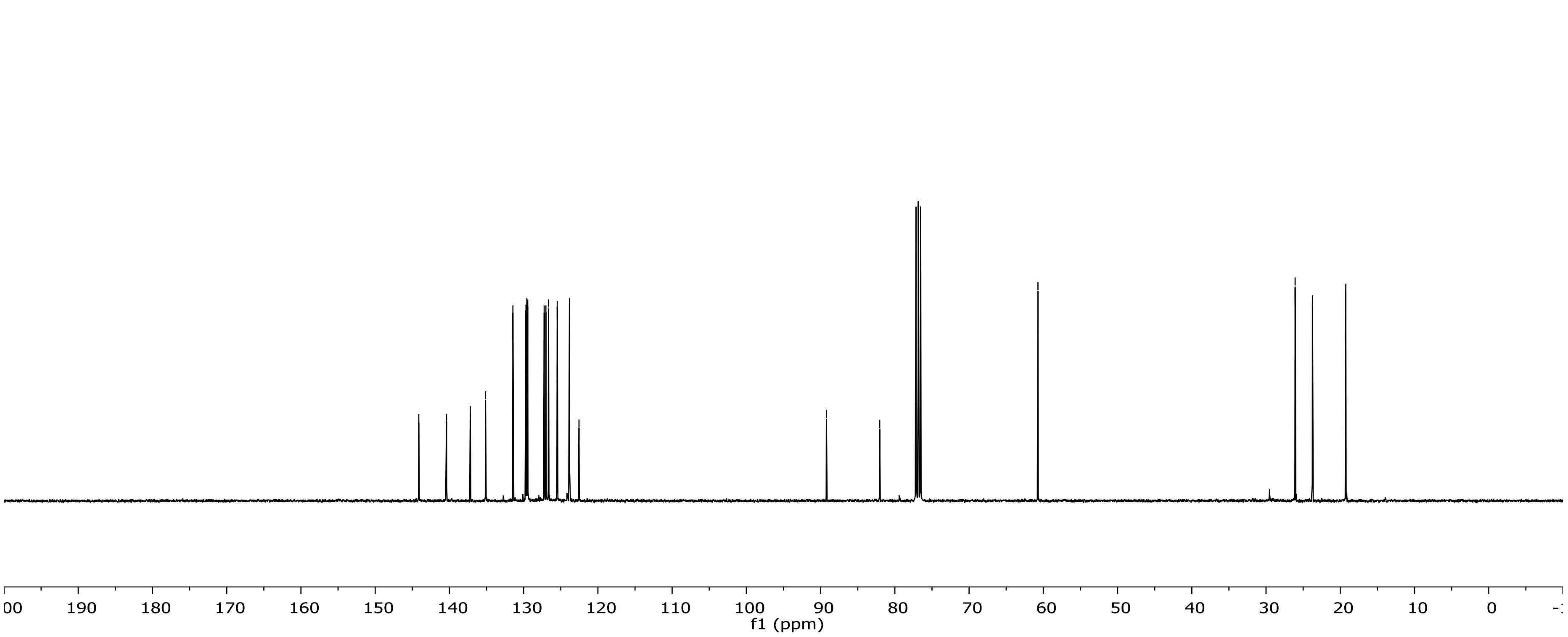

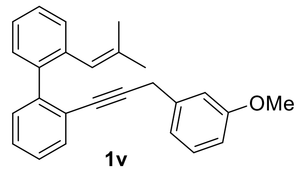

1v

^{1}H-NMR (400 MHz, $CDCl_3$)

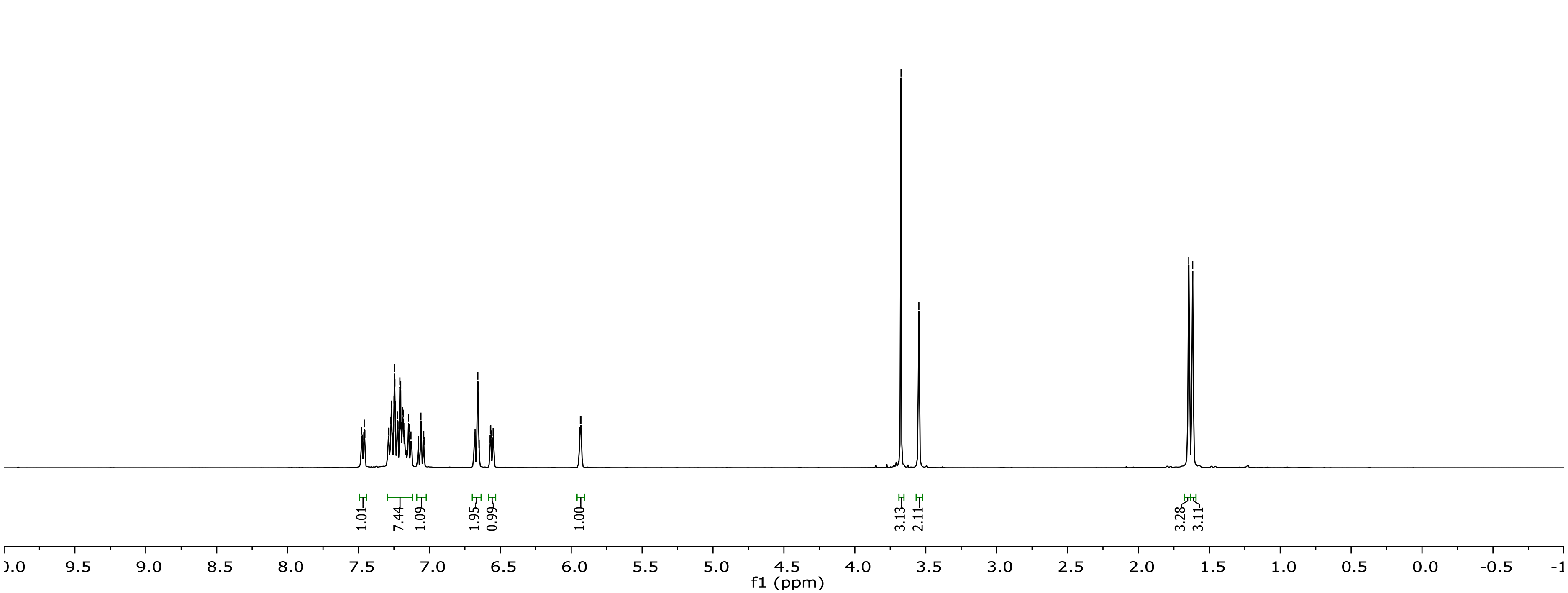

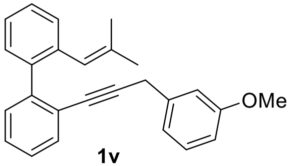

1v

^{13}C-NMR (100 MHz, $CDCl_3$)

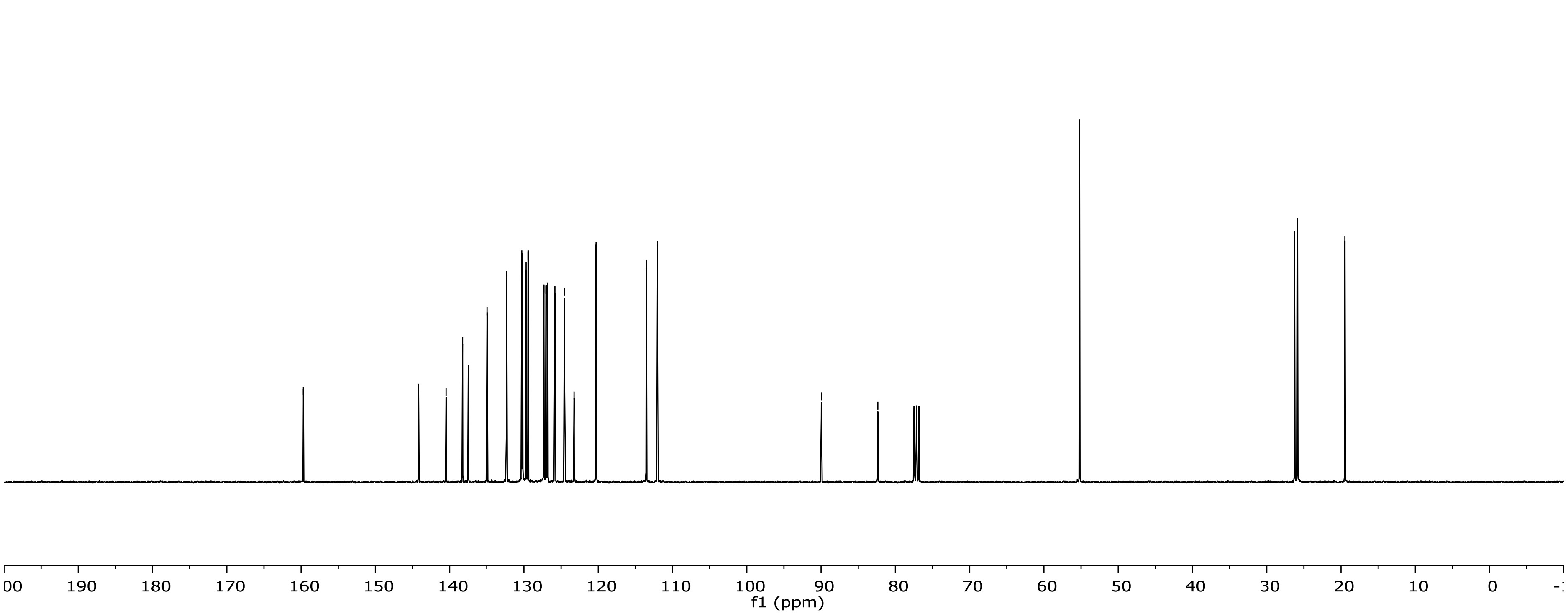

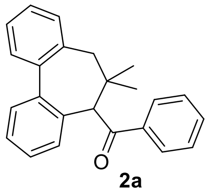

2a

¹H-NMR (300 MHz, CDCl$_3$)

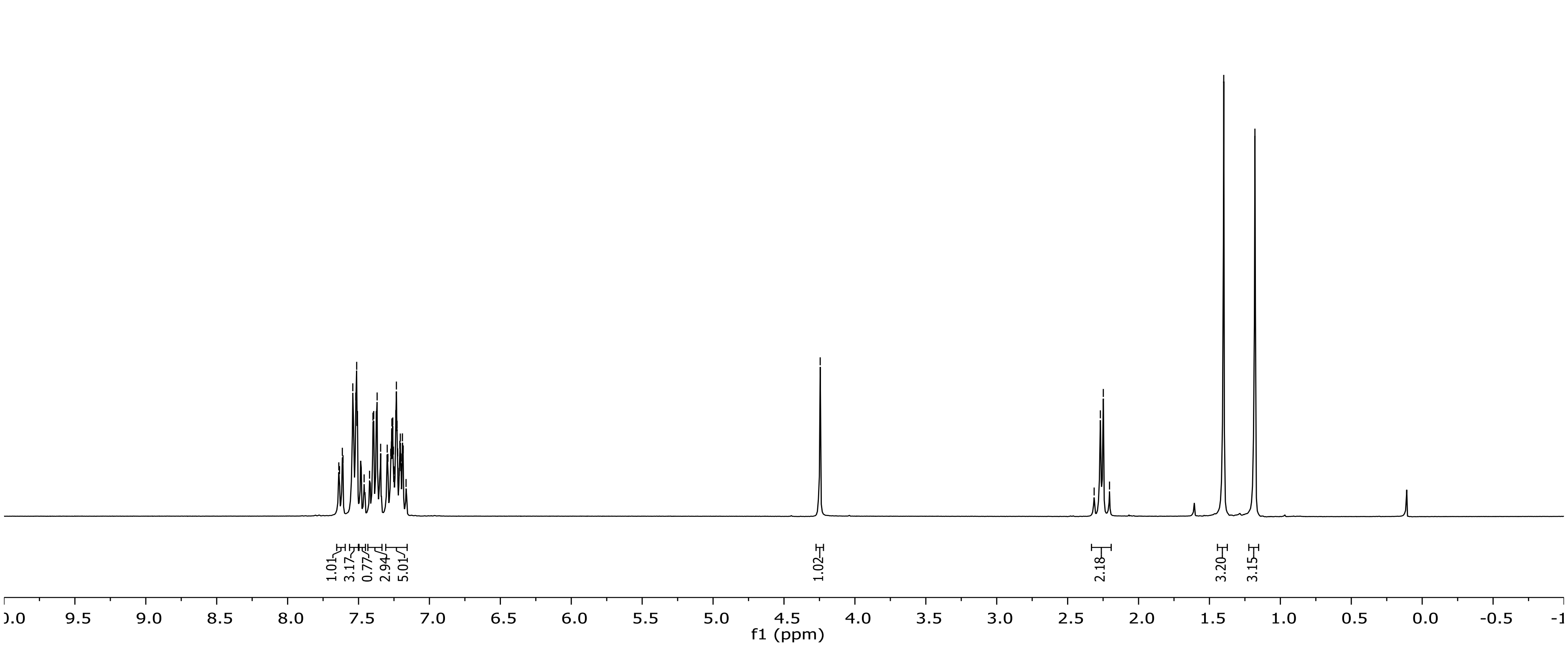

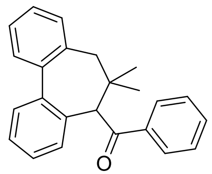

2a

^{13}C-NMR (75 MHz, CDCl$_3$)

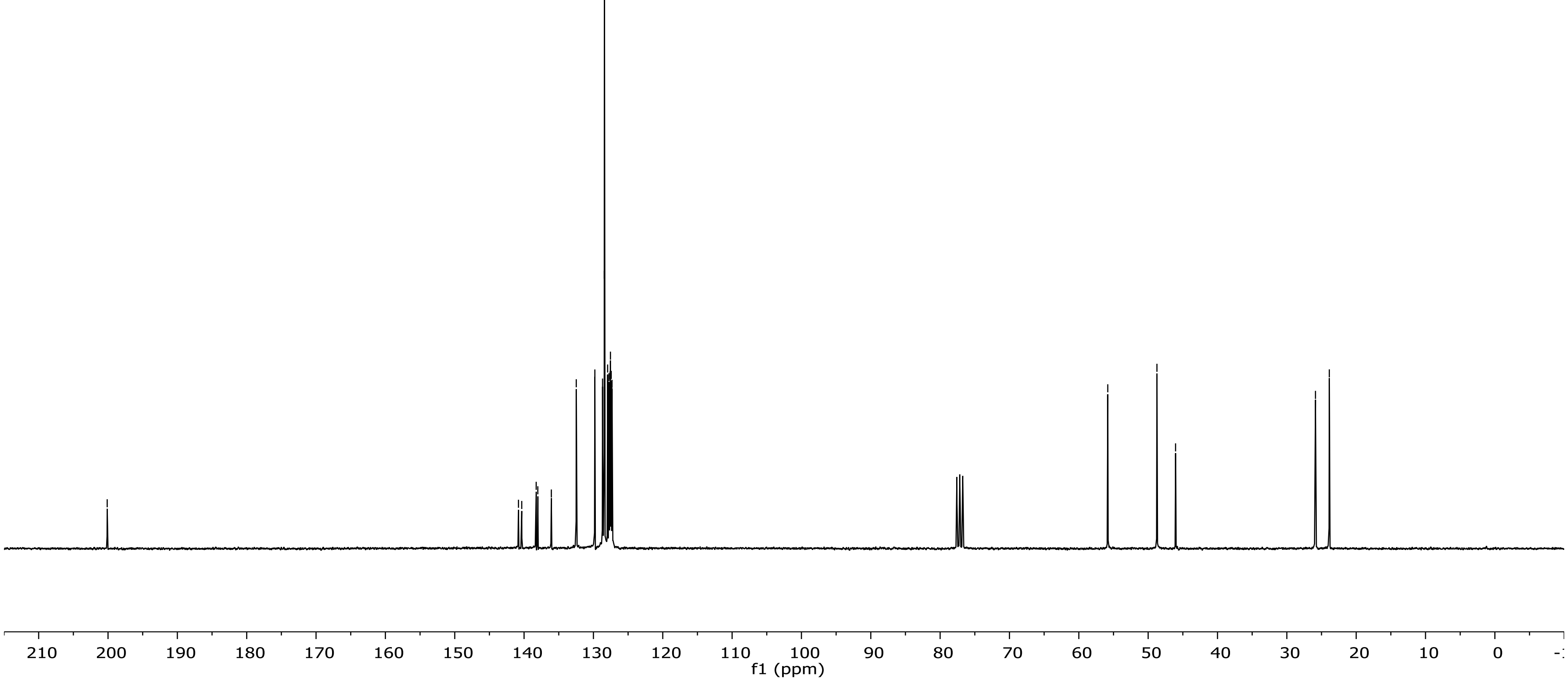

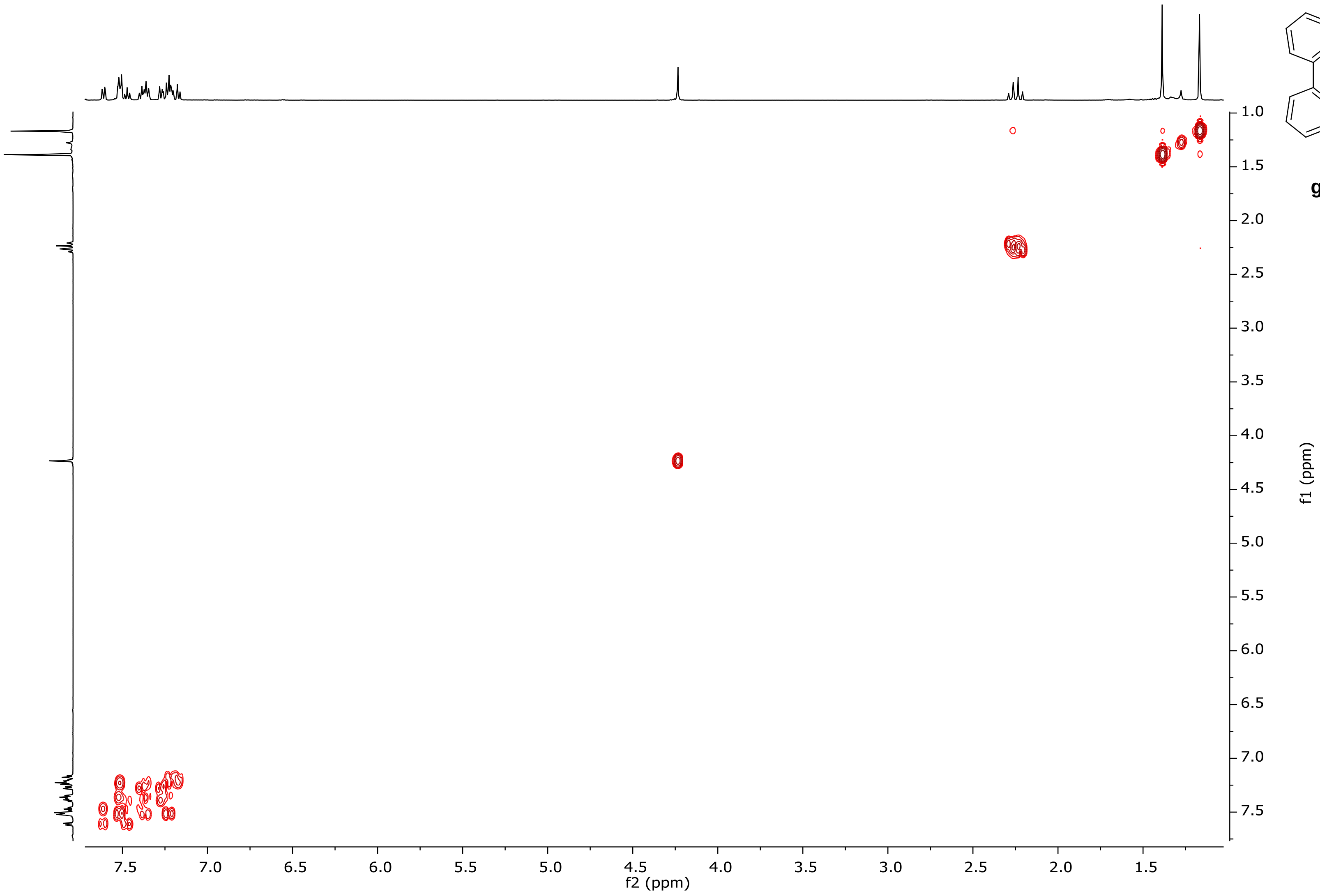
7.5
7.0
6.5
6.0
5.5
5.0
4.5
4.0
3.5
3.0
2.5
2.0
1.5
f2 (ppm)
1.0
1.5
2.0
2.5
3.0
3.5
4.0
4.5
5.0
5.5
6.0
6.5
7.0
7.5
f1 (ppm)

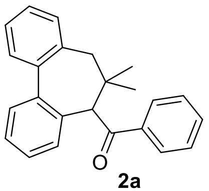
O

2a

gCOSY-NMR

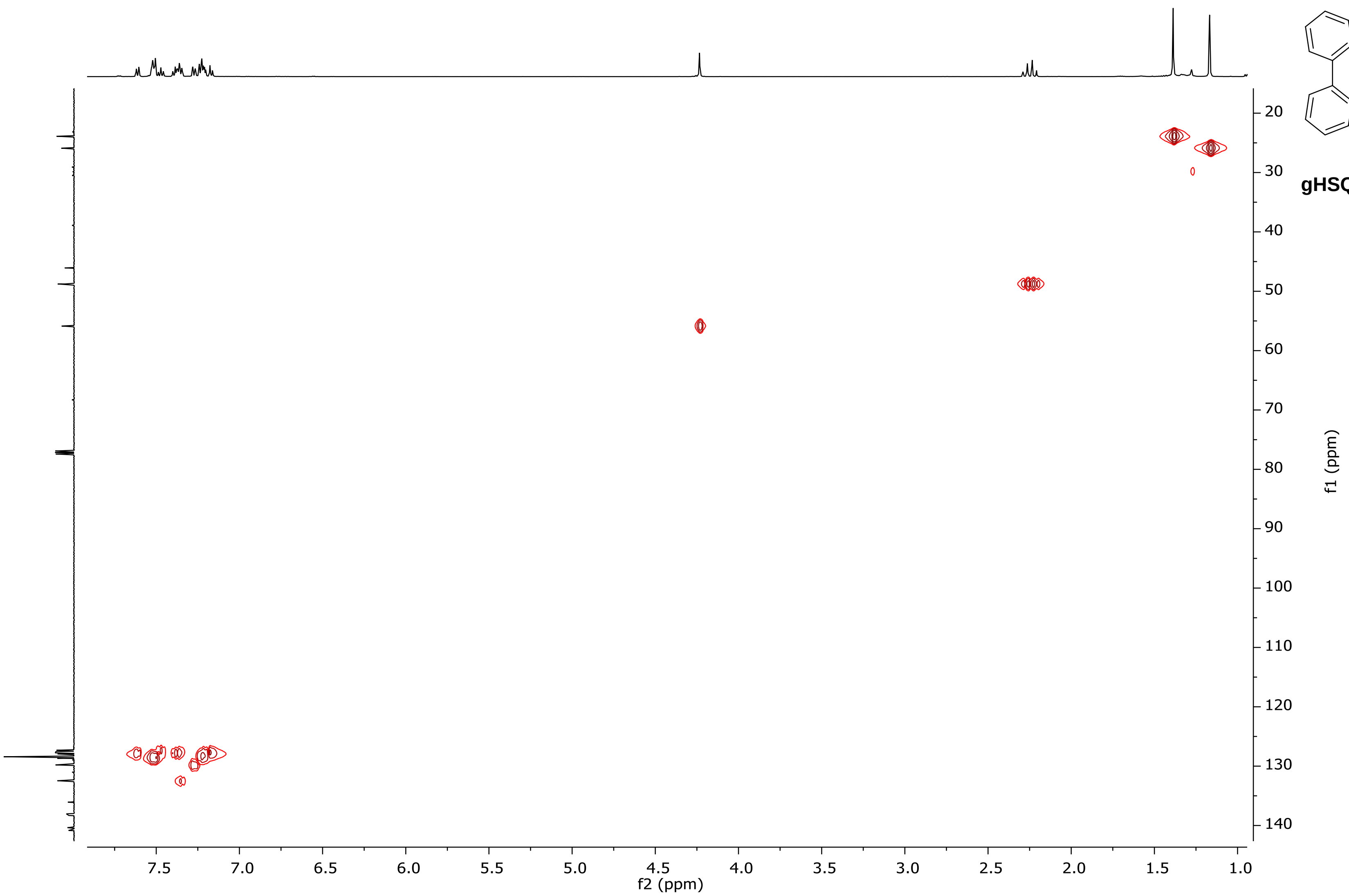

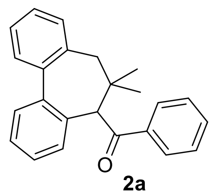

2a

gHSQC-NMR

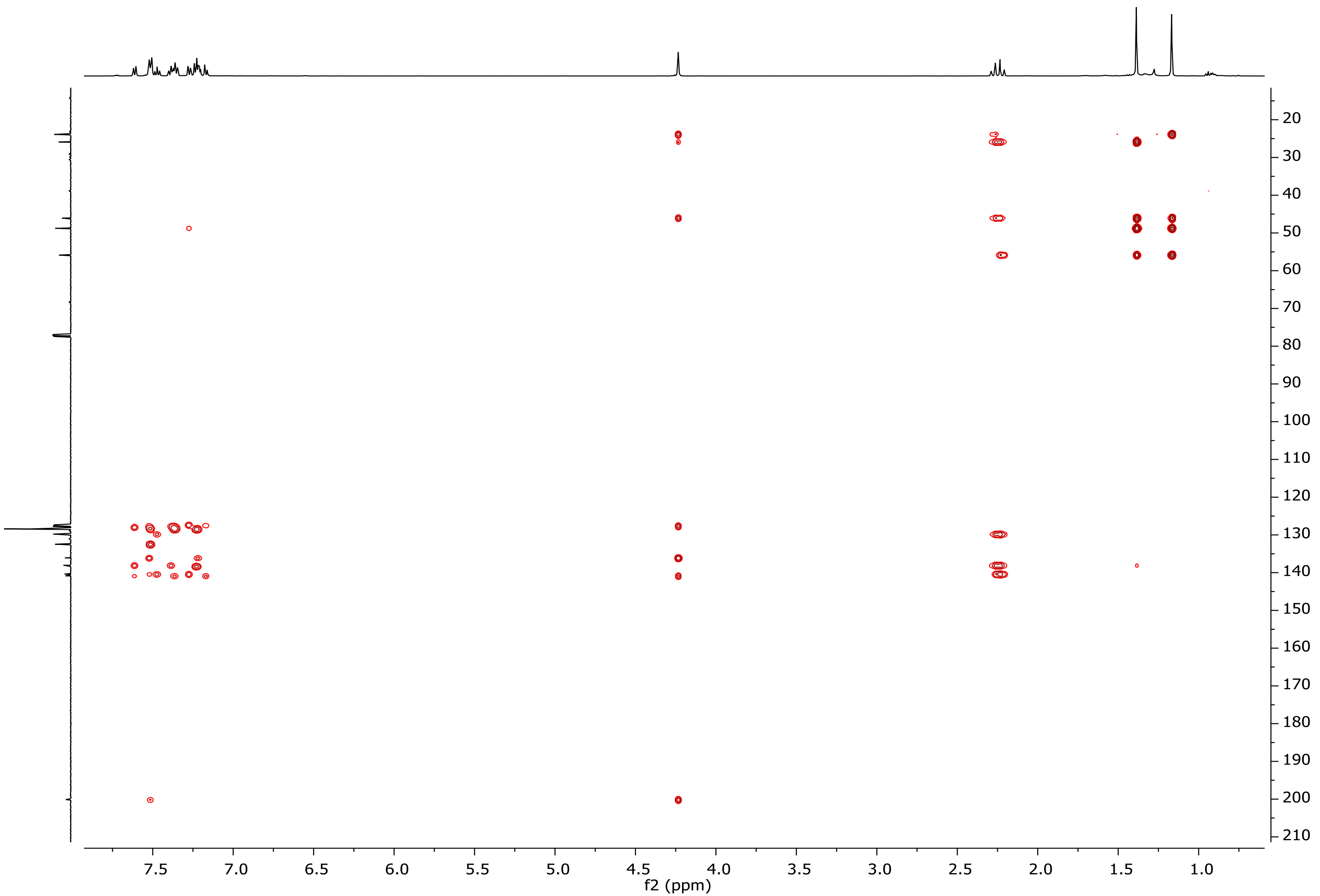

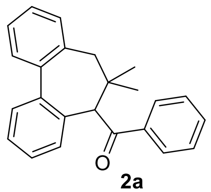

2a

gHMBC-NMR

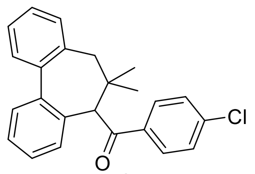

2c

^{1}H-NMR (300 MHz, $CDCl_3$)

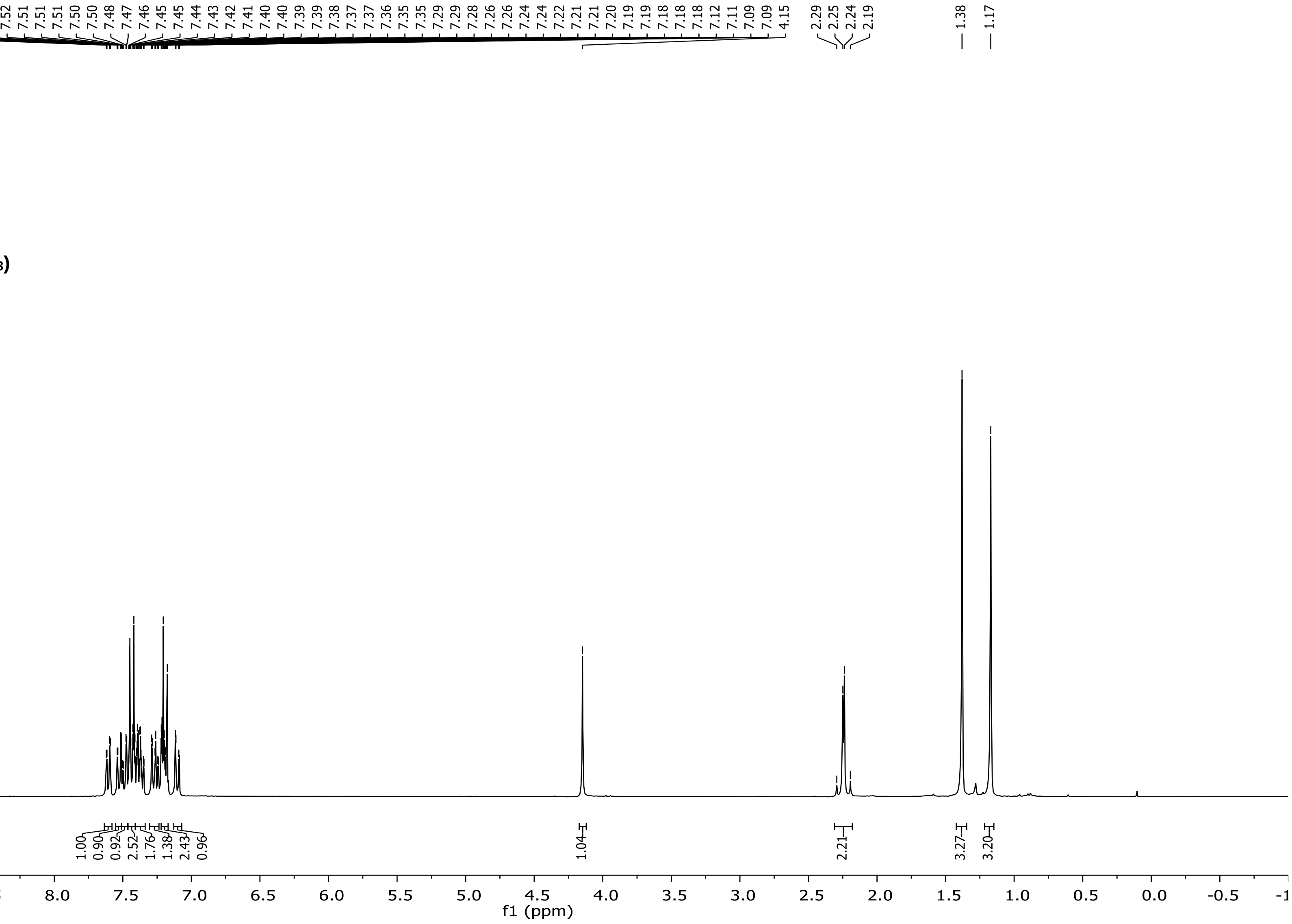

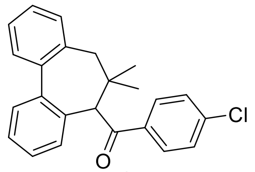

2c

^{13}C-NMR (75 MHz, $CDCl_3$)

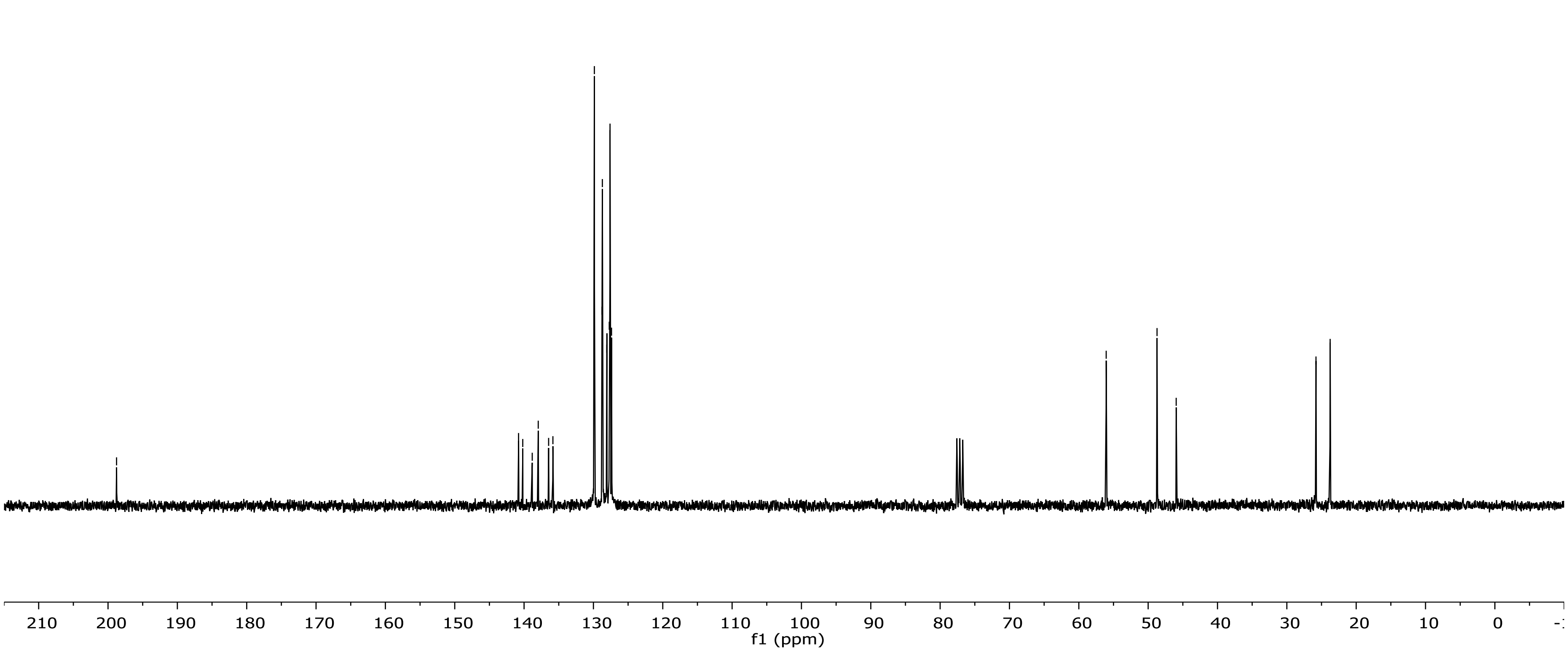

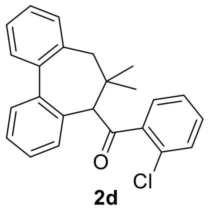

2d

^{1}H-NMR (300 MHz, $CDCl_3$)

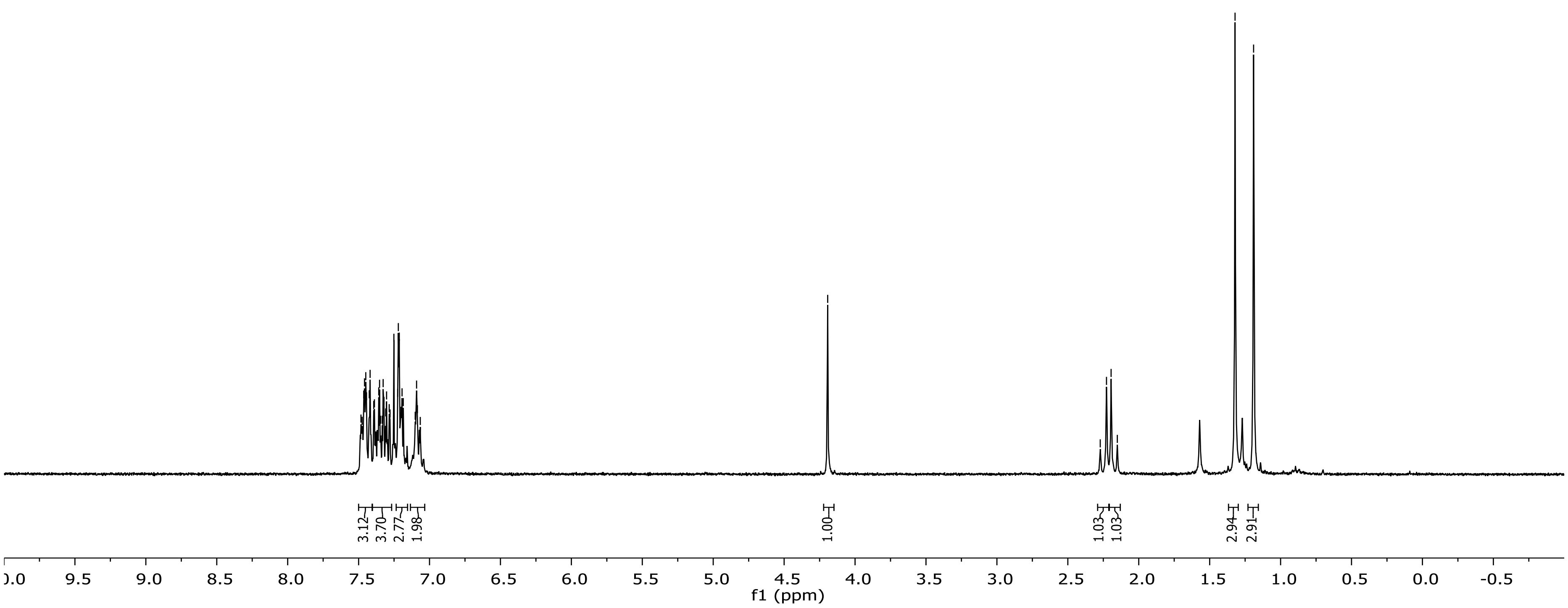

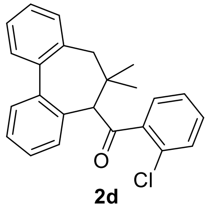

2d

^{13}C-NMR (75 MHz, $CDCl_3$)

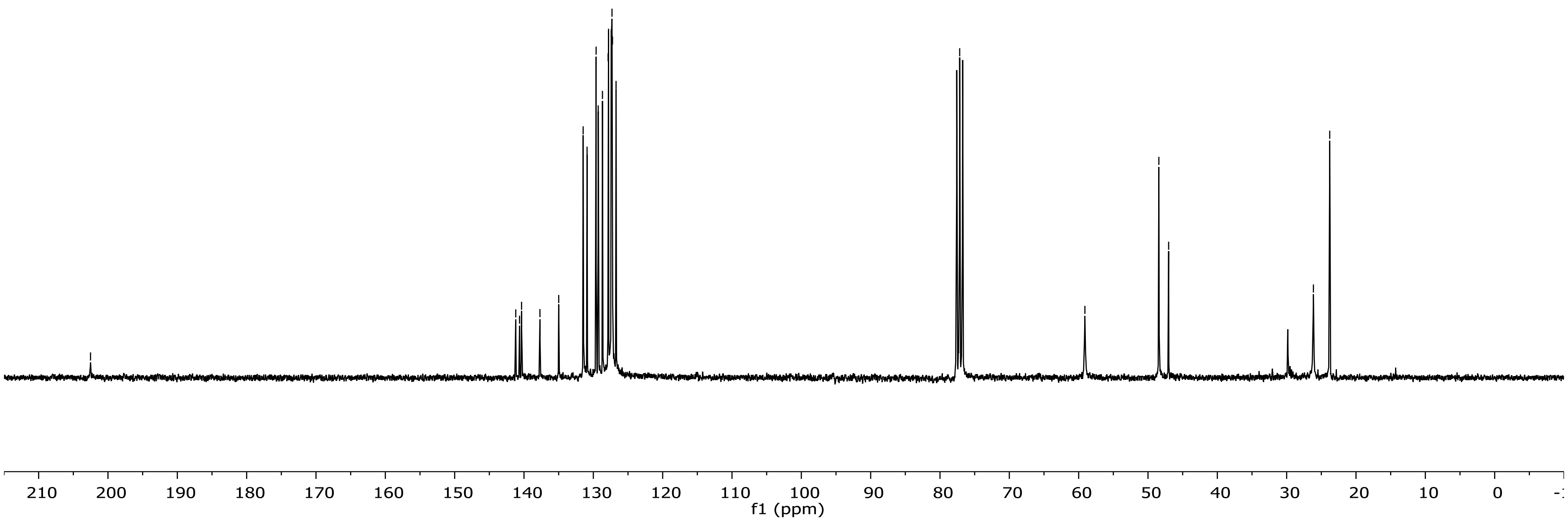

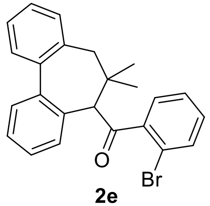

2e

^{1}H-NMR (300 MHz, $CDCl_3$)

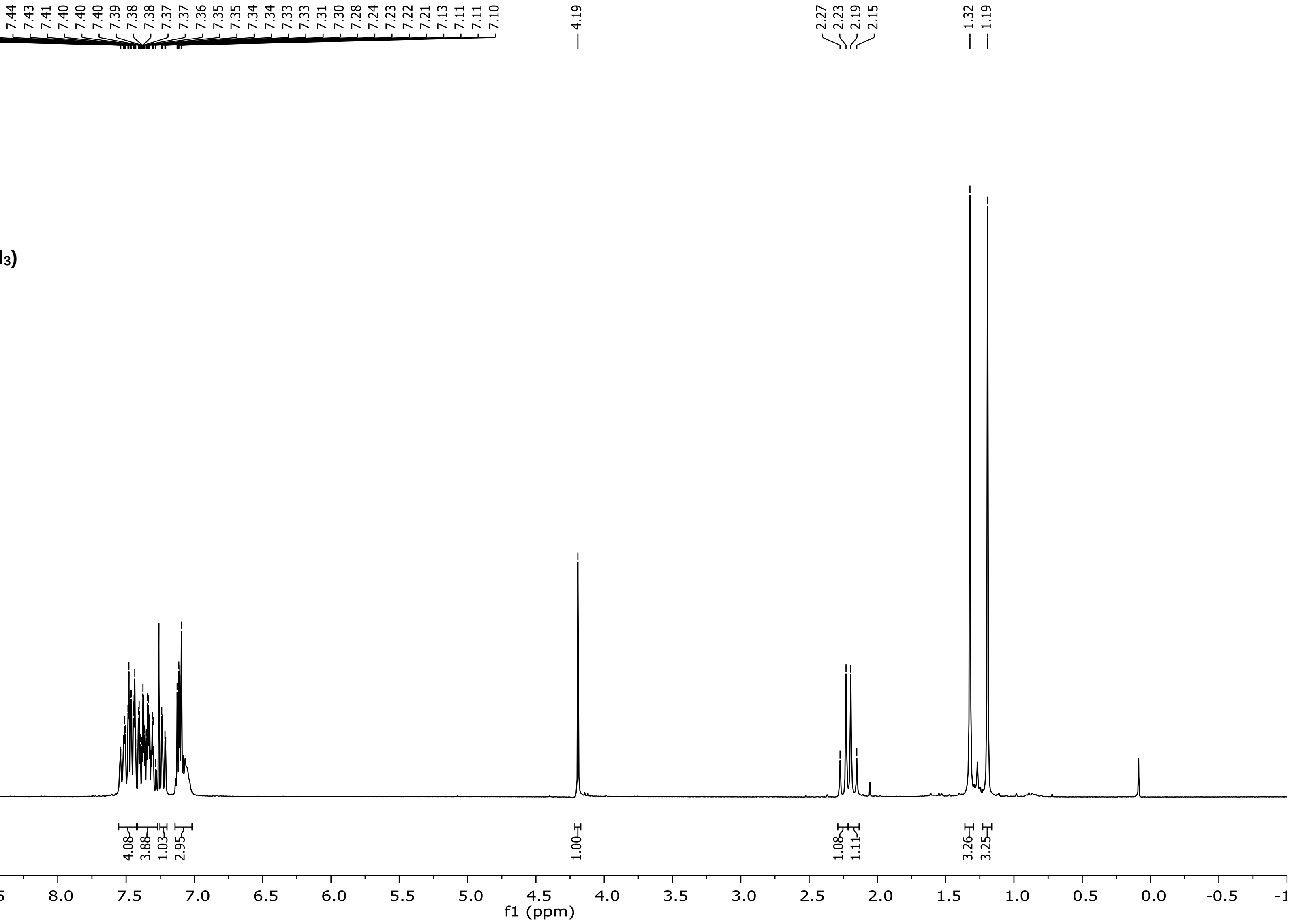

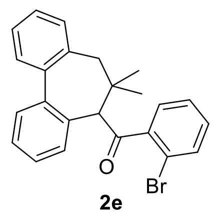

2e

^{13}C-NMR (75 MHz, CDCl$_3$)

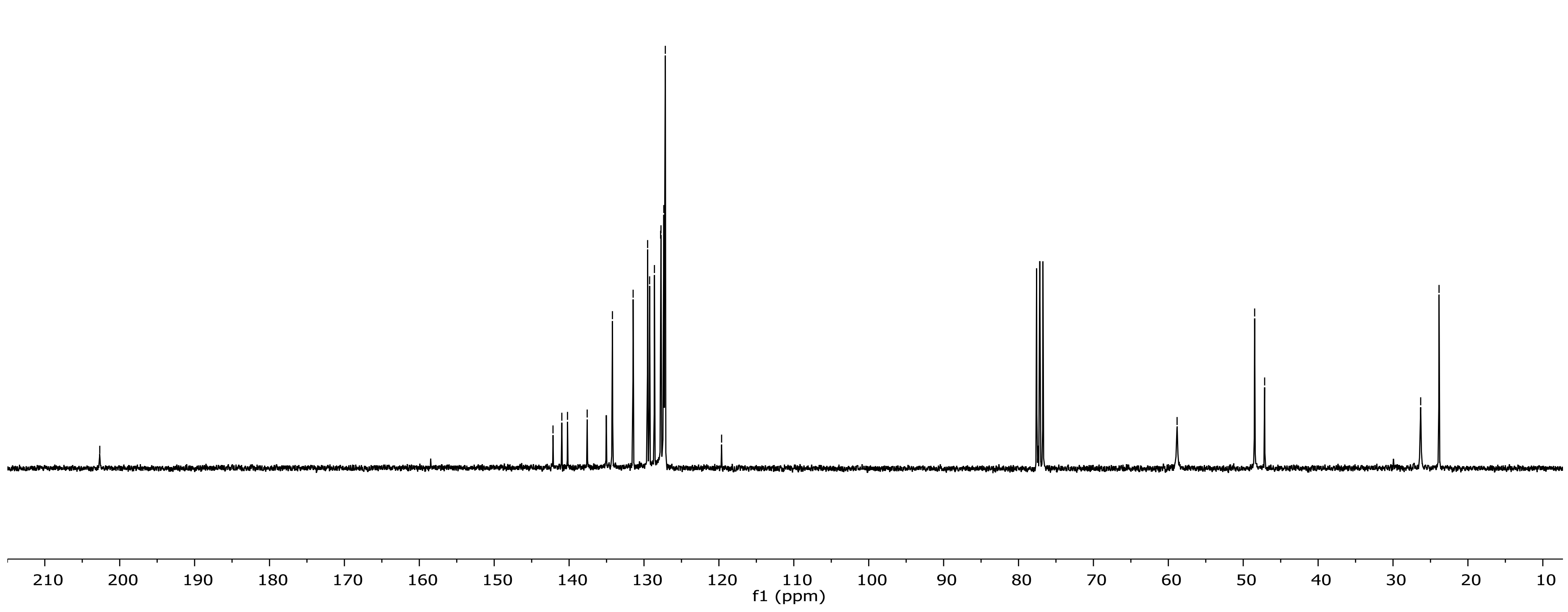

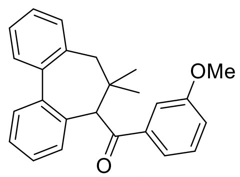

2f

^{1}H-NMR (300 MHz, $CDCl_3$)

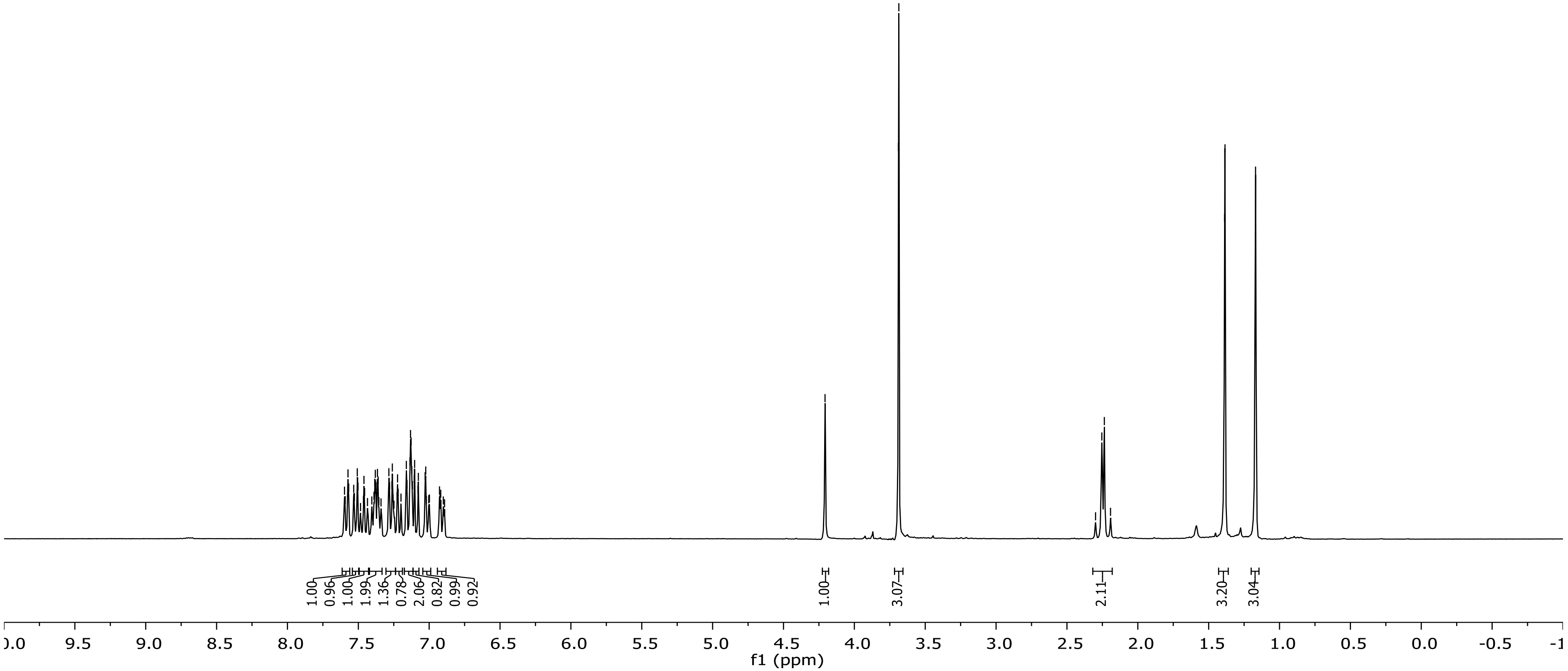

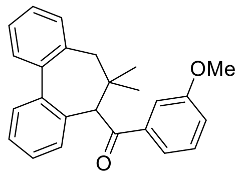

2f

^{13}C-NMR (75 MHz, $CDCl_3$)

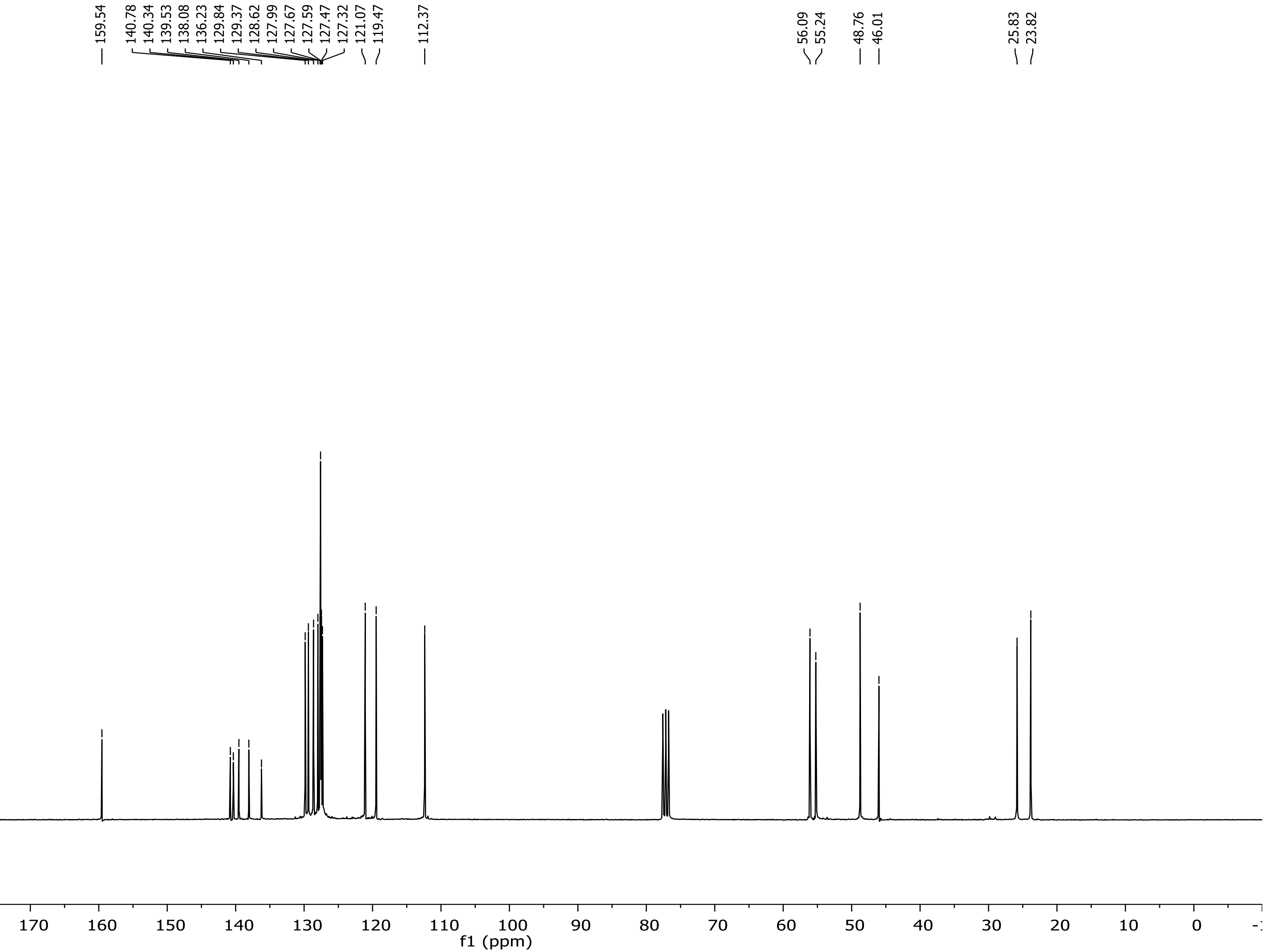

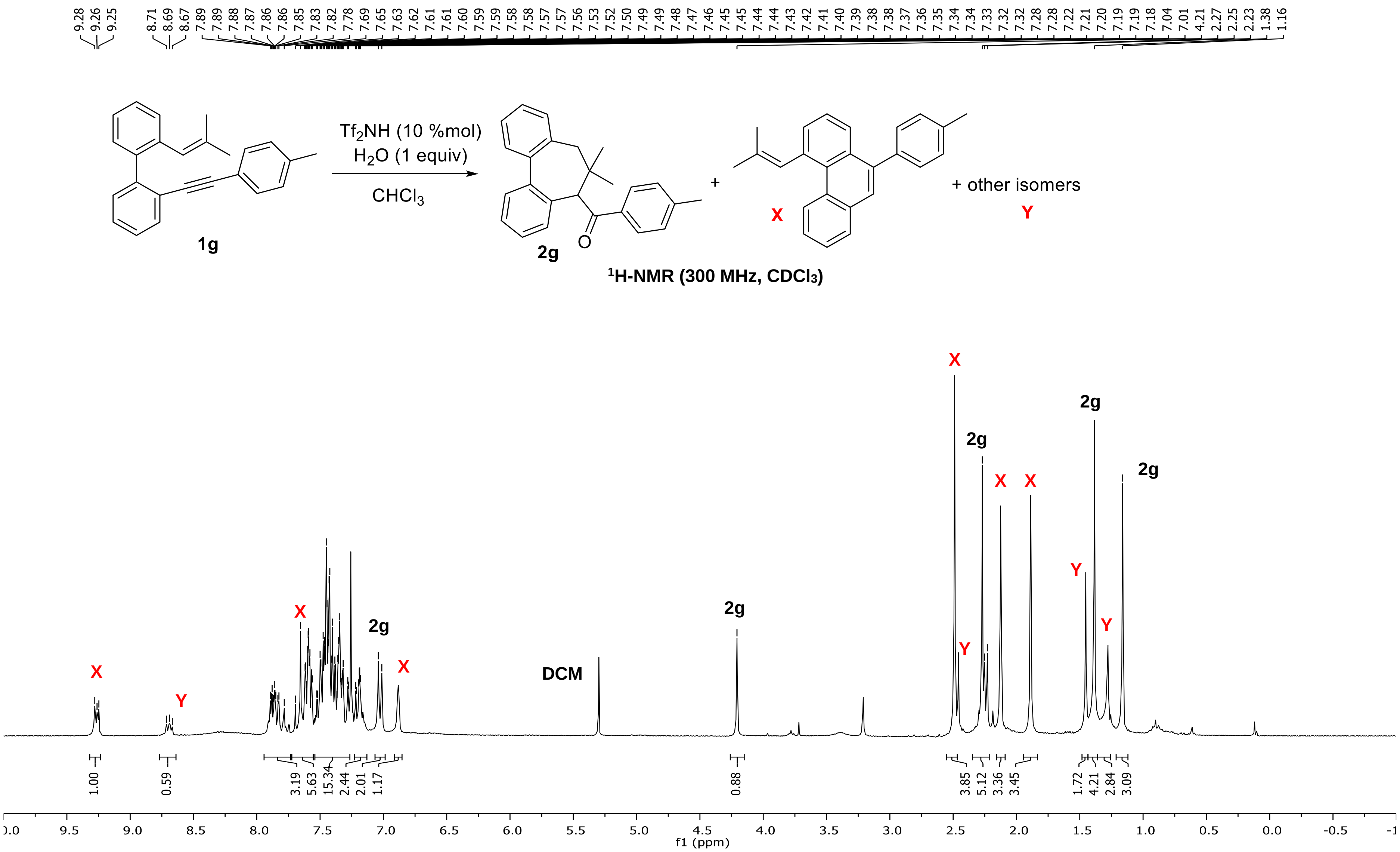
9.28 9.26 9.25 8.71 8.69 8.67 7.89 7.89 7.88 7.87 7.86 7.86 7.85 7.83 7.82 7.78 7.69 7.65 7.63 7.62 7.61 7.61 7.60 7.59 7.59 7.58 7.58 7.57 7.57 7.56 7.53 7.52 7.50 7.49 7.49 7.48 7.47 7.46 7.45 7.45 7.44 7.44 7.43 7.42 7.41 7.40 7.39 7.38 7.38 7.37 7.36 7.35 7.34 7.34 7.33 7.32 7.32 7.28 7.28 7.22 7.21 7.20 7.19 7.19 7.18 7.04 7.01 4.21 2.27 2.25 2.23 1.38 1.16
1g
Tf_2NH (10 %mol)
H_2O (1 equiv)
$CHCl_3$
2g
O
+
X
+ other isomers
Y
^{1}H-NMR (300 MHz, $CDCl_3$)
X
Y
X
2g
X
DCM
2g
X
Y
2g
X
X
Y
2g
Y
2g
1.00 0.59 3.19 5.63 15.34 2.44 2.01 1.17 0.88 3.85 5.12 3.36 3.45 1.72 4.21 2.84 3.09
f1 (ppm)

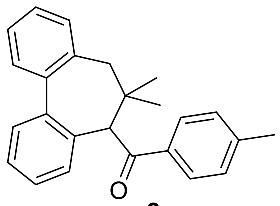

2g

^{1}H-NMR (300 MHz, $CDCl_3$)

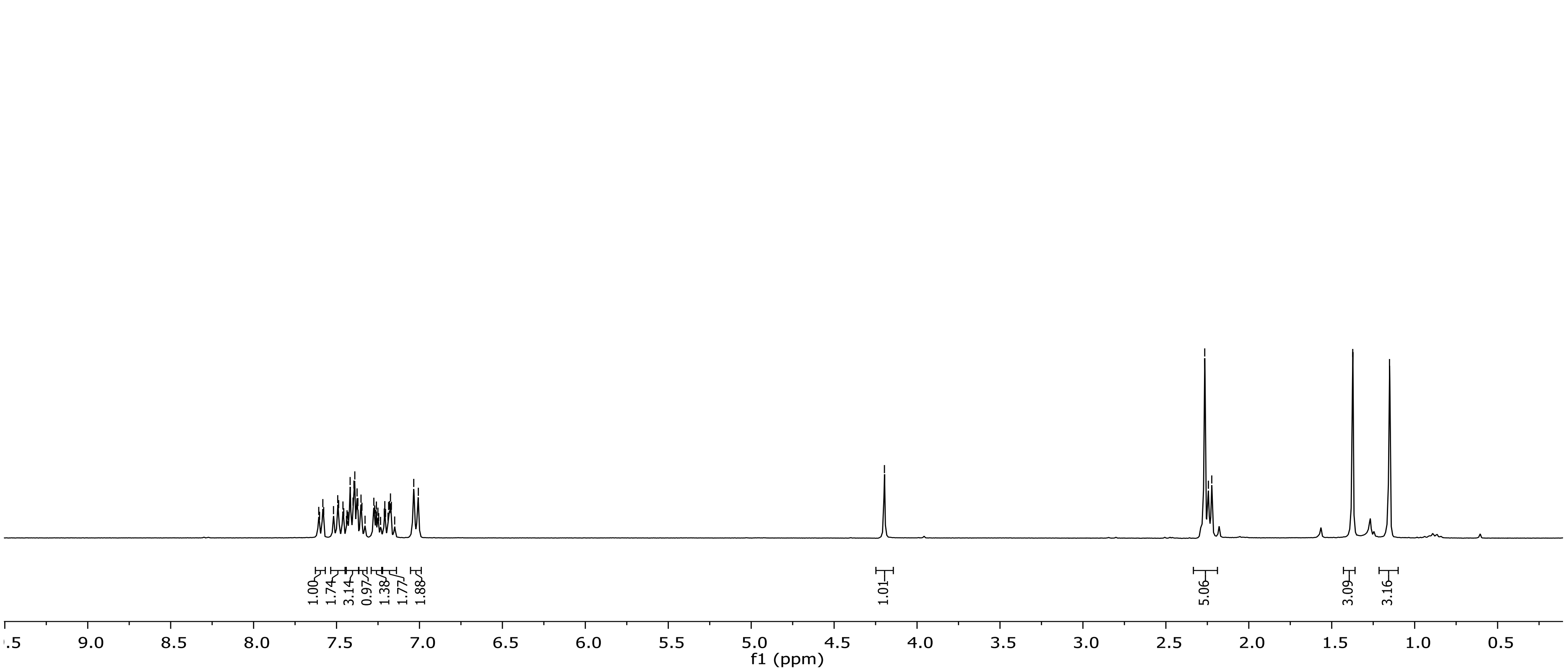

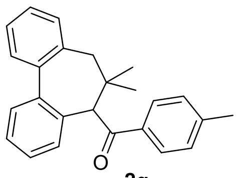

2g

^{13}C-NMR (75 MHz, CDCl$_3$)

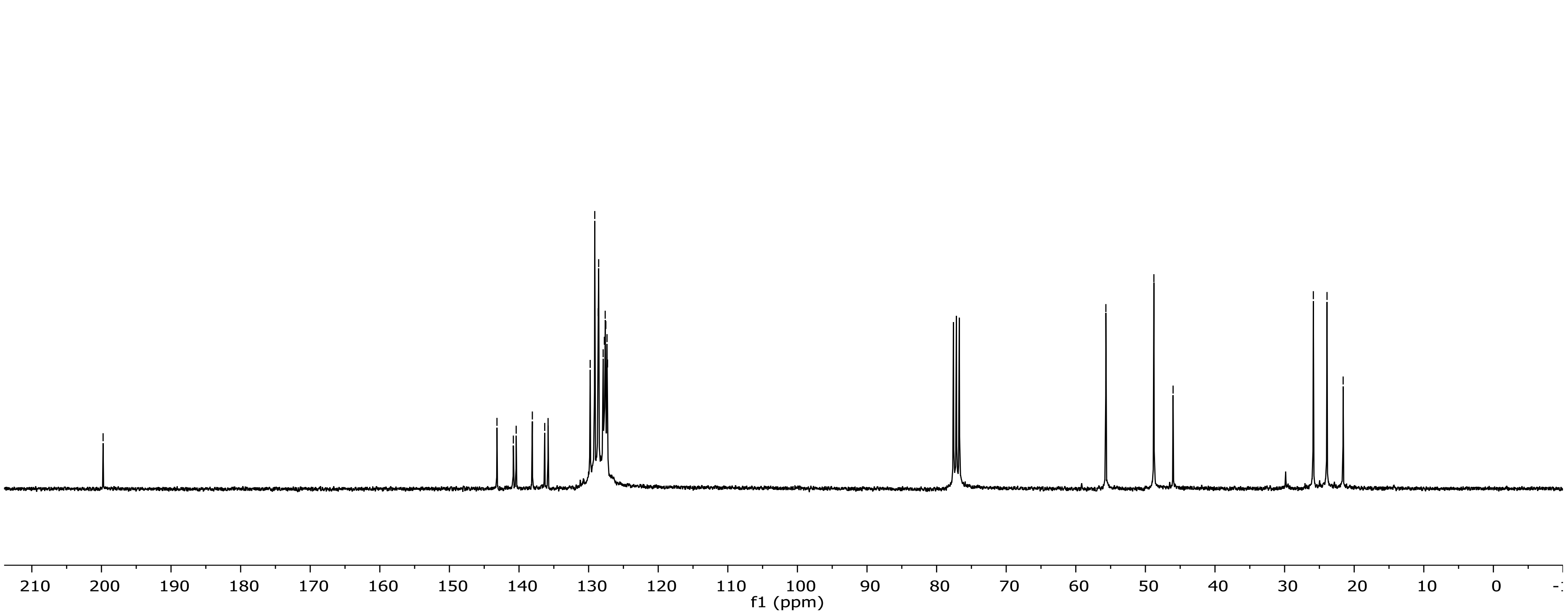

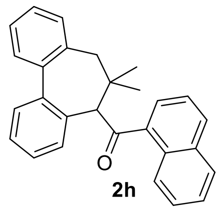

2h

^{1}H-NMR (300 MHz, $CDCl_3$)

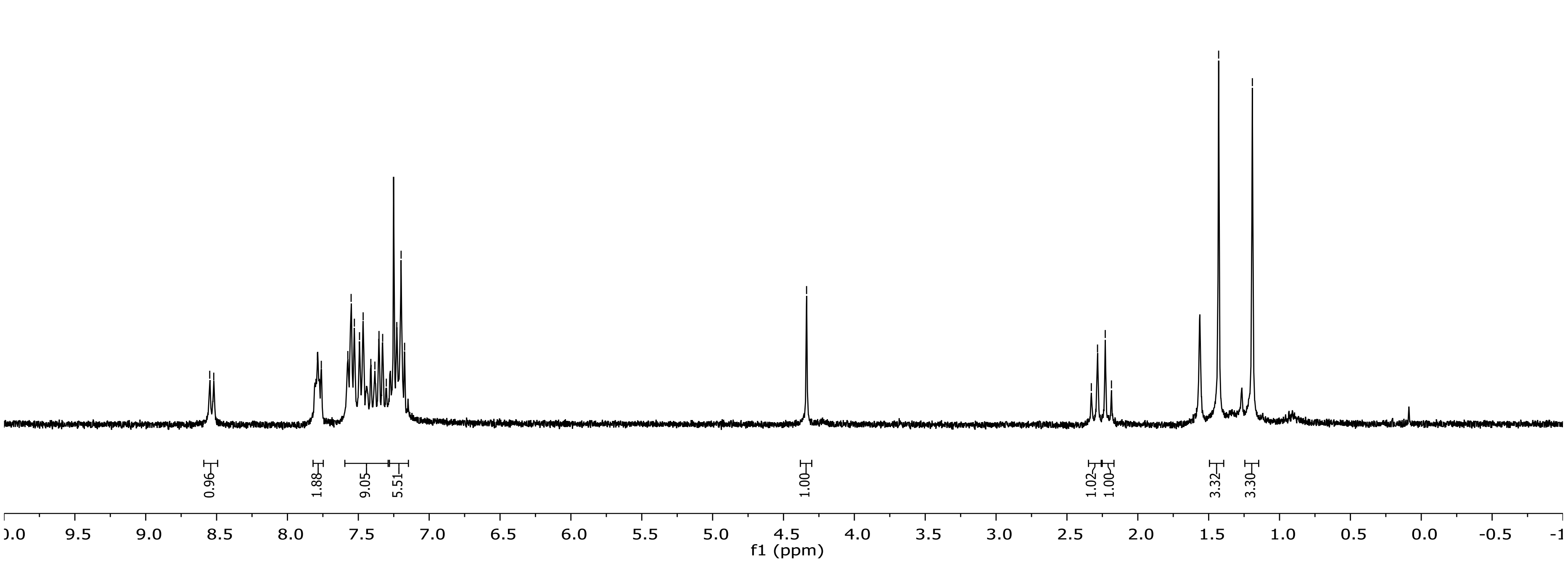

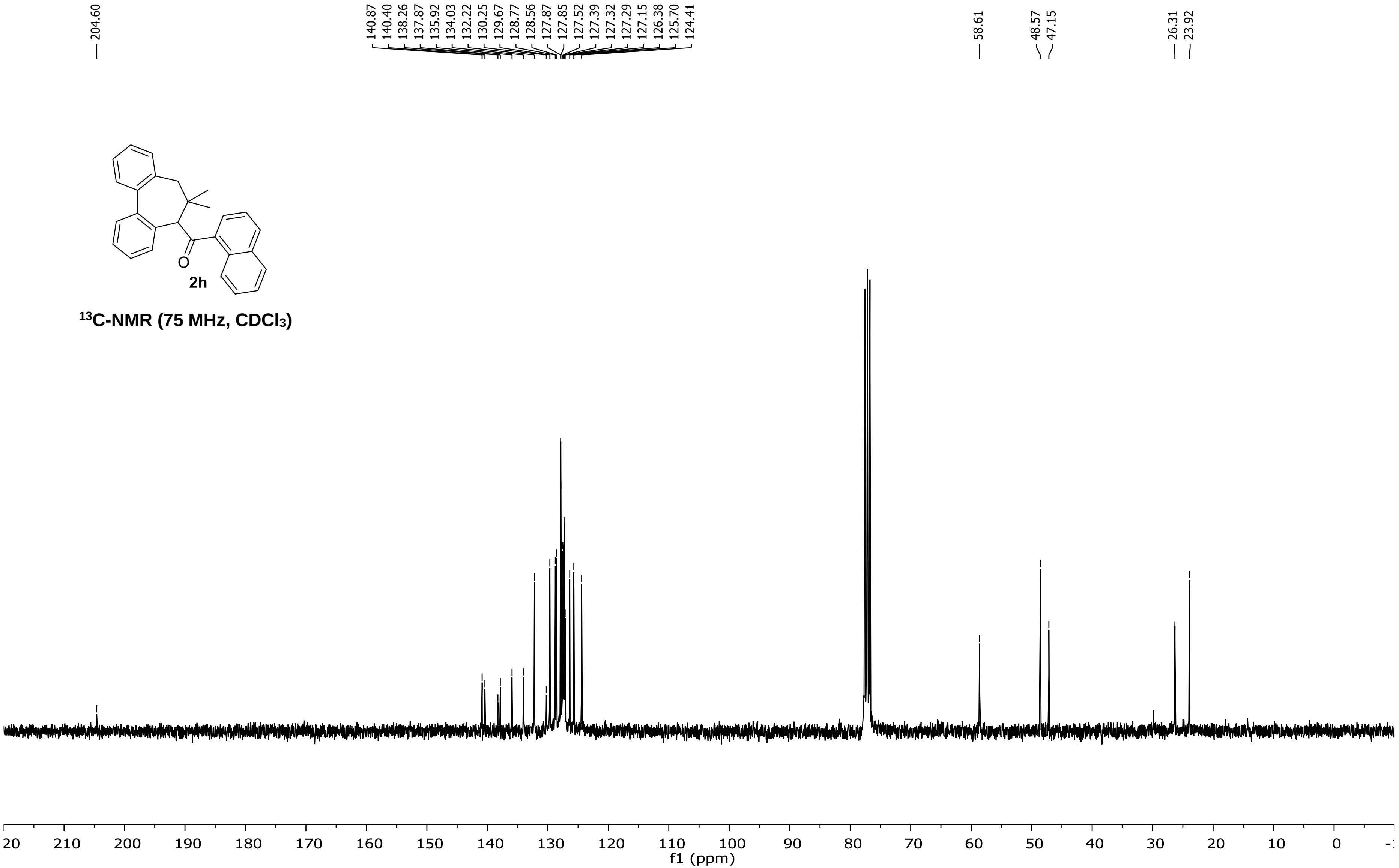

2h

^{13}C-NMR (75 MHz, $CDCl_3$)

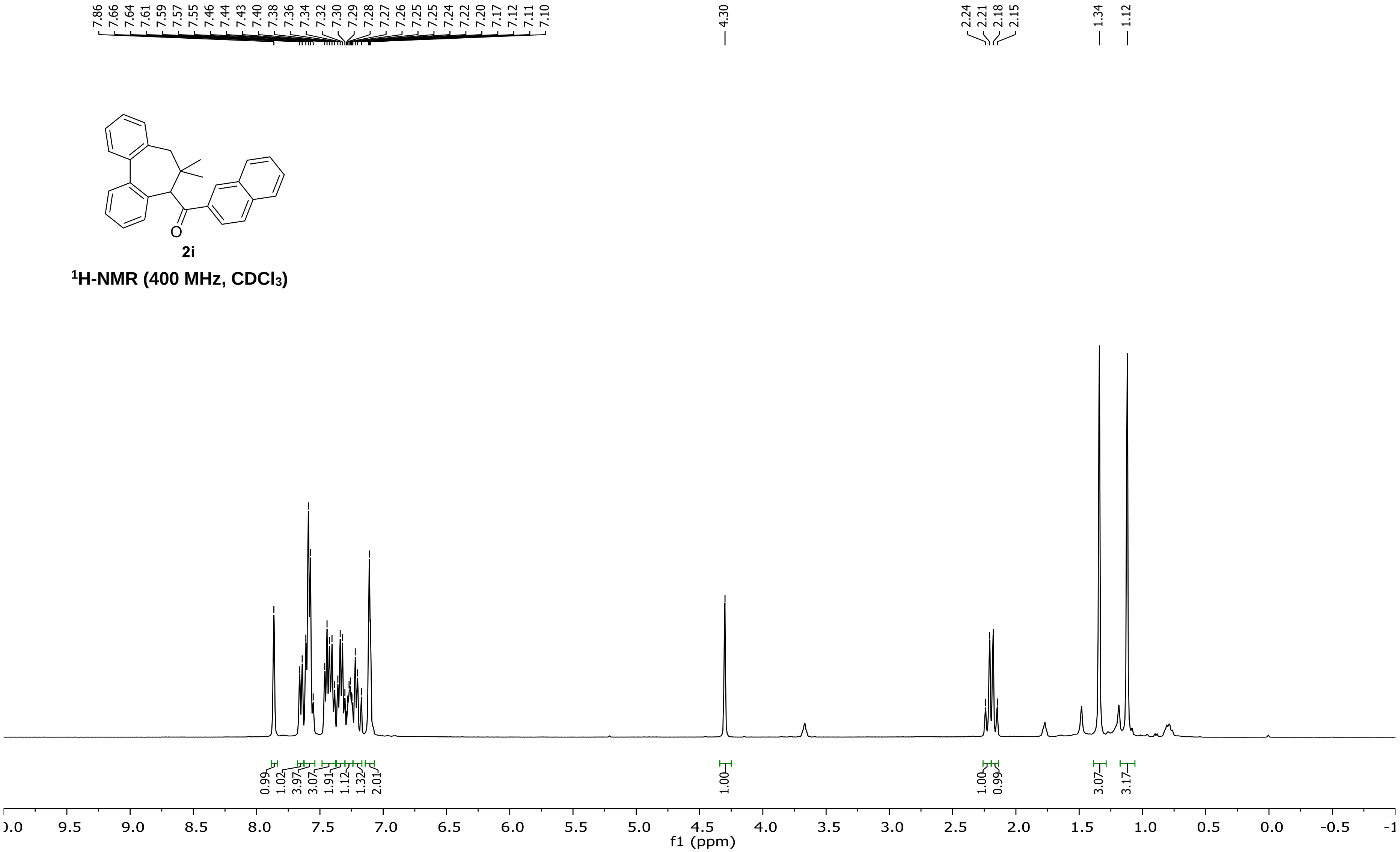

2i

^{1}H-NMR (400 MHz, CDCl$_3$)

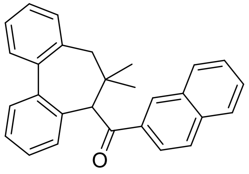

2i

^{13}C-NMR (100 MHz, CDCl$_3$)

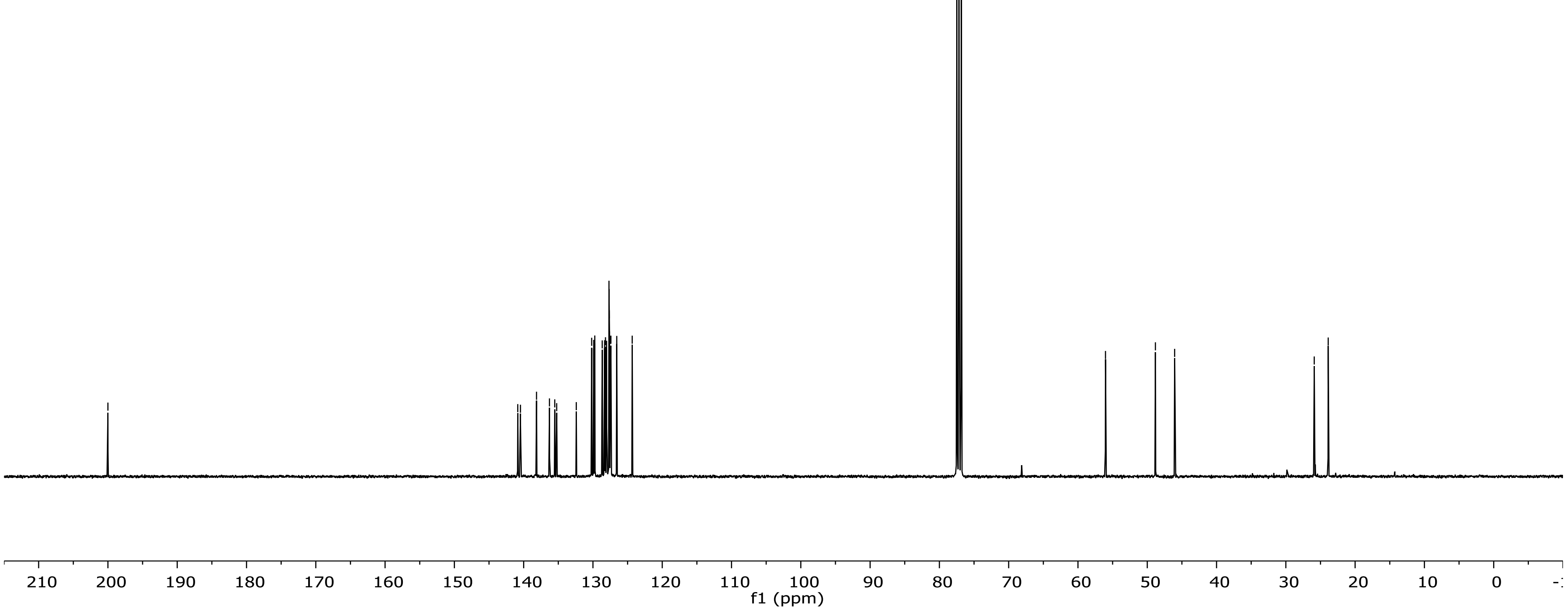

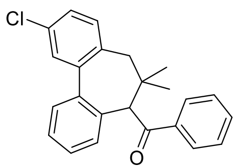

2j

^{1}H-NMR (300 MHz, $CDCl_3$)

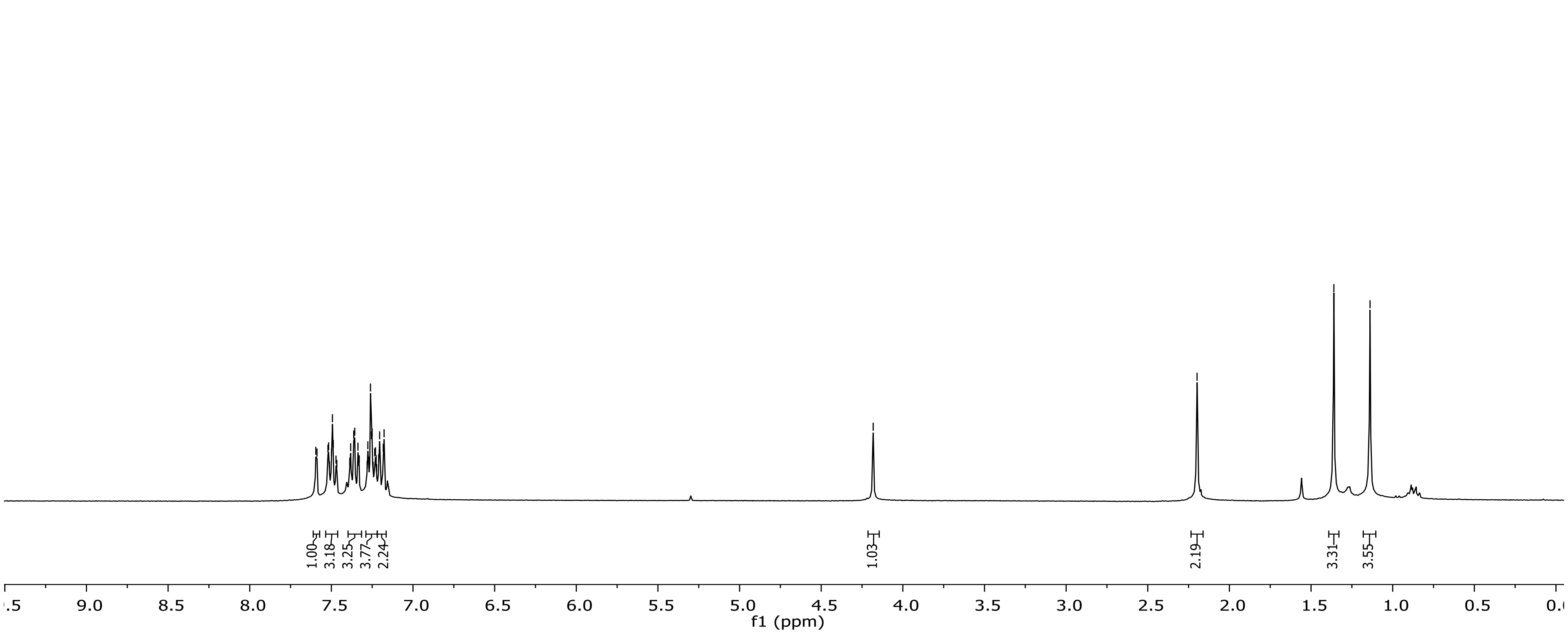

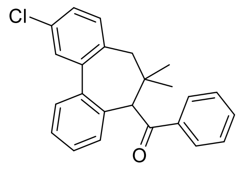

2j

^{13}C-NMR (75 MHz, CDCl$_3$)

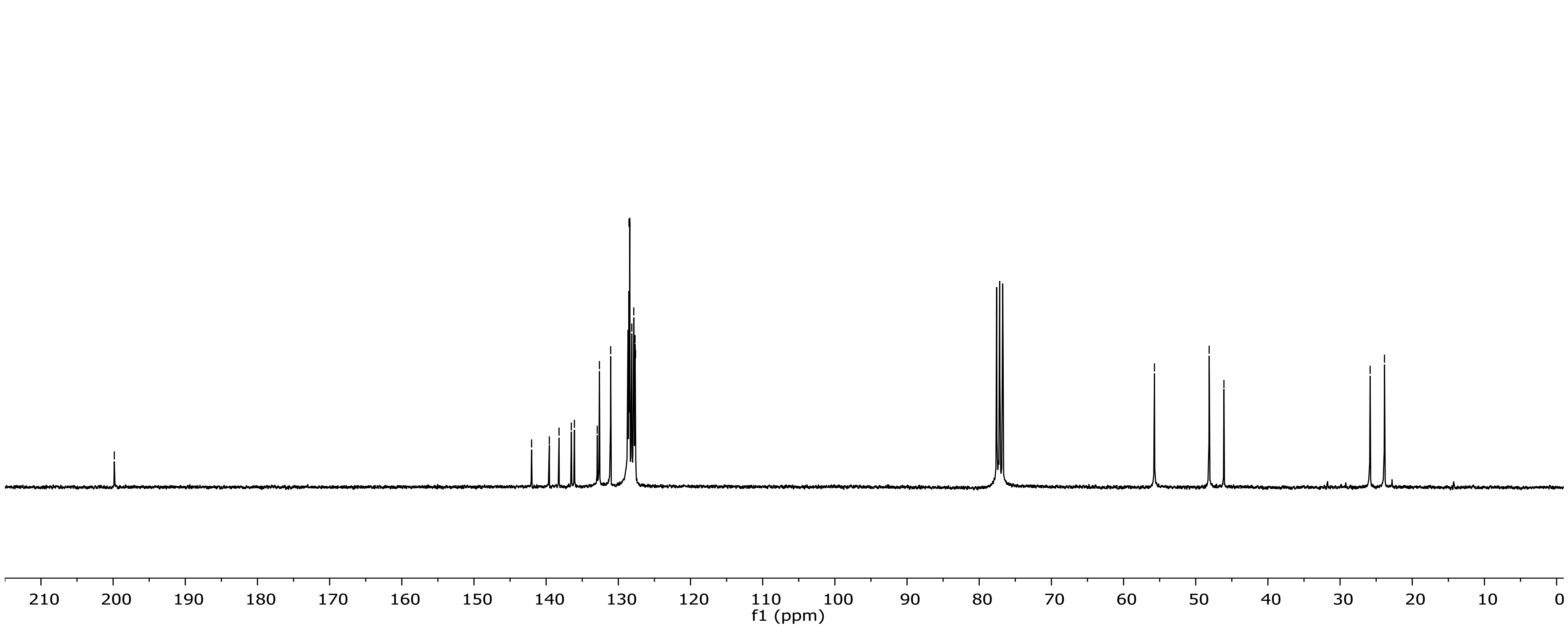

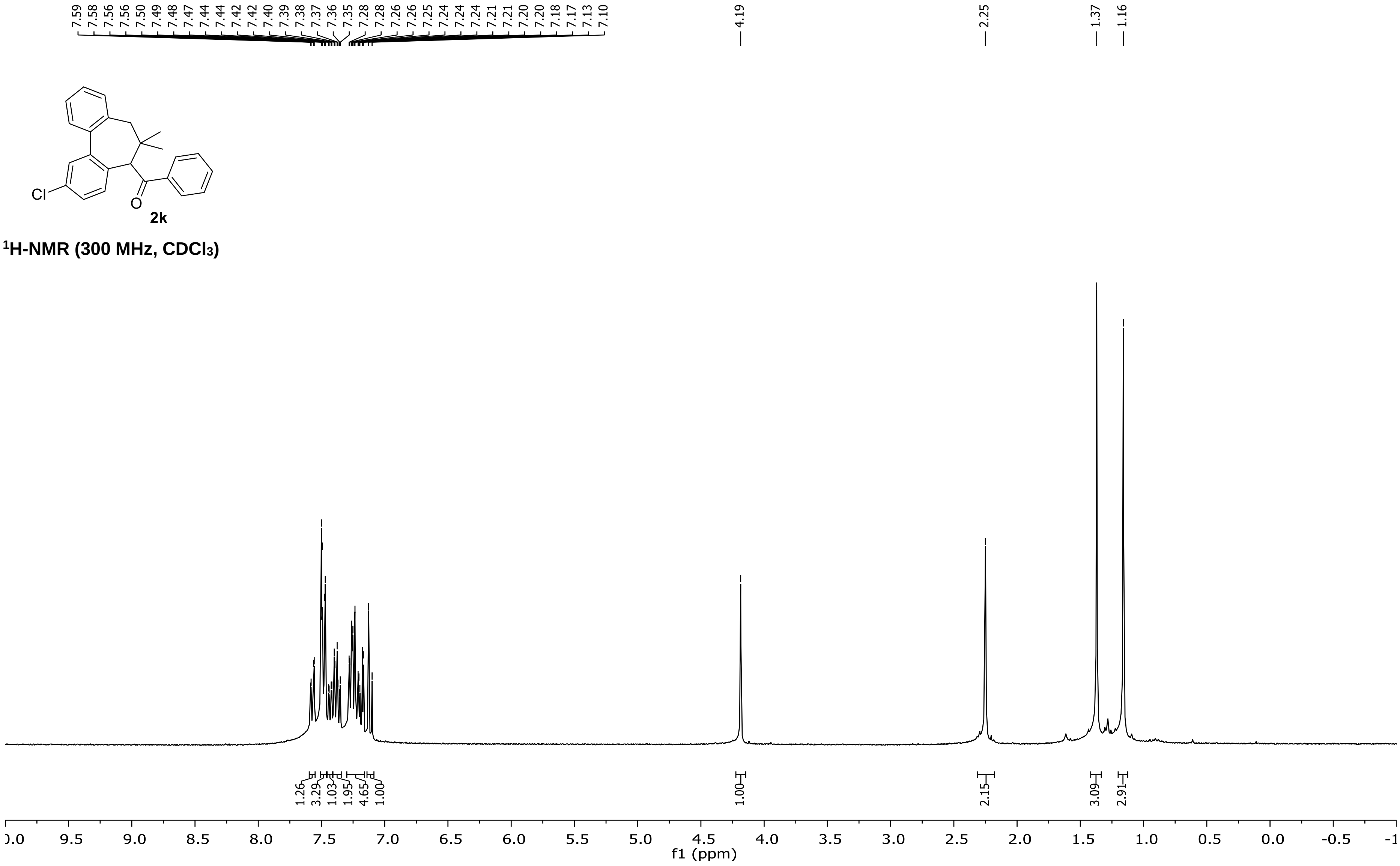
7.59
7.58
7.56
7.56
7.50
7.49
7.48
7.47
7.44
7.44
7.42
7.42
7.40
7.39
7.38
7.37
7.36
7.35
7.28
7.28
7.26
7.26
7.25
7.24
7.24
7.24
7.21
7.21
7.20
7.20
7.18
7.17
7.13
7.10
4.19
2.25
1.37
1.16
Cl
O
2k
1H-NMR (300 MHz, CDCl3)
1.26
3.29
1.03
1.95
4.65
1.00
1.00
2.15
3.09
2.91
9.5
9.0
8.5
8.0
7.5
7.0
6.5
6.0
5.5
5.0
4.5
4.0
3.5
3.0
2.5
2.0
1.5
1.0
0.5
0.0
-0.5
f1 (ppm)

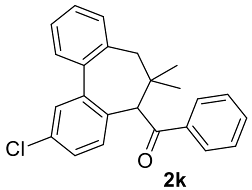

2k

^{13}C-NMR (75 MHz, $CDCl_3$)

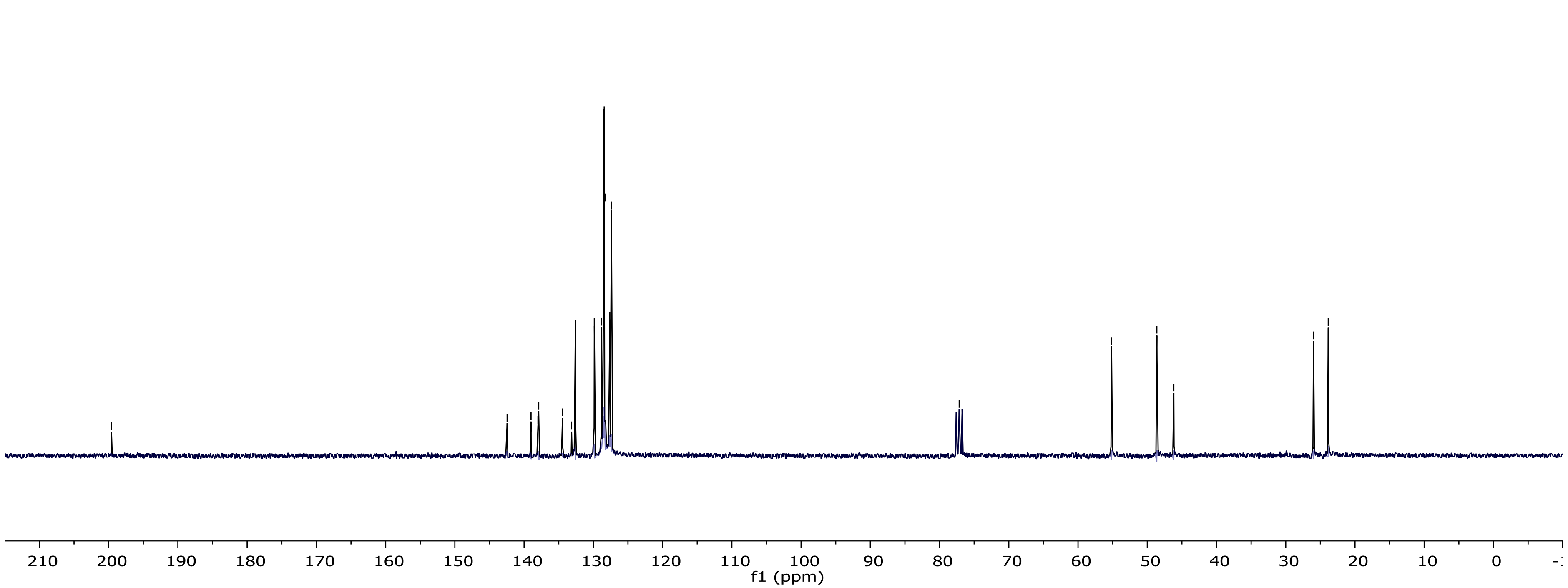

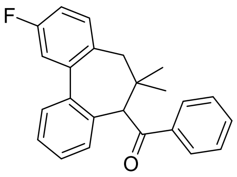

2l

^{1}H-NMR (300 MHz, $CDCl_3$)

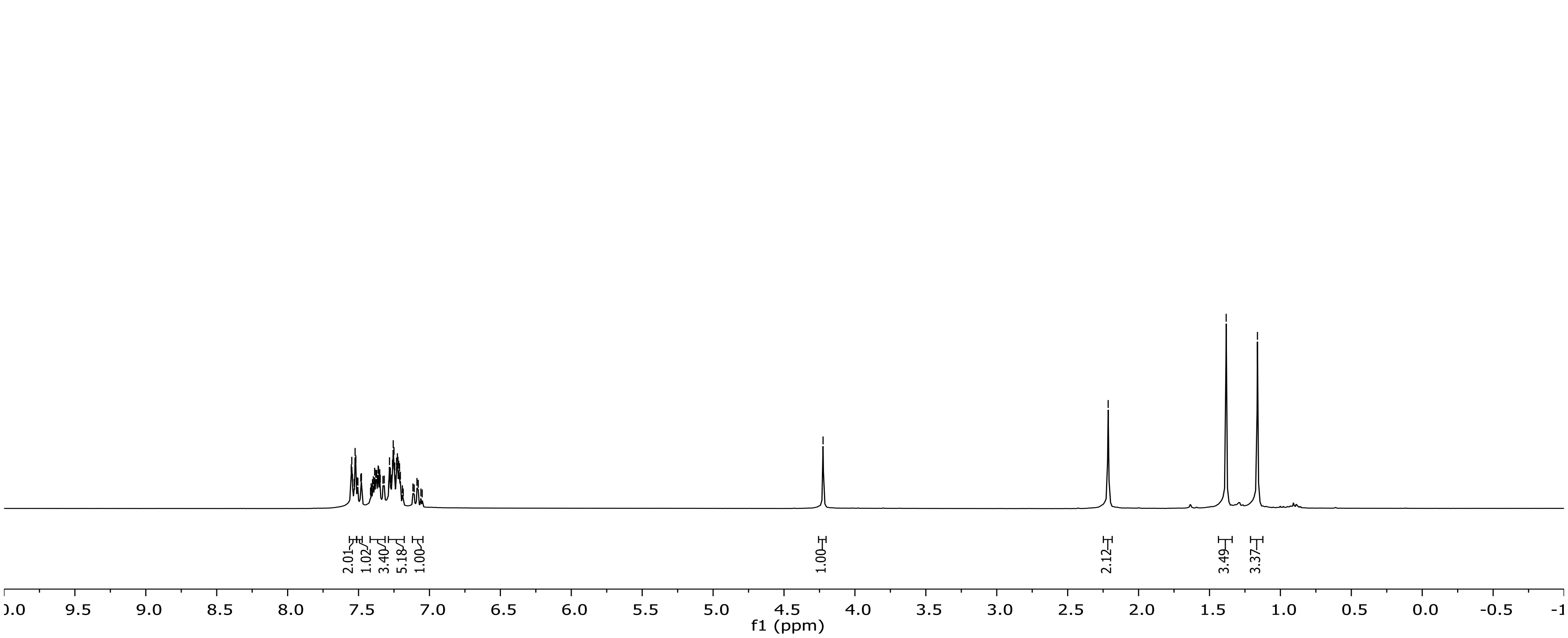

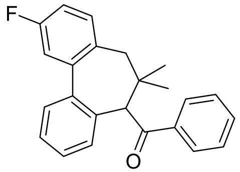

2l

^{13}C-NMR (75 MHz, $CDCl_3$)

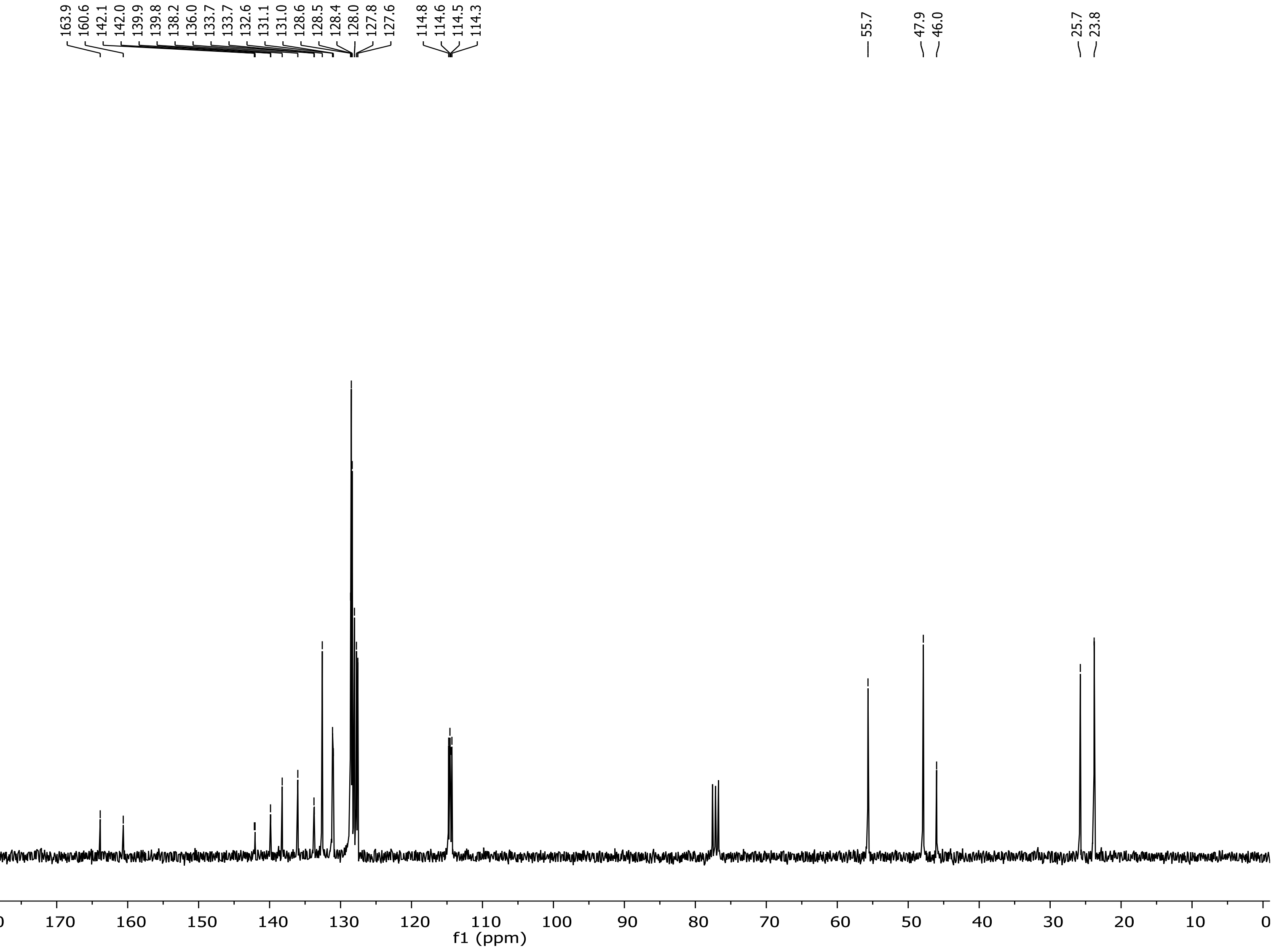

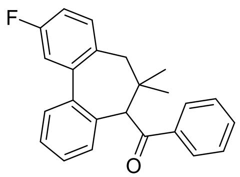

2l

^{19}F-NMR (283 MHz, CDCl$_3$)

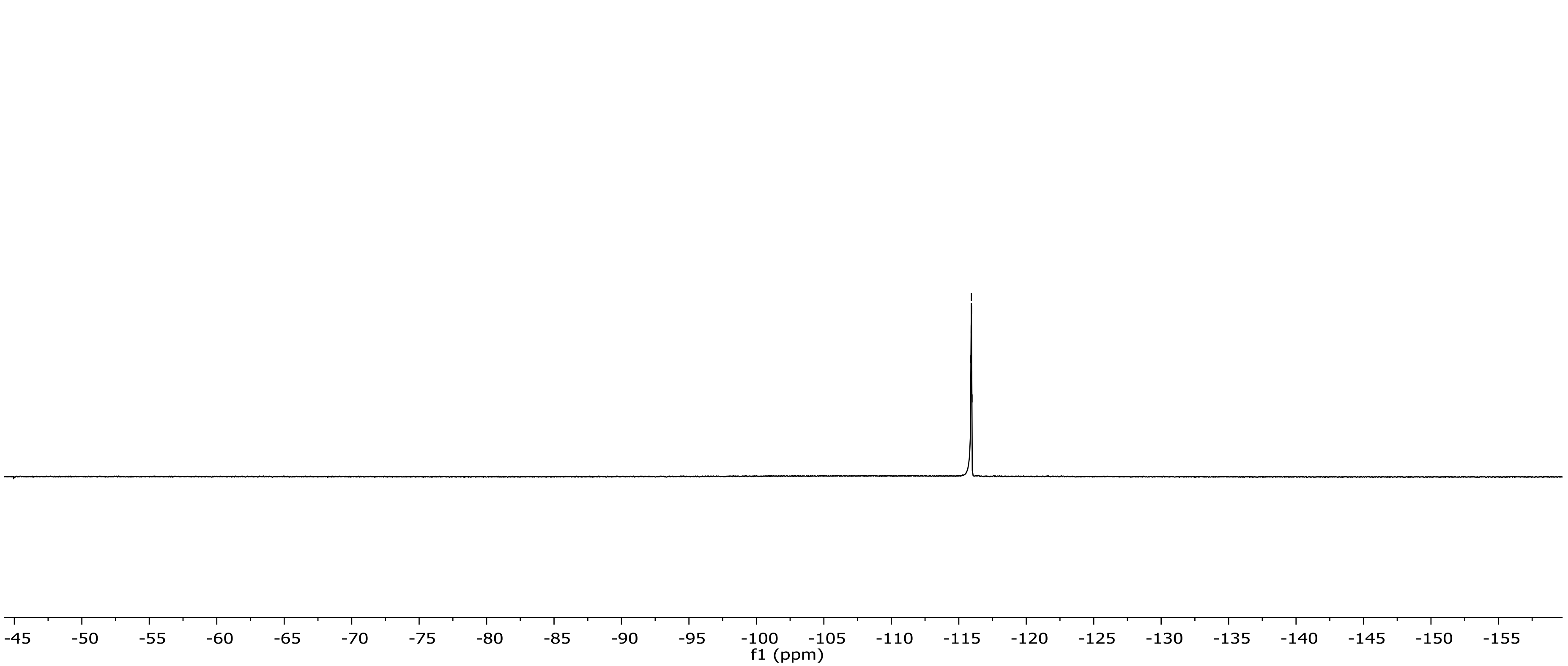

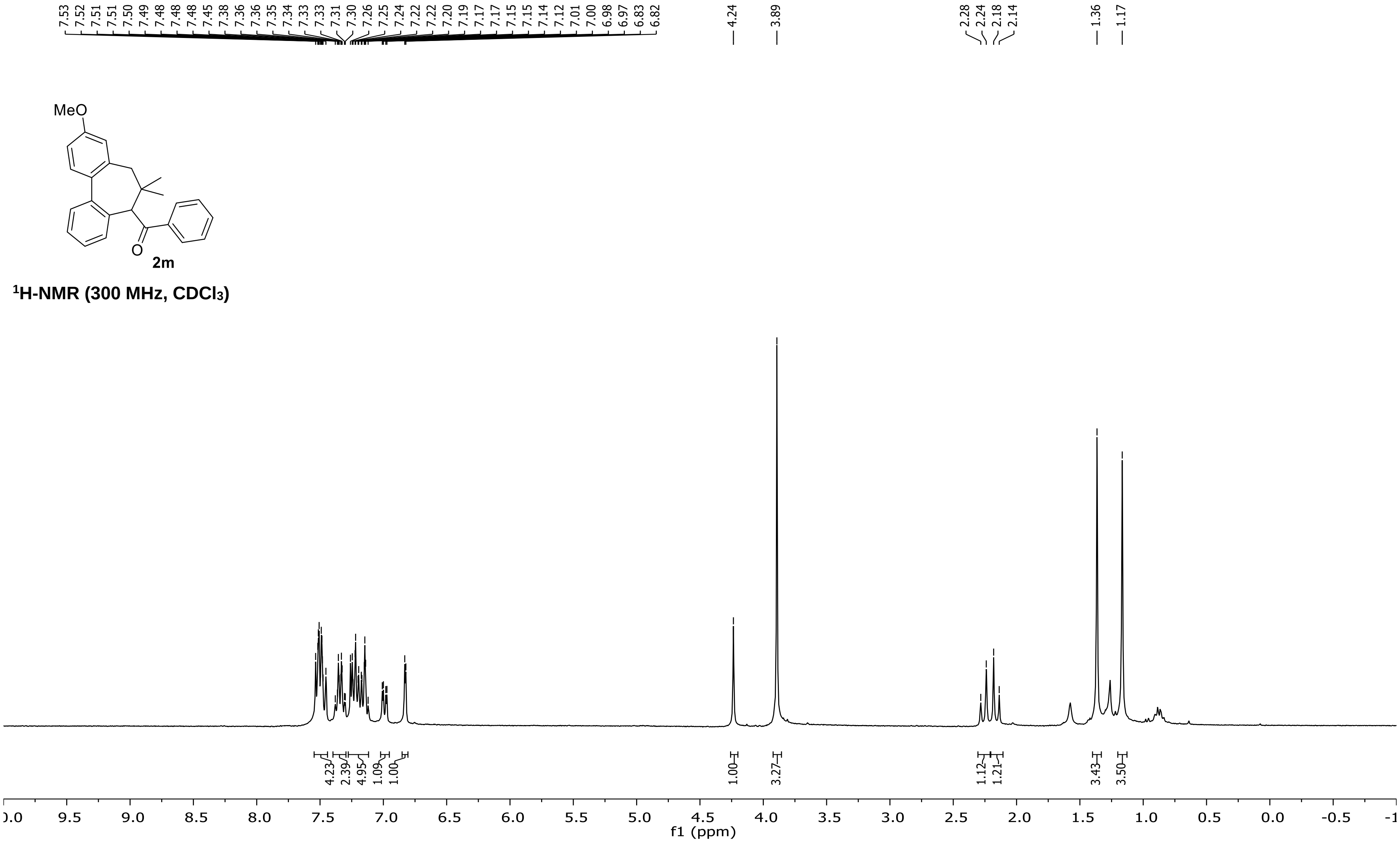

7.53
7.52
7.51
7.51
7.50
7.49
7.48
7.48
7.45
7.38
7.36
7.36
7.35
7.34
7.33
7.33
7.31
7.30
7.26
7.25
7.24
7.22
7.22
7.20
7.19
7.17
7.17
7.15
7.15
7.14
7.12
7.01
7.00
6.98
6.97
6.83
6.82
4.24
3.89
2.28
2.24
2.18
2.14
1.36
1.17
MeO
O
2m
1H-NMR (300 MHz, CDCl3)
4.23
2.39
4.95
1.09
1.00
1.00
3.27
1.12
1.21
3.43
3.50
f1 (ppm)

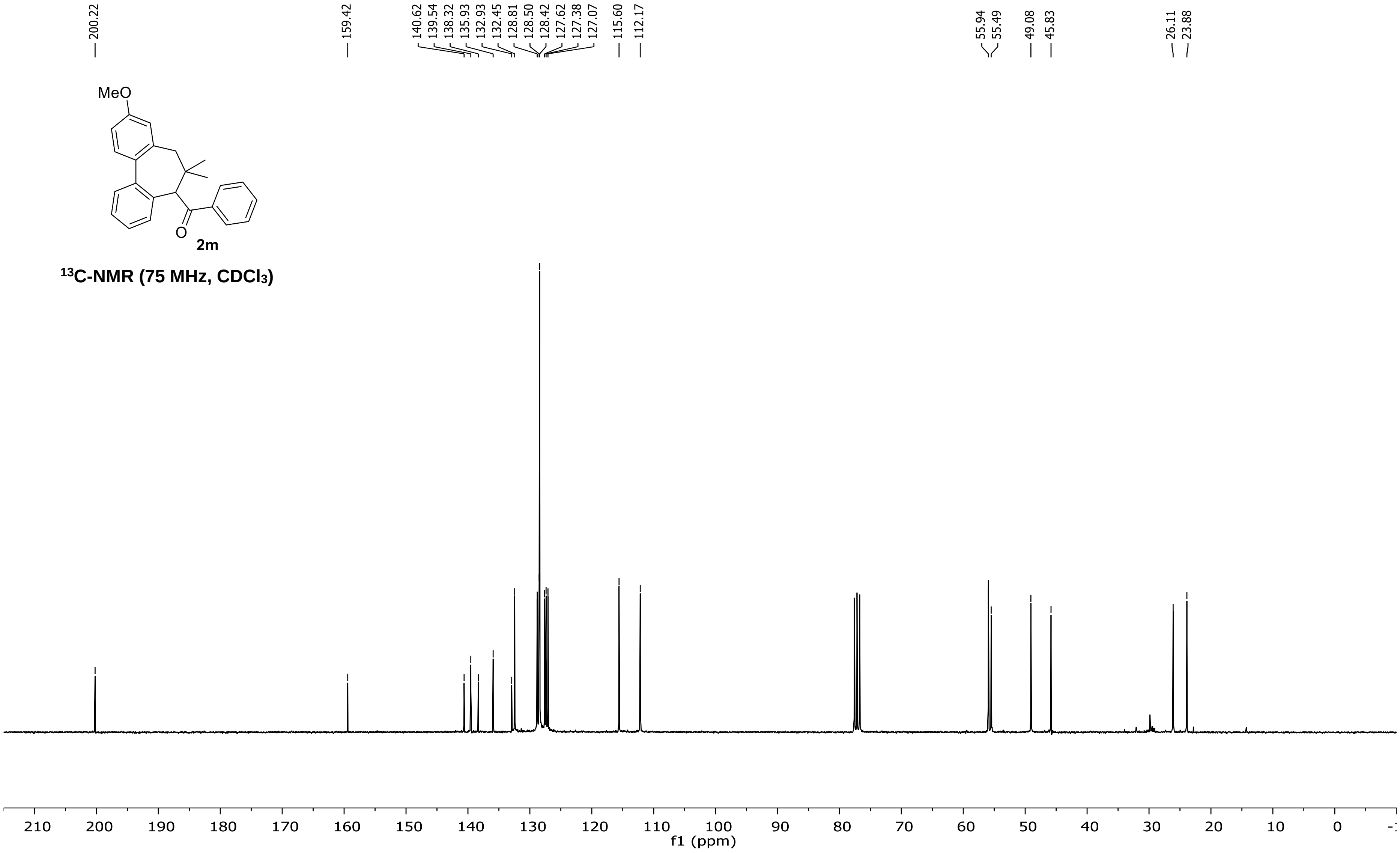

2m

^{13}C-NMR (75 MHz, $CDCl_3$)

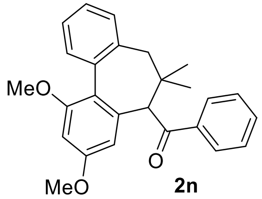

2n

¹H-NMR (300 MHz, CDCl₃)

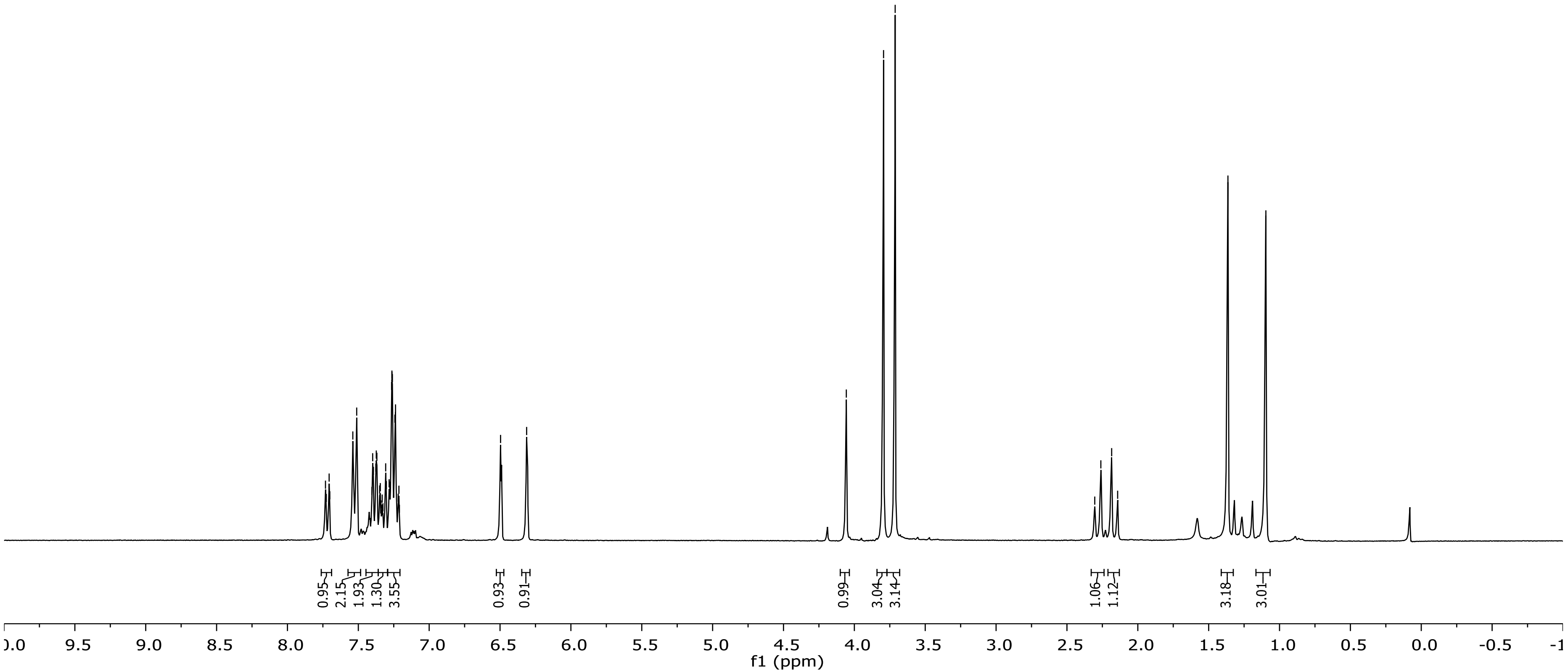

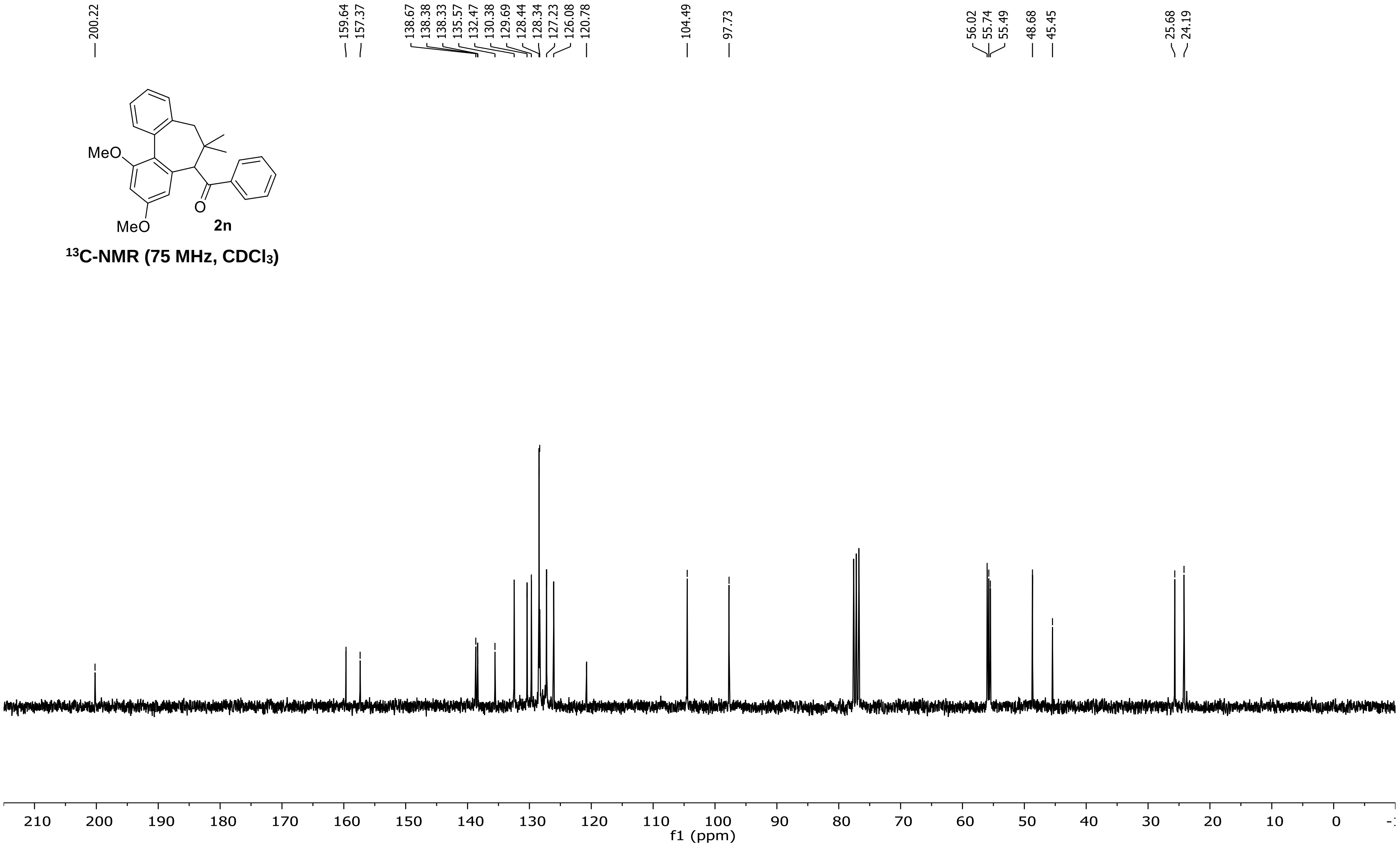

^{13}C-NMR (75 MHz, CDCl$_3$)

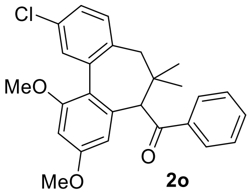

2o

^{1}H-NMR (500 MHz, CDCl$_3$)

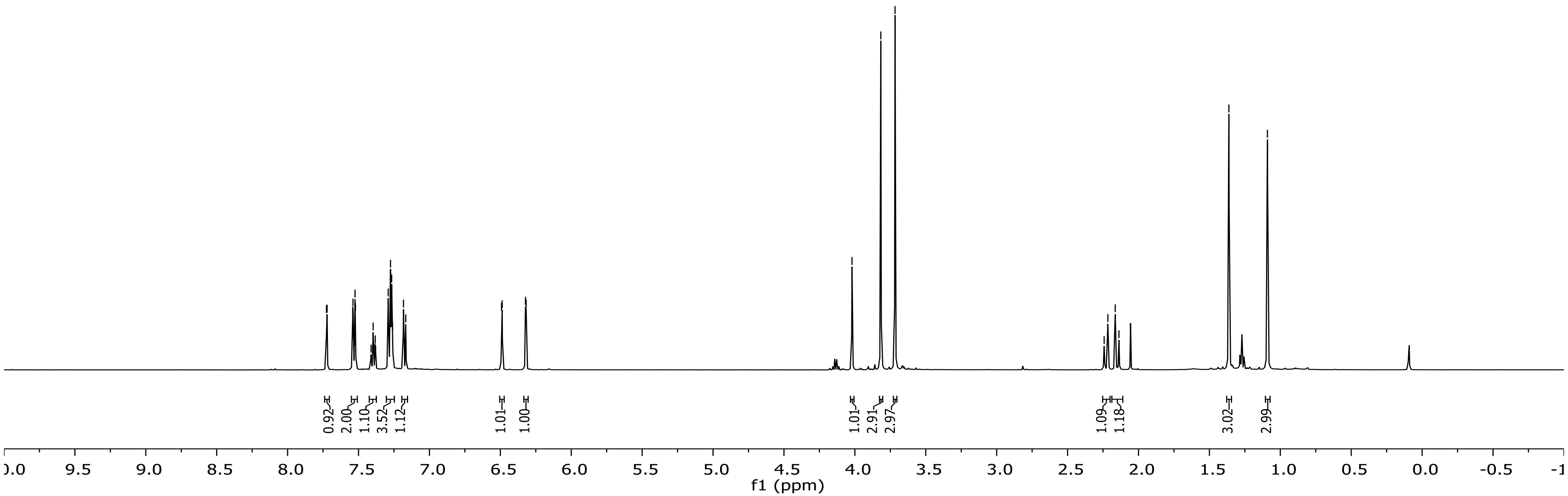

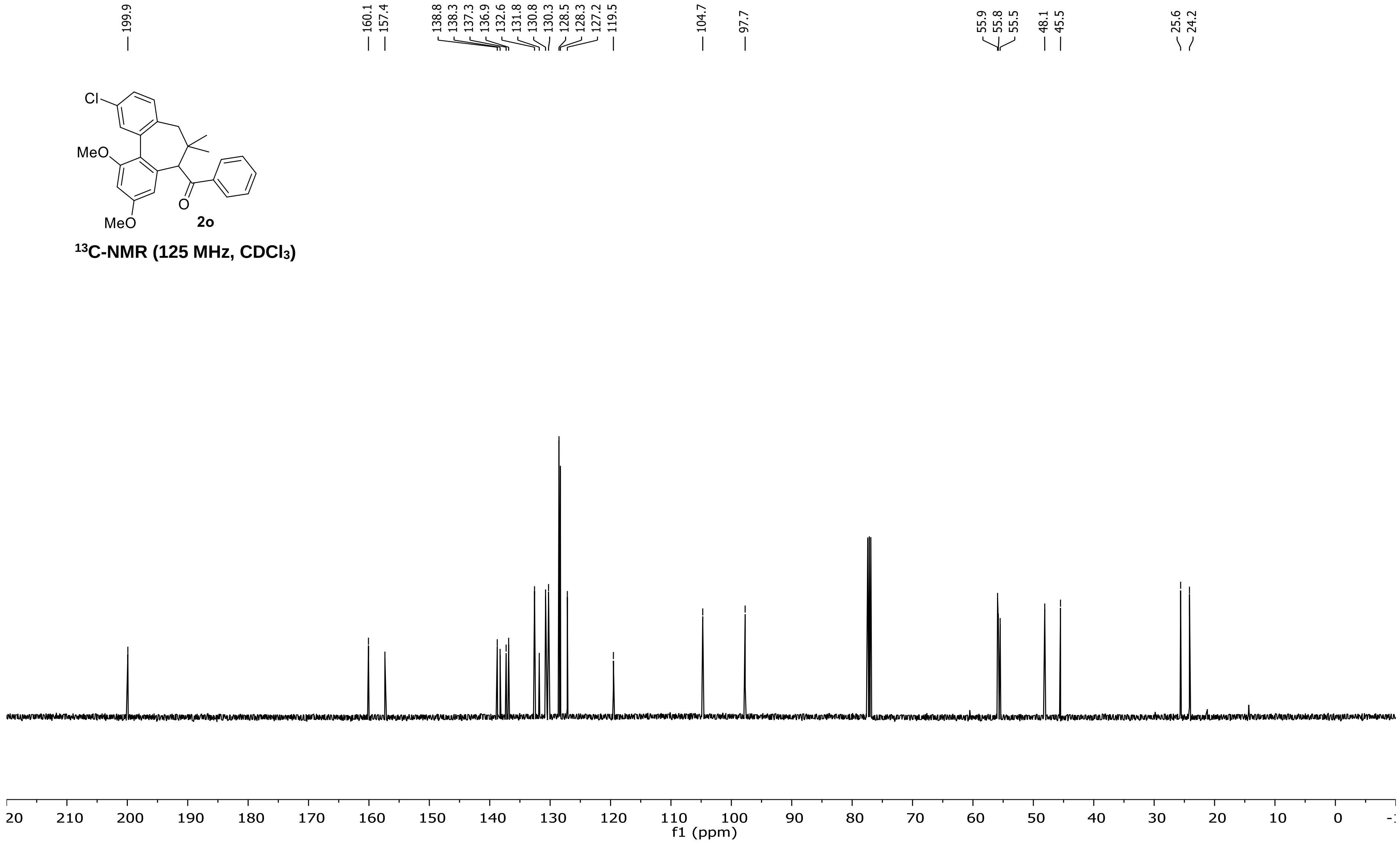

2o

^{13}C-NMR (125 MHz, $CDCl_3$)

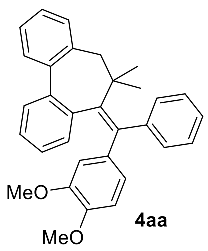

^{1}H-NMR (500 MHz, $CDCl_3$)

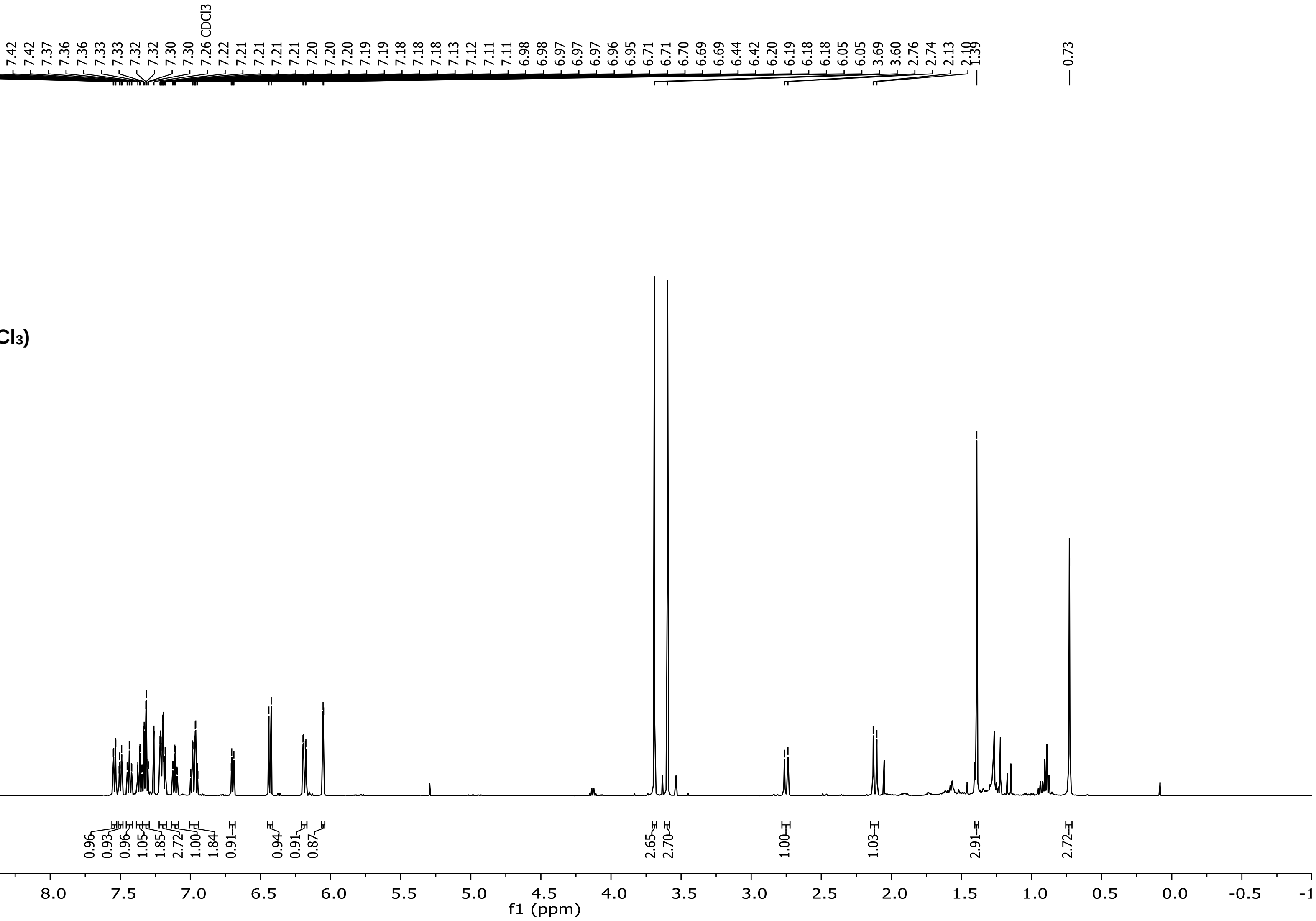

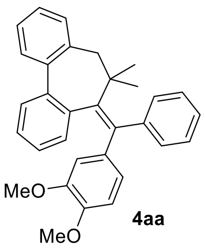

4aa

^{13}C-NMR (75 MHz, $CDCl_3$)

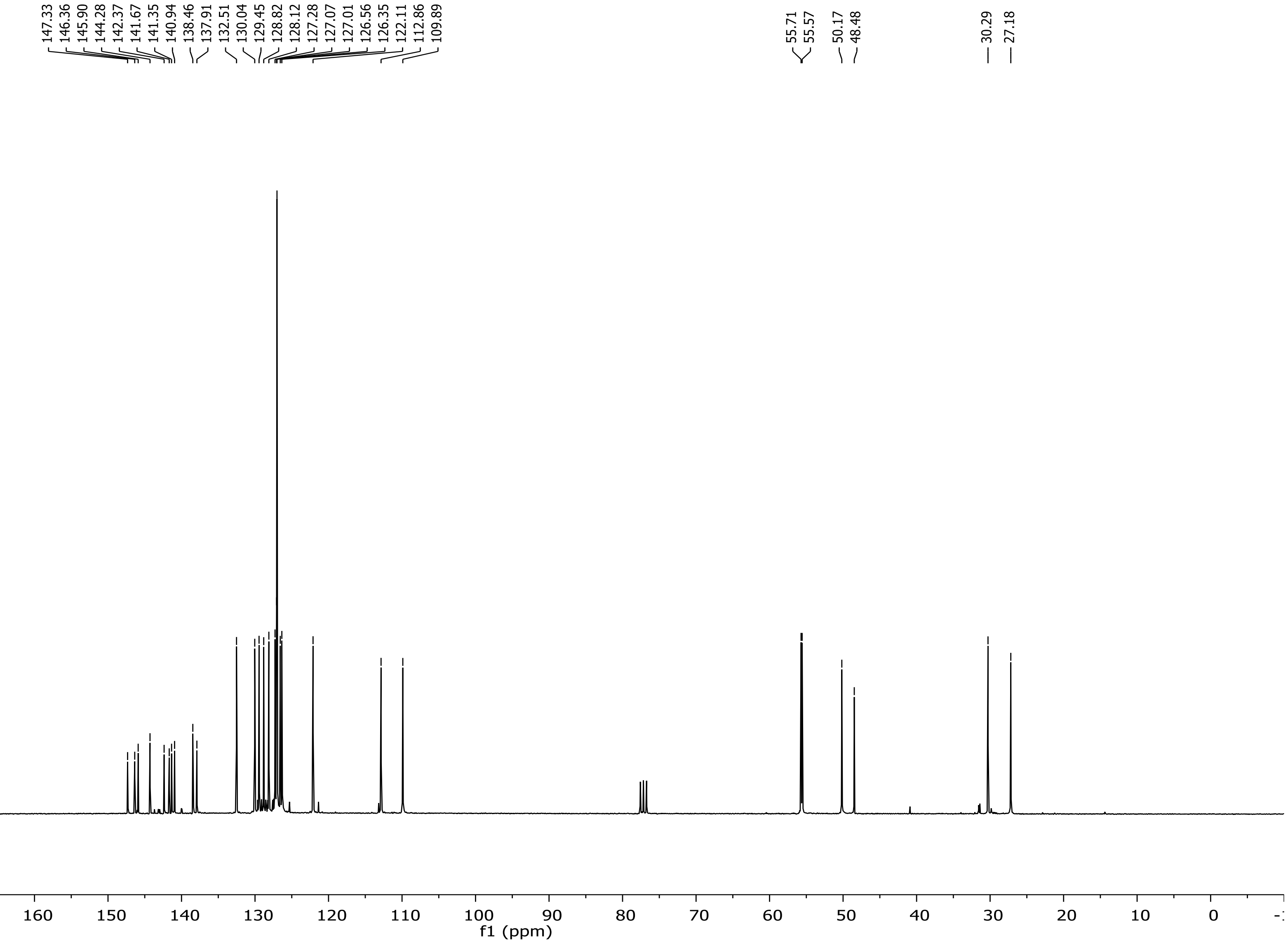

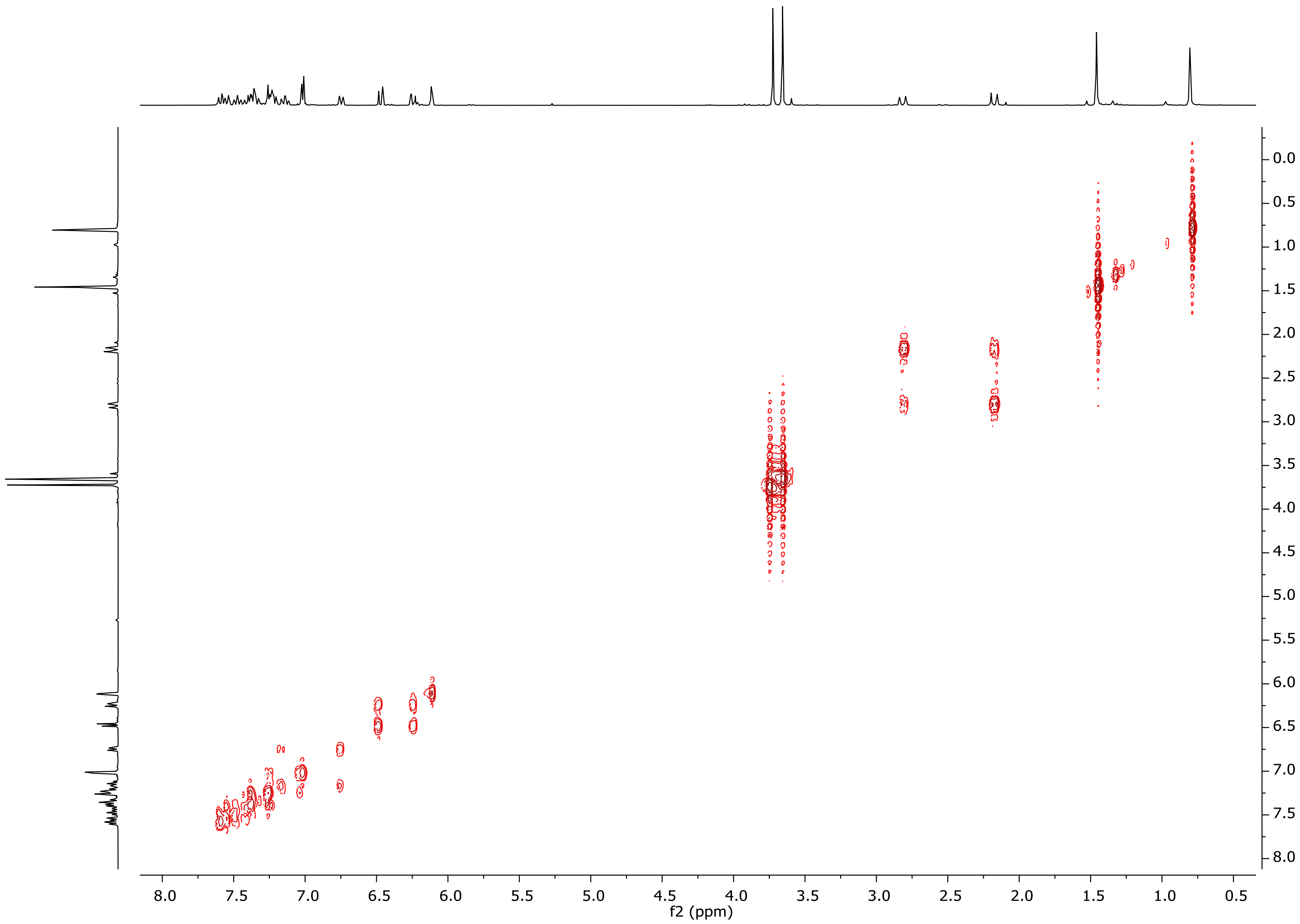

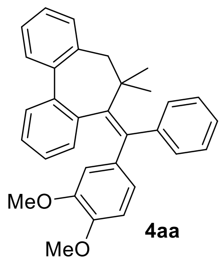

4aa

gCOSY-NMR

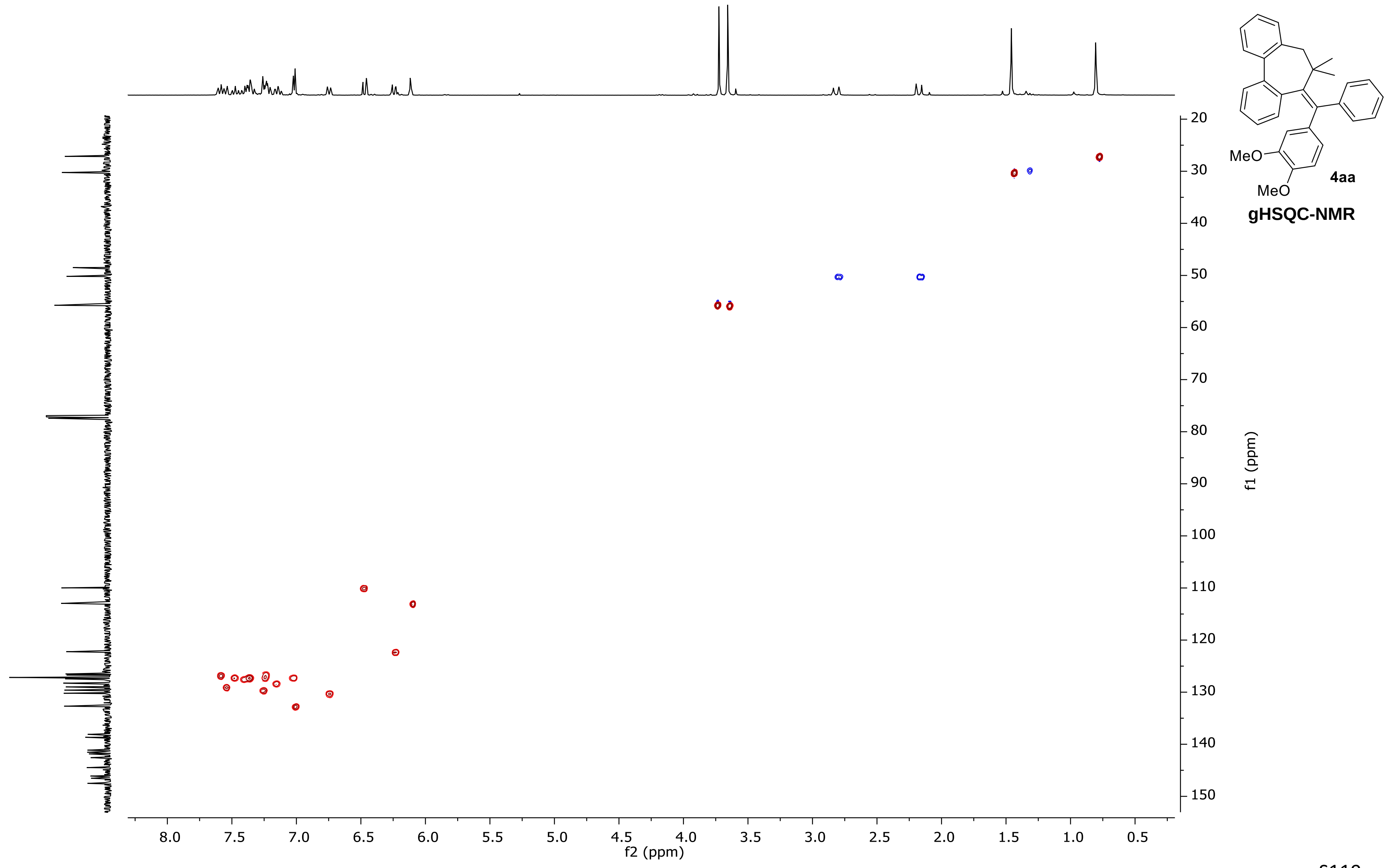
MeO
MeO
4aa
gHSQC-NMR
f1 (ppm)
f2 (ppm)

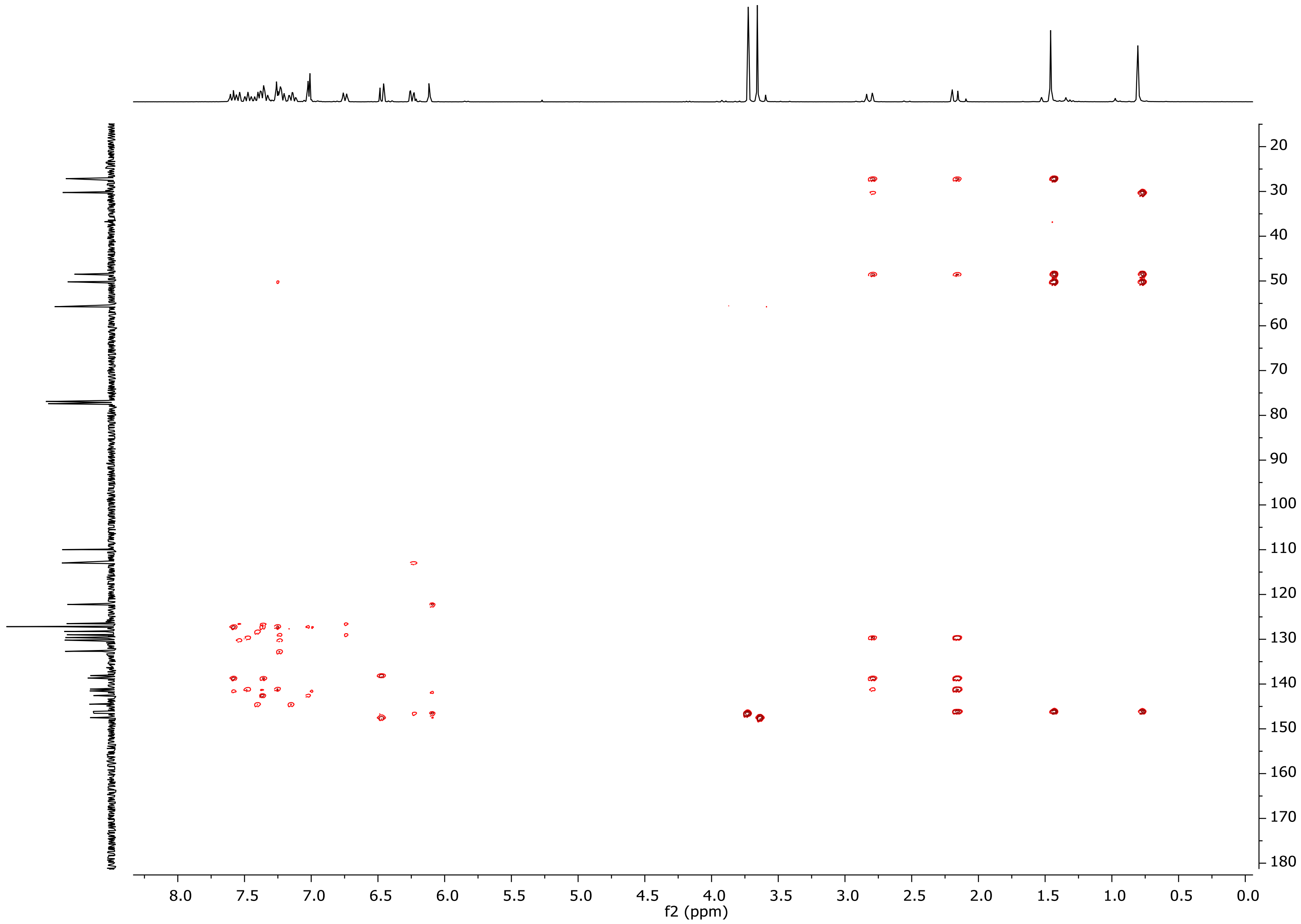

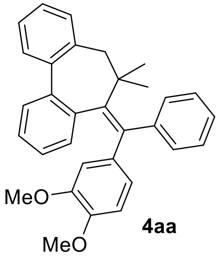

4aa

gHMBC-NMR

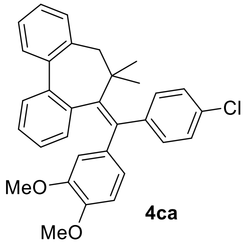

4ca

^{1}H-NMR (300 MHz, $CDCl_3$)

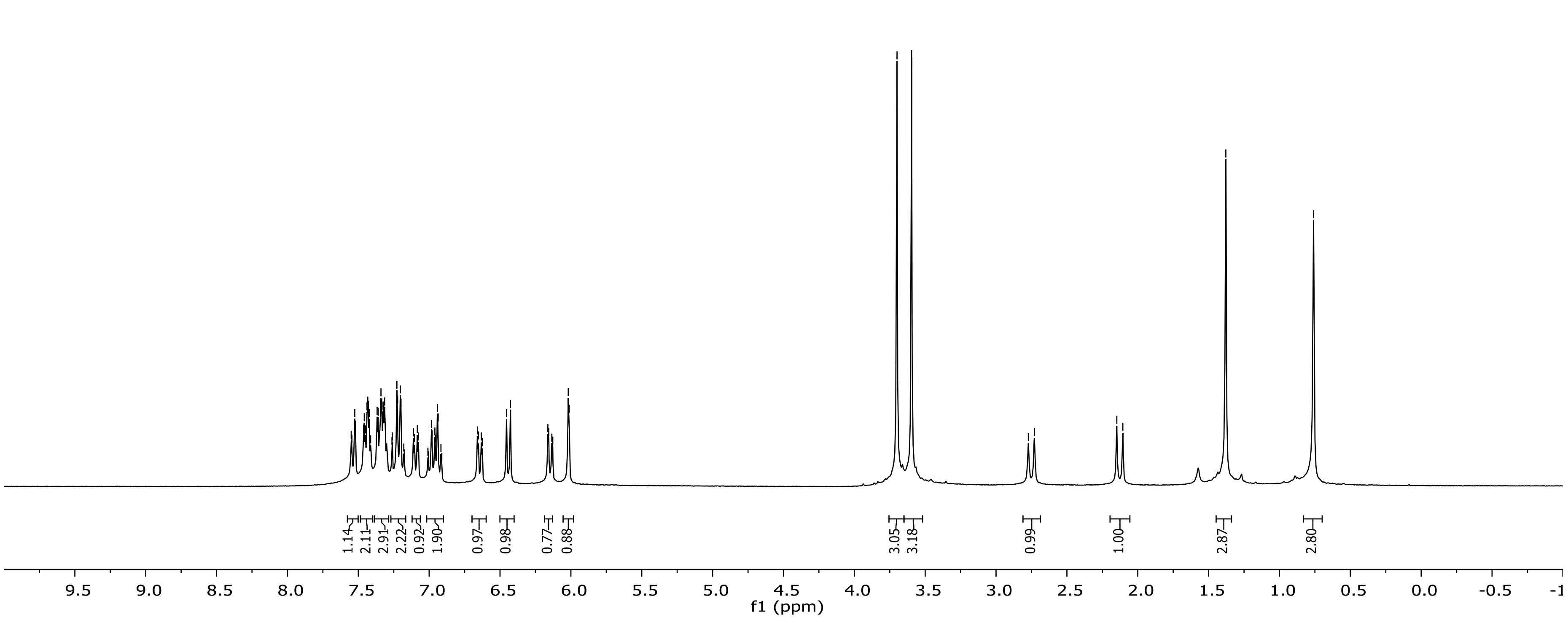

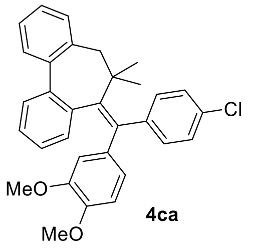

4ca

^{13}C-NMR (75 MHz, $CDCl_3$)

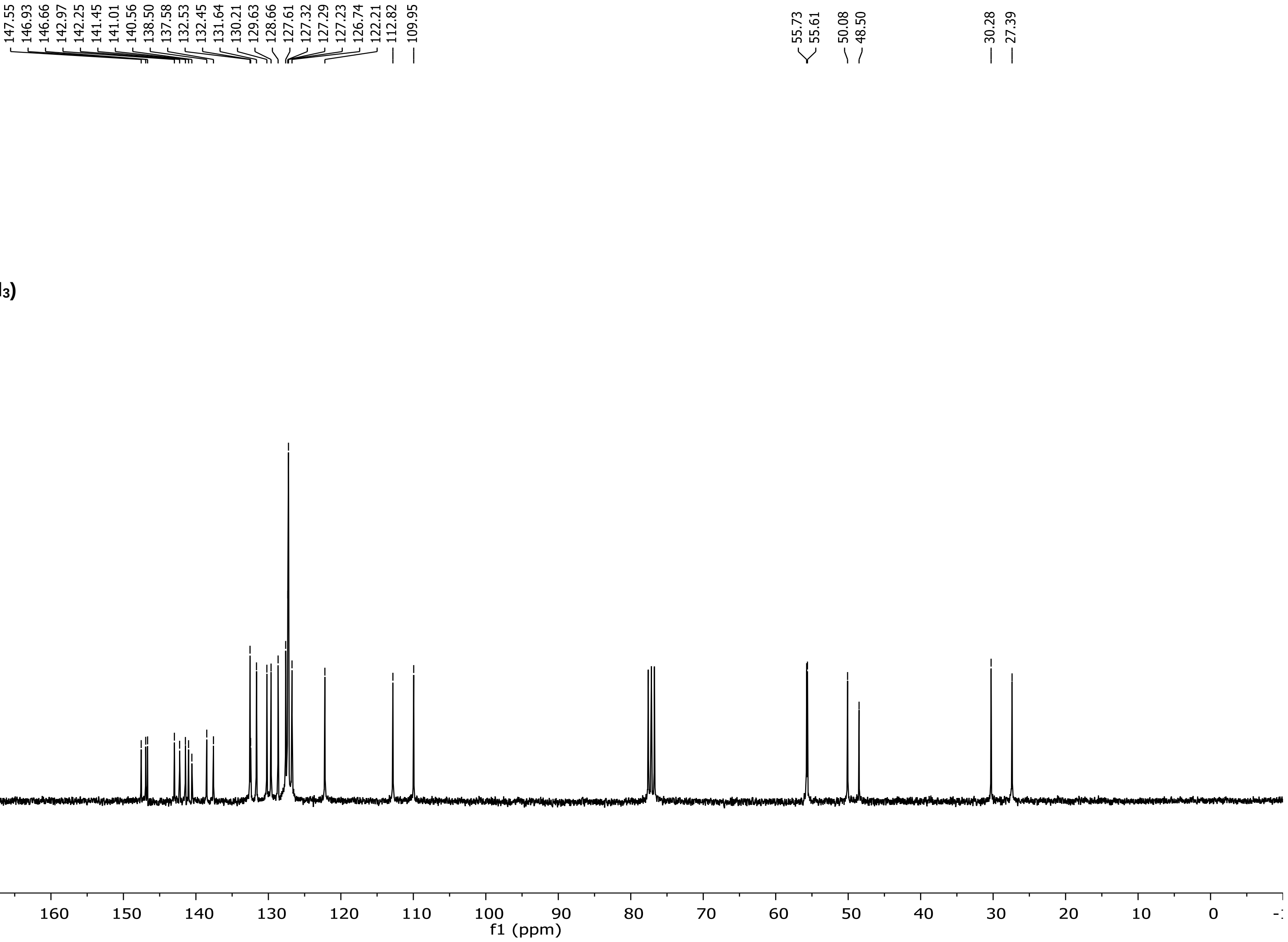

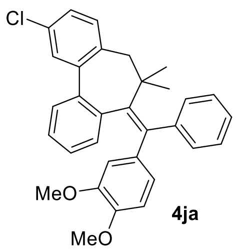

4ja

^{1}H-NMR (300 MHz, $CDCl_3$)

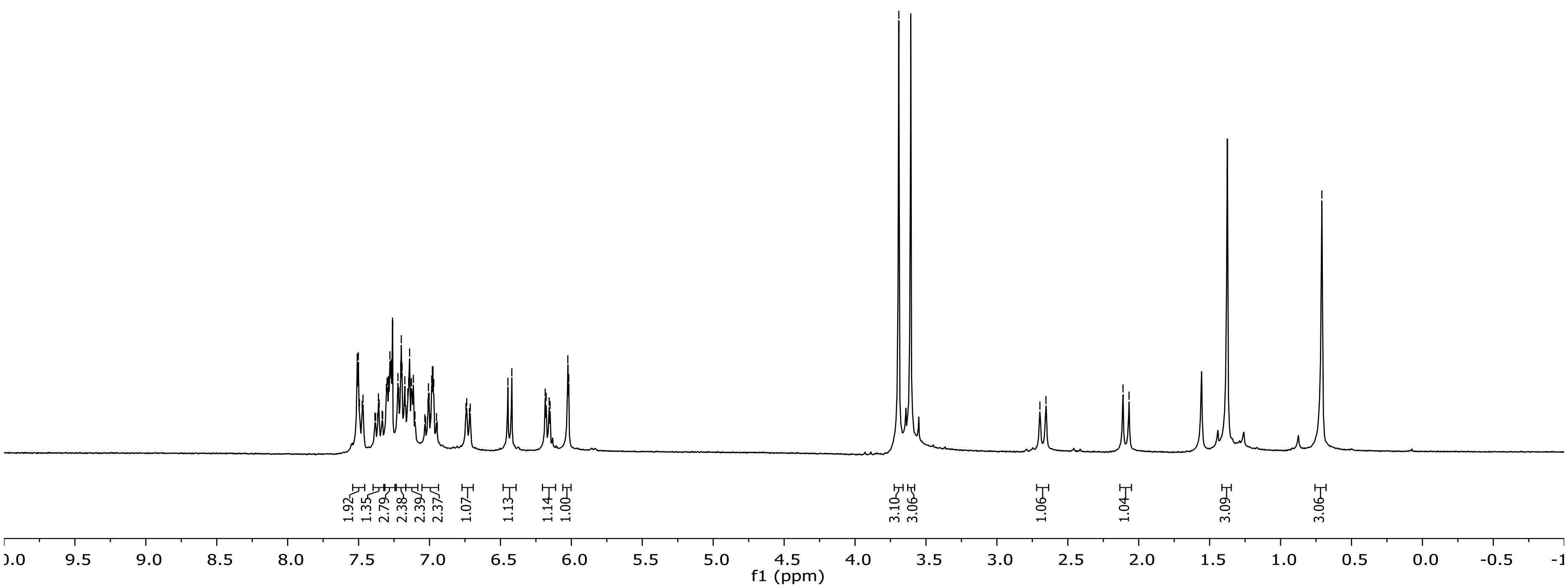

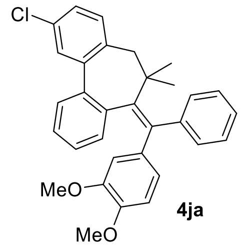

4ja

^{13}C-NMR (75 MHz, $CDCl_3$)

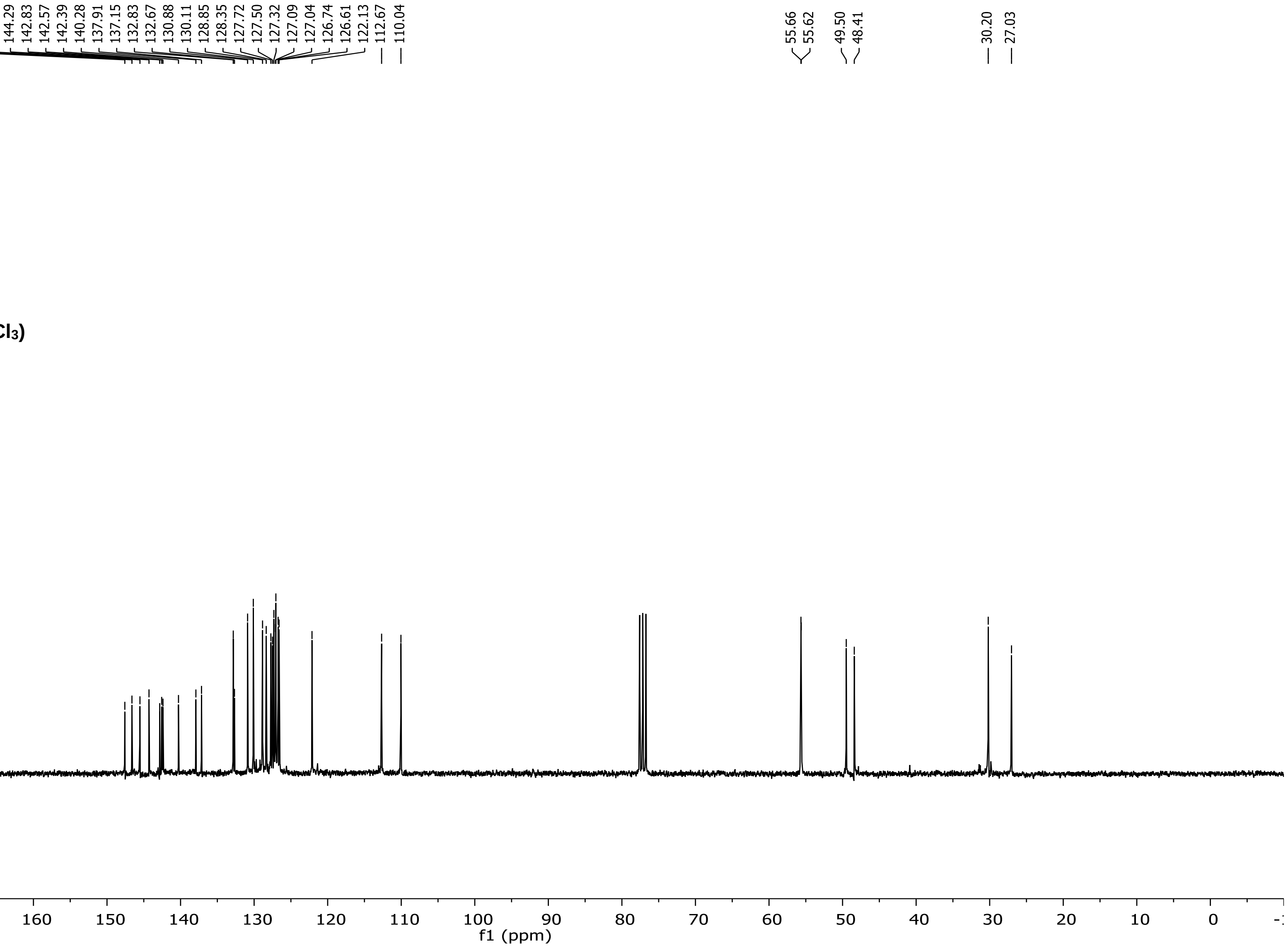

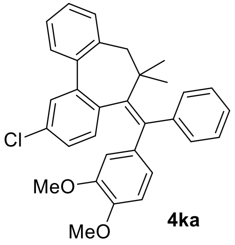

4ka

^{1}H-NMR (300 MHz, CDCl$_3$)

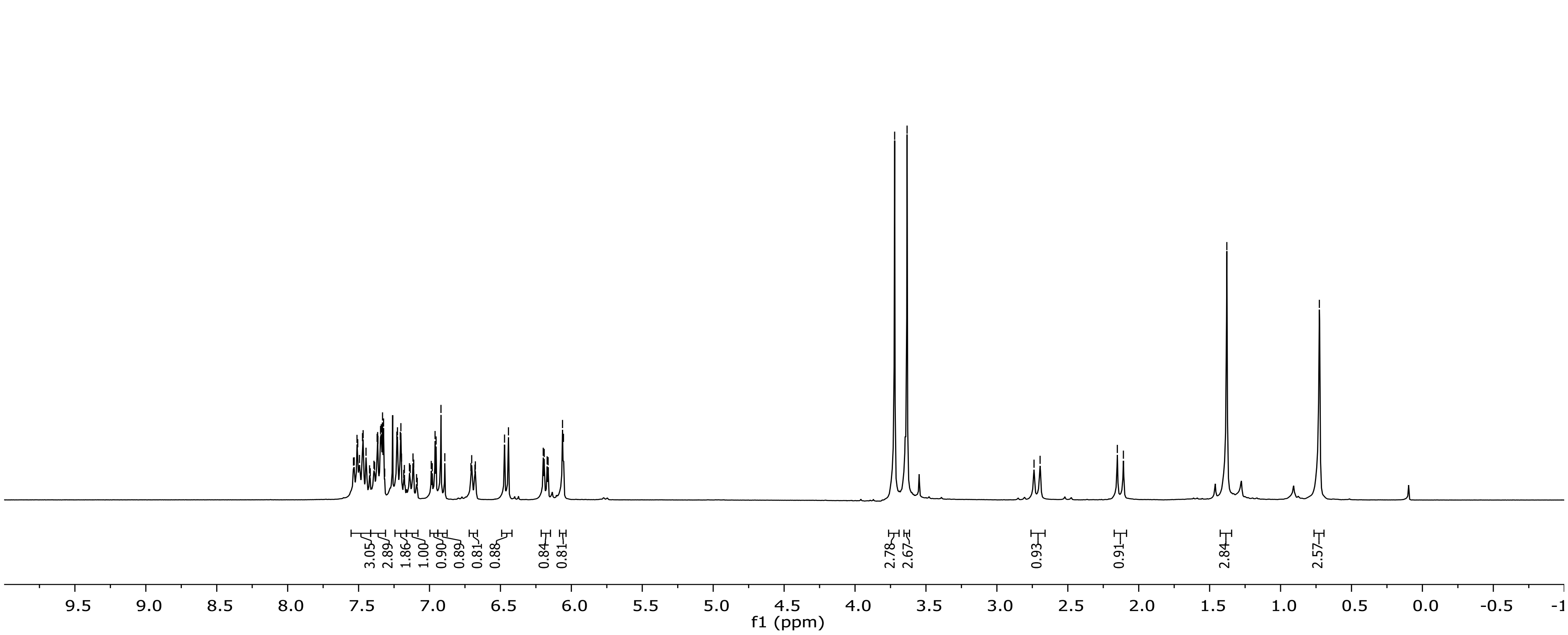

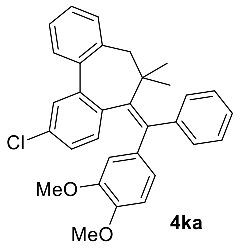

4ka

^{13}C-NMR (75 MHz, $CDCl_3$)

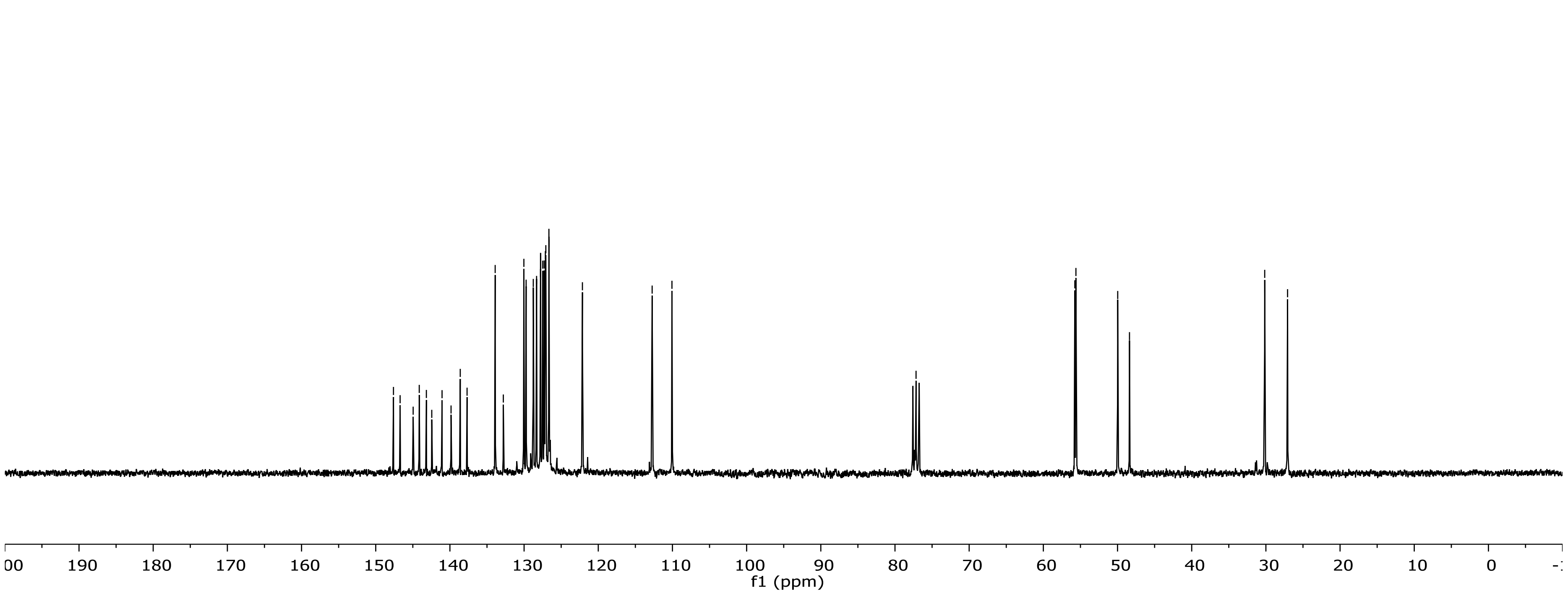

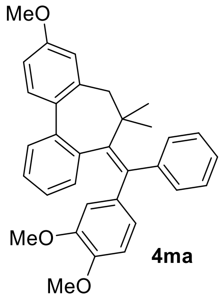

4ma

^{1}H-NMR (300 MHz, CDCl$_3$)

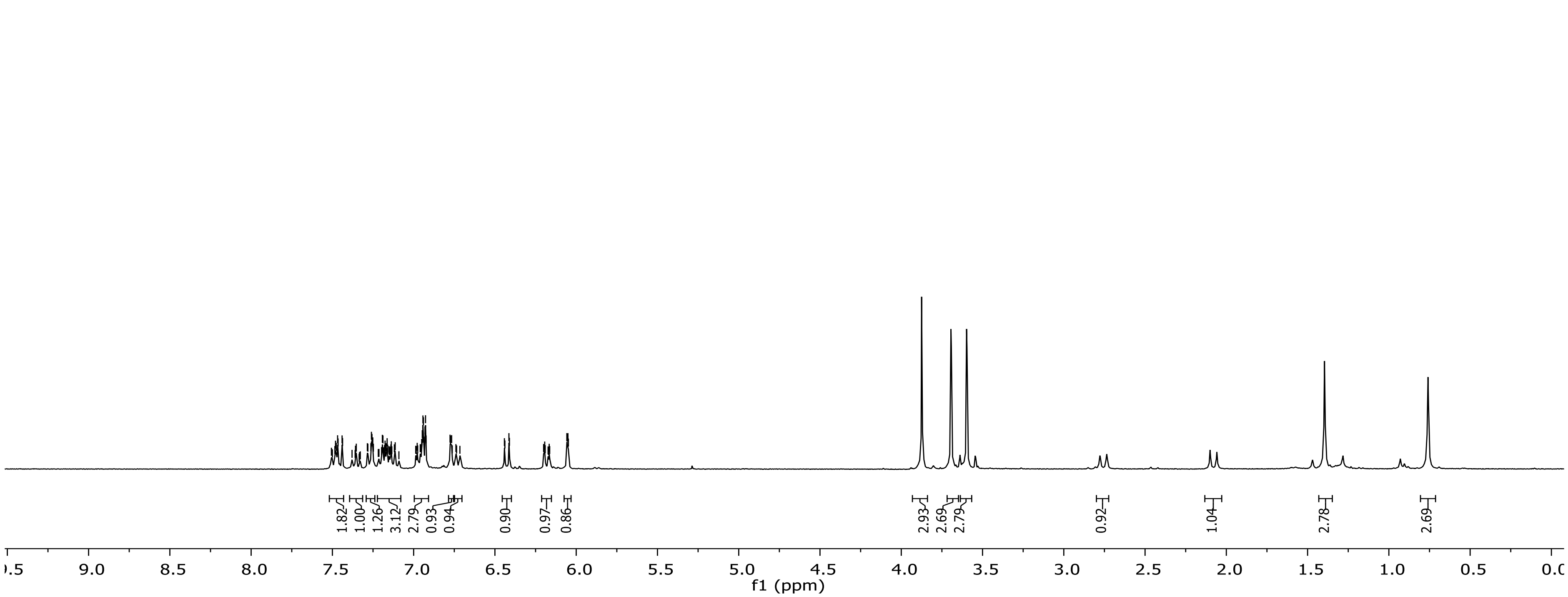

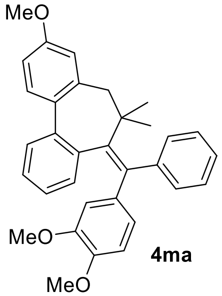

4ma

^{13}C-NMR (75 MHz, CDCl$_3$)

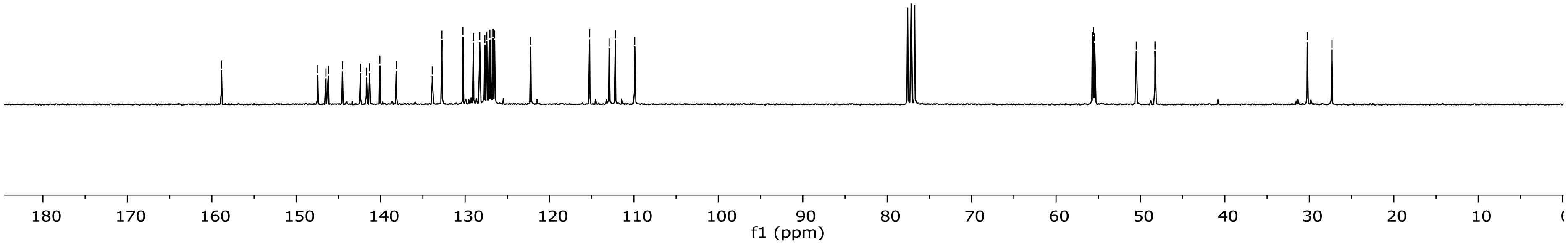

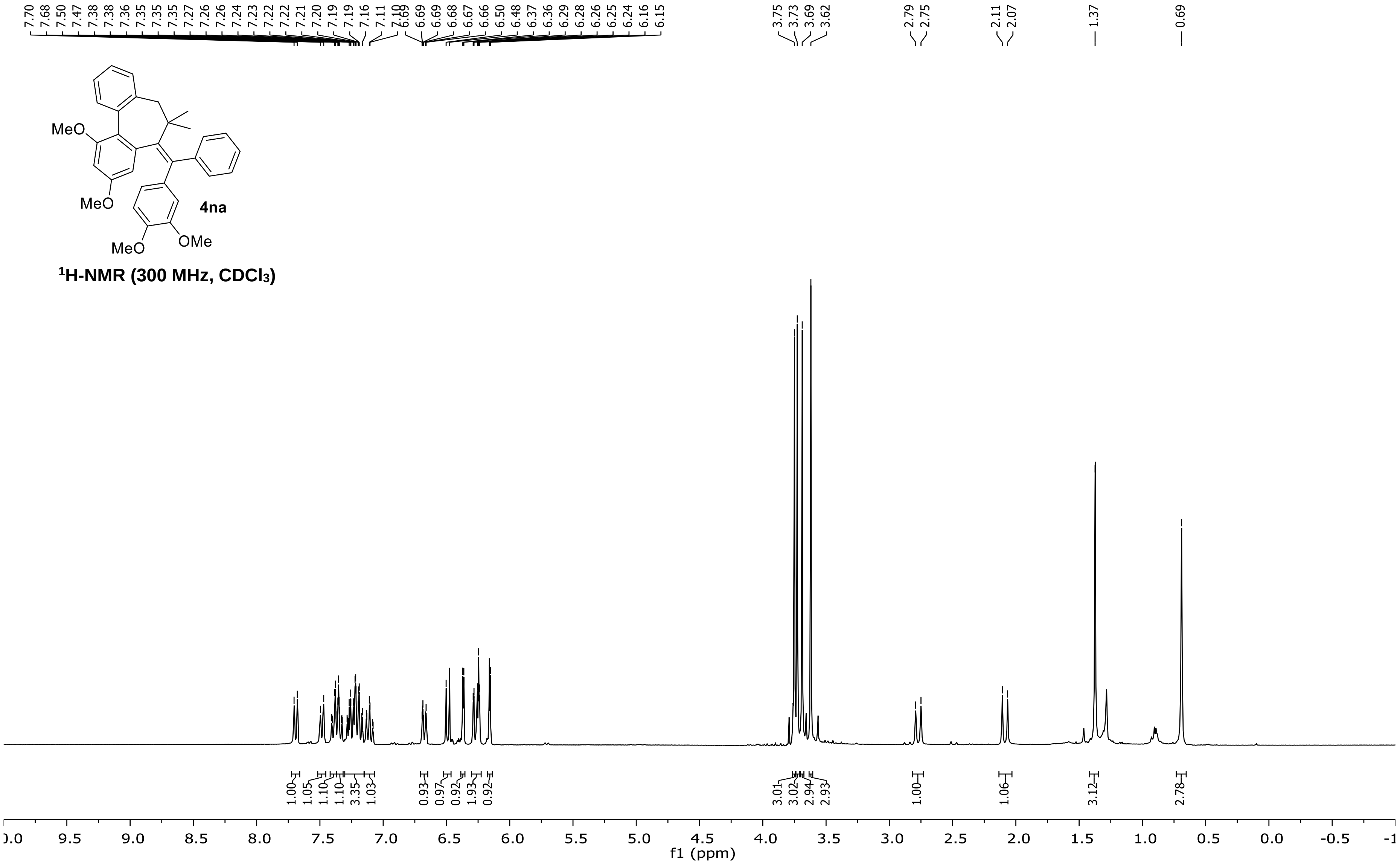

MeO
MeO
MeO
OMe
4na
^{1}H-NMR (300 MHz, CDCl₃)
f1 (ppm)

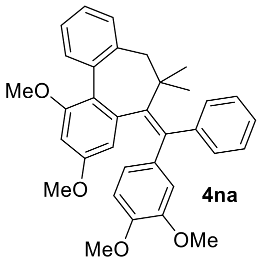

4na

^{13}C-NMR (75 MHz, $CDCl_3$)

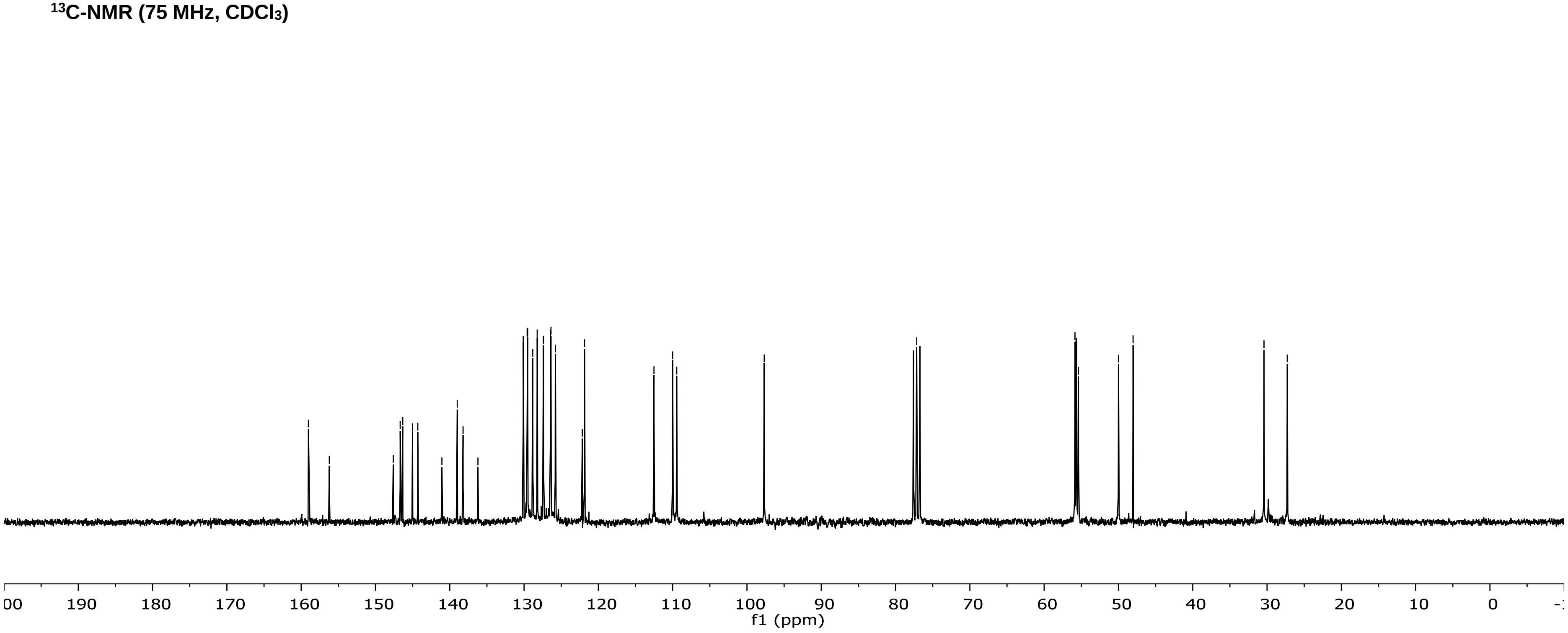

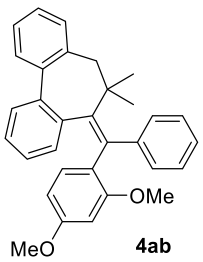

4ab

^{1}H-NMR (300 MHz, CDCl3)

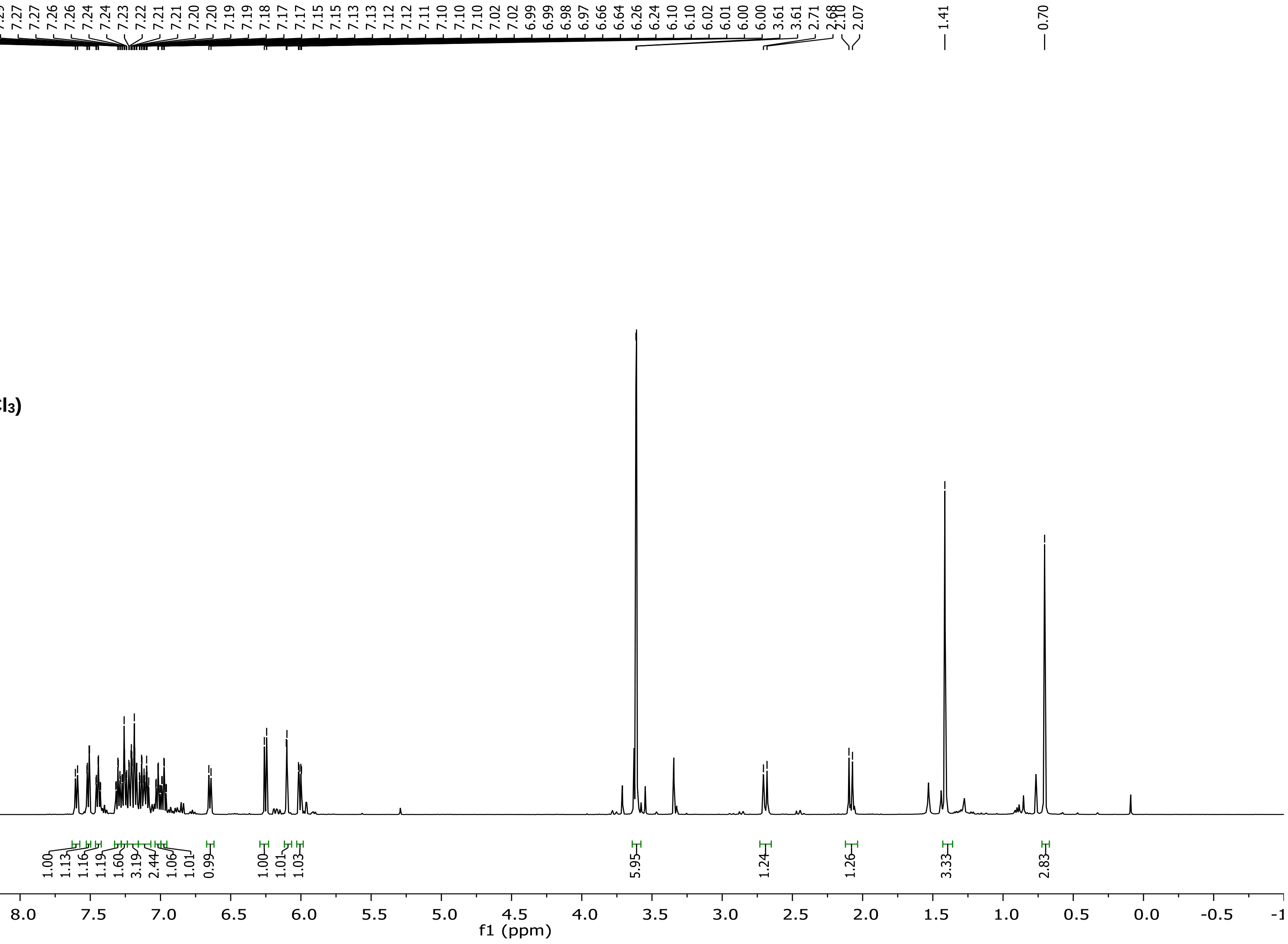

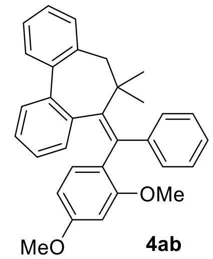

4ab

^{13}C-NMR (75 MHz, $CDCl_3$)

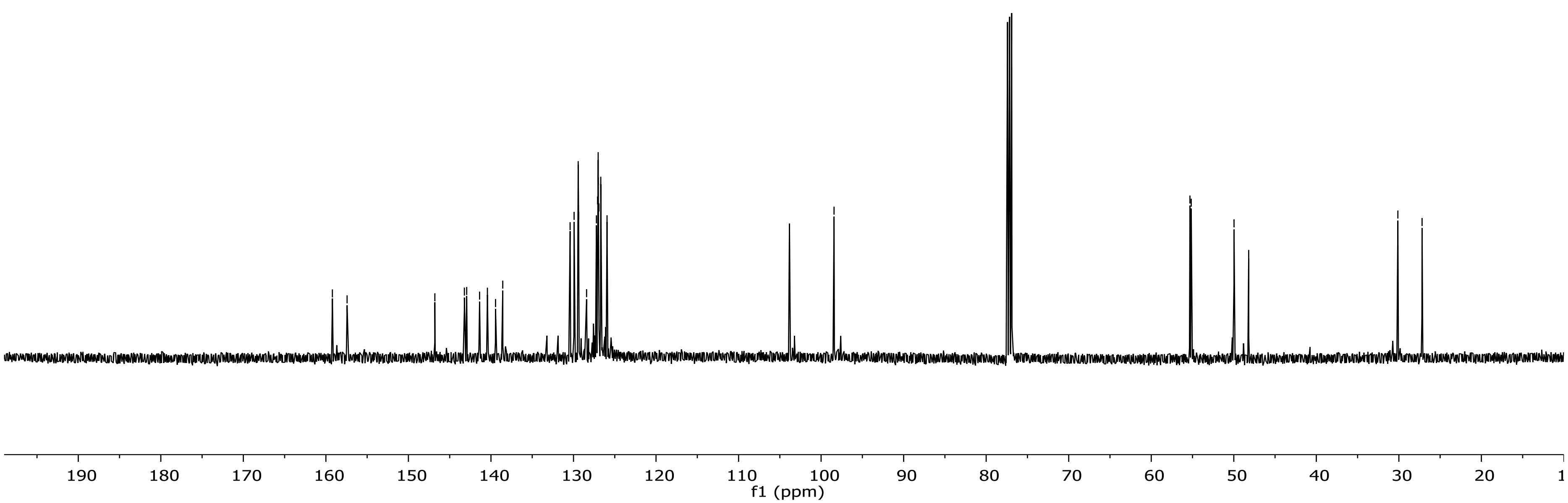

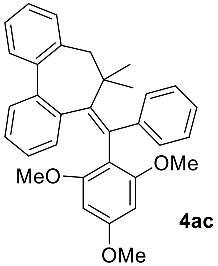

4ac

^{1}H-NMR (300 MHz, CDCl$_3$)

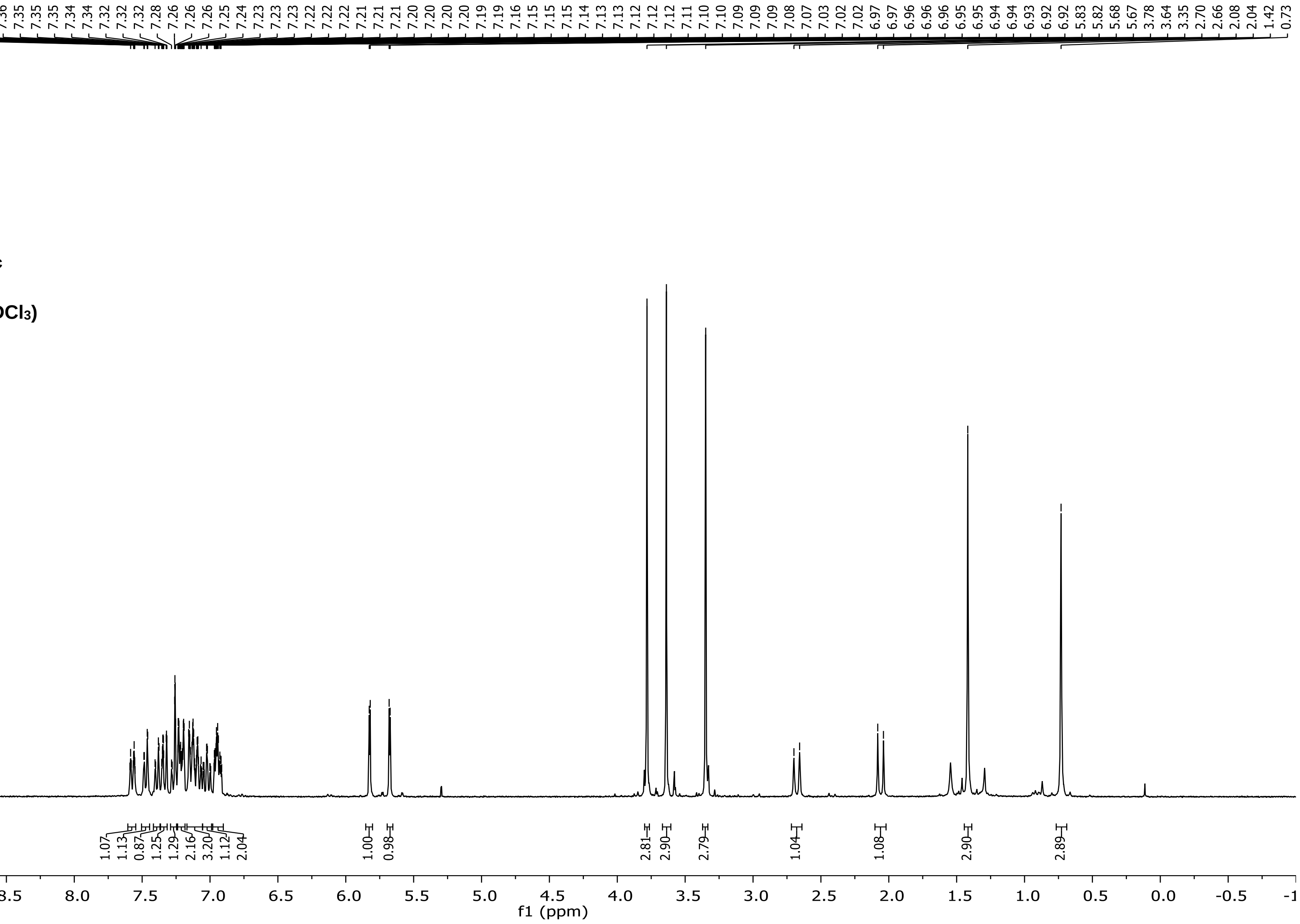

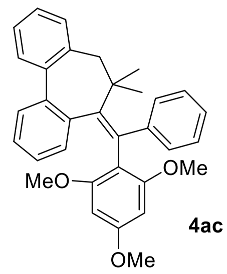

4ac

^{13}C-NMR (75 MHz, CDCl$_3$)

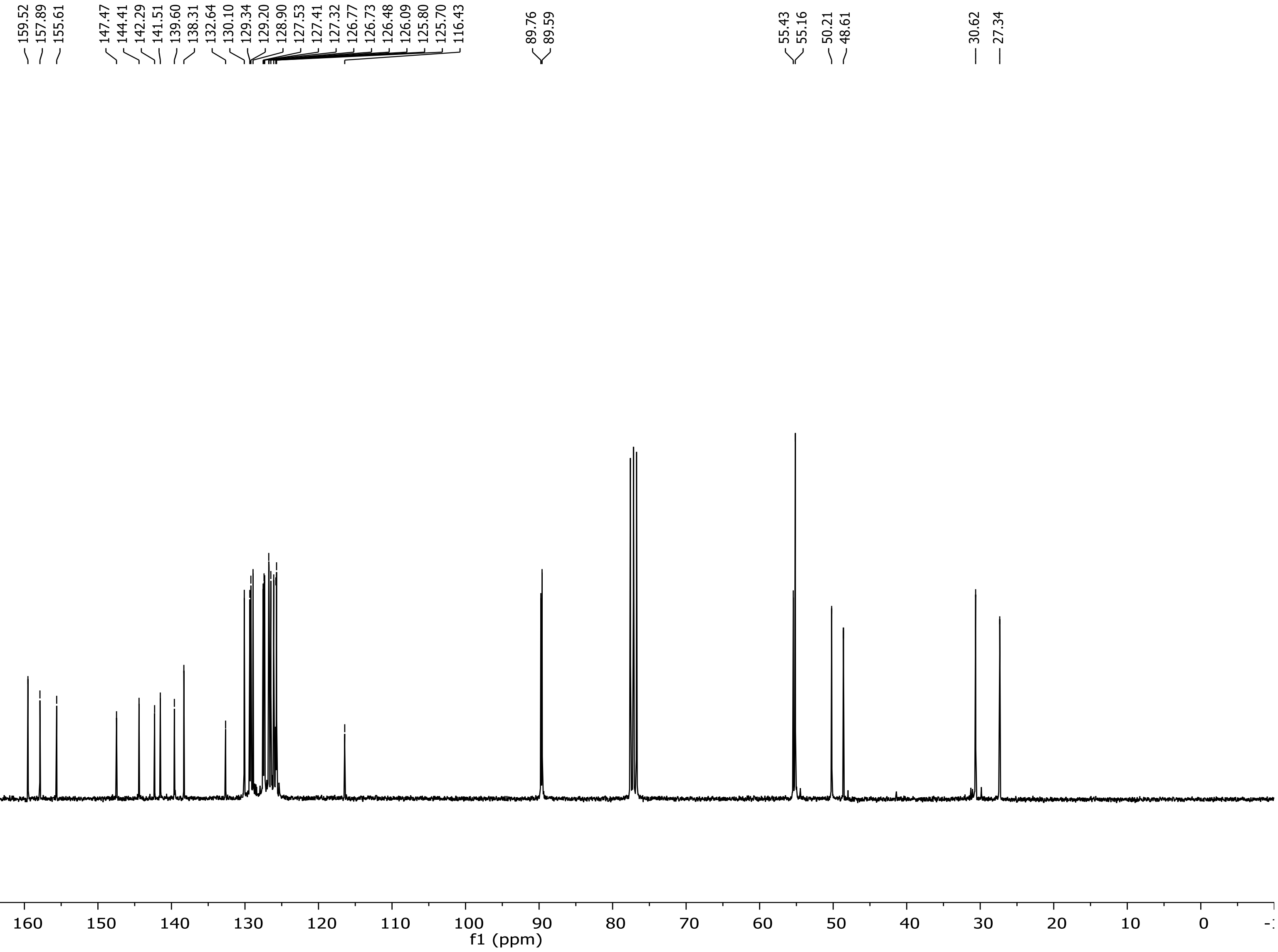

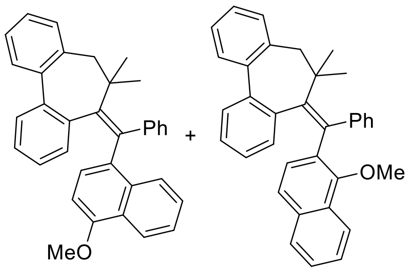

4ad **4ad'**

^{1}H-NMR (400 MHz, CDCl$_3$)

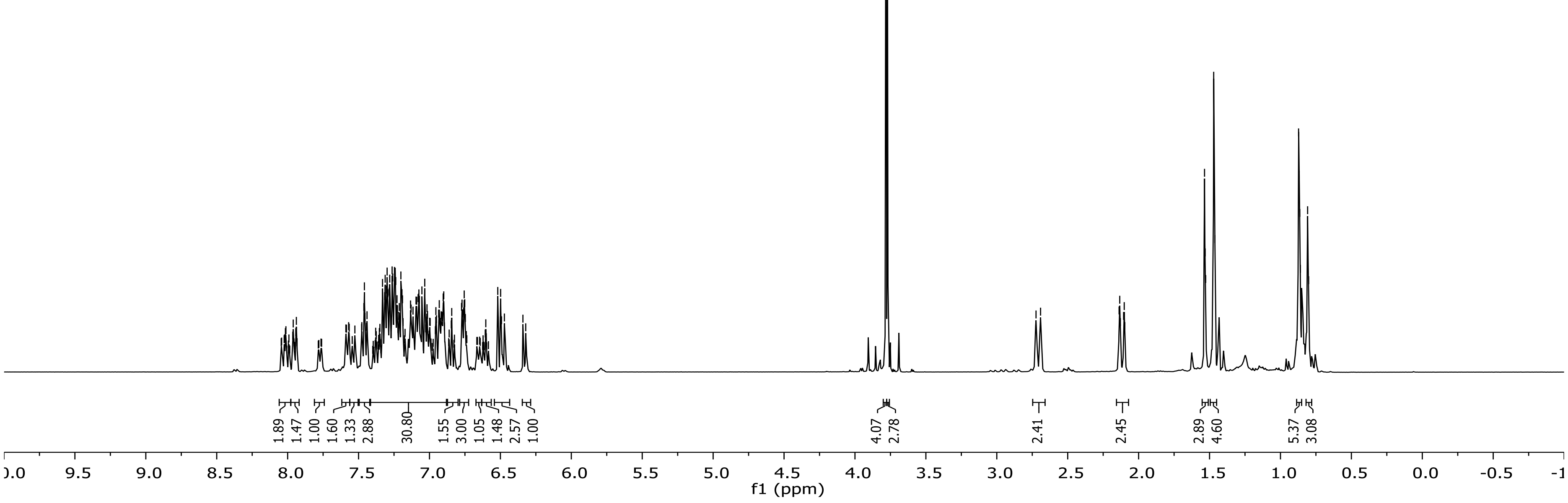

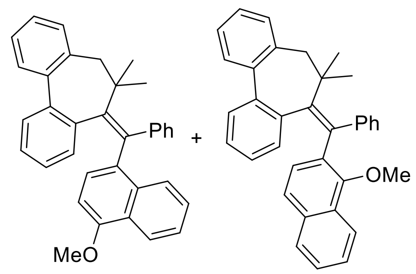

4ad **4ad'**

^{13}C-NMR (100 MHz, CDCl3)

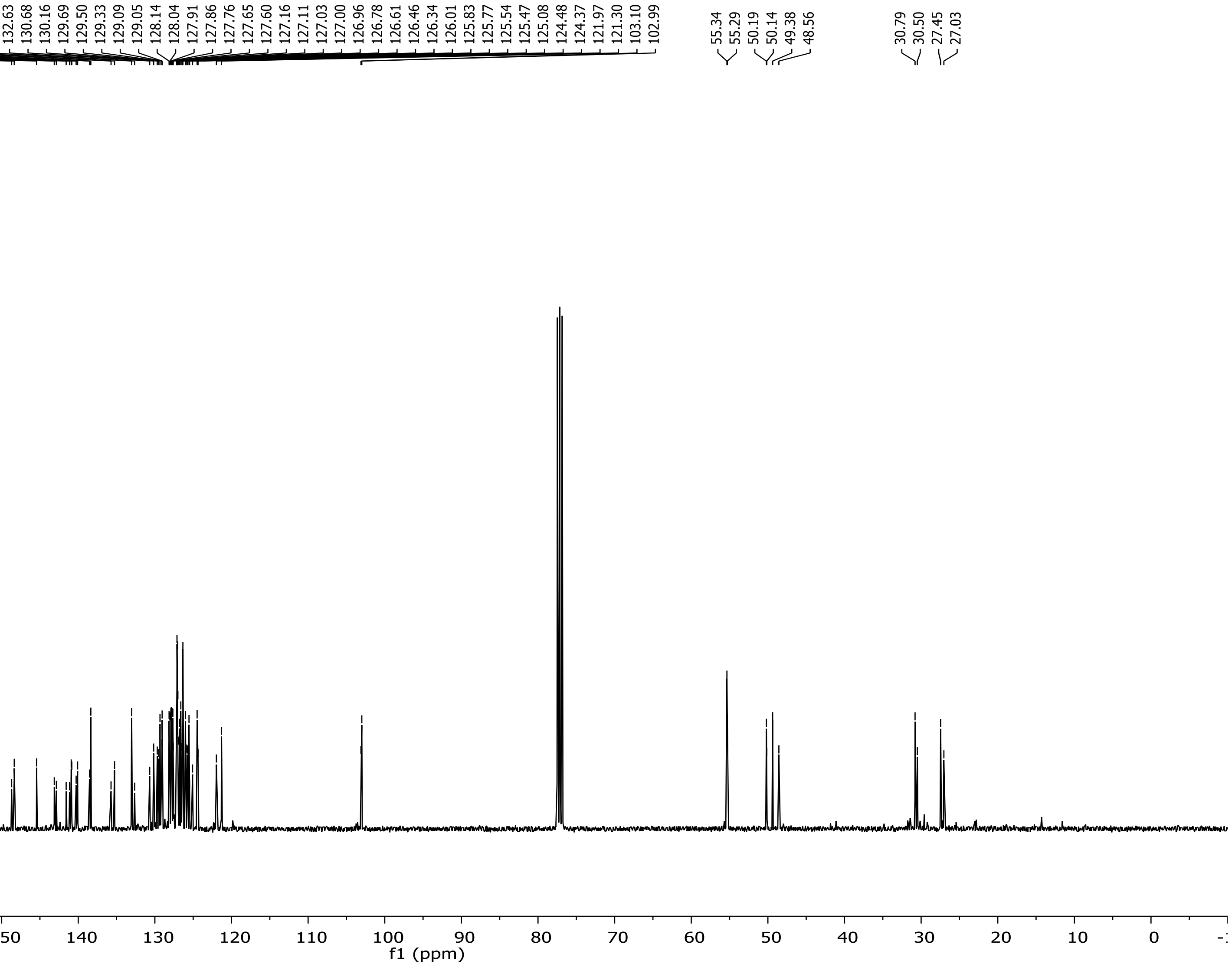

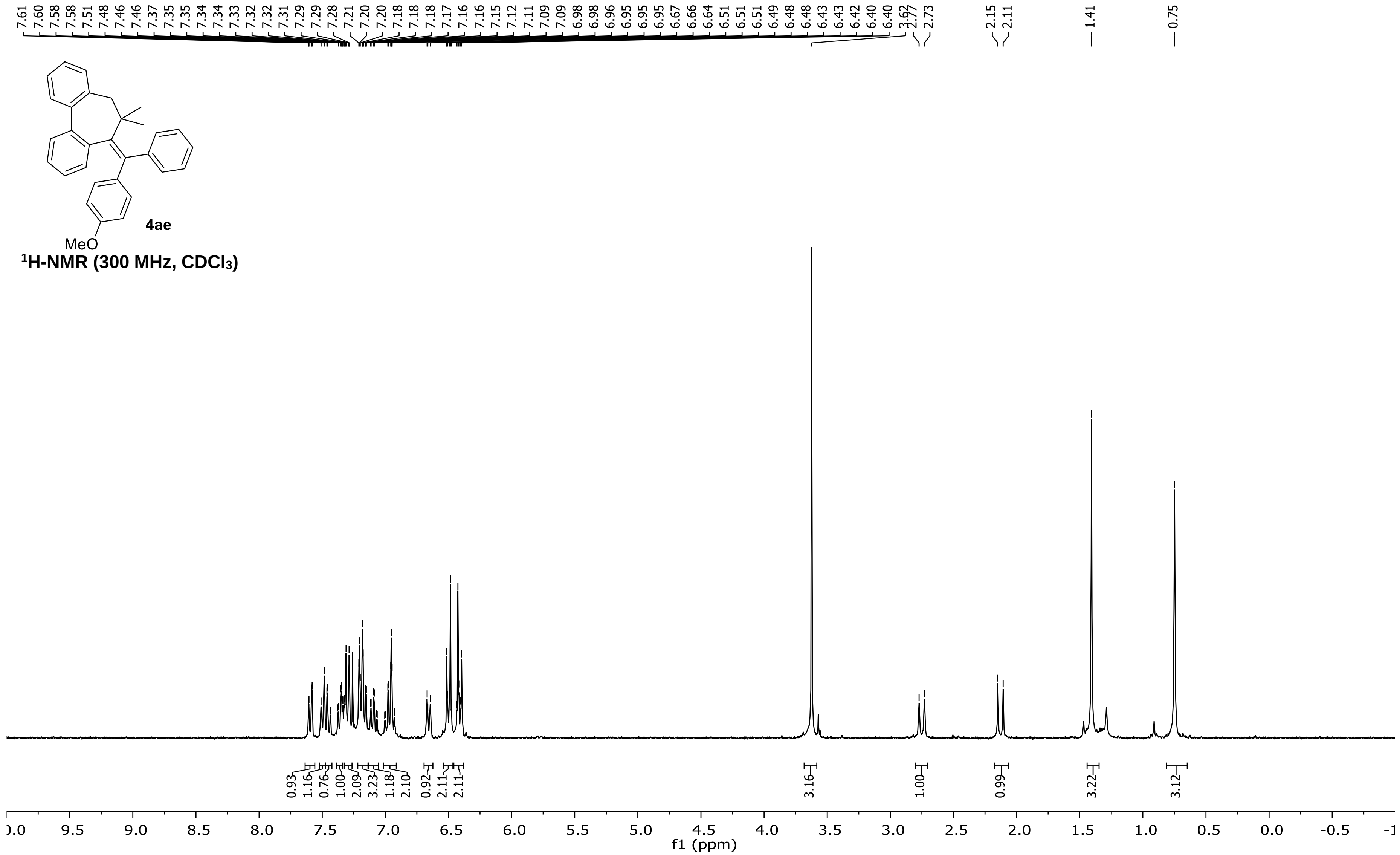
4ae
MeO
^{1}H-NMR (300 MHz, $CDCl_3$)
7.61 7.60 7.58 7.58 7.51 7.48 7.46 7.46 7.37 7.35 7.35 7.34 7.34 7.33 7.32 7.32 7.31 7.29 7.29 7.28 7.21 7.20 7.20 7.18 7.18 7.18 7.17 7.16 7.16 7.15 7.12 7.11 7.09 7.09 6.98 6.98 6.96 6.95 6.95 6.95 6.67 6.66 6.64 6.51 6.51 6.51 6.49 6.48 6.48 6.43 6.43 6.42 6.40 6.40 3.62 2.77 2.73
2.15 2.11
1.41
0.75
0.93 1.16 0.76 1.00 2.09 3.23 1.18 2.10 0.92 2.11 2.11
3.16
1.00
0.99
3.22
3.12
f1 (ppm)

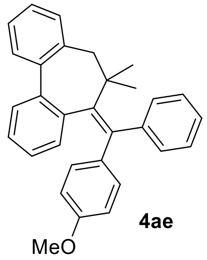

4ae

^{13}C-NMR (75 MHz, CDCl$_3$)

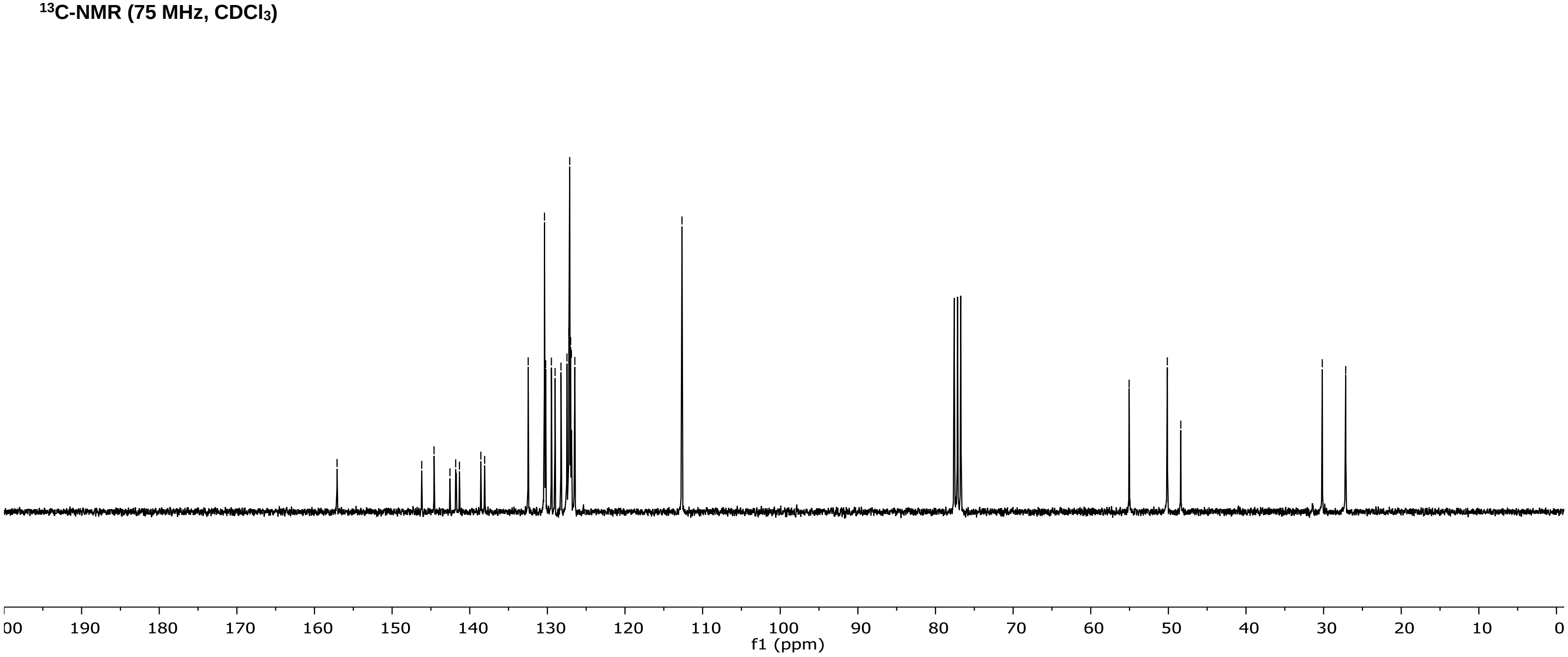

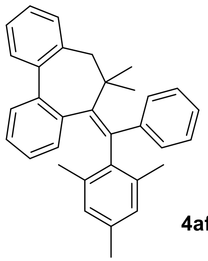

4af

^{1}H-NMR (300 MHz, $CDCl_3$)

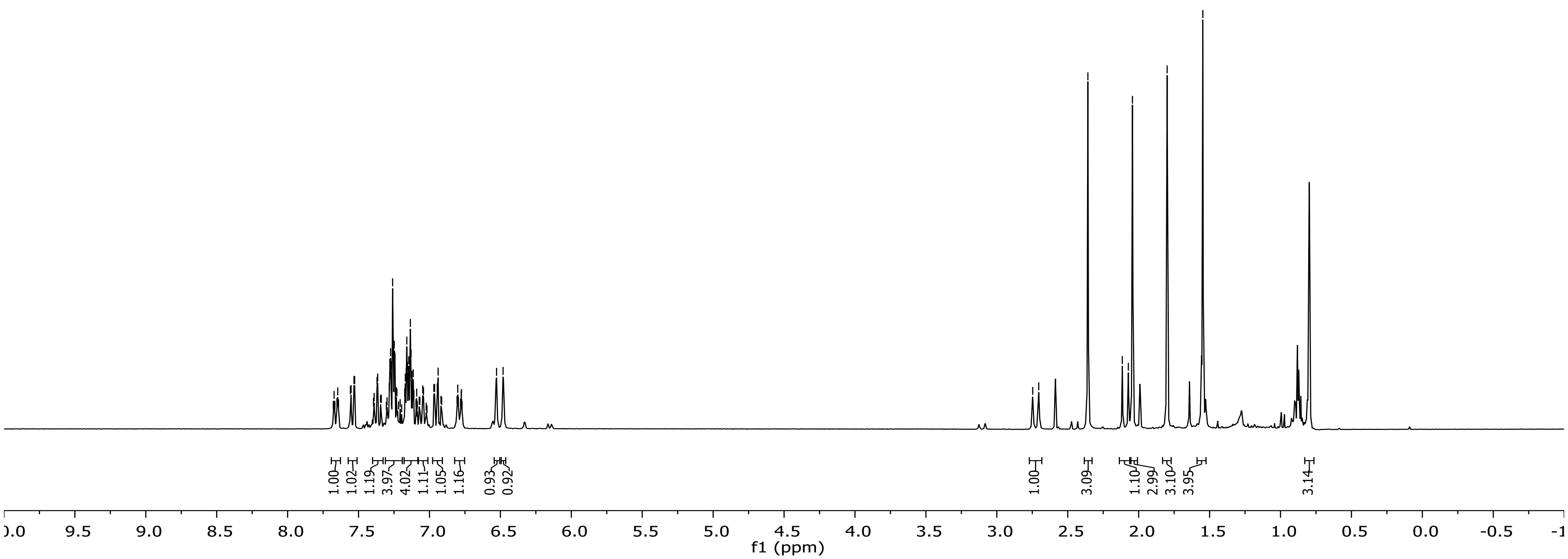

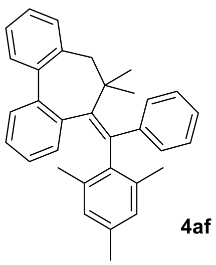

4af

^{13}C-NMR (75 MHz, $CDCl_3$)

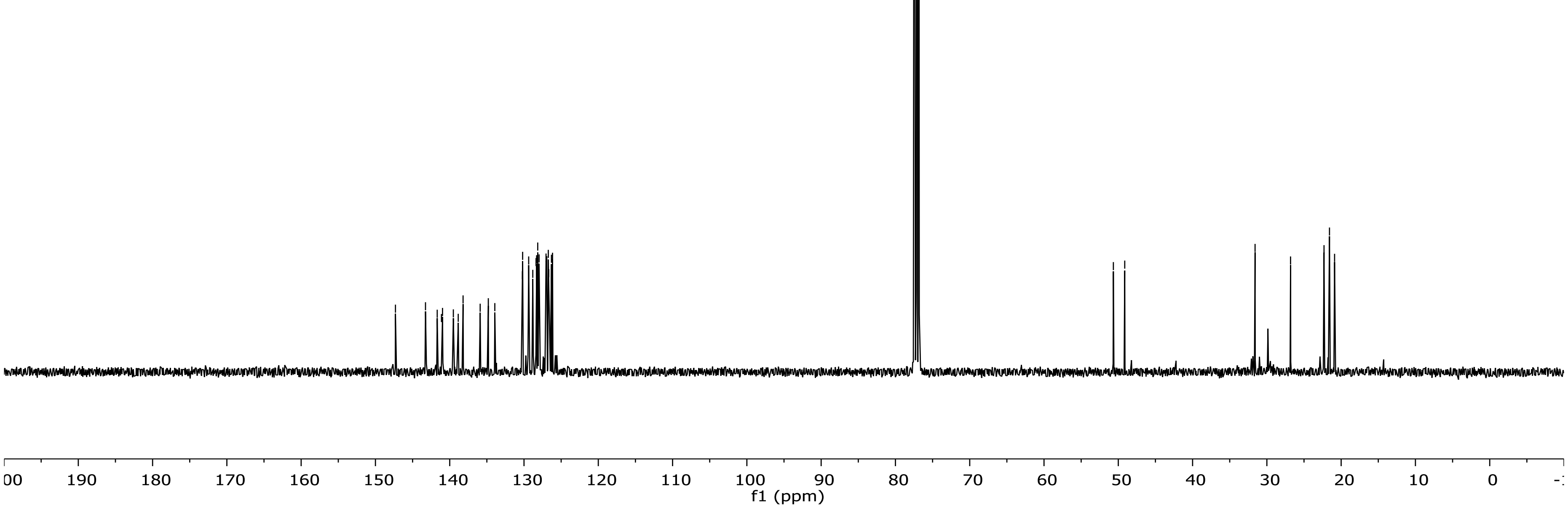

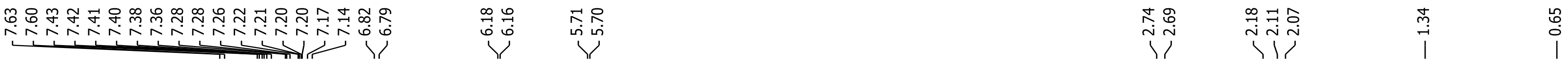

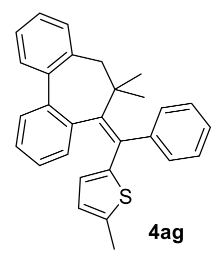

4ag

^{1}H-NMR (300 MHz, CDCl₃)

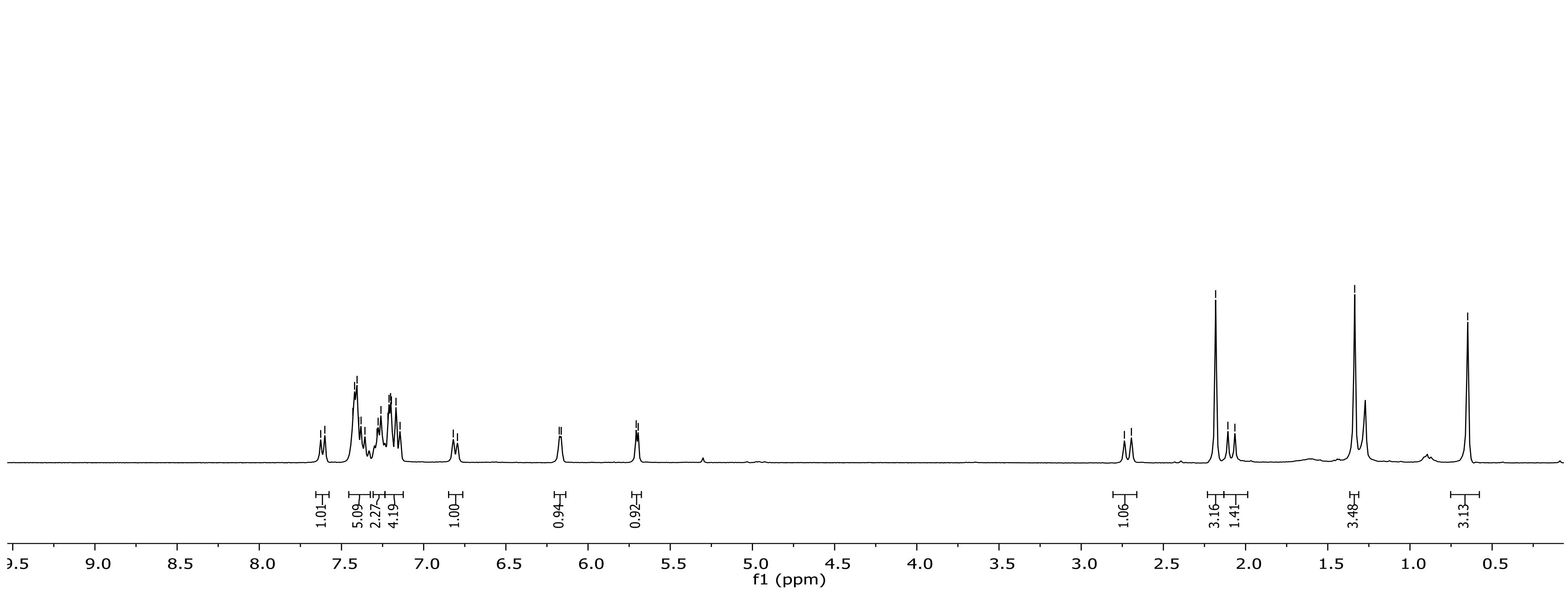

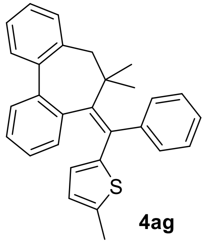

4ag

^{13}C-NMR (75 MHz, $CDCl_3$)

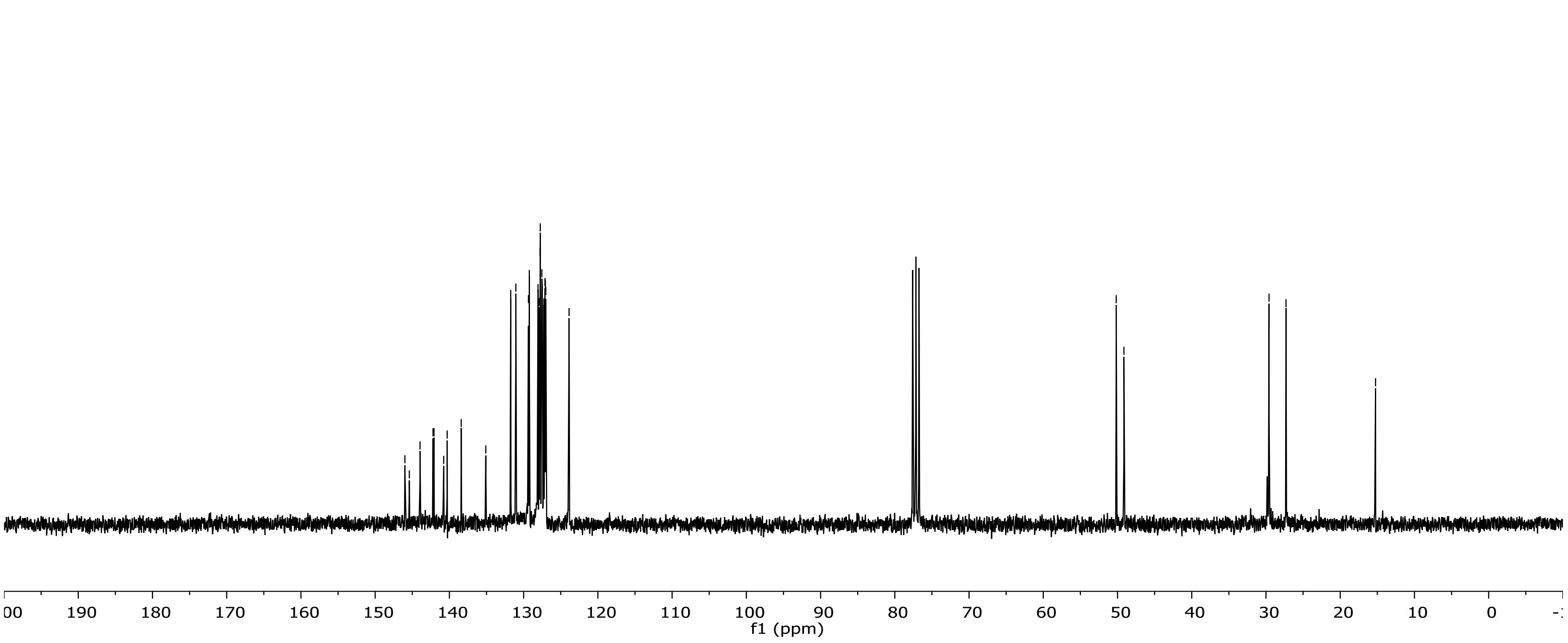

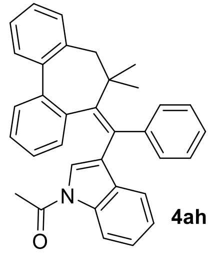

4ah

^{1}H-NMR (300 MHz, CDCl$_3$)

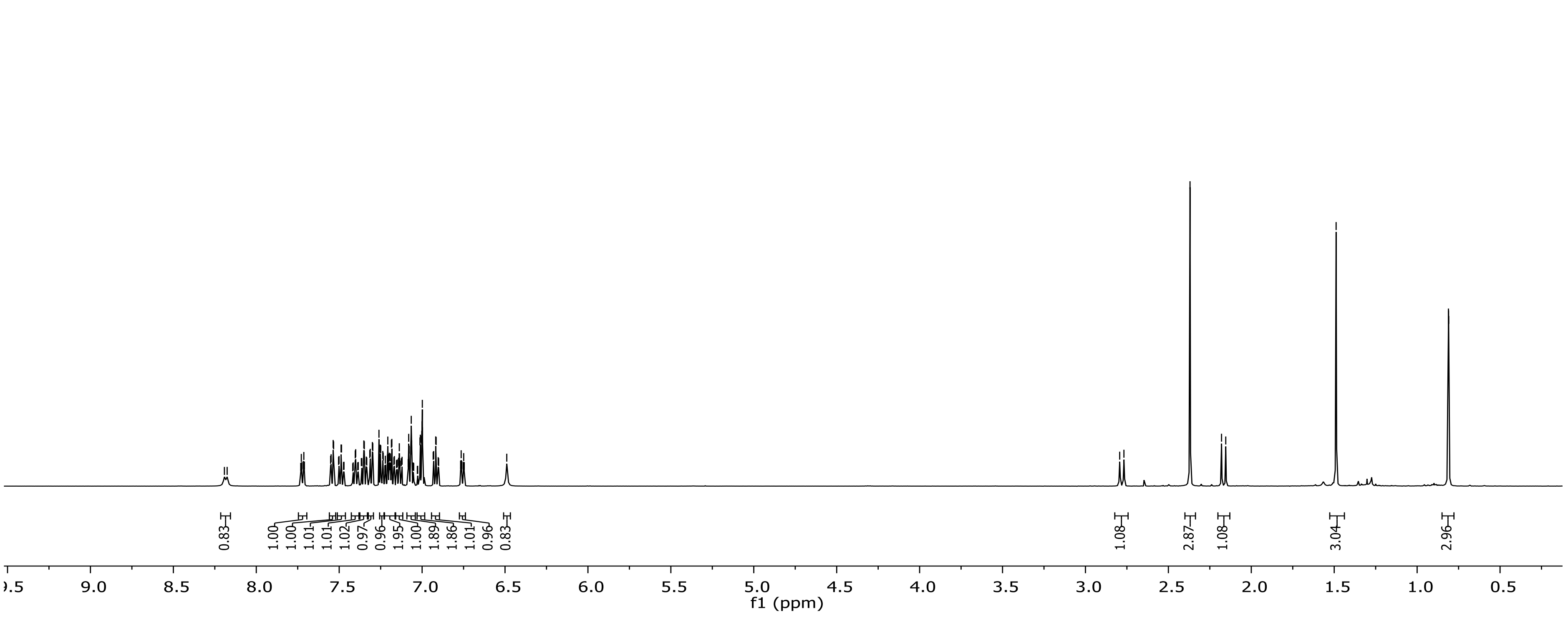

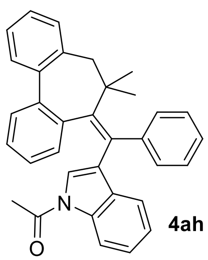

4ah

^{13}C-NMR (75 MHz, $CDCl_3$)

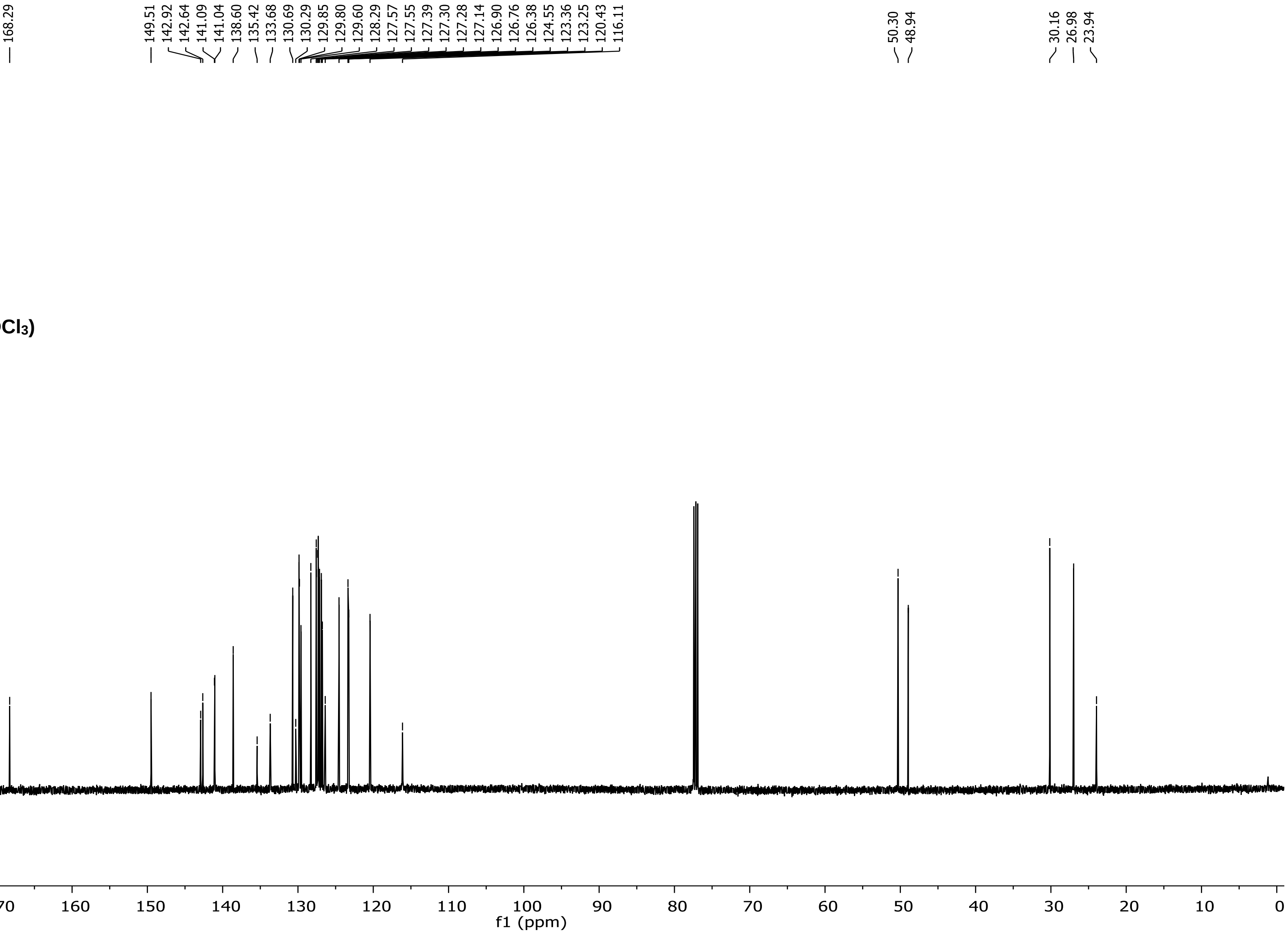

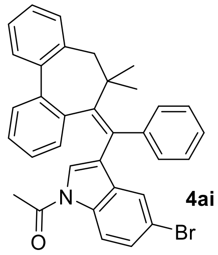

4ai

^{1}H-NMR (300 MHz, $CDCl_3$)

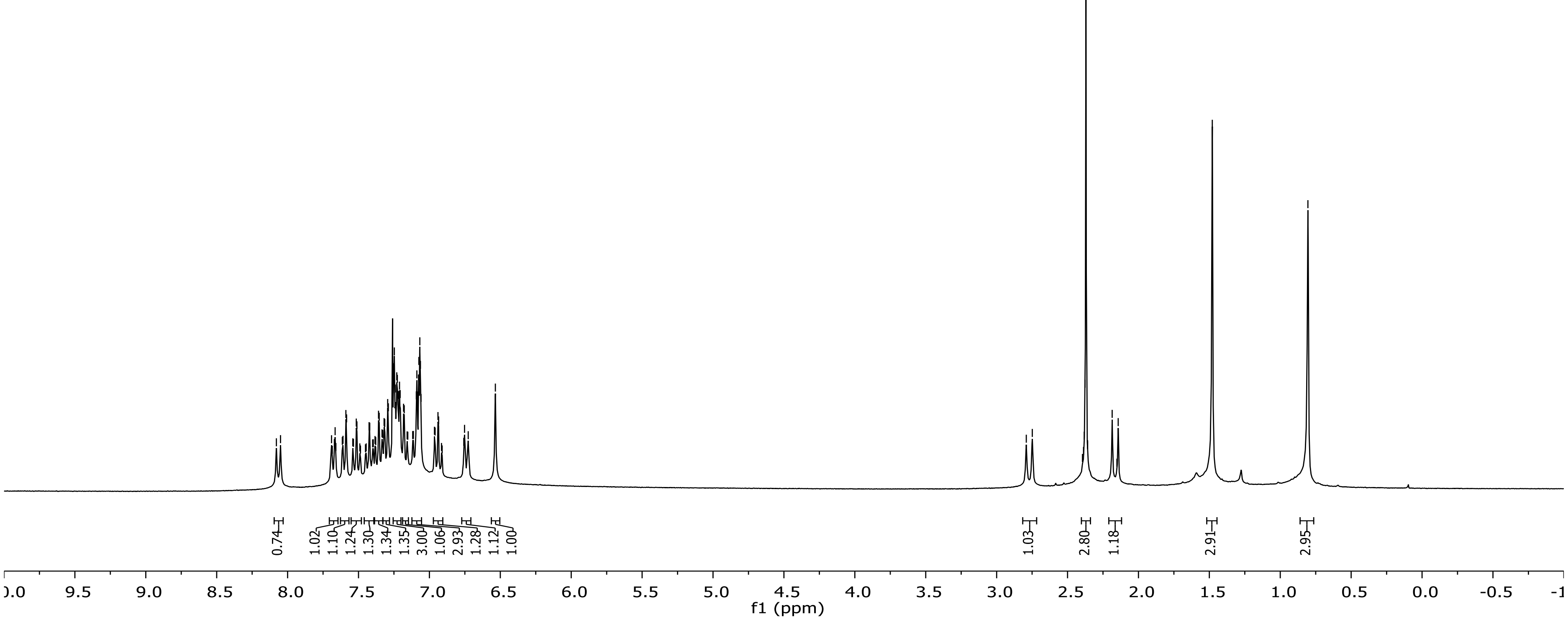

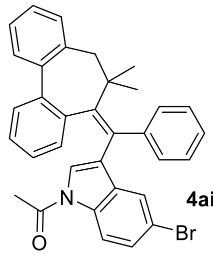

4ai

^{13}C-NMR (75 MHz, CDCl$_3$)

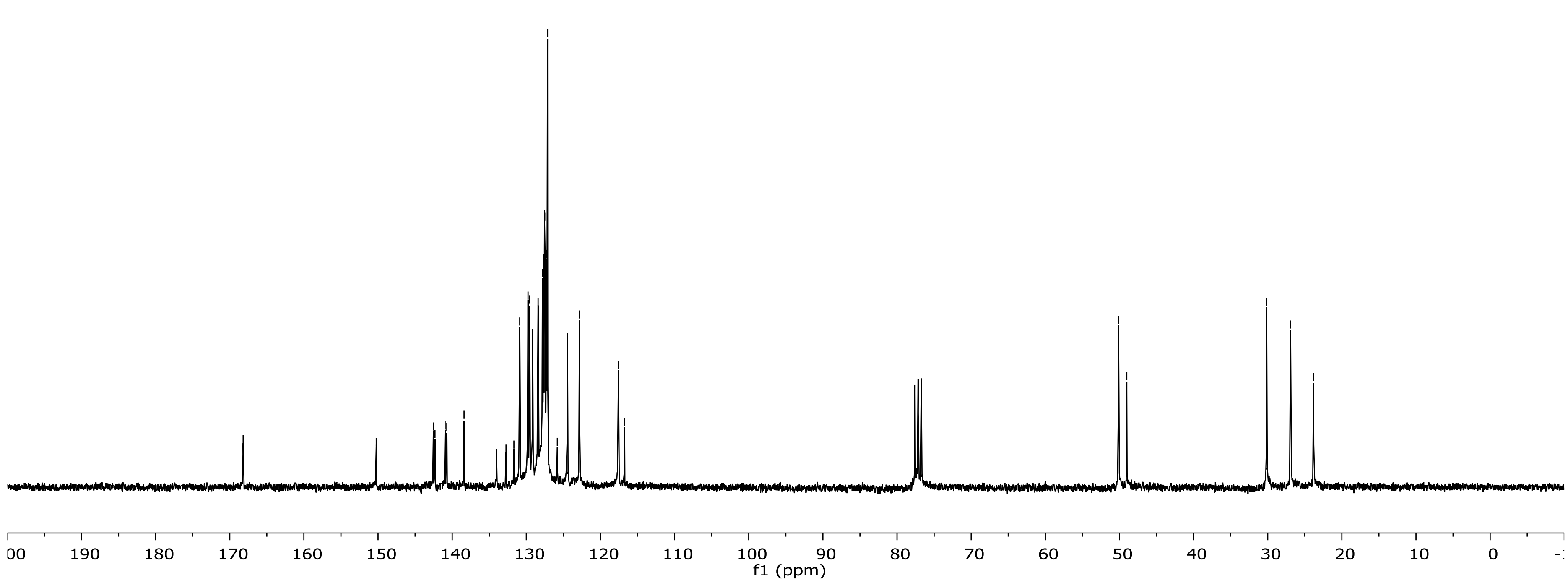

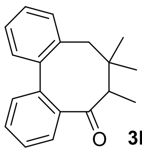

3b

^{1}H-NMR (500 MHz, $CDCl_3$)

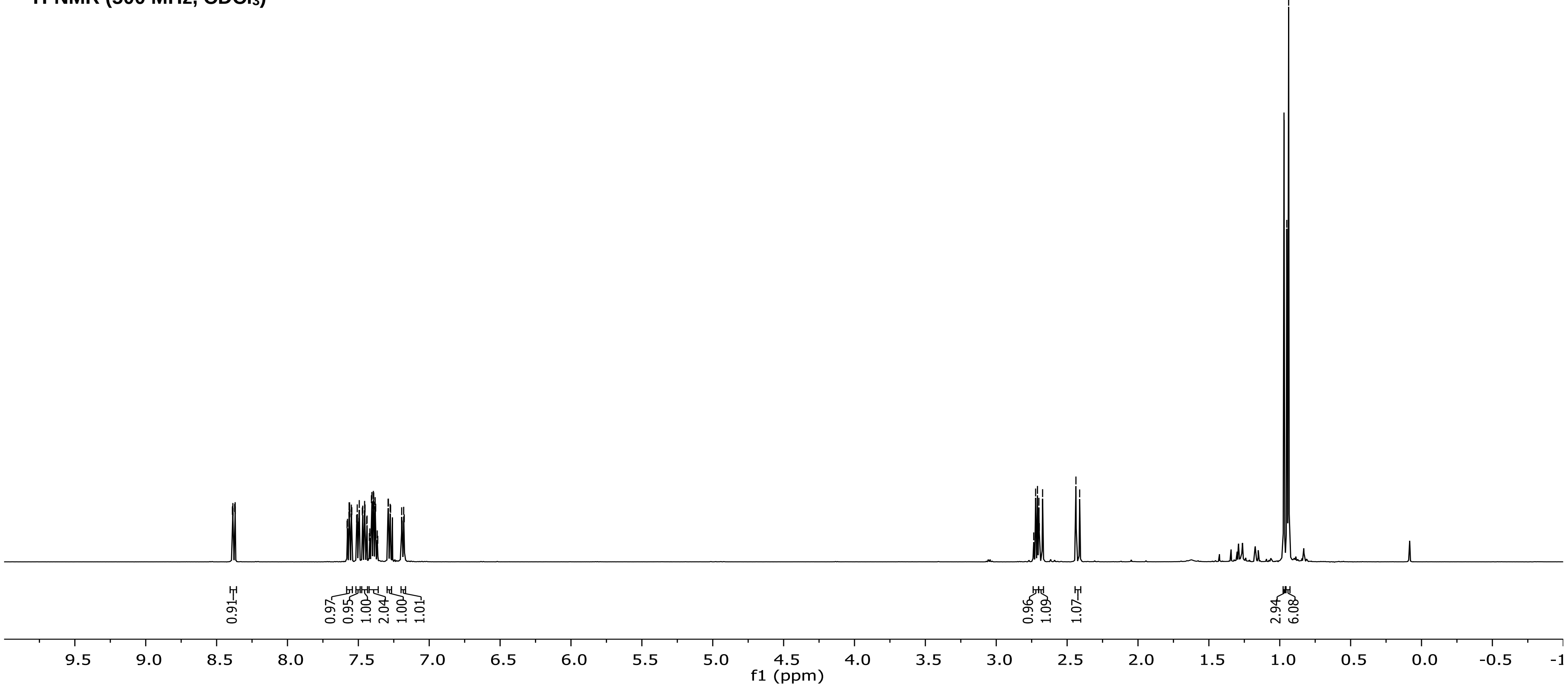

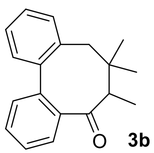

3b

^{13}C-NMR (125 MHz, $CDCl_3$)

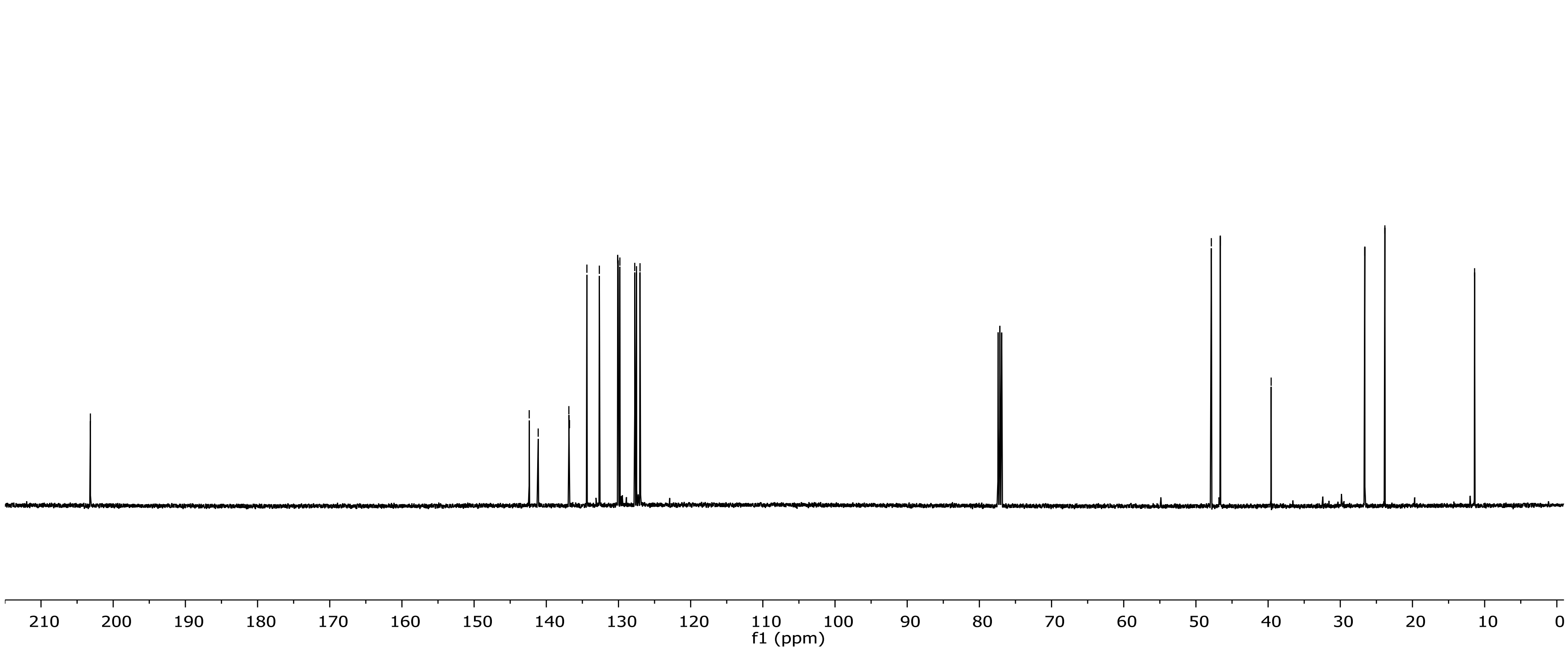

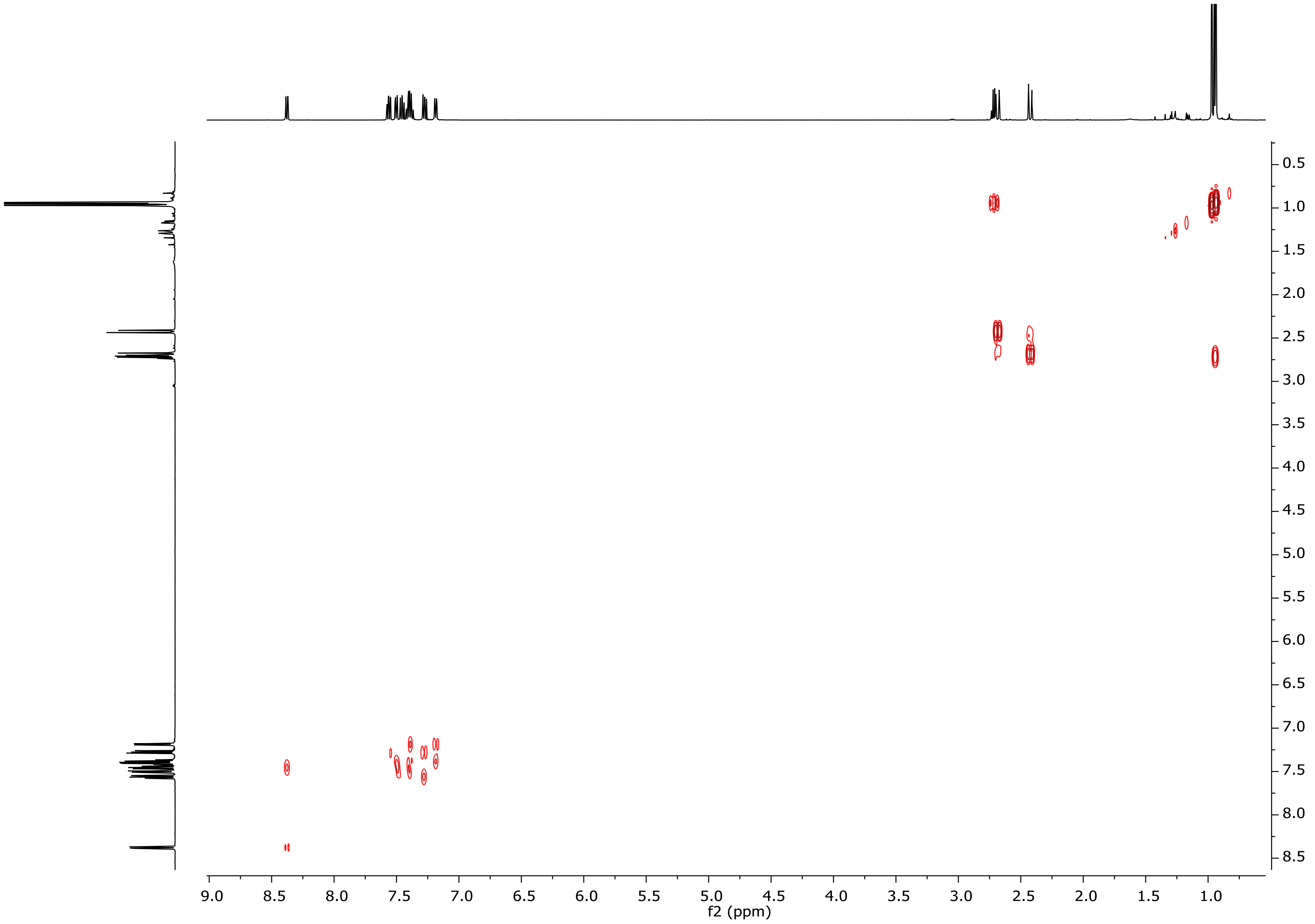

0.5
1.0
1.5
2.0
2.5
3.0
3.5
4.0
4.5
5.0
5.5
6.0
6.5
7.0
7.5
8.0
8.5
f1 (ppm)
9.0
8.5
8.0
7.5
7.0
6.5
6.0
5.5
5.0
4.5
4.0
3.5
3.0
2.5
2.0
1.5
1.0
f2 (ppm)

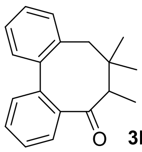

O

3b

gCOSY-NMR

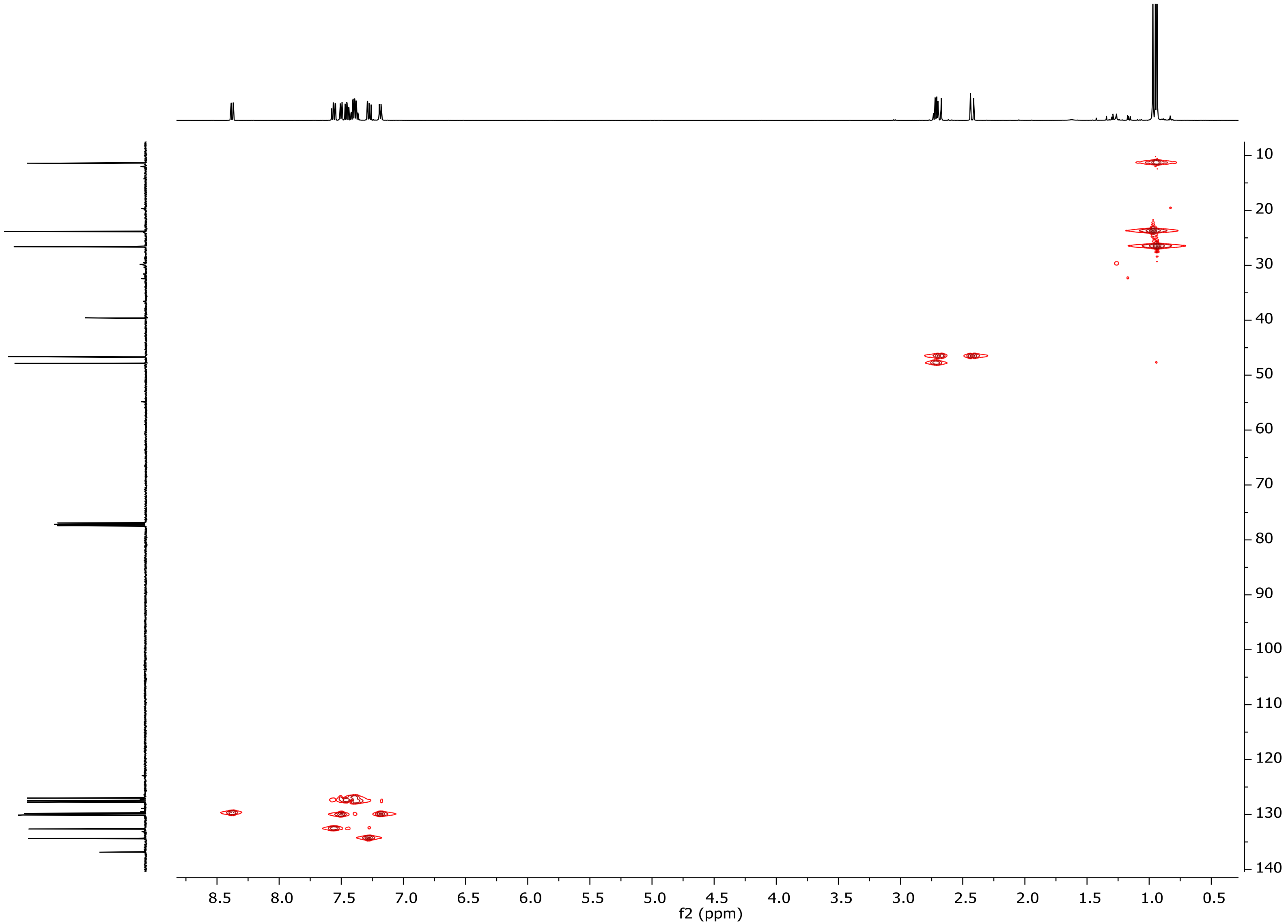
8.5
8.0
7.5
7.0
6.5
6.0
5.5
5.0
4.5
4.0
3.5
3.0
2.5
2.0
1.5
1.0
0.5
f2 (ppm)
10
20
30
40
50
60
70
80
90
100
110
120
130
140
f1 (ppm)

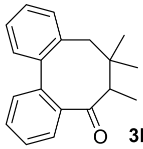
O

3b

gHSQC-NMR

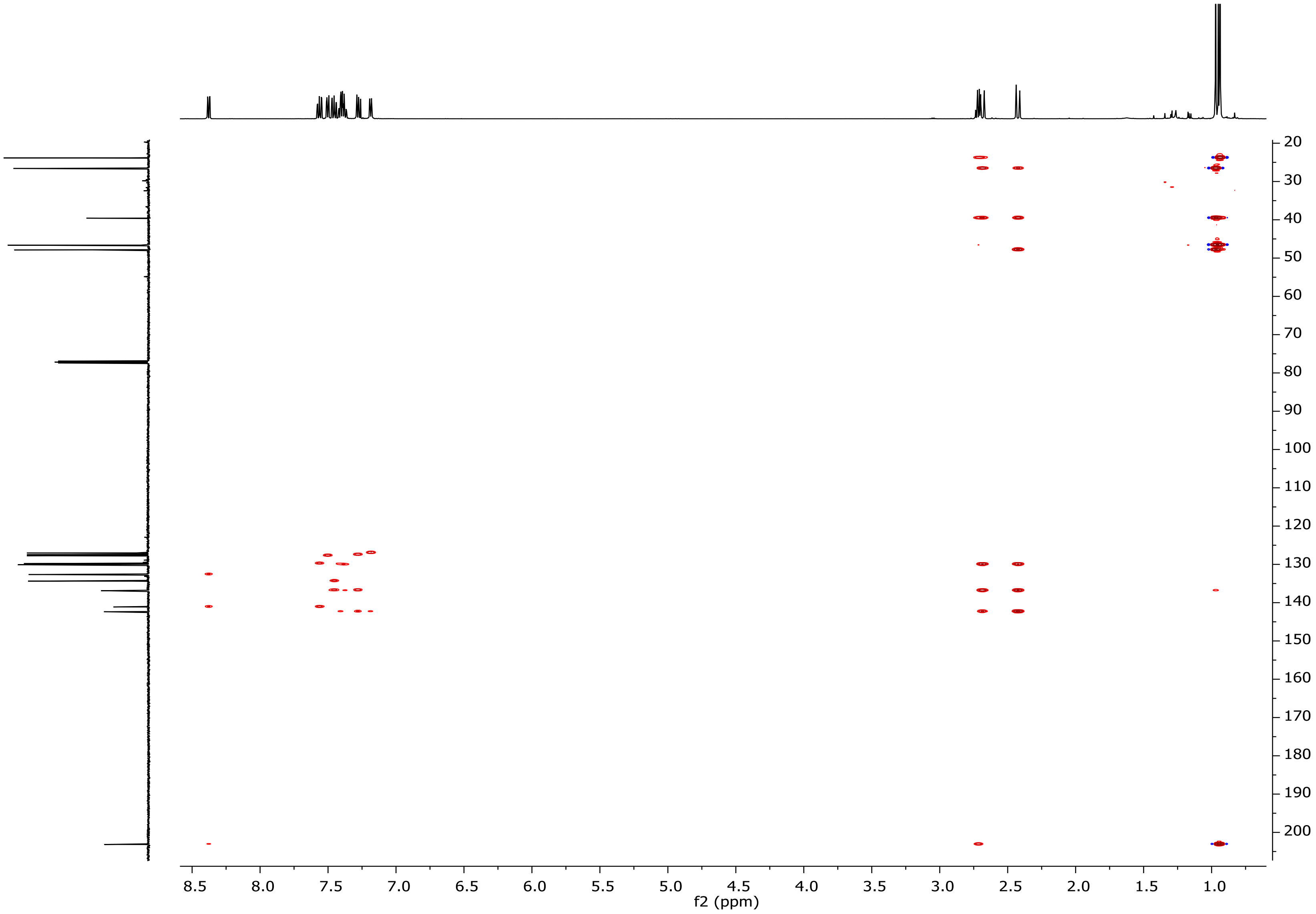

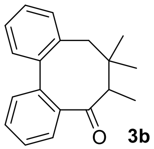

3b

gHMBC-NMR

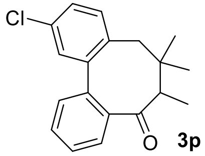

3p

^{1}H-NMR (300 MHz, $CDCl_3$)

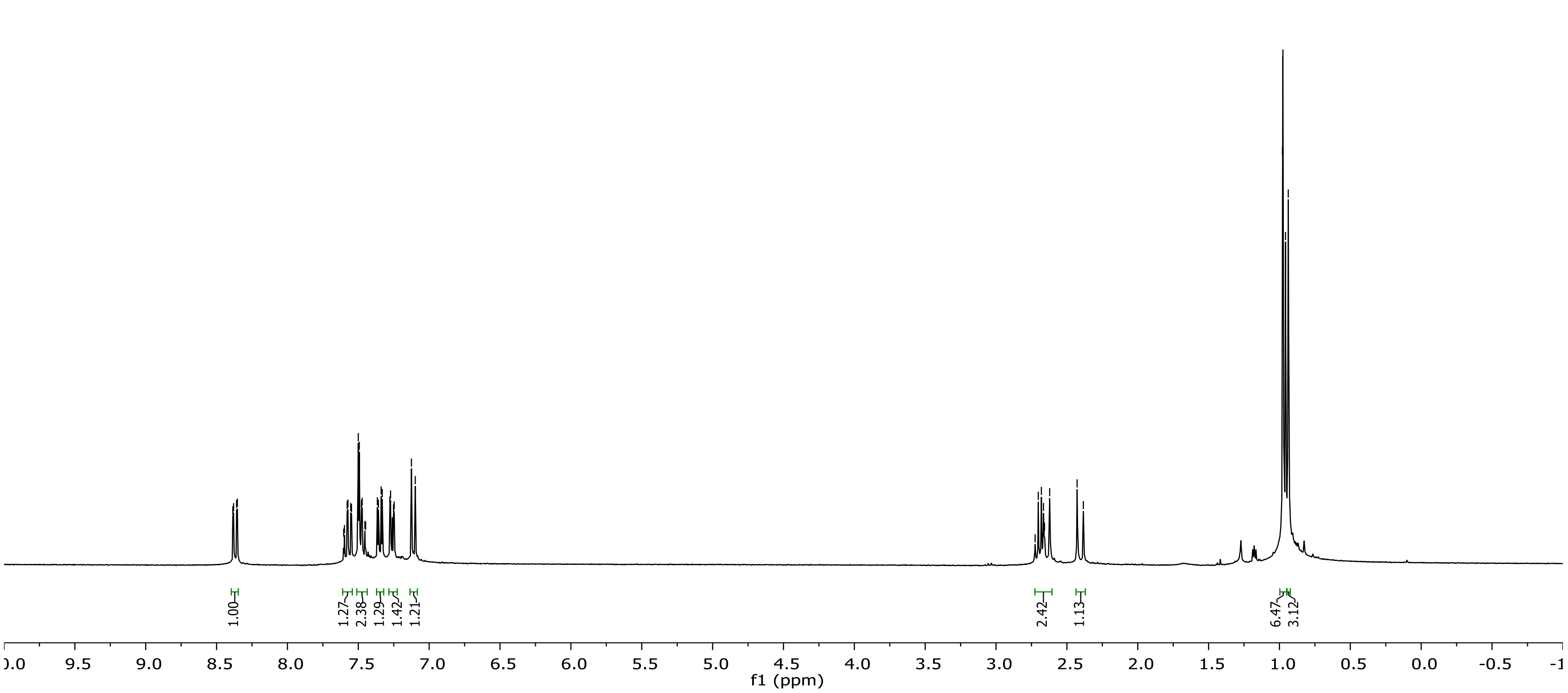

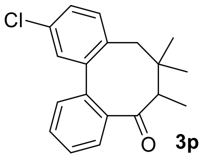

3p

^{13}C-NMR (75 MHz, $CDCl_3$)

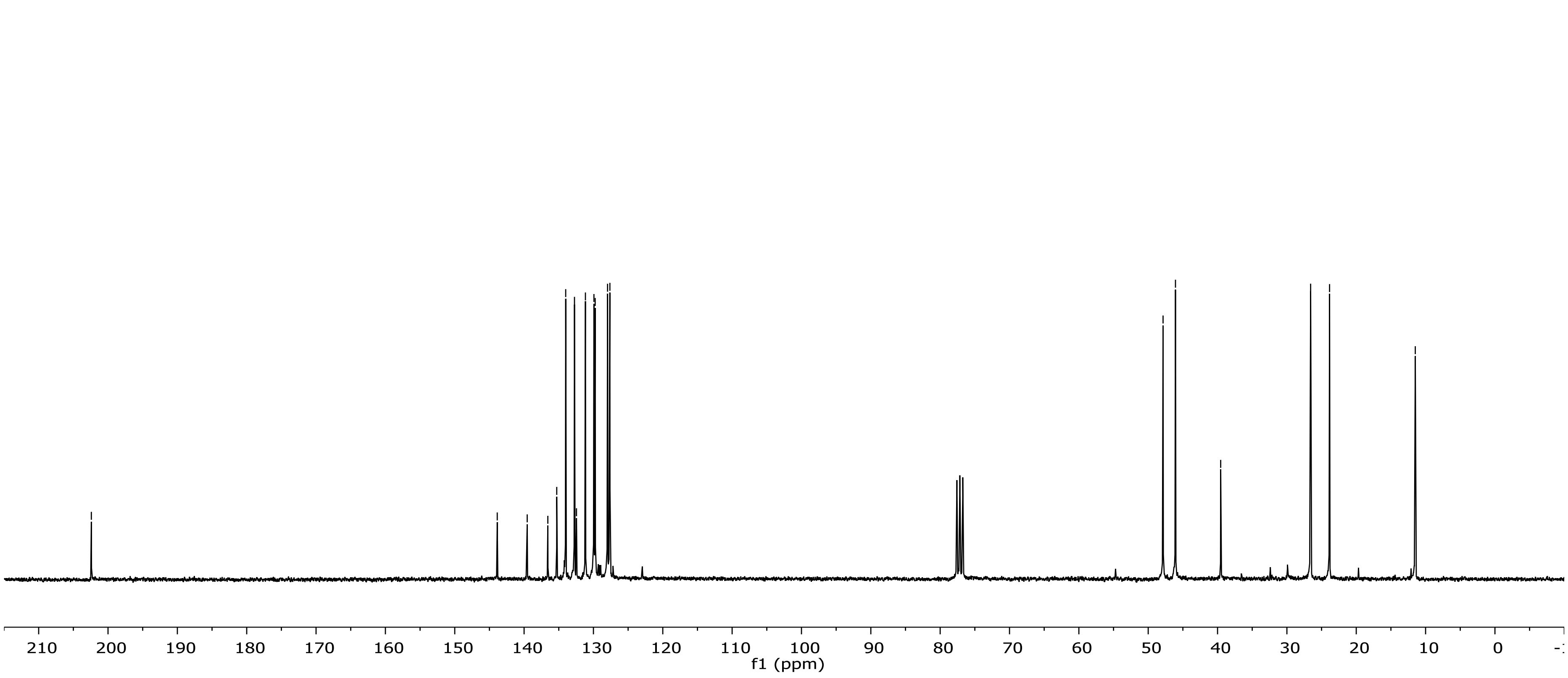

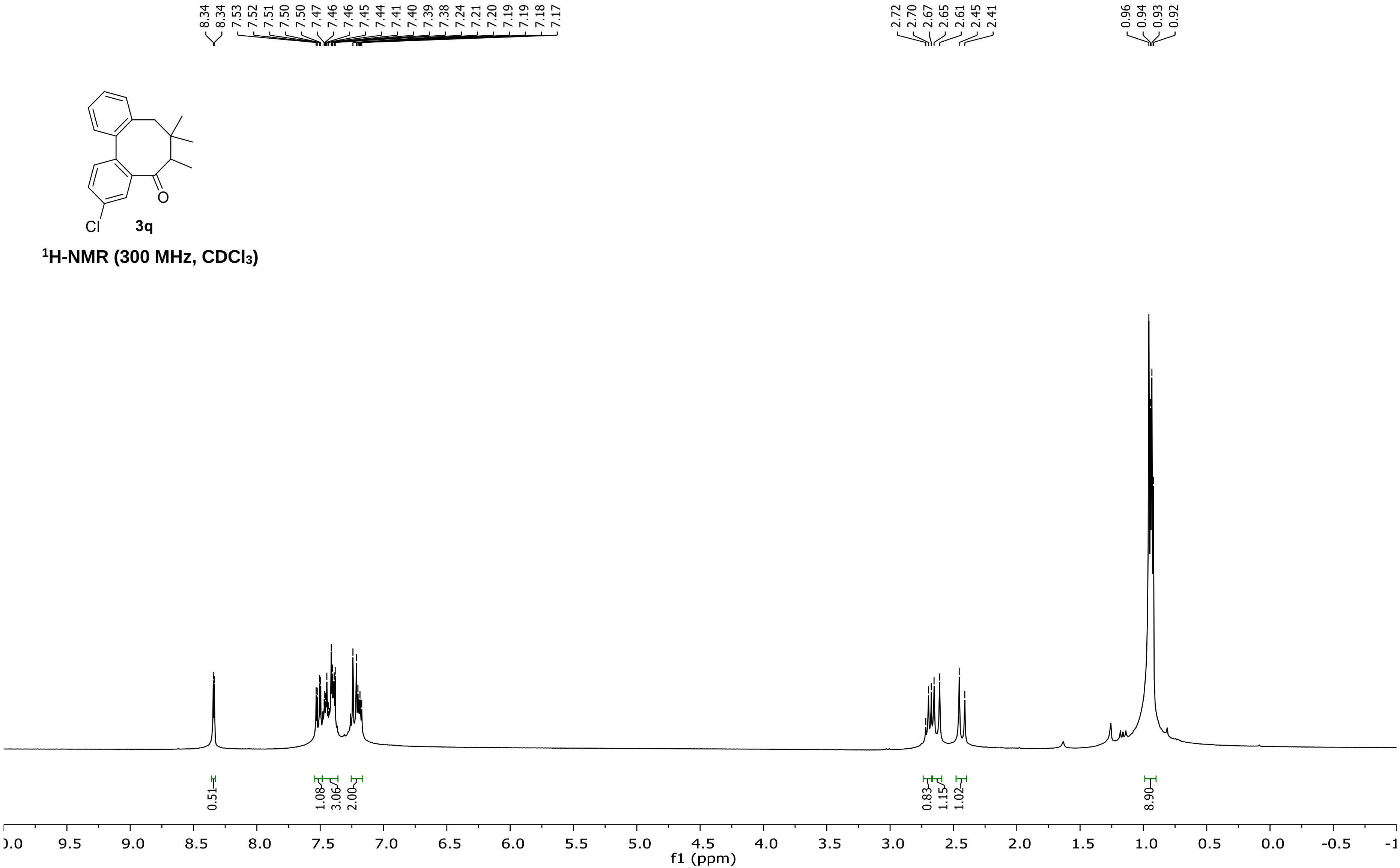
8.34
8.34
7.53
7.52
7.51
7.50
7.50
7.47
7.46
7.46
7.45
7.44
7.41
7.40
7.39
7.38
7.24
7.21
7.20
7.19
7.19
7.18
7.17
2.72
2.70
2.67
2.65
2.61
2.45
2.41
0.96
0.94
0.93
0.92
Cl
O
3q
¹H-NMR (300 MHz, CDCl₃)
0.51
1.08
3.06
2.00
0.83
1.15
1.02
8.90
f1 (ppm)

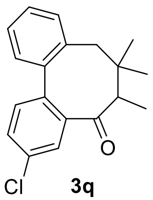

3q

^{13}C-NMR (75 MHz, $CDCl_3$)

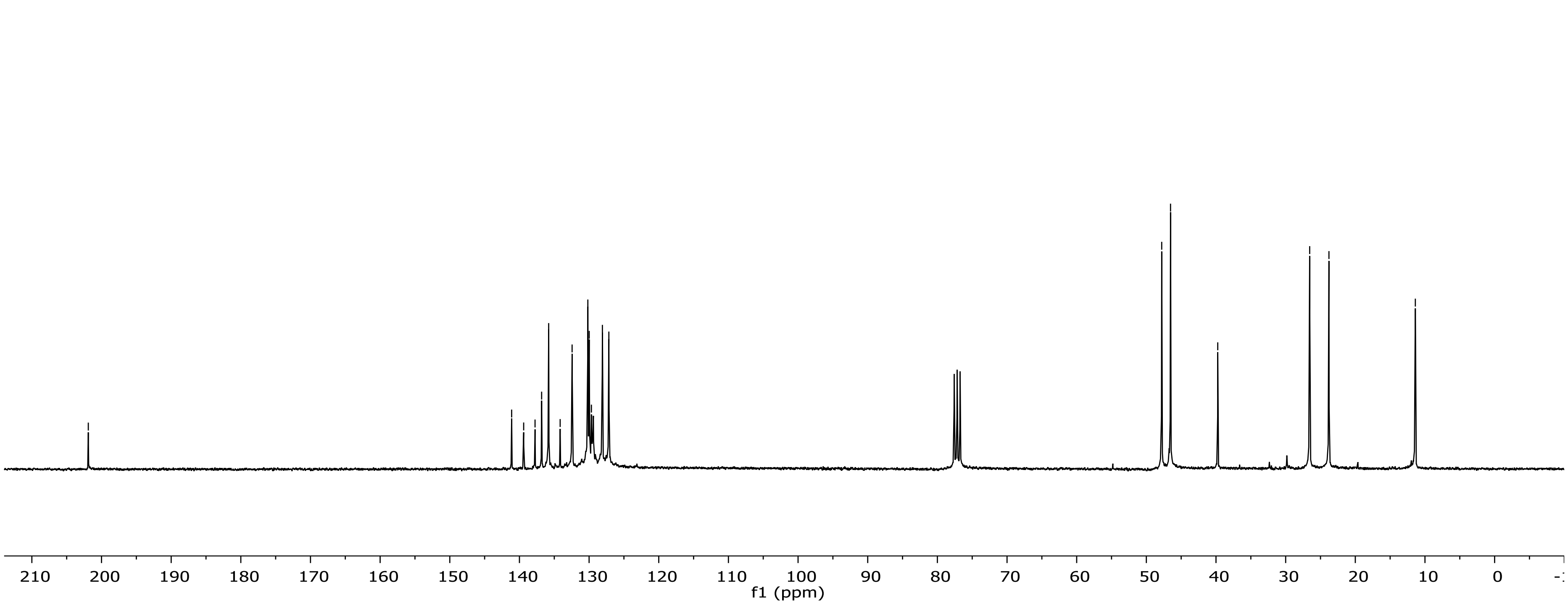

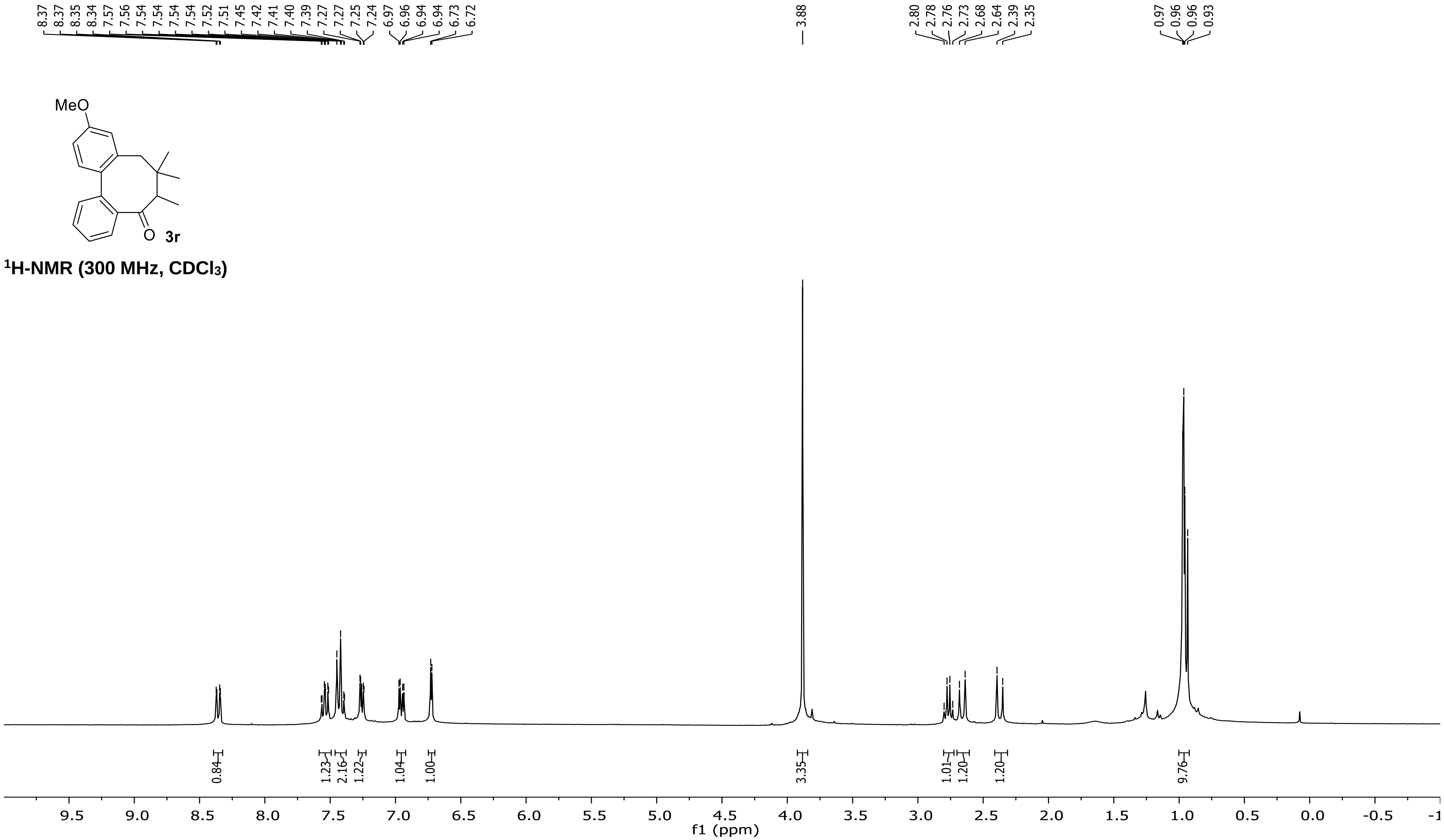

8.37 8.37 8.35 8.34 7.57 7.56 7.54 7.54 7.54 7.54 7.52 7.51 7.45 7.42 7.41 7.40 7.39 7.27 7.27 7.25 7.24 6.97 6.96 6.94 6.94 6.73 6.72
3.88
2.80 2.78 2.76 2.73 2.68 2.64 2.39 2.35
0.97 0.96 0.96 0.93
MeO
O
3r
^{1}H-NMR (300 MHz, $CDCl_3$)
0.84 1.23 2.16 1.22 1.04 1.00 3.35 1.01 1.20 1.20 9.76
9.5 9.0 8.5 8.0 7.5 7.0 6.5 6.0 5.5 5.0 4.5 4.0 3.5 3.0 2.5 2.0 1.5 1.0 0.5 0.0 -0.5 -1
f1 (ppm)

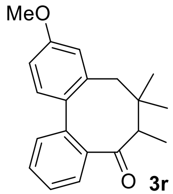

3r

^{13}C-NMR (75 MHz, $CDCl_3$)

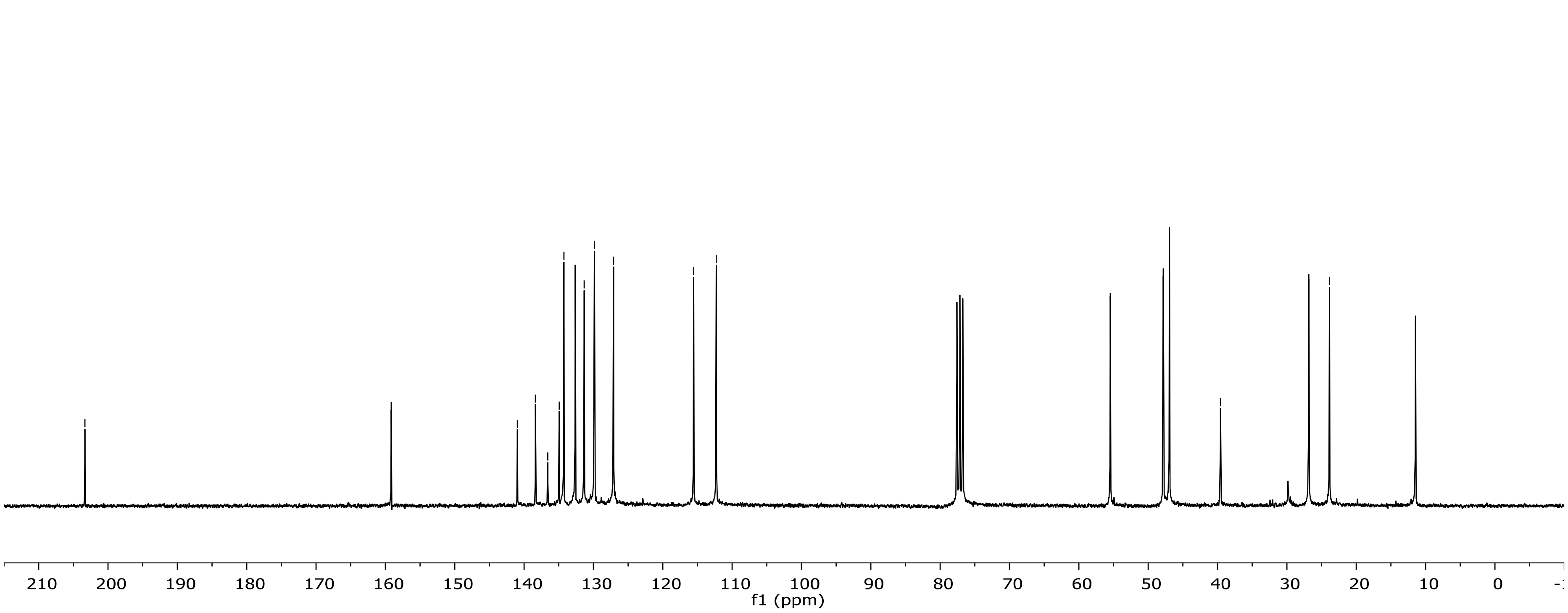

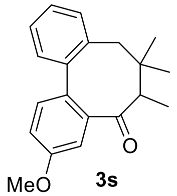

3s

^{1}H-NMR (300 MHz, CDCl$_3$)

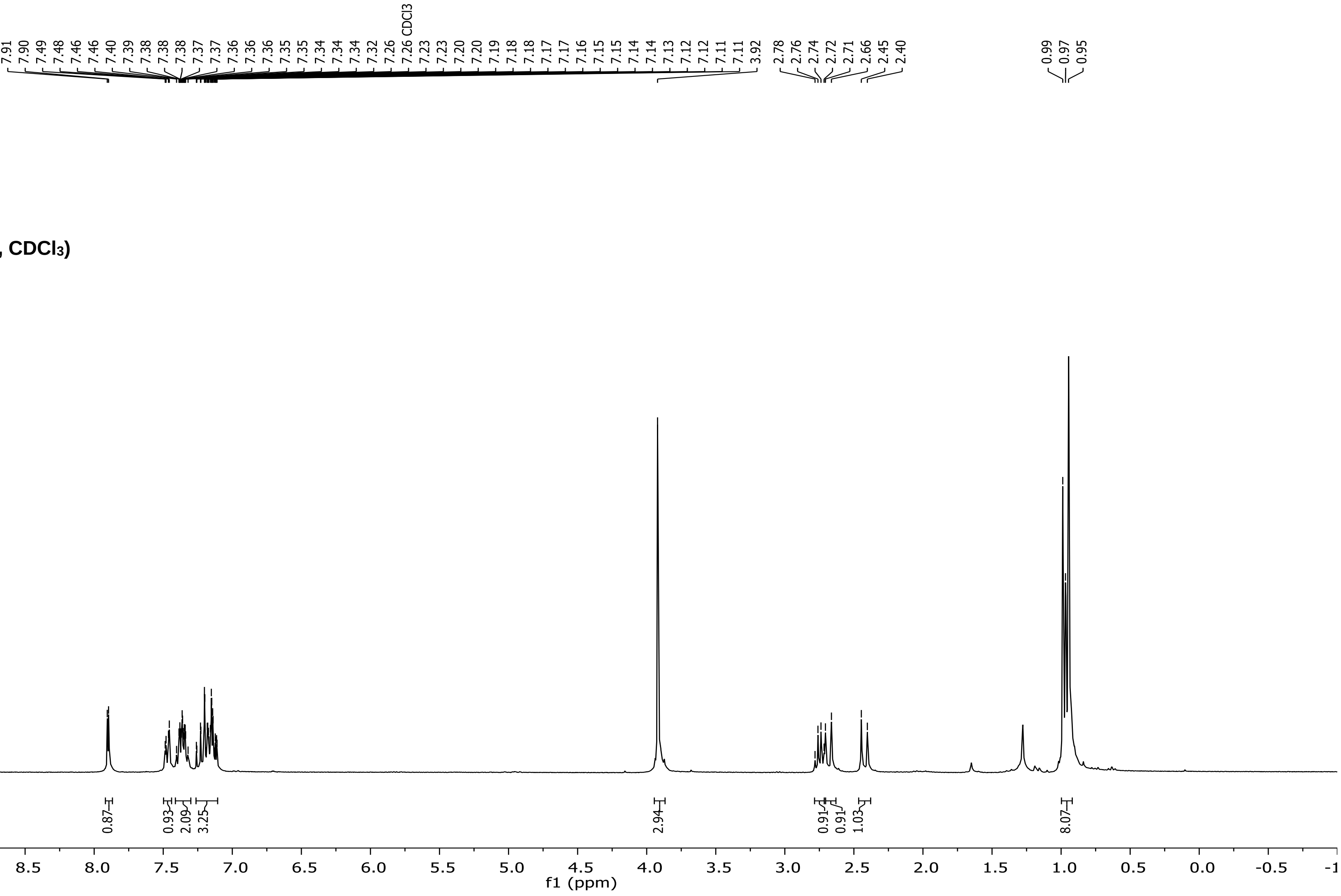

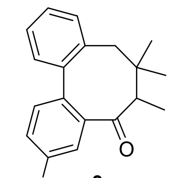

MeO 3s

^{13}C-NMR (75 MHz, CDCl$_3$)

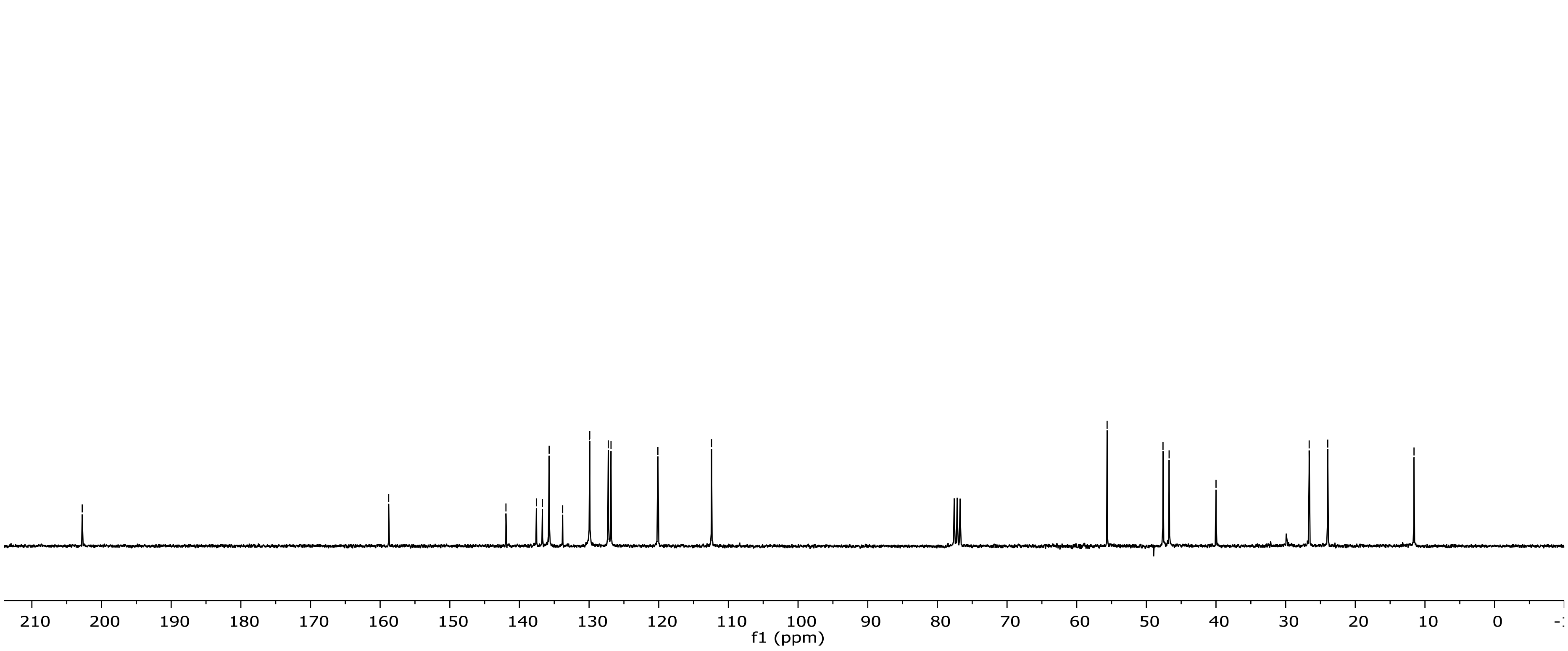

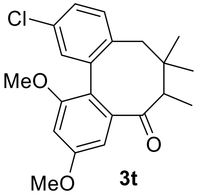

^{1}H-NMR (500 MHz, $CDCl_3$)

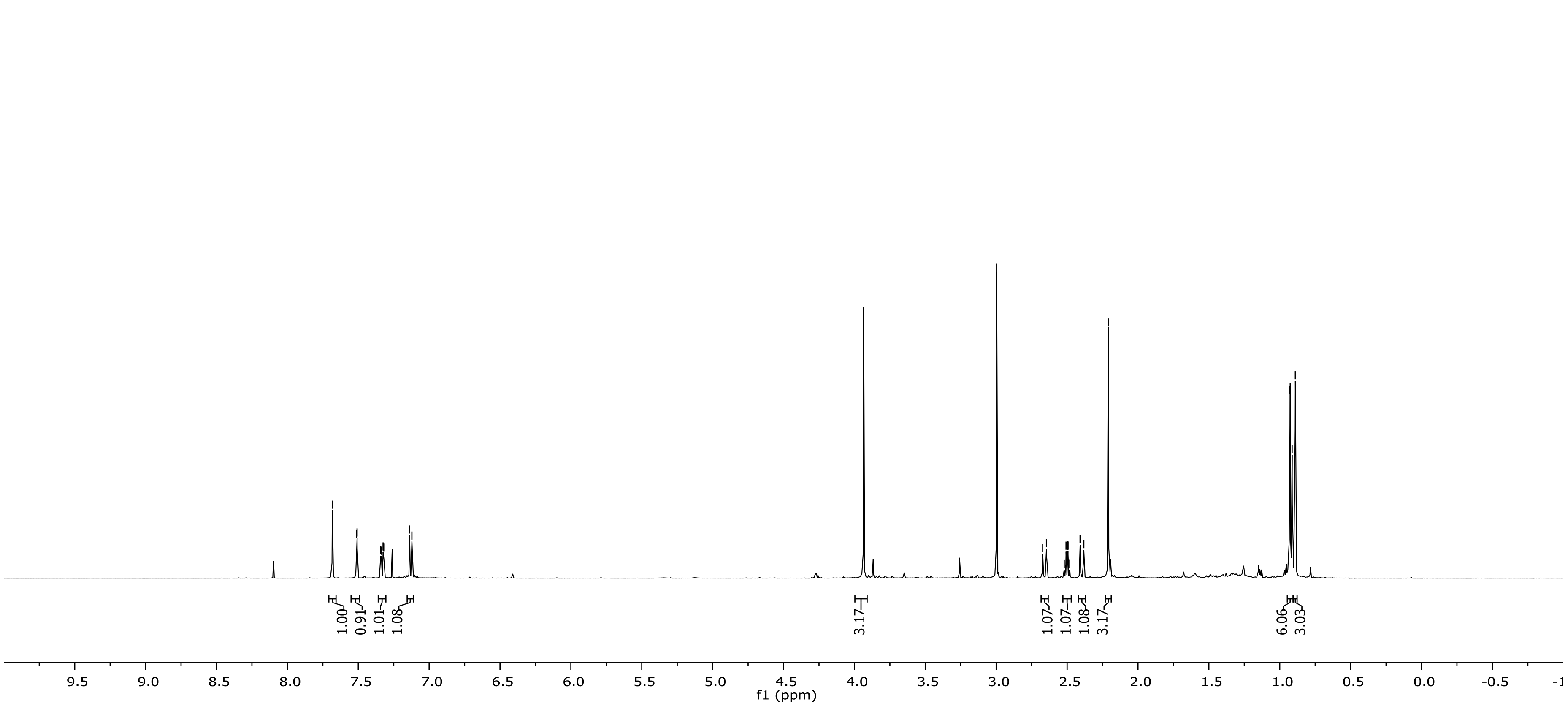

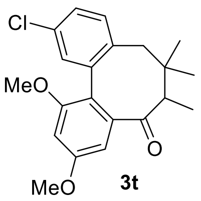

3t

^{13}C-NMR (125 MHz, CDCl$_3$)

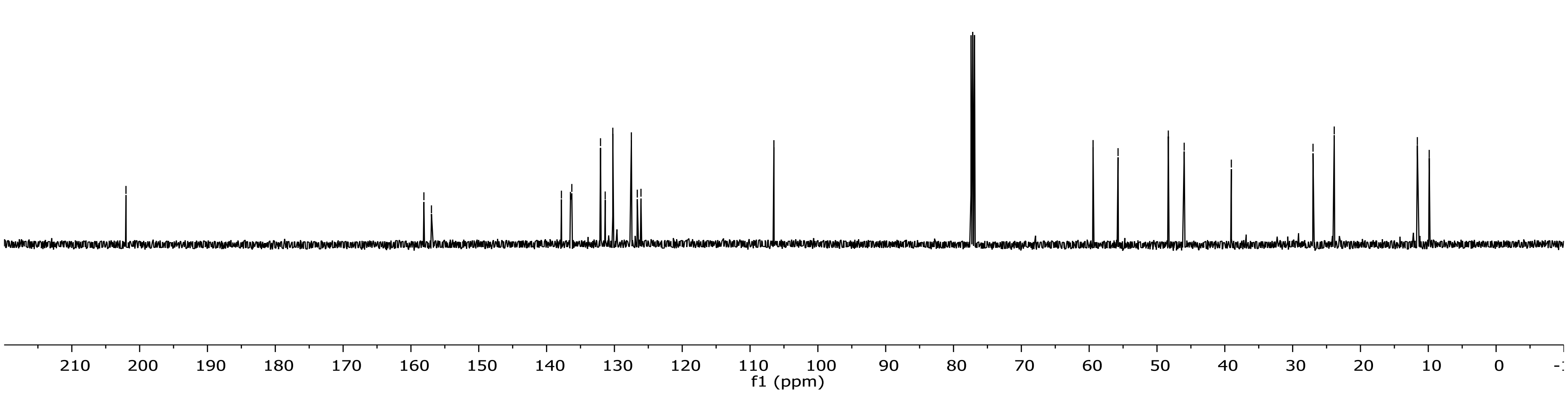

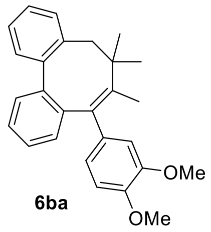

6ba

^{1}H-NMR (400 MHz, $CDCl_3$)

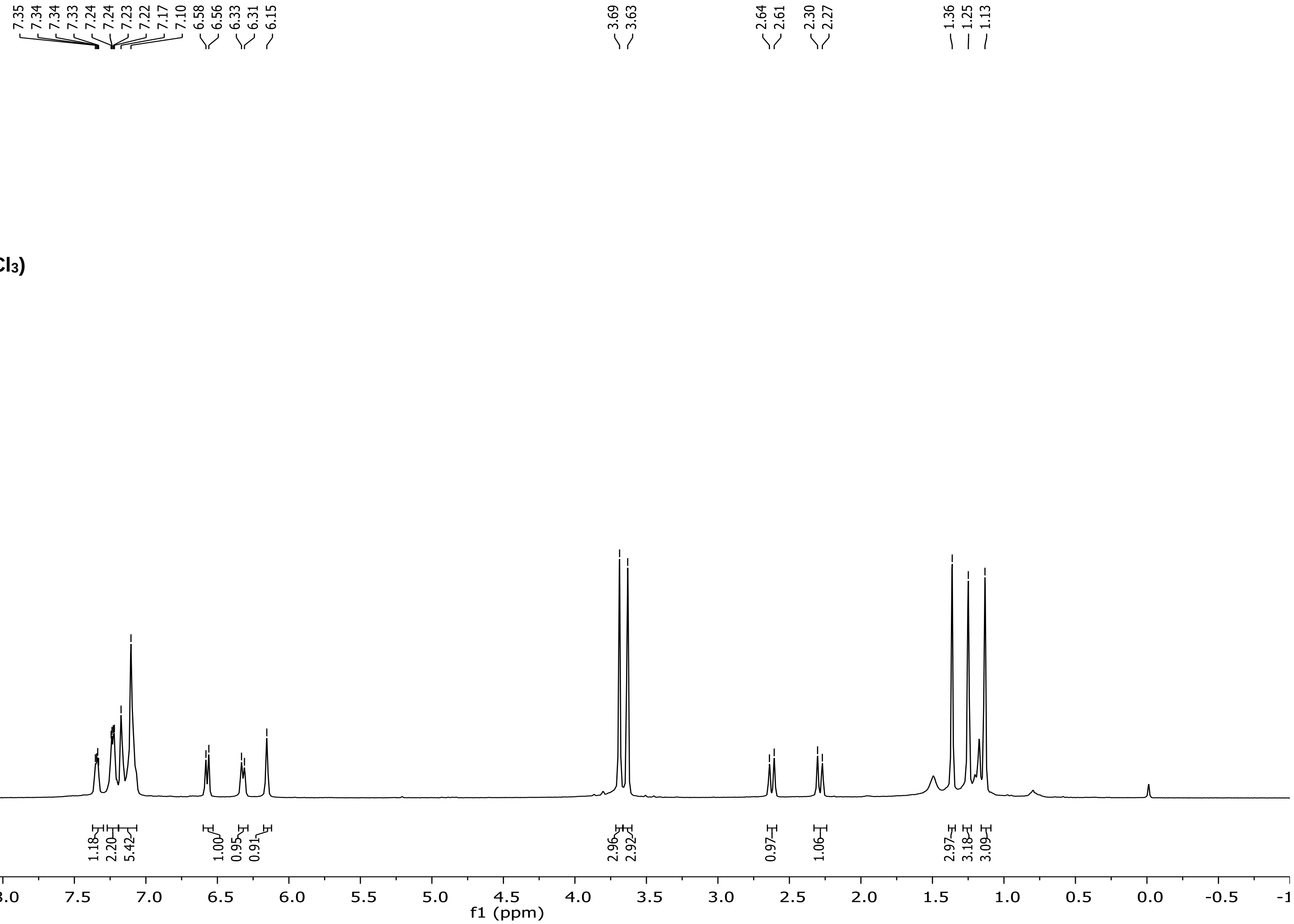

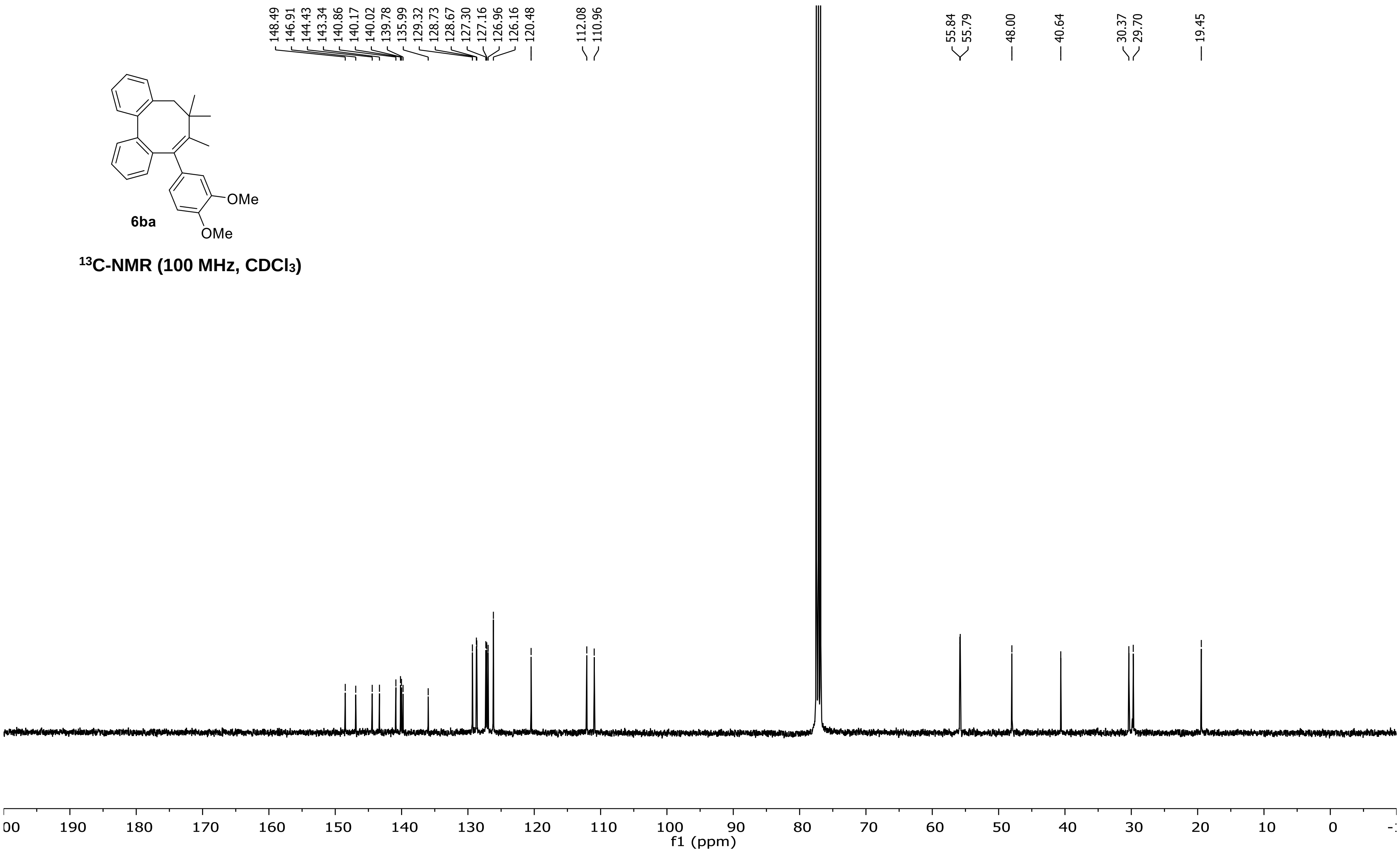
148.49
146.91
144.43
143.34
140.86
140.17
140.02
139.78
135.99
129.32
128.73
128.67
127.30
127.16
126.96
126.16
120.48
112.08
110.96
55.84
55.79
48.00
40.64
30.37
29.70
19.45
OMe
OMe
6ba
13C-NMR (100 MHz, CDCl3)
190
180
170
160
150
140
130
120
110
100
90
80
70
60
50
40
30
20
10
0
f1 (ppm)

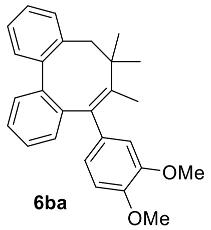
6ba

gCOSY-NMR

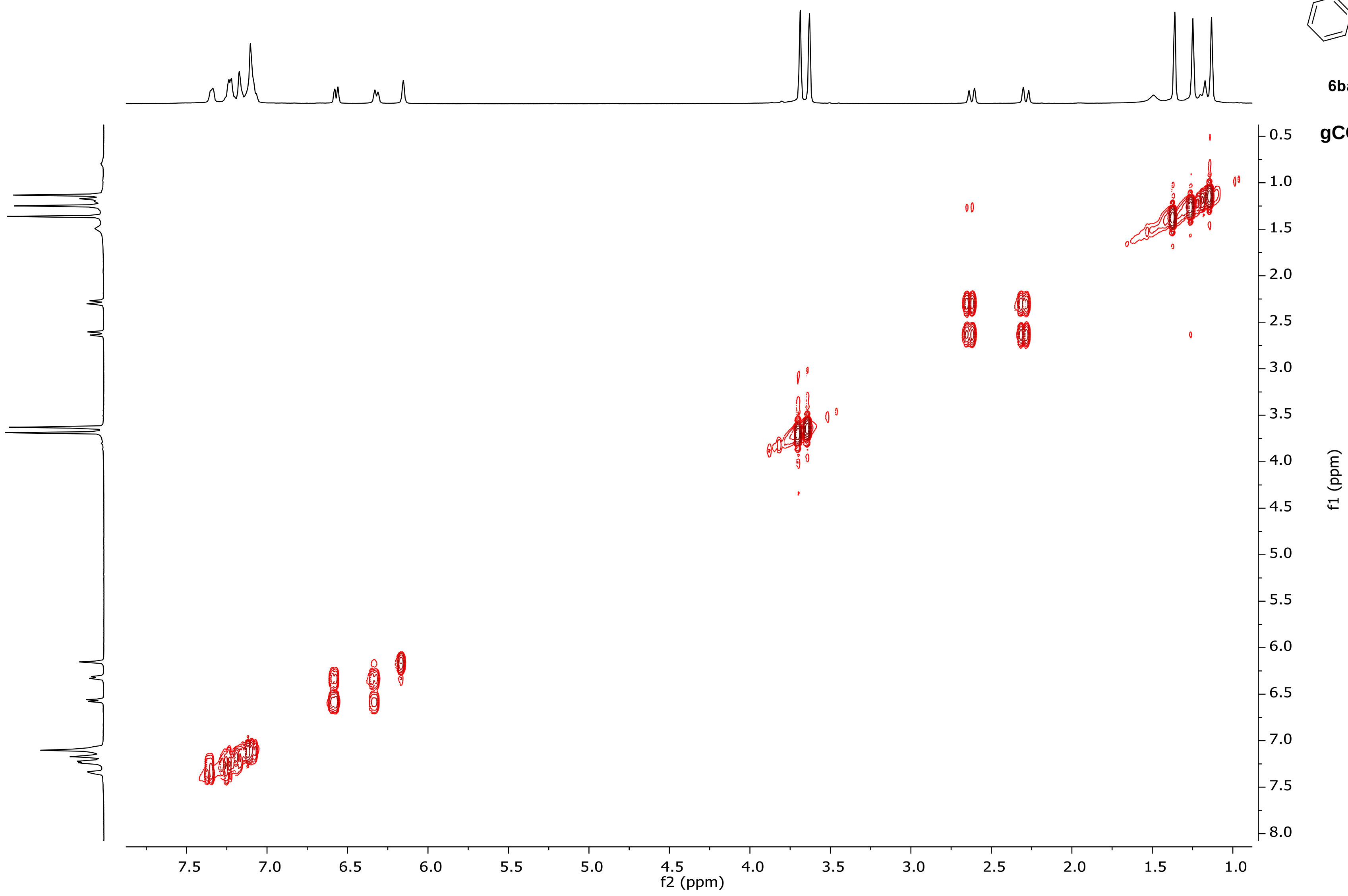

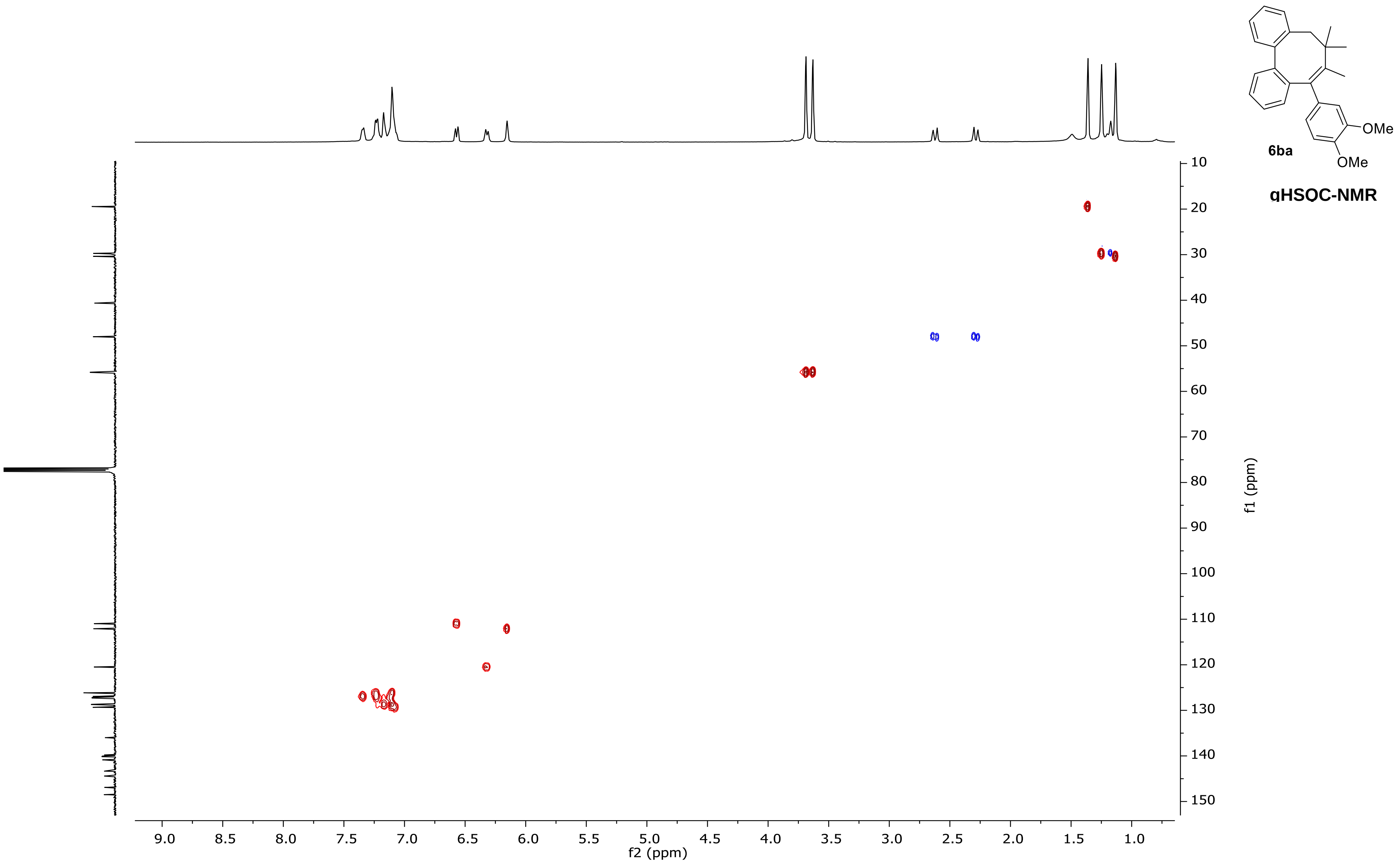
6ba
OMe
OMe
qHSQC-NMR
f2 (ppm)
f1 (ppm)

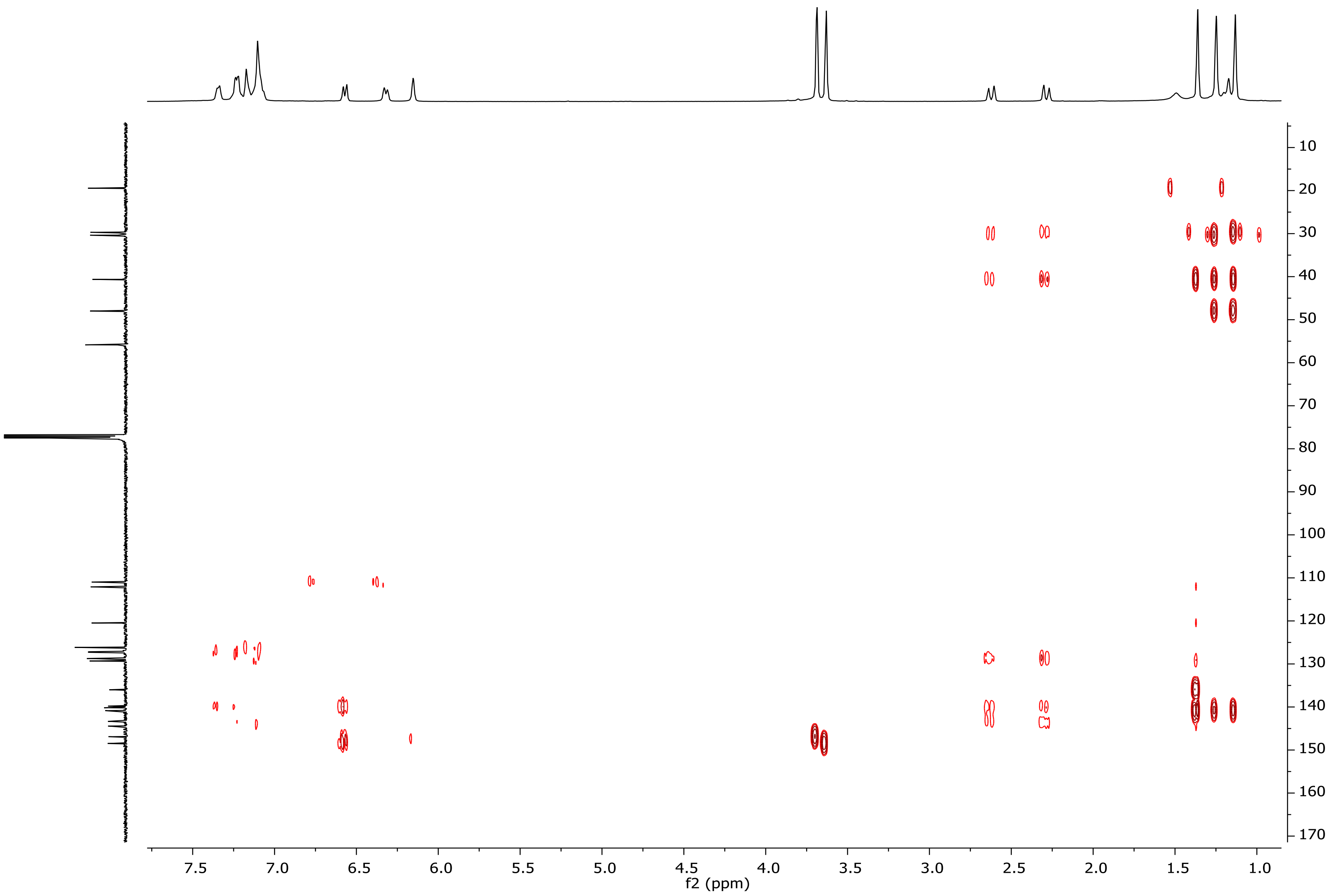

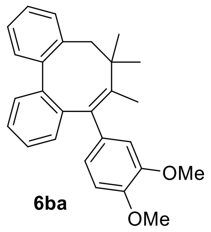

6ba

gHMBC-NMR

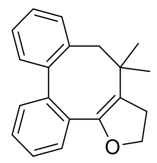

6u

^{1}H-NMR (500 MHz, CDCl$_3$)

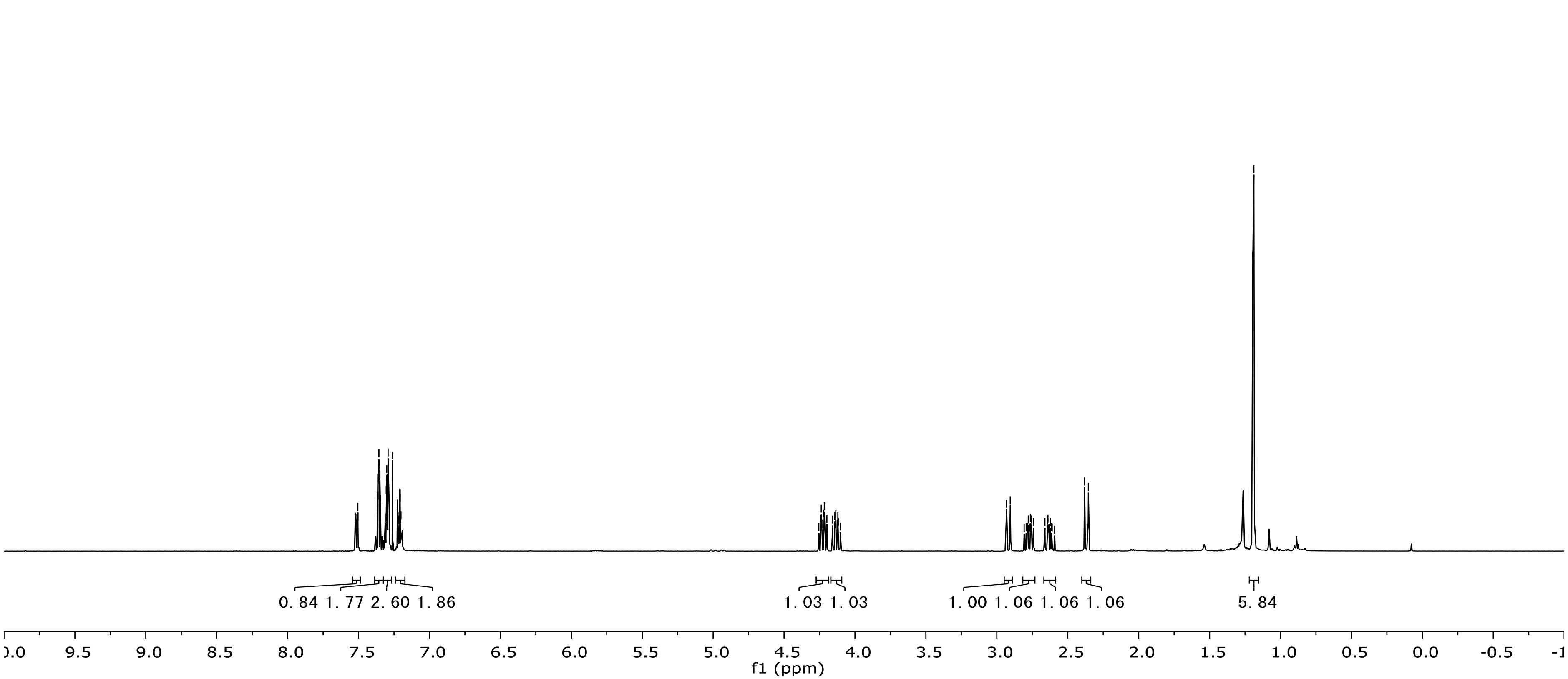

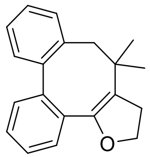

6u

^{13}C-NMR (125 MHz, CDCl$_3$)

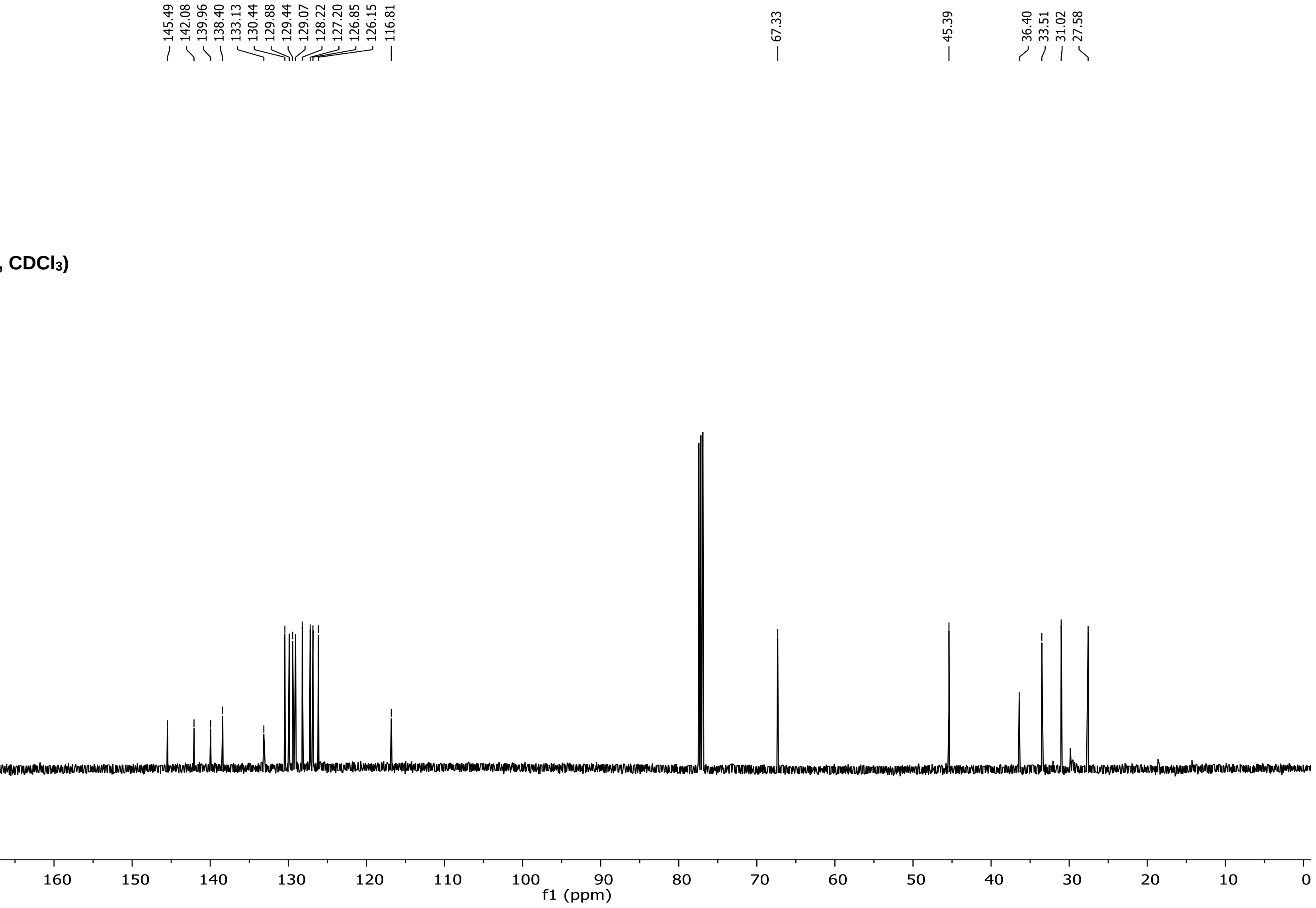

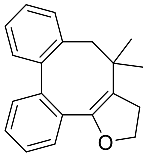

6u

gCOSY-NMR

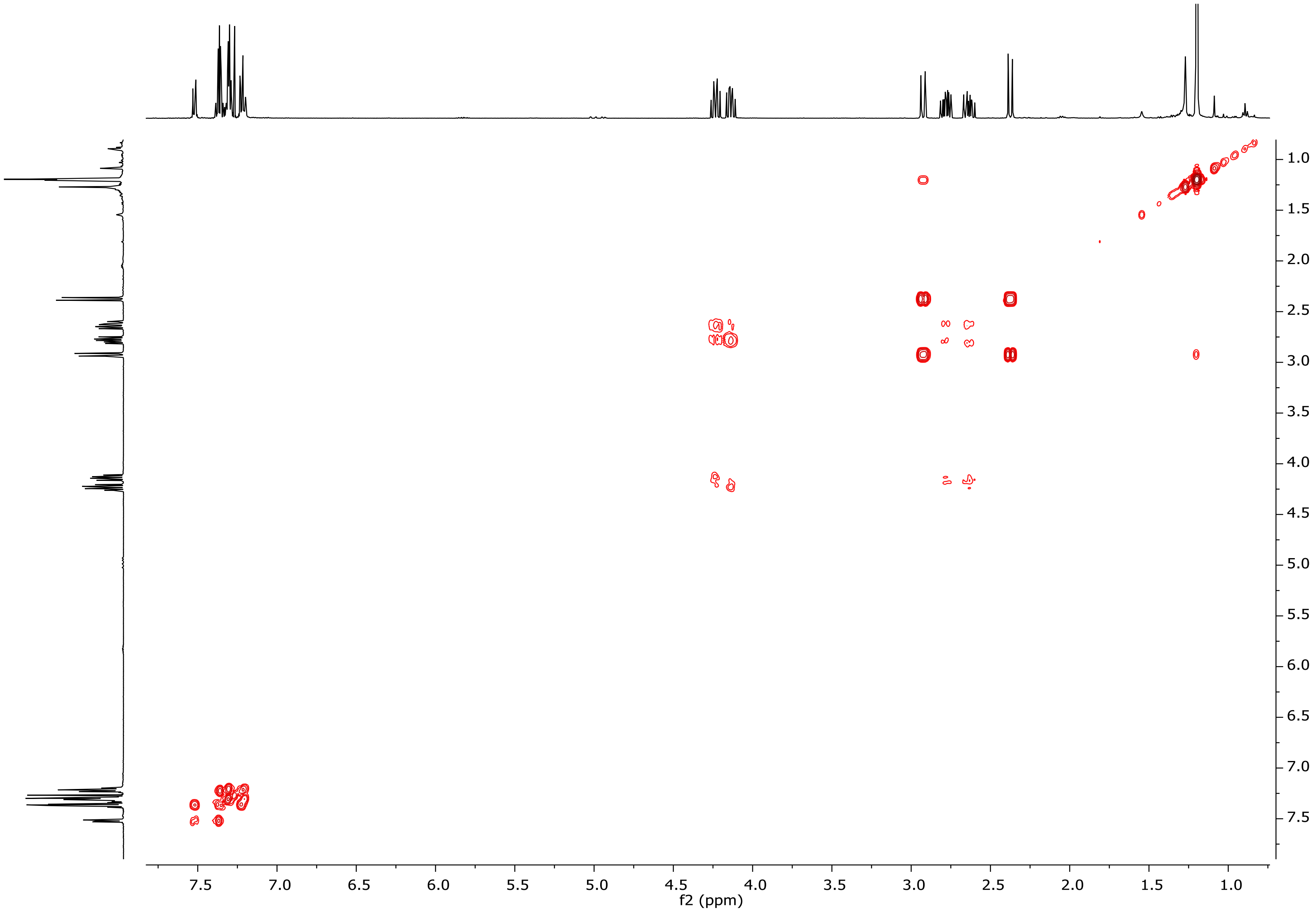

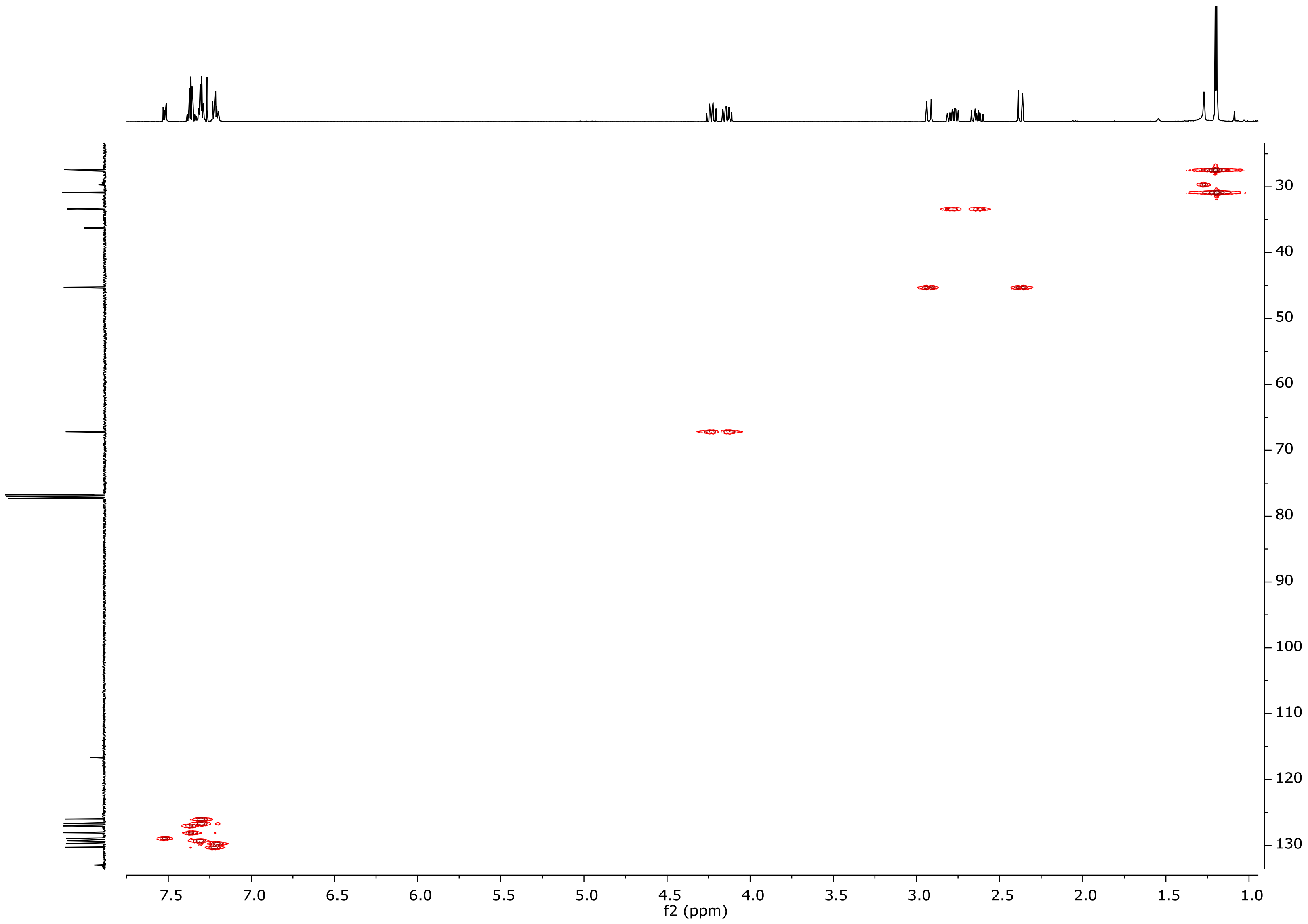

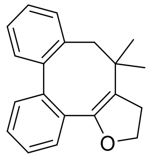

6u

qHSQC-NMR

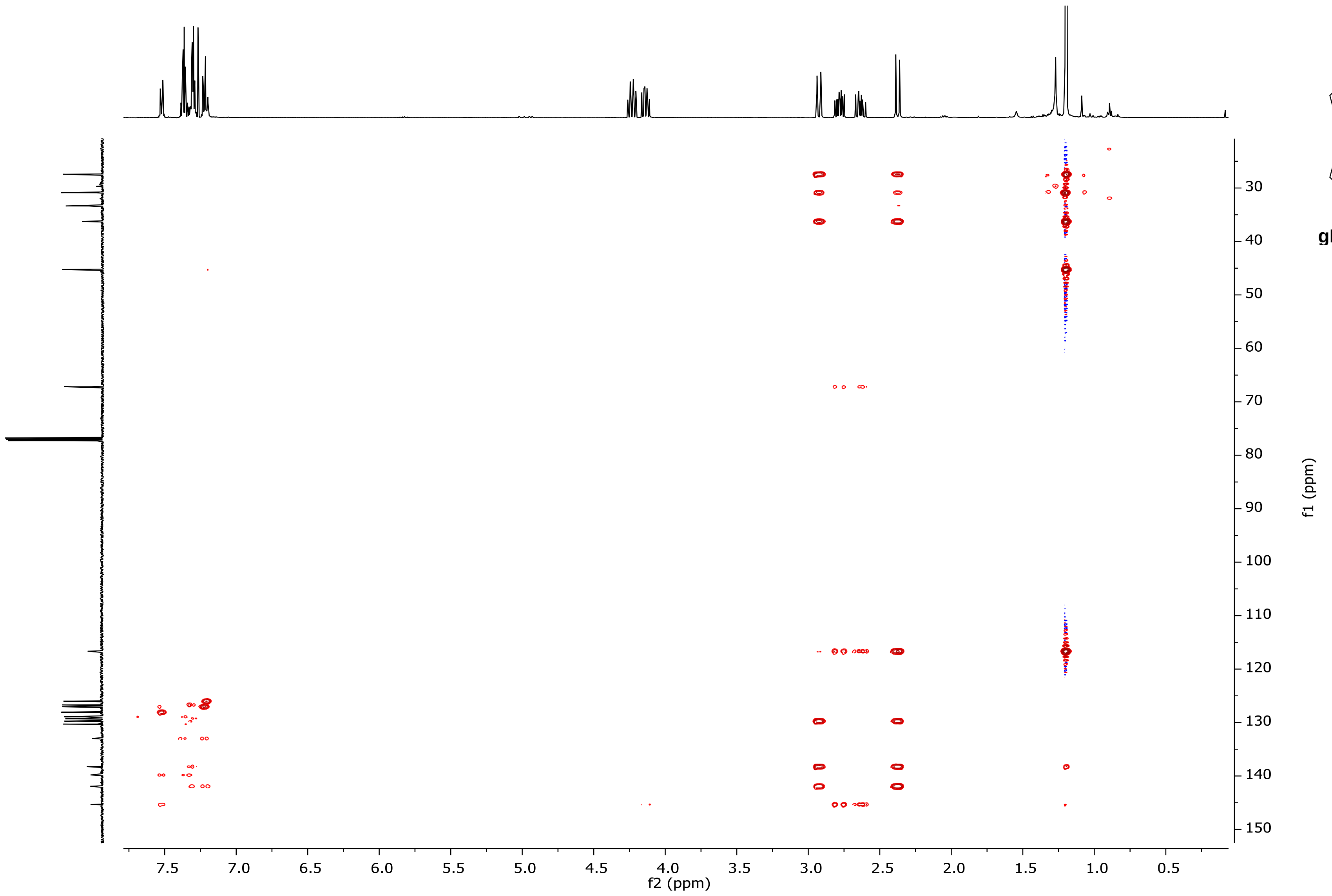

7.5
7.0
6.5
6.0
5.5
5.0
4.5
4.0
3.5
3.0
2.5
2.0
1.5
1.0
0.5
f2 (ppm)
30
40
50
60
70
80
90
100
110
120
130
140
150
f1 (ppm)

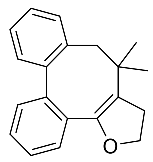

O

6u

gHMBC-NMR

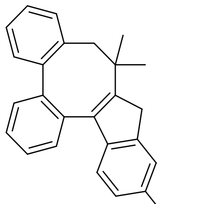

6v

^{1}H-NMR (400 MHz, CDCl$_3$)

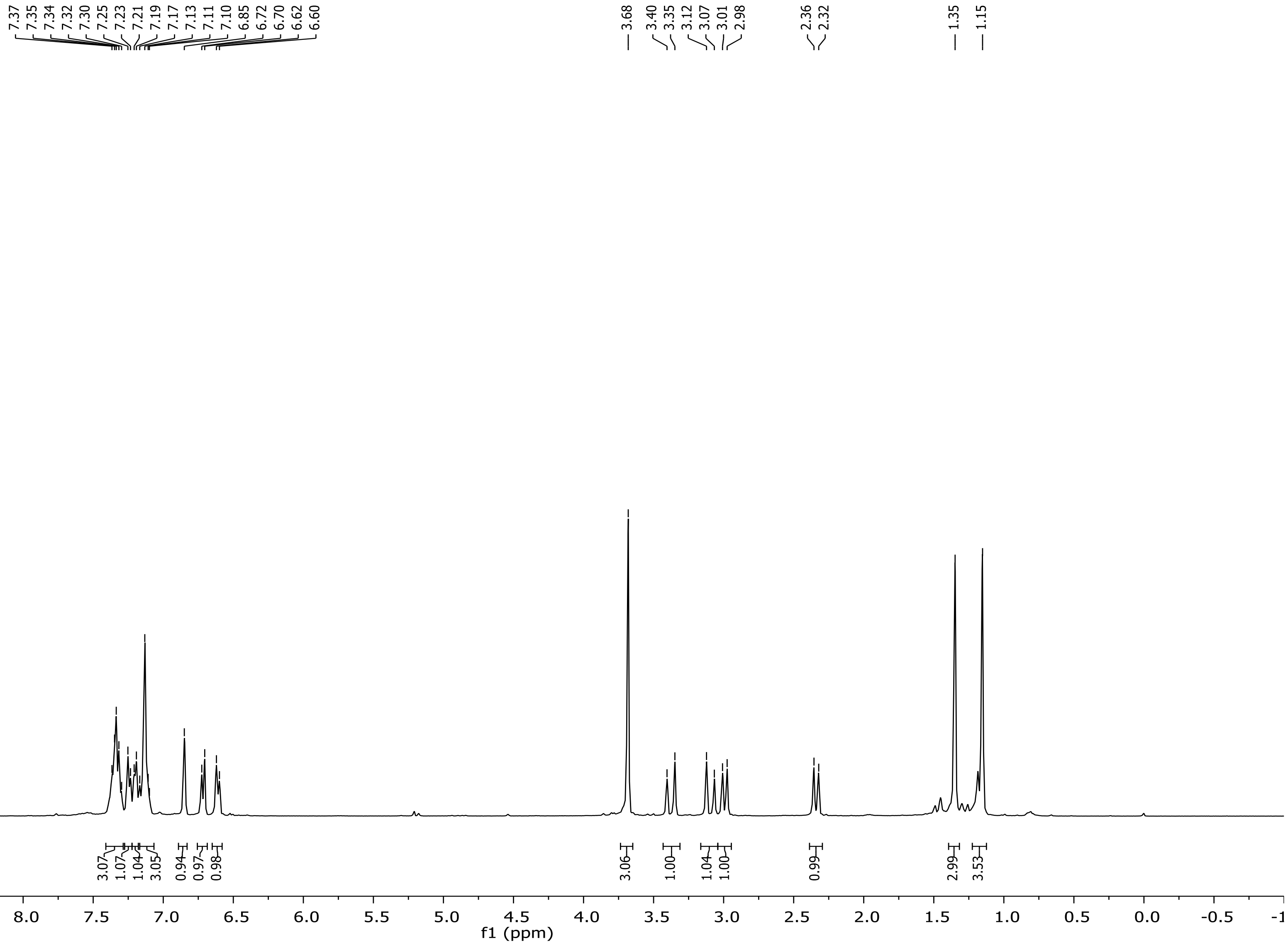

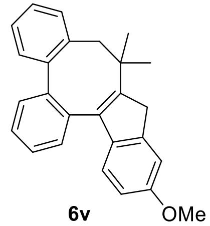

6v

^{13}C-NMR (100 MHz, CDCl$_3$)

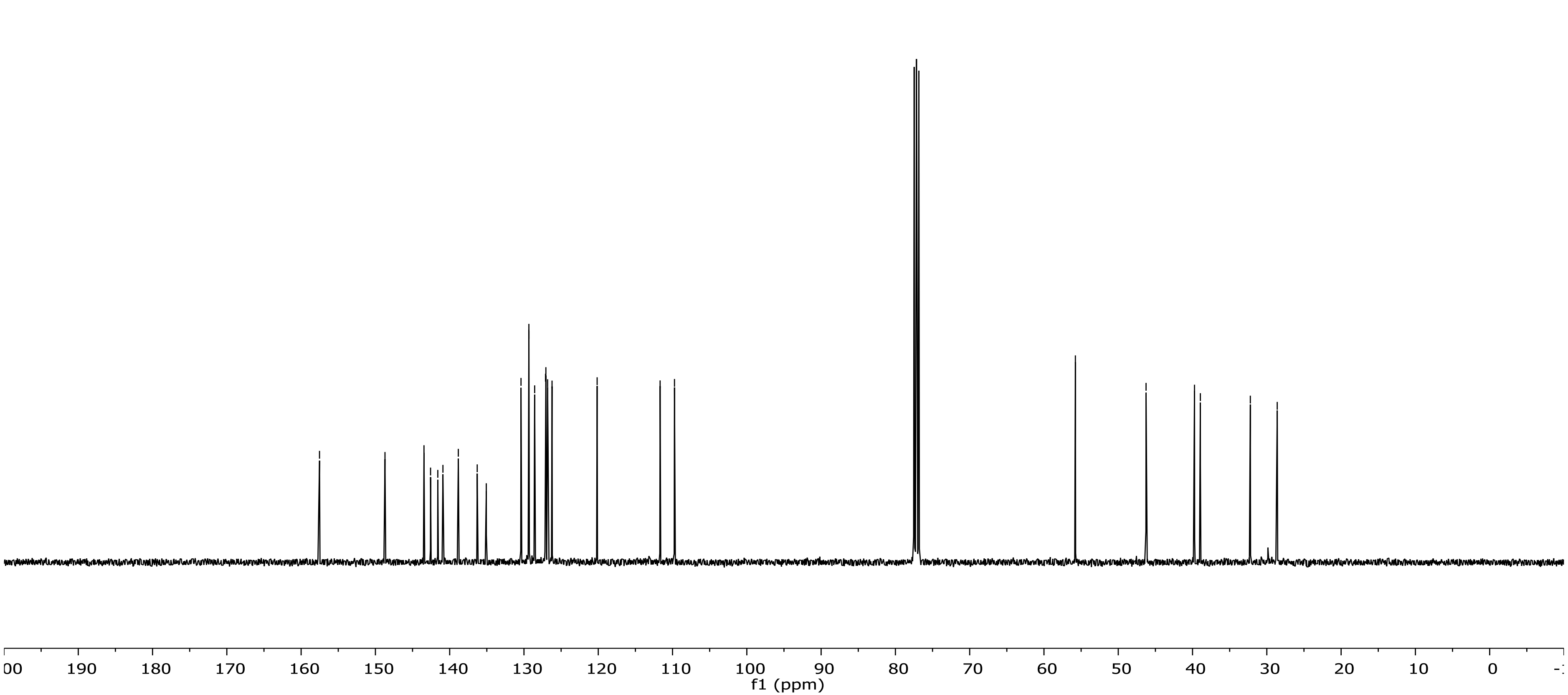

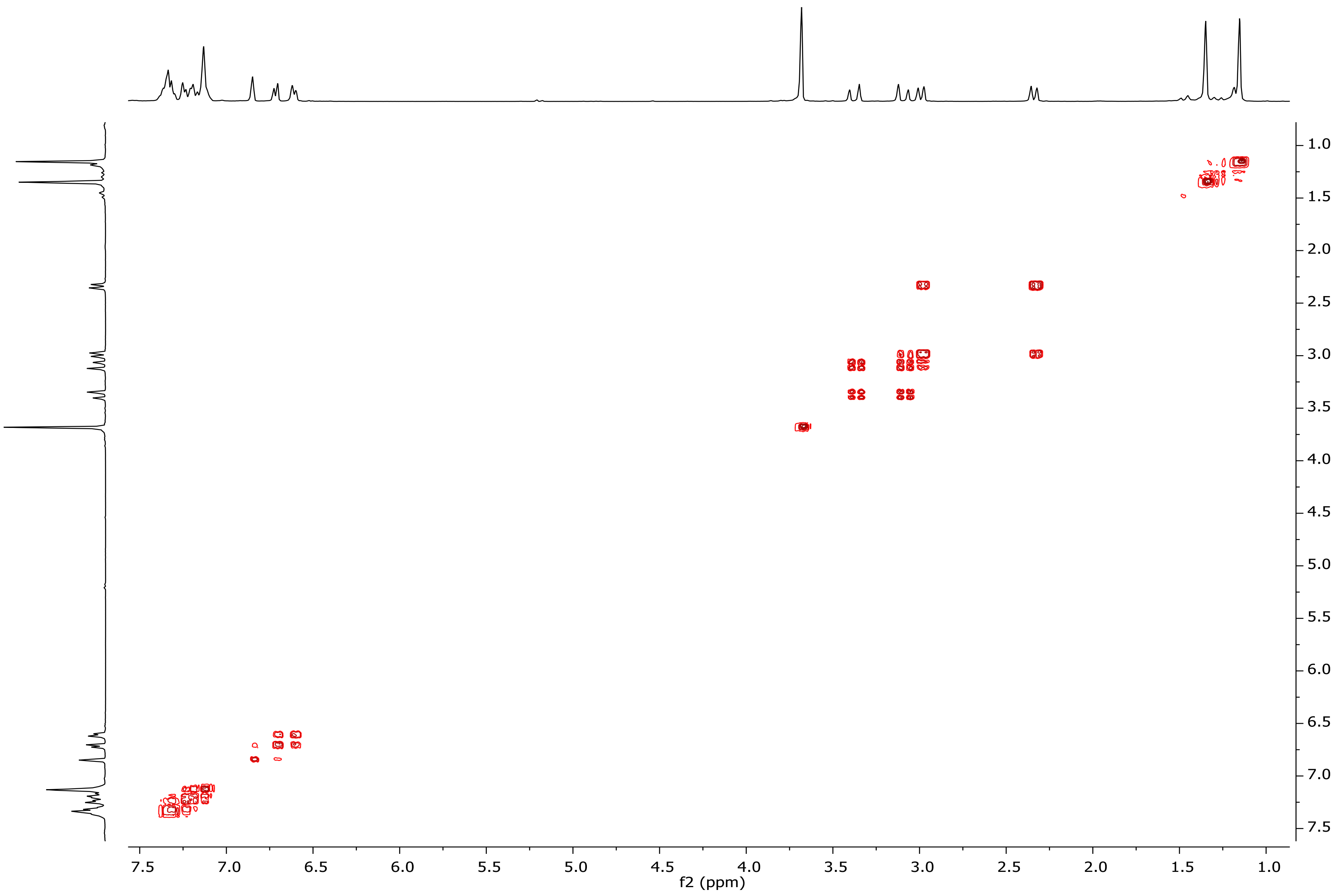

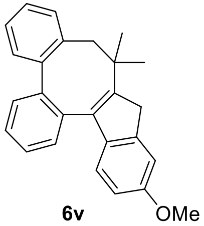

6v

gCOSY-NMR

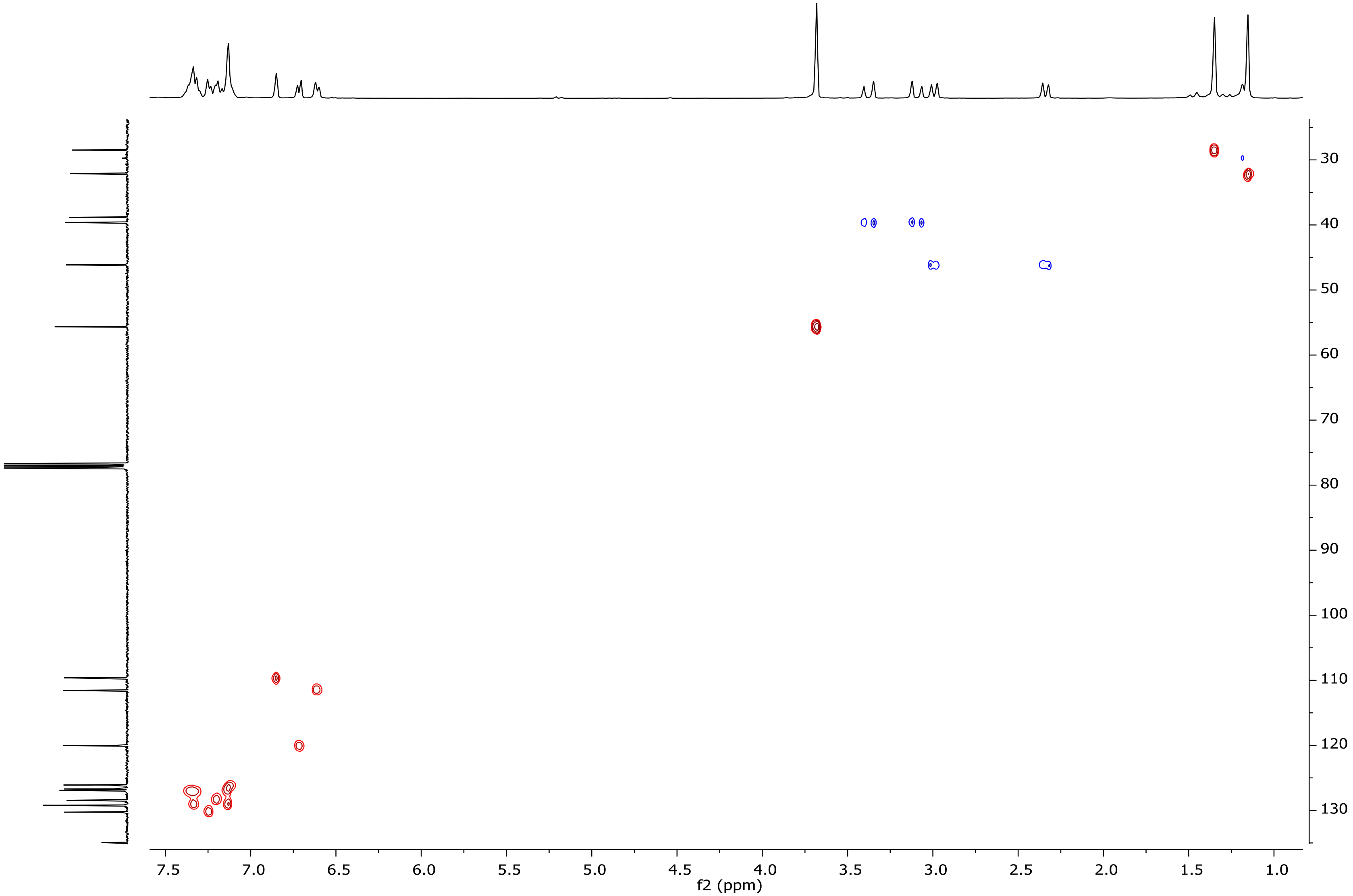

7.5
7.0
6.5
6.0
5.5
5.0
4.5
4.0
3.5
3.0
2.5
2.0
1.5
1.0
f2 (ppm)
30
40
50
60
70
80
90
100
110
120
130
f1 (ppm)

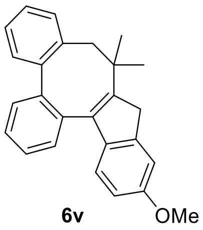

OMe

6v

αHSOC-NMR

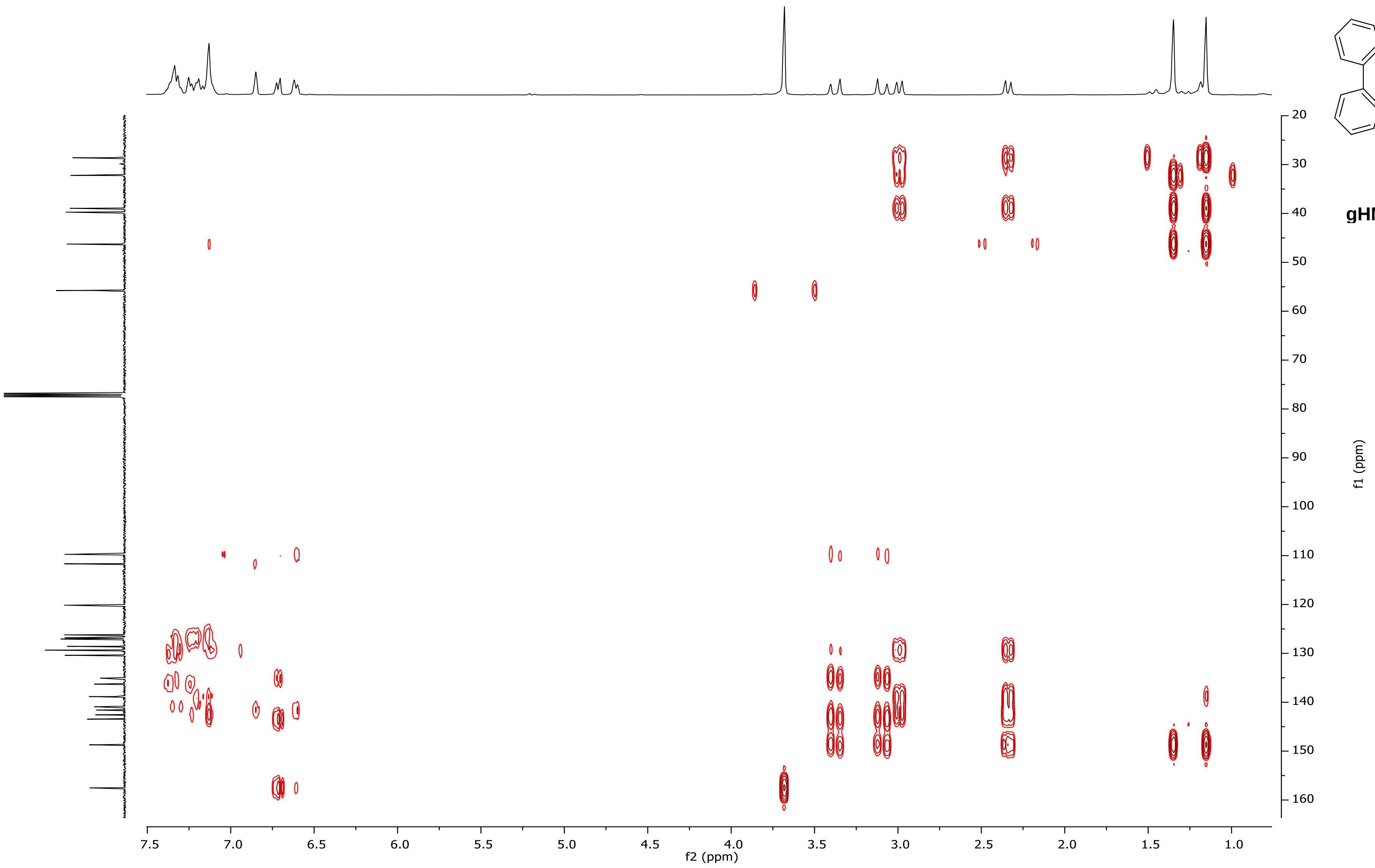

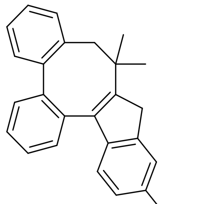

6v

gHMBC-NMR

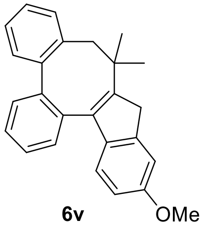

6v

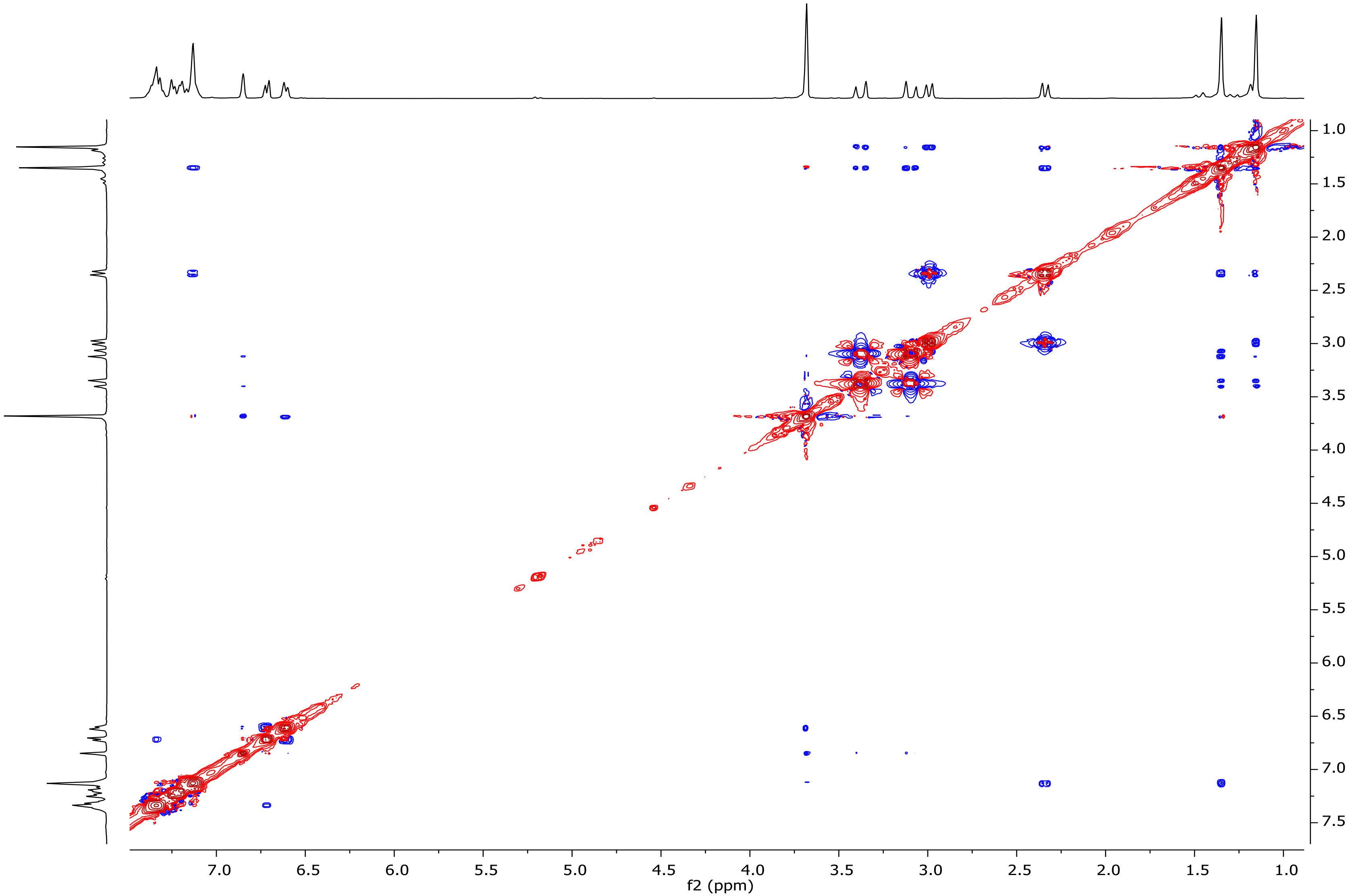

gNOESY-NMR

Crystallographic data for 2a, 3q, 4ae and 4ca:

Colorless crystals of **2a, 3q, 4ae** and **4ca** were grown by slow evaporation at room temperature from a solution of the compound in a mixture of *n*-hexane and dichloromethane.

Crystallographic data of 2a are presented in Tables S5-12. A single crystal of **2a** was coated in high-vacuum grease and mounted on a glass fibre. X-ray measurements were made using a Bruker D8 VENTURE PhotonIII area-detector diffractometer with Mo-Kα radiation (α = 0.71073 Å). Absorption corrections were applied, based on multiple and symmetry-equivalent measurements. The structure was solved by ShelXT structure solution program using Intrinsic Phasing and refined with the XL refinement package using Least Squares minimization.[7] All non-hydrogen atoms were assigned anisotropic displacement parameters and refined without positional constraints and all other hydrogen atoms were constrained to ideal geometries and refined with fixed isotropic displacement parameters. Refinement proceeded smoothly to give the residuals shown in Table S6. A colorless prism-like specimen of $C_{24}H_{22}O$, approximate dimensions 0.100 mm x 0.100 mm x 0.600 mm, mounted on a Mitegen Micromount, was used for the X-ray crystallographic analysis. The X-ray intensity data were measured (λ = 71073 Å).

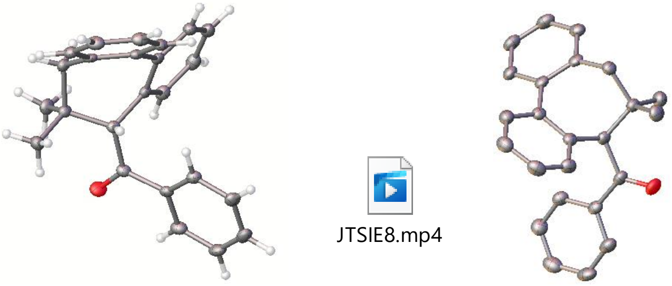

Table S5: Data collection details for 2a.

Axis	dx/mm	2θ/°	ω/°	φ/°	χ/°	Width/°	Frames	Time/s	Wavelength/Å	Voltage/kV	Current/mA	Temperature/K
Omega	60.004	-28.0	-20.0	0.00	45.00	0.50	240	2.00	0.71073	50	1.4	100
Omega	60.004	-28.0	-20.0	90.00	45.00	0.50	240	2.00	0.71073	50	1.4	100
Omega	60.004	28.00	36.00	180.00	45.00	0.50	240	2.00	0.71073	50	1.4	100
Omega	60.004	28.00	36.00	270.00	45.00	0.50	240	2.00	0.71073	50	1.4	100
Phi	60.004	0.00	0.00	0.00	45.00	1.00	180	1.00	0.71073	50	1.4	100

A total of 1140 frames were collected. The total exposure time was 0.58 hours. The frames were integrated with the Bruker SAINT software package using a narrow-frame algorithm. The integration of the data using a triclinic unit cell yielded a total of 20433 reflections to a maximum θ angle of 36.76 (0.59 Å resolution). The final cell constants of a = 9.0025(5) Å, b = 9.8912(5) Å, c = 11.9895(6) Å, α = 93.167(2)°, β = 108.062(2)°, γ = 116.662(2)°, volume = 883.02(11) $Å^3$, are based upon the refinement of the XYZ-centroids of 7452 reflections above 20 σ(I) with 5.285 < 2θ < 73.28. Data were corrected for absorption effects using the Multi-Scan method (SADABS). The ratio of minimum to maximum apparent transmission was 0.698.

[7] a) Sheldrick, G.M. (2015). Acta Cryst A71, 3-8; APEX3 Version 2016.7 (Bruker AXS Inc.) Bruker Instrument Service vV6.2.10. b) SAINT integration software, SAINT V8.38A (Bruker AXS Inc., 2017). c) SADABS-2016/2 - Bruker AXS area detector scaling and absorption correction (Sheldrick, Bruker AXS Inc.). d) *SHELXTL program system version 6.1;* XPREP Version 2013/3 (Sheldrick, Bruker AXS Inc.) XS Version 2013/1 (George M. Sheldrick, *Acta Cryst.* (2008). A**64**, 112-122). d) *International Tables for Crystallography*, Kluwer, Dordrecht, 1992, vol. C.

Table S6 Crystal data and structure refinement for 2a.

Empirical formula / Formula weight	$C_{24}H_{22}O$ / 326.41
Temperature/K	99.95
Crystal system / Space group	Triclinic / P-1
a/Å	9.0025(5)
b/Å	9.8912(5)
c/Å	11.9895(6)
α/°	93.167(2)
β/°	108.062(2)
γ/°	116.662(2)
Volume/Å^3	883.02(8)
Z	2
ρ_{calc}g/cm^3	1.228
μ/mm^{-1}	0.073
F(000)	348.0
Crystal size/mm^3	0.6 × 0.1 × 0.1
Radiation	MoKα (λ = 0.71073)
2Θ range for data collection/°	4.734 to 49.996
Index ranges	-10 ≤ h ≤ 10, -11 ≤ k ≤ 11, -14 ≤ l ≤ 14
Reflections collected	10376
Independent reflections	3062 [R_{int} = 0.1258, R_{sigma} = 0.1255]
Data/restraints/parameters	3062/0/229
Goodness-of-fit on F^2	1.041
Final R indexes [I>=2σ (I)]	R_1 = 0.0488, wR_2 = 0.1044
Final R indexes [all data]	R_1 = 0.1142, wR_2 = 0.1127
Largest diff. peak/hole / e Å^{-3}	0.28/−0.23

Table S7 Fractional Atomic Coordinates (×10^4) and Equivalent Isotropic Displacement Parameters (Å^2×10^3) for 2a. U_{eq} is defined as 1/3 of of the trace of the orthogonalised U_{IJ} tensor.

Atom	*x*	*y*	*z*	U(eq)
O^1	9102.4(15)	2760.3(14)	1688.8(9)	25.0(3)
C^1	7326(2)	3612(2)	7047.4(13)	24.8(4)
C^2	7798(2)	2700.9(19)	6464.2(13)	20.8(4)
C^3	6913(2)	2059.5(18)	5225.7(13)	16.6(3)
C^4	7336(2)	977.3(18)	4605.8(13)	18.4(3)
C^5	8344(2)	1614.3(18)	3755.3(13)	17.3(4)
C^6	7507.0(19)	2518.7(17)	3019.9(12)	15.7(4)
C^7	8206(2)	3162.7(18)	2055.1(12)	17.9(4)
C^8	7783(2)	4363.0(18)	1557.6(12)	17.4(4)
C^9	7064(2)	5103.2(18)	2071.7(13)	19.8(4)
C^{10}	6780(2)	6250.2(19)	1594.3(14)	23.5(4)
C^{11}	7195(2)	6664(2)	604.6(15)	26.8(4)
C^{12}	5976(2)	3897.8(19)	6403.6(13)	23.6(4)
C^{13}	10327(2)	2746.2(19)	4492.8(13)	22.9(4)
C^{14}	8185(2)	4787(2)	553.0(13)	23.3(4)
C^{15}	7896(2)	5934(2)	77.7(14)	27.0(4)
C^{16}	5473(2)	1627.7(17)	2510.4(13)	16.2(3)
C^{17}	4490(2)	815.9(18)	1308.3(13)	20.2(4)
C^{18}	2633(2)	59.5(19)	839.3(13)	24.2(4)
C^{19}	1722(2)	154.9(19)	1561.3(14)	23.8(4)
C^{20}	2686(2)	943.9(19)	2764.0(13)	20.7(4)
C^{21}	4540(2)	1642.0(17)	3260.2(13)	16.3(3)
C^{22}	5545(2)	2359.7(18)	4570.0(13)	16.6(3)
C^{23}	5086(2)	3271.5(19)	5169.9(13)	19.5(4)
C^{24}	8119(2)	204.3(19)	2968.4(14)	23.4(4)

Table S8 Anisotropic Displacement Parameters (Å^2×10^3) for 2a. The Anisotropic displacement factor exponent takes the form: $-2\pi^2[h^2a^{*2}U_{11}+2hka^*b^*U_{12}+…]$.

Atom	U_{11}	U_{22}	U_{33}	U_{23}	U_{13}	U_{12}
O^1	31.2(7)	28.6(7)	24.3(6)	5.7(5)	17.6(5)	17.5(6)
C^1	26.3(10)	26.0(10)	18.0(8)	2.7(6)	11.3(7)	7.9(8)
C^2	20.8(9)	21.4(9)	19.4(8)	8.1(6)	9.7(7)	8.0(7)
C^3	18.0(9)	14.3(8)	18.2(7)	6.5(6)	11.1(6)	5.3(7)
C^4	21.2(9)	16.6(8)	20.3(7)	6.6(6)	8.8(6)	10.6(7)

C^5	20.1(9)	17.2(9)	18.6(7)	5.1(6)	10.1(6)	10.4(7)
C^6	18.5(8)	13.9(8)	15.1(7)	1.5(6)	7.9(6)	7.6(7)
C^7	18.6(8)	18.3(9)	15.5(7)	0.7(6)	8.2(6)	7.1(7)
C^8	14.7(8)	16.0(9)	16.7(7)	2.8(6)	6.6(6)	3.3(7)
C^9	21.4(9)	17.1(9)	17.6(7)	2.1(6)	8.0(6)	6.6(7)
C^{10}	23.7(9)	16.1(9)	28.2(8)	4.9(6)	10.2(7)	7.5(7)
C^{11}	24.0(10)	18.1(9)	28.9(8)	8.5(7)	5.2(7)	5.3(8)
C^{12}	28.4(10)	21.2(9)	23.3(8)	3.3(6)	16.1(7)	10.0(8)
C^{13}	19.3(9)	26.6(10)	24.3(8)	7.3(7)	8.9(7)	11.8(8)
C^{14}	22.5(9)	25.9(10)	18.6(8)	3.8(6)	10.4(7)	8.0(8)
C^{15}	24.1(9)	29.2(10)	22.1(8)	11.2(7)	10.8(7)	6.6(8)
C^{16}	20.0(9)	13.1(8)	17.1(7)	4.9(6)	7.6(6)	8.9(7)
C^{17}	25.6(9)	17.4(8)	17.4(7)	1.8(6)	8.2(7)	10.6(7)
C^{18}	27.4(10)	19.0(9)	18.6(8)	1.3(6)	2.0(7)	9.8(8)
C^{19}	18.7(9)	20.2(9)	26.2(8)	7.2(7)	5.1(7)	6.3(7)
C^{20}	20.3(9)	20.8(9)	22.6(8)	8.0(6)	9.7(7)	9.8(7)
C^{21}	20.1(8)	12.2(8)	18.1(8)	5.7(6)	8.6(7)	8.1(7)
C^{22}	17.6(8)	15.3(8)	18.1(8)	5.9(6)	11.1(6)	6.0(7)
C^{23}	20.5(8)	19.7(9)	21.8(8)	6.9(6)	13.0(7)	9.2(7)
C^{24}	28.8(10)	20.0(9)	27.7(8)	5.0(6)	12.6(7)	15.6(8)

Table S9 Bond Lengths for 2a.

Atom	Atom	Length/Å	Atom	Atom	Length/Å
O^1	C^7	1.2214(16)	C^8	C^{14}	1.395(2)
C^1	C^2	1.3920(19)	C^9	C^{10}	1.386(2)
C^1	C^{12}	1.380(2)	C^{10}	C^{11}	1.381(2)
C^2	C^3	1.3967(19)	C^{11}	C^{15}	1.388(2)
C^3	C^4	1.5154(17)	C^{12}	C^{23}	1.390(2)
C^3	C^{22}	1.406(2)	C^{14}	C^{15}	1.387(2)
C^4	C^5	1.5470(19)	C^{16}	C^{17}	1.3960(18)
C^5	C^6	1.558(2)	C^{16}	C^{21}	1.4110(17)
C^5	C^{13}	1.530(2)	C^{17}	C^{18}	1.385(2)
C^5	C^{24}	1.5357(17)	C^{18}	C^{19}	1.389(2)
C^6	C^7	1.5206(19)	C^{19}	C^{20}	1.390(2)
C^6	C^{16}	1.517(2)	C^{20}	C^{21}	1.387(2)
C^7	C^8	1.501(2)	C^{21}	C^{22}	1.4860(18)
C^8	C^9	1.3989(18)	C^{22}	C^{23}	1.3961(18)

Table S10 Bond Angles for 2a.

Atom	Atom	Atom	Angle/˚	Atom	Atom	Atom	Angle/˚
C^{12}	C^1	C^2	120.18(14)	C^{10}	C^9	C^8	120.19(15)
C^1	C^2	C^3	120.78(15)	C^{11}	C^{10}	C^9	120.28(14)
C^2	C^3	C^4	120.78(14)	C^{10}	C^{11}	C^{15}	120.30(17)
C^2	C^3	C^{22}	118.97(12)	C^1	C^{12}	C^{23}	119.74(12)
C^{22}	C^3	C^4	120.15(13)	C^{15}	C^{14}	C^8	120.70(14)
C^3	C^4	C^5	116.69(13)	C^{14}	C^{15}	C^{11}	119.63(16)
C^4	C^5	C^6	107.33(11)	C^{17}	C^{16}	C^6	121.82(12)
C^{13}	C^5	C^4	110.05(12)	C^{17}	C^{16}	C^{21}	118.76(13)
C^{13}	C^5	C^6	108.50(13)	C^{21}	C^{16}	C^6	119.41(12)
C^{13}	C^5	C^{24}	110.82(12)	C^{18}	C^{17}	C^{16}	121.36(13)
C^{24}	C^5	C^4	106.64(12)	C^{17}	C^{18}	C^{19}	119.68(13)
C^{24}	C^5	C^6	113.41(12)	C^{18}	C^{19}	C^{20}	119.46(14)
C^7	C^6	C^5	116.81(11)	C^{21}	C^{20}	C^{19}	121.39(13)
C^{16}	C^6	C^5	112.68(12)	C^{16}	C^{21}	C^{22}	119.88(13)
C^{16}	C^6	C^7	110.47(11)	C^{20}	C^{21}	C^{16}	119.16(13)
O^1	C^7	C^6	122.91(15)	C^{20}	C^{21}	C^{22}	120.94(12)
O^1	C^7	C^8	119.50(14)	C^3	C^{22}	C^{21}	119.87(11)
C^8	C^7	C^6	117.59(11)	C^{23}	C^{22}	C^3	119.49(14)
C^9	C^8	C^7	123.35(14)	C^{23}	C^{22}	C^{21}	120.56(14)
C^{14}	C^8	C^7	117.74(13)	C^{12}	C^{23}	C^{22}	120.83(15)
C^{14}	C^8	C^9	118.89(15)				

Table S11 Torsion Angles for 2a.

A	B	C	D	Angle/˚	A	B	C	D	Angle/˚
O^{1}	C^{7}	C^{8}	C^{9}	-167.67(14)	C^{7}	C^{8}	C^{14}	C^{15}	-177.35(15)
O^{1}	C^{7}	C^{8}	C^{14}	10.3(2)	C^{8}	C^{9}	C^{10}	C^{11}	0.4(2)
C^{1}	C^{2}	C^{3}	C^{4}	-175.72(16)	C^{8}	C^{14}	C^{15}	C^{11}	-0.2(3)
C^{1}	C^{2}	C^{3}	C^{22}	0.6(2)	C^{9}	C^{8}	C^{14}	C^{15}	0.7(2)
C^{1}	C^{12}	C^{23}	C^{22}	-0.3(3)	C^{9}	C^{10}	C^{11}	C^{15}	0.1(3)
C^{2}	C^{1}	C^{12}	C^{23}	0.1(3)	C^{10}	C^{11}	C^{15}	C^{14}	-0.2(3)
C^{2}	C^{3}	C^{4}	C^{5}	-109.75(17)	C^{12}	C^{1}	C^{2}	C^{3}	-0.2(3)
C^{2}	C^{3}	C^{22}	C^{21}	-177.76(14)	C^{13}	C^{5}	C^{6}	C^{7}	65.13(14)
C^{2}	C^{3}	C^{22}	C^{23}	-0.8(2)	C^{13}	C^{5}	C^{6}	C^{16}	-165.38(10)
C^{3}	C^{4}	C^{5}	C^{6}	-41.62(17)	C^{14}	C^{8}	C^{9}	C^{10}	-0.7(2)
C^{3}	C^{4}	C^{5}	C^{13}	76.27(16)	C^{16}	C^{6}	C^{7}	O^{1}	-116.31(15)
C^{3}	C^{4}	C^{5}	C^{24}	-163.46(13)	C^{16}	C^{6}	C^{7}	C^{8}	64.67(16)
C^{3}	C^{22}	C^{23}	C^{12}	0.7(3)	C^{16}	C^{17}	C^{18}	C^{19}	2.5(2)
C^{4}	C^{3}	C^{22}	C^{21}	-1.4(2)	C^{16}	C^{21}	C^{22}	C^{3}	-44.3(2)
C^{4}	C^{3}	C^{22}	C^{23}	175.54(15)	C^{16}	C^{21}	C^{22}	C^{23}	138.84(16)
C^{4}	C^{5}	C^{6}	C^{7}	-175.97(11)	C^{17}	C^{16}	C^{21}	C^{20}	-4.4(2)
C^{4}	C^{5}	C^{6}	C^{16}	-46.48(14)	C^{17}	C^{16}	C^{21}	C^{22}	173.84(14)
C^{5}	C^{6}	C^{7}	O^{1}	14.2(2)	C^{17}	C^{18}	C^{19}	C^{20}	-3.2(2)
C^{5}	C^{6}	C^{7}	C^{8}	-164.80(12)	C^{18}	C^{19}	C^{20}	C^{21}	0.1(2)
C^{5}	C^{6}	C^{16}	C^{17}	-100.65(15)	C^{19}	C^{20}	C^{21}	C^{16}	3.7(2)
C^{5}	C^{6}	C^{16}	C^{21}	80.74(15)	C^{19}	C^{20}	C^{21}	C^{22}	-174.49(15)
C^{6}	C^{7}	C^{8}	C^{9}	11.4(2)	C^{20}	C^{21}	C^{22}	C^{3}	133.96(15)
C^{6}	C^{7}	C^{8}	C^{14}	-170.66(13)	C^{20}	C^{21}	C^{22}	C^{23}	-42.9(2)
C^{6}	C^{16}	C^{17}	C^{18}	-177.29(14)	C^{21}	C^{16}	C^{17}	C^{18}	1.3(2)
C^{6}	C^{16}	C^{21}	C^{20}	174.25(14)	C^{21}	C^{22}	C^{23}	C^{12}	177.57(14)
C^{6}	C^{16}	C^{21}	C^{22}	-7.5(2)	C^{22}	C^{3}	C^{4}	C^{5}	73.96(19)
C^{7}	C^{6}	C^{16}	C^{17}	32.03(17)	C^{24}	C^{5}	C^{6}	C^{7}	-58.46(18)
C^{7}	C^{6}	C^{16}	C^{21}	-146.59(13)	C^{24}	C^{5}	C^{6}	C^{16}	71.04(15)
C^{7}	C^{8}	C^{9}	C^{10}	177.18(15)					

Table S12 Hydrogen Atom Coordinates (Å×10^{4}) and Isotropic Displacement Parameters (Å^{2}×10^{3}) for 2a.

Atom	*x*	*y*	*z*	U(eq)
H^{1}	7936.32	4037.8	7890.78	30
H^{2}	8731.81	2513.3	6913.82	25
H^{4A}	6192.96	20.69	4133.81	22
H^{4B}	8061.54	675.18	5235.24	22
H^{6}	7845.03	3450.28	3620.93	19
H^{9}	6770.36	4819.1	2750.08	24
H^{10}	6298.25	6753.44	1949.57	28
H^{11}	6998.53	7452.6	282.83	32
H^{12}	5655.63	4520.31	6802.12	28
H^{13A}	10876.5	2191.18	4955.01	34
H^{13B}	10435.24	3581.25	5047.04	34
H^{13C}	10939.92	3191.3	3948.82	34
H^{14}	8660.61	4283.93	190.61	28
H^{15}	8177.54	6218.88	-604.6	32
H^{17}	5106.35	781.55	801.53	24
H^{18}	1985.09	-521.66	27.91	29
H^{19}	451.87	-315.12	1235.86	29
H^{20}	2059.92	1006.08	3256.13	25
H^{23}	4152.89	3466.3	4729.41	23
H^{24A}	8875.77	562.42	2493.39	35
H^{24B}	6861.22	-432.22	2424.77	35
H^{24C}	8475.69	-417.25	3484.78	35

Crystallographic data of 3q are presented in Tables S13-20. A single crystal of **3q** was coated in high-vacuum grease and mounted on a glass fiber. X-ray measurements were made using a Bruker D8 VENTURE PhotonIII area-detector diffractometer with Mo-Kα radiation (α = 1.54 Å). Absorption corrections were applied, based on multiple and symmetry-equivalent measurements. The structure was solved by ShelXT structure solution program using Intrinsic Phasing and refined with the XL refinement package using Least Squares minimization.[7] All non-hydrogen atoms were assigned anisotropic displacement parameters and refined without positional constraints and all other hydrogen atoms were constrained to ideal geometries and refined with fixed isotropic displacement parameters. Refinement proceeded smoothly to give the residuals shown in Table S14.

A colorless prism-like specimen of $C_{19}H_{19}ClO$, approximate dimensions 0.020 mm x 0.040 mm x 0.100 mm, was used for the X-ray crystallographic analysis. The X-ray intensity data were measured (λ = 1.54184 Å).

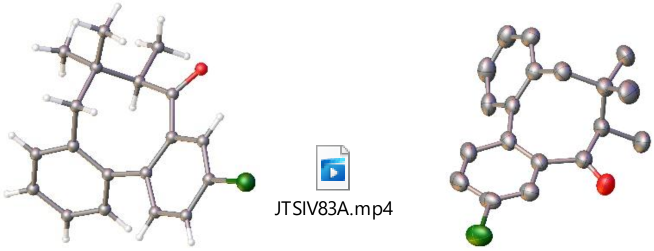

Table S13: Data collection details for 3q.

Axis	dx/mm	2θ/°	ω/°	φ/°	χ/°	Width/°	Frames	Time/s	Wavelength/Å	Voltage/kV	Current/mA	Temperature/K
Omega	39.899	90.94	-12.43	-120.00	61.50	2.00	55	10.00	1.54184	50	1.1	180
Omega	39.899	90.94	-12.43	80.00	61.50	2.00	55	10.00	1.54184	50	1.1	180
Phi	39.899	90.94	89.00	0.00	-44.50	2.00	180	10.00	1.54184	50	1.1	180
Phi	39.899	90.94	354.86	0.00	24.00	2.00	180	10.00	1.54184	50	1.1	180
Omega	39.899	-33.14	-132.80	180.00	44.50	2.00	51	10.00	1.54184	50	1.1	180
Omega	39.899	-45.94	-132.80	0.00	44.50	2.00	44	10.00	1.54184	50	1.1	180
Omega	39.899	-45.94	-132.80	270.00	44.50	2.00	44	10.00	1.54184	50	1.1	180
Omega	39.899	-45.94	-132.80	90.00	44.50	2.00	44	10.00	1.54184	50	1.1	180
Omega	39.899	-48.14	-53.09	51.00	-61.50	2.00	55	10.00	1.54184	50	1.1	180
Omega	39.899	90.94	-12.43	0.00	61.50	2.00	55	10.00	1.54184	50	1.1	180
Omega	39.899	75.94	74.00	90.00	-44.50	2.00	51	10.00	1.54184	50	1.1	180
Omega	39.899	90.94	-12.43	160.00	61.50	2.00	55	10.00	1.54184	50	1.1	180
Omega	39.899	-18.14	-124.02	270.00	44.50	2.00	54	10.00	1.54184	50	1.1	180
Omega	39.899	90.94	-12.43	-40.00	61.50	2.00	55	10.00	1.54184	50	1.1	180
Omega	39.899	75.94	74.00	0.00	-44.50	2.00	51	10.00	1.54184	50	1.1	180
Omega	39.899	-33.14	-132.80	90.00	44.50	2.00	51	10.00	1.54184	50	1.1	180
Phi	39.899	75.94	74.00	0.00	-44.50	2.00	180	10.00	1.54184	50	1.1	180
Omega	39.899	75.94	74.00	180.00	-44.50	2.00	51	10.00	1.54184	50	1.1	180
Omega	39.899	-18.14	-124.02	0.00	44.50	2.00	54	10.00	1.54184	50	1.1	180

A total of 1365 frames were collected. The total exposure time was 3.79 hours. The frames were integrated with the Bruker SAINT software package using a narrow-frame algorithm. The integration of the data using a triclinic unit cell yielded a total of 30198 reflections to a maximum θ angle of 60.03° (0.89 Å resolution). The final cell constants of a = 6.5607(3) Å, b = 14.7169(8) Å, c = 16.5256(8) Å, α = 89.991(3)°, β = 90.025(3)°, γ = 89.998(3)°, volume = 1595.59(14) Å3, are based upon the refinement of the XYZ-centroids of 8918 reflections above 20 σ(I) with 8.044° < 2θ < 116.1°. Data were corrected for absorption effects using the Multi-Scan method (SADABS). The ratio of minimum to maximum apparent transmission was 0.642.

Table S14 Crystal data and structure refinement for **3q**.

Empirical formula	$C_{19}H_{19}ClO$
Formula weight	298.79
Temperature/K	180.0
Crystal system	orthorhombic
Space group	Pba2
a/Å	14.7165(8)
b/Å	16.5287(8)
c/Å	6.5604(4)
α/°	90
β/°	90
γ/°	90
Volume/Å^3	1595.78(15)
Z	4
ρ_{calc}g/cm^3	1.244
μ/mm^{-1}	2.072
F(000)	632.0
Crystal size/mm^3	0.1 × 0.04 × 0.02
Radiation	CuKα (λ = 1.54178)
2Θ range for data collection/°	8.044 to 119.756
Index ranges	-16 ≤ h ≤ 16, -18 ≤ k ≤ 18, -7 ≤ l ≤ 7
Reflections collected	18217
Independent reflections	2338 [R_{int} = 0.1066, R_{sigma} = 0.0560]
Data/restraints/parameters	2338/1/194
Goodness-of-fit on F^2	1.049
Final R indexes [I>=2σ (I)]	R_1 = 0.0518, wR_2 = 0.1259
Final R indexes [all data]	R_1 = 0.0835, wR_2 = 0.1563
Largest diff. peak/hole / e Å^{-3}	0.16/-0.21
Flack parameter	0.04(5)

Table S15 Fractional Atomic Coordinates (×10^4) and Equivalent Isotropic Displacement Parameters (Å^2×10^3) for **3q**. U_{eq} is defined as 1/3 of of the trace of the orthogonalised U_{IJ} tensor.

Atom	*x*	*y*	*z*	U(eq)
Cl1	2040.6(13)	4683.6(14)	9882(4)	115.3(9)
O1	2813(3)	1939(3)	6831(10)	97.9(18)
C6	5391(4)	3102(3)	3868(10)	61.0(15)
C15	3907(4)	3767(3)	4904(10)	60.1(15)
C1	4634(4)	3583(3)	3393(10)	60.4(15)
C14	3411(4)	3218(3)	6097(10)	62.5(16)
C16	3735(4)	4602(3)	5199(12)	72.6(17)
C5	6112(4)	3074(4)	2499(11)	75.0(19)
C11	4014(4)	1852(3)	4462(10)	67.4(18)
C13	3382(4)	2309(3)	5857(11)	67.1(17)
C2	4621(5)	3987(3)	1517(11)	74.5(18)
C7	5416(4)	2597(3)	5758(10)	65.9(16)
C19	2831(4)	3521(4)	7615(11)	71.9(18)
C8	4968(4)	1755(3)	5454(11)	69.3(17)
C18	2723(4)	4335(4)	7907(12)	80(2)
C3	5343(6)	3924(4)	168(13)	85(2)
C4	6087(5)	3486(4)	648(12)	84(2)
C17	3158(5)	4885(4)	6673(13)	80(2)
C12	3573(5)	1043(4)	3836(14)	91(2)
C9	5575(5)	1239(4)	4074(14)	94(2)
C10	4904(6)	1355(5)	7558(14)	103(3)

Table S16 Anisotropic Displacement Parameters (Å^2×10^3) for **3q**. The Anisotropic displacement factor exponent takes the form: $-2\pi^2[h^2a^{*2}U_{11}+2hka^*b^*U_{12}+\ldots]$.

Atom	U_{11}	U_{22}	U_{33}	U_{23}	U_{13}	U_{12}
Cl1	95.3(13)	121.5(16)	129.1(19)	-59.2(15)	18.7(13)	6.5(11)
O1	93(3)	67(3)	133(5)	-2(3)	31(3)	-9(2)
C6	63(4)	55(3)	64(4)	-12(3)	-4(3)	-3(3)
C15	68(3)	50(3)	63(4)	-9(3)	-14(3)	3(3)
C1	68(4)	44(3)	69(4)	-6(3)	-10(3)	-4(2)
C14	60(3)	56(3)	72(4)	-5(3)	-8(3)	3(3)
C16	77(4)	53(3)	88(5)	-7(4)	-14(4)	0(3)
C5	69(4)	69(4)	86(5)	-9(4)	1(4)	-8(3)
C11	69(3)	48(3)	86(5)	-11(3)	-2(3)	3(3)
C13	60(3)	58(3)	83(5)	-2(3)	-5(3)	-1(3)
C2	100(5)	48(3)	76(5)	4(3)	-6(4)	-4(3)
C7	72(4)	62(4)	64(4)	-6(3)	-7(3)	3(3)
C19	66(4)	73(4)	76(5)	-14(4)	-3(3)	1(3)
C8	71(4)	57(3)	80(5)	0(3)	-4(3)	7(3)
C18	60(4)	82(5)	98(6)	-38(5)	-4(4)	10(3)
C3	123(6)	65(4)	68(5)	5(4)	7(5)	-6(4)
C4	101(5)	69(4)	82(5)	-9(4)	19(4)	-10(4)
C17	76(4)	63(4)	103(6)	-29(4)	-14(4)	10(4)
C12	89(5)	56(3)	126(7)	-23(4)	-2(5)	-8(3)
C9	88(5)	61(4)	132(7)	-6(4)	6(5)	15(3)
C10	109(6)	96(6)	105(7)	37(5)	-16(5)	2(5)

Table S17 Bond Lengths for **3q.**

Atom	Atom	Length/Å	Atom	Atom	Length/Å
Cl1	C18	1.738(7)	C5	C4	1.393(10)
O1	C13	1.218(7)	C11	C13	1.508(8)
C6	C1	1.404(8)	C11	C8	1.555(8)
C6	C5	1.390(9)	C11	C12	1.542(8)
C6	C7	1.495(9)	C2	C3	1.387(10)
C15	C1	1.490(9)	C7	C8	1.554(8)
C15	C14	1.403(8)	C19	C18	1.367(9)
C15	C16	1.416(7)	C8	C9	1.531(9)
C1	C2	1.400(9)	C8	C10	1.533(11)
C14	C13	1.511(8)	C18	C17	1.376(10)
C14	C19	1.403(9)	C3	C4	1.350(10)
C16	C17	1.369(10)			

Table S18 Bond Angles for **3q.**

Atom	Atom	Atom	Angle/˚	Atom	Atom	Atom	Angle/˚
C1	C6	C7	121.3(6)	O1	C13	C11	119.4(5)
C5	C6	C1	118.7(6)	C11	C13	C14	122.9(6)
C5	C6	C7	119.9(5)	C3	C2	C1	120.9(7)
C14	C15	C1	127.9(5)	C6	C7	C8	112.5(5)
C14	C15	C16	117.4(6)	C18	C19	C14	121.4(7)
C16	C15	C1	114.7(5)	C7	C8	C11	110.1(4)
C6	C1	C15	122.5(6)	C9	C8	C11	109.7(6)
C2	C1	C6	118.5(6)	C9	C8	C7	109.1(5)
C2	C1	C15	118.5(5)	C9	C8	C10	109.1(6)
C15	C14	C13	126.8(6)	C10	C8	C11	111.5(6)
C15	C14	C19	118.8(5)	C10	C8	C7	107.3(6)
C19	C14	C13	114.3(6)	C19	C18	Cl1	119.9(6)
C17	C16	C15	122.7(7)	C19	C18	C17	120.9(6)
C6	C5	C4	121.8(6)	C17	C18	Cl1	119.2(5)

C13	C11	C8	110.8(5)	C4	C3	C2	120.8(7)
C13	C11	C12	109.7(5)	C3	C4	C5	119.1(7)
C12	C11	C8	113.6(5)	C16	C17	C18	118.6(6)
O1	C13	C14	117.7(6)				

Table S19 Torsion Angles for **3q.**

A	B	C	D	Angle/˚	A	B	C	D	Angle/˚
Cl1	C18	C17	C16	176.2(5)	C16	C15	C14	C13	171.0(6)
C6	C1	C2	C3	-0.9(9)	C16	C15	C14	C19	-5.0(8)
C6	C5	C4	C3	0.0(10)	C5	C6	C1	C15	-169.0(5)
C6	C7	C8	C11	-49.9(7)	C5	C6	C1	C2	2.8(8)
C6	C7	C8	C9	70.5(7)	C5	C6	C7	C8	-91.5(7)
C6	C7	C8	C10	-171.4(6)	C13	C14	C19	C18	-174.5(6)
C15	C1	C2	C3	171.2(5)	C13	C11	C8	C7	-63.9(7)
C15	C14	C13	O1	-169.4(6)	C13	C11	C8	C9	176.1(5)
C15	C14	C13	C11	9.9(10)	C13	C11	C8	C10	55.1(7)
C15	C14	C19	C18	2.0(9)	C2	C3	C4	C5	2.0(10)
C15	C16	C17	C18	-0.5(10)	C7	C6	C1	C15	13.6(8)
C1	C6	C5	C4	-2.4(9)	C7	C6	C1	C2	-174.6(5)
C1	C6	C7	C8	85.9(6)	C7	C6	C5	C4	175.1(6)
C1	C15	C14	C13	-11.8(10)	C19	C14	C13	O1	6.8(9)
C1	C15	C14	C19	172.2(6)	C19	C14	C13	C11	-173.9(5)
C1	C15	C16	C17	-173.2(6)	C19	C18	C17	C16	-2.8(10)
C1	C2	C3	C4	-1.5(10)	C8	C11	C13	O1	-102.3(7)
C14	C15	C1	C6	-52.3(8)	C8	C11	C13	C14	78.3(7)
C14	C15	C1	C2	135.9(6)	C12	C11	C13	O1	23.9(9)
C14	C15	C16	C17	4.4(9)	C12	C11	C13	C14	-155.4(6)
C14	C19	C18	Cl1	-176.9(5)	C12	C11	C8	C7	172.1(6)
C14	C19	C18	C17	2.1(10)	C12	C11	C8	C9	52.1(8)
C16	C15	C1	C6	125.0(6)	C12	C11	C8	C10	-68.9(8)
C16	C15	C1	C2	-46.8(7)					

Table S20 Hydrogen Atom Coordinates ($Å\times10^4$) and Isotropic Displacement Parameters ($Å^2\times10^3$) for **3q**.

Atom	*x*	*y*	*z*	U(eq)
H16	4032.62	4981.12	4338.78	87
H5	6635.63	2765.65	2836.39	90
H11	4091.94	2182.85	3197.33	81
H2	4109.59	4308.74	1163.71	89
H7A	5096.09	2887.93	6865.51	79
H7B	6055.77	2522.07	6183.29	79
H19	2507.26	3153.4	8458.03	86
H3	5313.58	4192.61	-1110.68	102
H4	6587.09	3458.72	-265.14	101
H17	3060.49	5449.08	6839.72	97
H12A	3566.41	675.42	5007.1	136
H12B	3924.19	799.3	2724.77	136
H12C	2949.1	1140.93	3376.91	136
H9A	5358.34	678.56	4082.31	141
H9B	6201.62	1257.48	4577.08	141
H9C	5553.33	1451.76	2680.73	141
H10A	4551.28	1701.43	8475.26	155
H10B	5516.88	1279.96	8113.06	155
H10C	4604.73	827.92	7429.89	155

Crystallographic data of 4ae are presented in Tables S21-28. A single crystal of **4ae** was coated in high-vacuum grease and mounted on a glass fiber. X-ray measurements were made using a Bruker D8 VENTURE PhotonIII area-detector diffractometer with Cu-Kα radiation (α = 1.54 Å). Absorption corrections were applied, based on multiple and symmetry-equivalent measurements. The structure was solved by ShelXT structure solution program using Intrinsic Phasing and refined with the XL refinement package using Least Squares minimization.[7] All non-hydrogen atoms were assigned anisotropic displacement parameters and refined without positional constraints and all other hydrogen atoms were constrained to ideal geometries and refined with fixed isotropic displacement parameters. Refinement proceeded smoothly to give the residuals shown in Table S22.

A colorless prism-like specimen of $C_{33}H_{32}O_3$, approximate dimensions 0.040 mm x 0.050 mm x 0.300 mm, was used for the X-ray crystallographic analysis. The X-ray intensity data were measured (λ = 1.54184 Å).

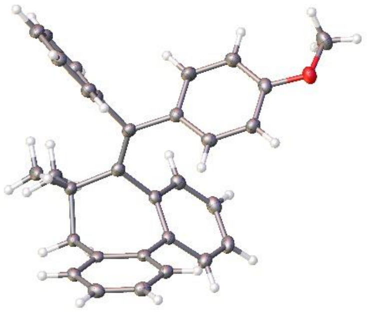

JTSII137A.mp4

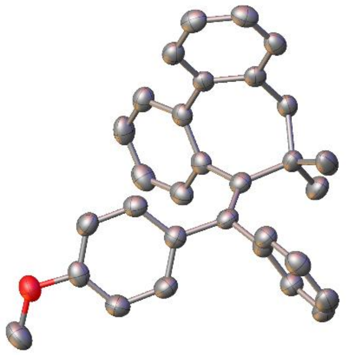

Table S21: Data collection details for 4ae.

Axis	dx/mm	2θ/°	ω/°	φ/°	χ/°	Width/°	Frames	Time/s	Wavelength/Å	Voltage/kV	Current/mA	Temperature/K
Omega	39.880	108.54	107.22	160.00	-44.50	1.60	67	10.00	1.54184	50	1.1	100
Omega	39.880	108.54	107.22	80.00	-44.50	1.60	67	10.00	1.54184	50	1.1	100
Phi	39.880	93.54	91.61	0.00	-44.50	1.60	225	10.00	1.54184	50	1.1	100
Omega	39.880	108.54	-6.42	80.00	61.50	1.60	76	10.00	1.54184	50	1.1	100
Omega	39.880	-48.14	-53.49	102.00	-61.50	1.60	69	10.00	1.54184	50	1.1	100
Omega	39.880	-48.14	-153.22	51.00	44.50	1.60	67	10.00	1.54184	50	1.1	100
Omega	39.880	108.54	-6.42	-120.00	61.50	1.60	76	10.00	1.54184	50	1.1	100
Omega	39.880	108.54	107.22	120.00	-44.50	1.60	67	10.00	1.54184	50	1.1	100
Omega	39.880	-18.14	-123.22	0.00	44.50	1.60	67	10.00	1.54184	50	1.1	100
Omega	39.880	108.54	107.22	40.00	-44.50	1.60	67	10.00	1.54184	50	1.1	100
Omega	39.880	-63.54	-168.62	90.00	44.50	1.60	67	10.00	1.54184	50	1.1	100
Phi	39.880	108.54	108.75	0.00	-24.00	1.60	225	10.00	1.54184	50	1.1	100
Omega	39.880	108.54	-6.42	0.00	61.50	1.60	76	10.00	1.54184	50	1.1	100
Omega	39.880	78.54	77.22	270.00	-44.50	1.60	67	10.00	1.54184	50	1.1	100
Phi	39.880	78.54	76.61	0.00	-44.50	1.60	225	10.00	1.54184	50	1.1	100

A total of 1508 frames were collected. The total exposure time was 4.19 h. The frames were integrated with the Bruker SAINT software package using a narrow-frame algorithm. The integration of the data using a monoclinic unit cell yielded a total of 23289 reflections to a maximum θ angle of 72.59° (0.81 Å resolution). The final cell constants of a = 16.6086(3) Å, b = 6.84724(10) Å, c =20.6686(4) Å, β = 103.5624(8)°, volume = 2284.94(8) $Å^3$, are based upon the refinement of the XYZ-centroids of 9988 reflections above 20 σ(I) with 8.802° < 2θ < 144.1°. Data were corrected for absorption effects using the Multi-Scan method (SADABS). The ratio of minimum to maximum apparent transmission was 0.832.

Table S22 Crystal data and structure refinement for **JTSII137A**.

Empirical formula	$C_{31}H_{28}O$
Formula weight	416.53
Temperature/K	100.0
Crystal system	monoclinic
Space group	$P2_1/c$
a/Å	16.6086(3)
b/Å	6.84720(10)
c/Å	20.6686(4)
α/°	90
β/°	103.5620(10)
γ/°	90
Volume/Å^3	2284.94(7)
Z	4
ρ_{calc}g/cm^3	1.211
μ/mm^{-1}	0.544
F(000)	888.0
Crystal size/mm^3	0.3 × 0.05 × 0.04
Radiation	CuKα (λ = 1.54178)
2Θ range for data collection/°	5.474 to 144.658
Index ranges	-20 ≤ h ≤ 20, -7 ≤ k ≤ 8, -22 ≤ l ≤ 25
Reflections collected	21720
Independent reflections	4480 [R_{int} = 0.0514, R_{sigma} = 0.0380]
Data/restraints/parameters	4480/0/292
Goodness-of-fit on F^2	1.031
Final R indexes [I>=2σ (I)]	R_1 = 0.0476, wR_2 = 0.1080
Final R indexes [all data]	R_1 = 0.0604, wR_2 = 0.1211
Largest diff. peak/hole / e Å^{-3}	0.27/-0.27

Table S23 Fractional Atomic Coordinates (×10^4) and Equivalent Isotropic Displacement Parameters (Å^2×10^3) for **4ae**. U_{eq} is defined as 1/3 of of the trace of the orthogonalised U_{IJ} tensor.

Atom	*x*	*y*	*z*	U(eq)
O^1	4004.1(7)	3850.1(18)	6492.6(6)	27.8(3)
C^{21}	7182.1(9)	2230(2)	5221.2(7)	18.9(3)
C^{16}	7210.9(9)	1228(2)	5820.1(8)	19.8(3)
C^{22}	7779.0(9)	3825(2)	5199.9(7)	18.4(3)
C^{27}	8624.6(9)	3520(2)	5488.3(7)	18.6(3)
C^8	7470.4(9)	2638(2)	6938.9(7)	19.6(3)
C^{20}	6587.5(10)	1705(2)	4647.9(8)	22.8(3)
C^{28}	8896.2(9)	1591(2)	5812.5(7)	19.7(3)
C^{26}	9188.2(10)	4999(2)	5451.7(7)	21.1(3)
C^{15}	7798.0(9)	1841(2)	6462.9(7)	19.3(3)
C^2	4846.2(9)	3562(2)	6639.9(8)	22.3(3)
C^{17}	6658.8(10)	-312(2)	5822.6(8)	22.5(3)
C^{23}	7519.8(10)	5593(2)	4883.9(7)	21.6(3)
C^5	6563.0(9)	3054(2)	6835.8(7)	20.3(3)
C^{10}	8151.9(10)	5016(2)	7825.3(8)	22.9(3)
C^{24}	8089.7(10)	7053(2)	4853.9(8)	23.7(3)
C^6	6119.5(10)	4196(2)	6311.1(8)	21.7(3)
C^{14}	8156.5(10)	1616(3)	8103.2(8)	23.0(3)
C^9	7946.5(9)	3095(2)	7632.2(7)	20.8(3)
C^3	5275.0(10)	2484(3)	7176.6(8)	25.1(4)
C^{18}	6079.7(10)	-831(2)	5249.7(9)	25.6(4)
C^7	5271.6(10)	4463(2)	6215.3(8)	22.9(3)
C^{29}	8718.4(9)	1353(2)	6514.2(7)	20.8(3)
C^{13}	8578.4(10)	2039(3)	8752.5(8)	25.6(4)
C^{25}	8925.1(10)	6751(2)	5136.0(8)	24.0(3)

C^{4}	6126.9(10)	2266(2)	7275.0(8)	24.3(4)
C^{19}	6037.7(10)	188(3)	4663.6(8)	25.8(4)
C^{31}	8865.0(11)	-807(3)	6714.8(8)	27.4(4)
C^{11}	8584.7(10)	5437(3)	8468.5(8)	26.0(4)
C^{30}	9345.1(10)	2638(3)	6992.3(8)	28.2(4)
C^{12}	8799.6(10)	3946(3)	8933.2(8)	26.7(4)
C^{1}	3533.0(11)	2593(3)	6817.1(10)	33.1(4)

Table S24 Anisotropic Displacement Parameters (Å^2×10^3) for **4ae**. The Anisotropic displacement factor exponent takes the form: $-2\pi^2[h^2a^{*2}U_{11}+2hka^*b^*U_{12}+\ldots]$.

**Atom**	**U_{11}**	**U_{22}**	**U_{33}**	**U_{23}**	**U_{13}**	**U_{12}**
O^{1}	18.9(6)	32.9(7)	31.3(6)	2.9(5)	5.6(5)	0.9(5)
C^{21}	17.9(7)	19.5(8)	19.6(7)	-2.7(6)	5.0(6)	2.9(6)
C^{16}	18.2(7)	19.5(8)	21.9(8)	-1.4(6)	5.2(6)	2.6(6)
C^{22}	19.5(7)	22.4(8)	13.7(7)	-3.4(6)	4.5(6)	0.2(6)
C^{27}	20.4(7)	22.2(8)	13.8(7)	-3.7(6)	5.1(6)	1.2(6)
C^{8}	21.6(8)	19.3(8)	17.3(7)	3.2(6)	3.2(6)	-0.4(6)
C^{20}	22.1(8)	24.9(9)	20.0(8)	-2.1(6)	1.8(6)	2.0(6)
C^{28}	17.5(7)	23.7(8)	17.7(7)	-2.1(6)	3.6(6)	1.3(6)
C^{26}	19.0(7)	25.4(9)	19.7(7)	-5.0(6)	6.1(6)	-1.1(6)
C^{15}	20.8(7)	17.8(8)	18.8(7)	2.4(6)	3.8(6)	0.2(6)
C^{2}	19.0(7)	22.3(8)	24.9(8)	-4.6(6)	3.9(6)	0.0(6)
C^{17}	22.1(8)	20.7(8)	26.0(8)	0.8(6)	8.0(6)	1.6(6)
C^{23}	22.0(8)	24.9(9)	17.9(7)	-1.2(6)	4.3(6)	2.4(6)
C^{5}	21.0(8)	19.5(8)	20.0(7)	-0.8(6)	3.9(6)	0.3(6)
C^{10}	23.1(8)	23.6(9)	22.1(8)	3.0(6)	5.8(6)	2.0(6)
C^{24}	30.4(9)	19.6(8)	21.8(8)	0.6(6)	7.8(6)	3.2(7)
C^{6}	24.9(8)	19.9(8)	21.3(8)	1.4(6)	7.6(6)	0.7(6)
C^{14}	22.4(8)	24.1(9)	22.5(8)	1.5(6)	5.2(6)	0.3(6)
C^{9}	17.5(7)	27.4(9)	17.8(7)	0.6(6)	5.2(6)	2.6(6)
C^{3}	23.9(8)	28.4(9)	24.4(8)	2.6(7)	8.3(6)	-1.6(7)
C^{18}	20.8(8)	22.1(9)	34.5(9)	-3.9(7)	7.5(7)	-2.9(6)
C^{7}	23.6(8)	23.4(8)	20.6(8)	2.4(6)	3.2(6)	3.8(6)
C^{29}	18.4(7)	27.5(9)	16.1(7)	0.8(6)	2.9(6)	2.7(6)
C^{13}	23.3(8)	35.3(10)	19.0(8)	6.0(7)	6.4(6)	4.8(7)
C^{25}	27.6(8)	22.7(9)	24.1(8)	-3.0(6)	10.6(6)	-5.0(6)
C^{4}	25.9(8)	25.8(9)	21.2(8)	5.3(6)	5.5(6)	2.7(7)
C^{19}	19.2(8)	29.6(9)	25.9(8)	-7.5(7)	-0.1(6)	0.0(7)
C^{31}	26.6(8)	32.3(10)	23.2(8)	7.4(7)	5.5(7)	8.5(7)
C^{11}	25.0(8)	27.8(9)	26.7(8)	-5.5(7)	9.0(7)	-1.7(7)
C^{30}	19.4(8)	44.4(11)	20.0(8)	-4.5(7)	3.4(6)	-1.7(7)
C^{12}	23.7(8)	38.4(10)	17.5(7)	-3.7(7)	3.7(6)	1.4(7)
C^{1}	21.9(8)	37.7(11)	41.6(10)	0.1(8)	11.4(7)	-3.2(7)

Table S25 Bond Lengths for **4ae**.

Atom	Atom	Length/Å	Atom	Atom	Length/Å
O^{1}	C^{2}	1.3740(19)	C^{2}	C^{3}	1.383(2)
O^{1}	C^{1}	1.431(2)	C^{2}	C^{7}	1.393(2)
C^{21}	C^{16}	1.406(2)	C^{17}	C^{18}	1.385(2)
C^{21}	C^{22}	1.483(2)	C^{23}	C^{24}	1.388(2)
C^{21}	C^{20}	1.400(2)	C^{5}	C^{6}	1.398(2)
C^{16}	C^{15}	1.512(2)	C^{5}	C^{4}	1.396(2)
C^{16}	C^{17}	1.398(2)	C^{10}	C^{9}	1.394(2)
C^{22}	C^{27}	1.407(2)	C^{10}	C^{11}	1.385(2)
C^{22}	C^{23}	1.394(2)	C^{24}	C^{25}	1.389(2)
C^{27}	C^{28}	1.502(2)	C^{6}	C^{7}	1.388(2)
C^{27}	C^{26}	1.393(2)	C^{14}	C^{9}	1.391(2)
C^{8}	C^{15}	1.347(2)	C^{14}	C^{13}	1.391(2)
C^{8}	C^{5}	1.499(2)	C^{3}	C^{4}	1.389(2)
C^{8}	C^{9}	1.498(2)	C^{18}	C^{19}	1.385(2)
C^{20}	C^{19}	1.388(2)	C^{29}	C^{31}	1.540(2)
C^{28}	C^{29}	1.555(2)	C^{29}	C^{30}	1.534(2)
C^{26}	C^{25}	1.387(2)	C^{13}	C^{12}	1.384(3)
C^{15}	C^{29}	1.544(2)	C^{11}	C^{12}	1.390(2)

Table S26 Bond Angles for **4ae.**

Atom	Atom	Atom	Angle/˚	Atom	Atom	Atom	Angle/˚
C^{2}	O^{1}	C^{1}	116.72(13)	C^{24}	C^{23}	C^{22}	120.48(14)
C^{16}	C^{21}	C^{22}	120.04(13)	C^{6}	C^{5}	C^{8}	123.07(14)
C^{20}	C^{21}	C^{16}	119.43(15)	C^{4}	C^{5}	C^{8}	119.47(14)
C^{20}	C^{21}	C^{22}	120.53(14)	C^{4}	C^{5}	C^{6}	117.46(14)
C^{21}	C^{16}	C^{15}	121.32(14)	C^{11}	C^{10}	C^{9}	120.54(15)
C^{17}	C^{16}	C^{21}	118.96(14)	C^{23}	C^{24}	C^{25}	119.91(15)
C^{17}	C^{16}	C^{15}	119.66(14)	C^{7}	C^{6}	C^{5}	121.13(14)
C^{27}	C^{22}	C^{21}	119.09(14)	C^{13}	C^{14}	C^{9}	120.72(16)
C^{23}	C^{22}	C^{21}	121.21(14)	C^{10}	C^{9}	C^{8}	120.65(14)
C^{23}	C^{22}	C^{27}	119.68(14)	C^{14}	C^{9}	C^{8}	120.45(15)
C^{22}	C^{27}	C^{28}	119.02(14)	C^{14}	C^{9}	C^{10}	118.83(14)
C^{26}	C^{27}	C^{22}	119.09(15)	C^{2}	C^{3}	C^{4}	119.34(15)
C^{26}	C^{27}	C^{28}	121.88(14)	C^{17}	C^{18}	C^{19}	120.05(15)
C^{15}	C^{8}	C^{5}	122.56(14)	C^{6}	C^{7}	C^{2}	119.95(15)
C^{15}	C^{8}	C^{9}	124.77(14)	C^{15}	C^{29}	C^{28}	108.35(12)
C^{9}	C^{8}	C^{5}	112.58(13)	C^{31}	C^{29}	C^{28}	107.40(13)
C^{19}	C^{20}	C^{21}	120.64(15)	C^{31}	C^{29}	C^{15}	108.55(13)
C^{27}	C^{28}	C^{29}	113.91(13)	C^{30}	C^{29}	C^{28}	106.99(13)
C^{25}	C^{26}	C^{27}	120.85(14)	C^{30}	C^{29}	C^{15}	115.55(13)
C^{16}	C^{15}	C^{29}	115.12(13)	C^{30}	C^{29}	C^{31}	109.70(13)
C^{8}	C^{15}	C^{16}	117.83(13)	C^{12}	C^{13}	C^{14}	119.89(16)
C^{8}	C^{15}	C^{29}	126.93(14)	C^{26}	C^{25}	C^{24}	119.99(15)
O^{1}	C^{2}	C^{3}	124.09(14)	C^{3}	C^{4}	C^{5}	121.96(15)
O^{1}	C^{2}	C^{7}	115.91(14)	C^{18}	C^{19}	C^{20}	119.91(15)
C^{3}	C^{2}	C^{7}	119.99(14)	C^{10}	C^{11}	C^{12}	120.14(16)
C^{18}	C^{17}	C^{16}	120.97(15)	C^{13}	C^{12}	C^{11}	119.85(15)

Table S27 Torsion Angles for **4ae.**

A	B	C	D	Angle/˚	A	B	C	D	Angle/˚
O^1	C^2	C^3	C^4	179.24(15)	C^{26}	C^{27}	C^{28}	C^{29}	104.15(16)
O^1	C^2	C^7	C^6	-177.66(14)	C^{15}	C^{16}	C^{17}	C^{18}	176.08(14)
C^{21}	C^{16}	C^{15}	C^8	110.66(17)	C^{15}	C^8	C^5	C^6	-55.1(2)
C^{21}	C^{16}	C^{15}	C^{29}	-73.02(19)	C^{15}	C^8	C^5	C^4	124.36(18)
C^{21}	C^{16}	C^{17}	C^{18}	-1.2(2)	C^{15}	C^8	C^9	C^{10}	105.39(19)
C^{21}	C^{22}	C^{27}	C^{28}	-0.4(2)	C^{15}	C^8	C^9	C^{14}	-77.7(2)
C^{21}	C^{22}	C^{27}	C^{26}	178.19(13)	C^2	C^3	C^4	C^5	-2.0(3)
C^{21}	C^{22}	C^{23}	C^{24}	-178.35(14)	C^{17}	C^{16}	C^{15}	C^8	-66.6(2)
C^{21}	C^{20}	C^{19}	C^{18}	-0.5(2)	C^{17}	C^{16}	C^{15}	C^{29}	109.76(16)
C^{16}	C^{21}	C^{22}	C^{27}	47.3(2)	C^{17}	C^{18}	C^{19}	C^{20}	1.3(2)
C^{16}	C^{21}	C^{22}	C^{23}	-134.55(15)	C^{23}	C^{22}	C^{27}	C^{28}	-178.54(13)
C^{16}	C^{21}	C^{20}	C^{19}	-1.1(2)	C^{23}	C^{22}	C^{27}	C^{26}	0.0(2)
C^{16}	C^{15}	C^{29}	C^{28}	35.48(18)	C^{23}	C^{24}	C^{25}	C^{26}	-0.5(2)
C^{16}	C^{15}	C^{29}	C^{31}	-80.85(16)	C^5	C^8	C^{15}	C^{16}	-4.2(2)
C^{16}	C^{15}	C^{29}	C^{30}	155.47(14)	C^5	C^8	C^{15}	C^{29}	179.98(15)
C^{16}	C^{17}	C^{18}	C^{19}	-0.4(2)	C^5	C^8	C^9	C^{10}	-78.10(18)
C^{22}	C^{21}	C^{16}	C^{15}	4.8(2)	C^5	C^8	C^9	C^{14}	98.80(17)
C^{22}	C^{21}	C^{16}	C^{17}	-177.98(14)	C^5	C^6	C^7	C^2	-1.0(2)
C^{22}	C^{21}	C^{20}	C^{19}	178.82(14)	C^{10}	C^{11}	C^{12}	C^{13}	-0.2(2)
C^{22}	C^{27}	C^{28}	C^{29}	-77.35(17)	C^6	C^5	C^4	C^3	4.3(2)
C^{22}	C^{27}	C^{26}	C^{25}	0.0(2)	C^{14}	C^{13}	C^{12}	C^{11}	1.3(2)
C^{22}	C^{23}	C^{24}	C^{25}	0.5(2)	C^9	C^8	C^{15}	C^{16}	172.00(14)
C^{27}	C^{22}	C^{23}	C^{24}	-0.2(2)	C^9	C^8	C^{15}	C^{29}	-3.8(3)
C^{27}	C^{28}	C^{29}	C^{15}	51.71(17)	C^9	C^8	C^5	C^6	128.28(16)
C^{27}	C^{28}	C^{29}	C^{31}	168.79(13)	C^9	C^8	C^5	C^4	-52.2(2)
C^{27}	C^{28}	C^{29}	C^{30}	-73.49(16)	C^9	C^{10}	C^{11}	C^{12}	-1.7(2)
C^{27}	C^{26}	C^{25}	C^{24}	0.3(2)	C^9	C^{14}	C^{13}	C^{12}	-0.6(2)
C^8	C^{15}	C^{29}	C^{28}	-148.59(16)	C^3	C^2	C^7	C^6	3.3(2)
C^8	C^{15}	C^{29}	C^{31}	95.07(19)	C^7	C^2	C^3	C^4	-1.8(3)
C^8	C^{15}	C^{29}	C^{30}	-28.6(2)	C^{13}	C^{14}	C^9	C^8	-178.21(14)
C^8	C^5	C^6	C^7	176.77(15)	C^{13}	C^{14}	C^9	C^{10}	-1.3(2)
C^8	C^5	C^4	C^3	-175.24(15)	C^4	C^5	C^6	C^7	-2.7(2)
C^{20}	C^{21}	C^{16}	C^{15}	-175.32(14)	C^{11}	C^{10}	C^9	C^8	179.33(14)
C^{20}	C^{21}	C^{16}	C^{17}	1.9(2)	C^{11}	C^{10}	C^9	C^{14}	2.4(2)
C^{20}	C^{21}	C^{22}	C^{27}	-132.61(15)	C^1	O^1	C^2	C^3	-15.3(2)
C^{20}	C^{21}	C^{22}	C^{23}	45.5(2)	C^1	O^1	C^2	C^7	165.75(15)
C^{28}	C^{27}	C^{26}	C^{25}	178.45(14)					

Table S28 Hydrogen Atom Coordinates (Å×10^4) and Isotropic Displacement Parameters (Å^2×10^3) for **4ae**.

Atom	*x*	*y*	*z*	U(eq)
H^{20}	6559.84	2393.34	4244.21	27
H^{28A}	9498.49	1437.31	5851.68	24
H^{28B}	8610.29	531.74	5521.02	24
H^{26}	9760.27	4803.91	5645.59	25
H^{17}	6681.07	-1013.29	6223.03	27
H^{23}	6949.16	5798.93	4687.66	26
H^{10}	7993.75	6045.79	7513.25	27
H^{24}	7908.49	8257.08	4640.59	28
H^6	6403.63	4798.73	6015.14	26

H^{14}	8010.1	303.95	7980.11	28
H^{3}	4990.01	1898.32	7475.04	30
H^{18}	5711.06	-1885.96	5258.67	31
H^{7}	4981.37	5260.81	5860.47	27
H^{13}	8714.47	1019.93	9071.44	31
H^{25}	9316.38	7745.2	5112.74	29
H^{4}	6421.21	1560.35	7652.43	29
H^{19}	5633.31	-150.98	4272.98	31
H^{31A}	8456.56	-1621.65	6413.75	41
H^{31B}	8807.93	-983.72	7172.42	41
H^{31C}	9423.97	-1190.88	6685.82	41
H^{11}	8734.93	6746.87	8592.13	31
H^{30A}	9905.96	2333.79	6947.45	42
H^{30B}	9306.37	2386.19	7450.77	42
H^{30C}	9223.36	4016.9	6884.26	42
H^{12}	9097.58	4235.42	9373.89	32
H^{1A}	2940.81	2798.07	6626.93	50
H^{1B}	3660.49	2890.94	7294.07	50
H^{1C}	3675.34	1229.26	6752.5	50

Crystallographic data of 4ca are presented in Tables S29-36. A single crystal of **4ae** was coated in high-vacuum grease and mounted on a glass fiber. X-ray measurements were made using a Bruker D8 VENTURE PhotonIII area-detector diffractometer with Cu-Kα radiation (α = 1.54 Å). Absorption corrections were applied, based on multiple and symmetry-equivalent measurements. The structure was solved by ShelXT structure solution program using Intrinsic Phasing and refined with the XL refinement package using Least Squares minimization.[7] All non-hydrogen atoms were assigned anisotropic displacement parameters and refined without positional constraints and all other hydrogen atoms were constrained to ideal geometries and refined with fixed isotropic displacement parameters. Refinement proceeded smoothly to give the residuals shown in Table S30.

A colorless prism-like specimen of $C_{32}H_{29}ClO_2$, approximate dimensions 0.100 mm x 0.100 mm x 0.200 mm, was used for the X-ray crystallographic analysis. The X-ray intensity data were measured (λ = 1.54184 Å).

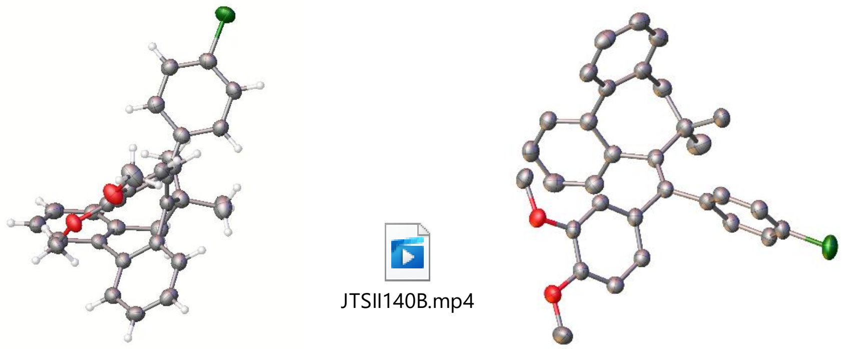

Table S29: Data collection details for 4ca.

Axis	dx/mm	2θ/°	ω/°	φ/°	χ/°	Width/°	Frames	Time/s	Wavelength/Å	Voltage/kV	Current/mA	Temperature/K
Omega	39.896	108.54	107.62	-120.00	-44.50	1.20	89	10.00	1.54184	50	1.1	100
Omega	39.896	108.54	107.62	80.00	-44.50	1.20	89	10.00	1.54184	50	1.1	100
Omega	39.896	108.54	107.62	160.00	-44.50	1.20	89	10.00	1.54184	50	1.1	100
Omega	39.896	108.54	-7.22	0.00	61.50	1.20	102	10.00	1.54184	50	1.1	100
Phi	39.896	-48.14	311.78	-93.65	24.00	1.20	216	10.00	1.54184	50	1.1	100
Omega	39.896	108.54	107.62	120.00	-44.50	1.20	89	10.00	1.54184	50	1.1	100
Omega	39.896	108.54	107.62	-160.00	-44.50	1.20	89	10.00	1.54184	50	1.1	100
Omega	39.896	108.54	107.62	-80.00	-44.50	1.20	89	10.00	1.54184	50	1.1	100
Omega	39.896	-18.14	-124.02	270.00	44.50	1.20	90	10.00	1.54184	50	1.1	100
Phi	39.896	93.54	91.61	0.00	-44.50	1.20	300	10.00	1.54184	50	1.1	100
Omega	39.896	108.54	-7.22	-40.00	61.50	1.20	102	10.00	1.54184	50	1.1	100
Omega	39.896	108.54	107.62	40.00	-44.50	1.20	89	10.00	1.54184	50	1.1	100
Omega	39.896	108.54	-7.22	-80.00	61.50	1.20	102	10.00	1.54184	50	1.1	100
Omega	39.896	108.54	-7.22	-120.00	61.50	1.20	102	10.00	1.54184	50	1.1	100

A total of 1637 frames were collected. The total exposure time was 4.55 hours. The frames were integrated with the Bruker SAINT software package using a narrow-frame algorithm. The integration of the data using a monoclinic unit cell yielded a total of 40092 reflections to a maximum θ angle of 72.52° (0.81 Å resolution). The final cell constants of a = 11.4512(7) Å, b = 9.9600(6) Å, c = 22.1248(14) Å, β = 95.211(3)°, volume = 2513.0(3) Å^3, are based upon the refinement of the XYZ-centroids of 9727 reflections above 20 σ(I) with 8.025 < 2θ < 145.8°. Data were corrected for absorption effects using the Multi-Scan method (SADABS). The ratio of minimum to maximum apparent transmission was 0.594.

Table S30 Crystal data and structure refinement for **4ca**.

Empirical formula	$C_{32}H_{29}ClO_2$
Formula weight	481.00
Temperature/K	100.0
Crystal system	monoclinic
Space group	$P2_1/c$
a/Å	11.4512(7)
b/Å	9.9600(6)
c/Å	22.1248(14)
α/°	90
β/°	95.211(3)
γ/°	90
Volume/Å^3	2513.0(3)
Z	4
ρ_{calc}g/cm^3	1.271
μ/mm^{-1}	1.551
F(000)	1016.0
Crystal size/mm^3	0.2 × 0.1 × 0.1
Radiation	CuKα (λ = 1.54178)
2Θ range for data collection/°	7.752 to 145.898
Index ranges	-14 ≤ h ≤ 14, -12 ≤ k ≤ 12, -27 ≤ l ≤ 27
Reflections collected	32906
Independent reflections	4848 [R_{int} = 0.0783, R_{sigma} = 0.0532]
Data/restraints/parameters	4848/0/321
Goodness-of-fit on F^2	1.085
Final R indexes [I>=2σ (I)]	R_1 = 0.1090, wR_2 = 0.3147
Final R indexes [all data]	R_1 = 0.1154, wR_2 = 0.3196
Largest diff. peak/hole / e Å^{-3}	0.81/-0.47

Table S31 Fractional Atomic Coordinates (×10^4) and Equivalent Isotropic Displacement Parameters (Å^2×10^3) for **4ca**. U_{eq} is defined as 1/3 of of the trace of the orthogonalised U_{IJ} tensor.

Atom	*x*	*y*	*z*	U(eq)
Cl^1	1413.3(14)	5185.2(16)	4736.7(7)	54.8(5)
O^1	7125(3)	5579(4)	1684.5(16)	41.4(9)
O^2	8146(3)	6569(5)	2678.5(17)	48.8(10)
C^{30}	5174(4)	6073(5)	1996(2)	34.9(10)
C^{25}	4495(4)	6664(5)	2422(2)	34.4(10)
C^{10}	2597(5)	5552(5)	652(2)	37.0(11)
C^{26}	5065(4)	7204(5)	2942(2)	36.6(11)
C^{28}	6951(4)	6644(5)	2623(2)	36.7(11)
C^{11}	3275(4)	6819(5)	761(2)	36.8(11)
C^{27}	6282(5)	7207(5)	3046(2)	37.6(11)
C^{16}	3201(4)	7537(5)	1308(2)	36.3(11)
C^{22}	1910(4)	5590(6)	4034(2)	39.9(11)
C^{20}	2231(4)	7258(5)	3286(2)	38.3(11)
C^{23}	2355(5)	4610(6)	3690(2)	40.2(11)
C^{21}	1847(4)	6910(6)	3848(2)	39.7(11)
C^{29}	6384(4)	6104(5)	2090(2)	34.9(10)
C^{31}	6620(5)	5289(6)	1096(2)	43.3(12)
C^{14}	4500(5)	9218(6)	948(3)	46.8(13)
C^{12}	3974(5)	7292(6)	330(2)	40.1(11)
C^{17}	2553(4)	6988(5)	1813(2)	36.0(11)
C^{15}	3829(5)	8744(6)	1387(2)	41.4(12)
C^{13}	4589(5)	8491(6)	420(3)	45.0(12)
C^{19}	2668(4)	6276(6)	2919(2)	37.0(11)
C^1	583(5)	5871(7)	2025(3)	49.4(14)
C^9	3106(5)	4407(6)	423(2)	42.7(12)
C^{24}	2746(4)	4947(6)	3130(2)	39.8(11)
C^6	742(5)	4390(6)	608(2)	45.5(13)
C^8	2435(6)	3264(6)	287(2)	48.5(14)
C^{18}	3178(4)	6649(5)	2336(2)	36.5(11)
C^5	1396(5)	5536(6)	747(2)	40.4(12)
C^3	1195(5)	6986(6)	1690(2)	43.2(12)
C^4	866(5)	6784(6)	999(2)	43.0(12)
C^7	1251(6)	3254(6)	368(2)	48.1(13)
C^{32}	8706(5)	6984(9)	3245(3)	59.0(17)
C^2	717(6)	8359(8)	1861(3)	60.2(17)

Table S32 Anisotropic Displacement Parameters (Å^2×10^3) for **4ca**. The Anisotropic displacement factor exponent takes the form: $-2\pi^2[h^2a^{*2}U_{11}+2hka^*b^*U_{12}+\ldots]$.

Atom	U_{11}	U_{22}	U_{33}	U_{23}	U_{13}	U_{12}
Cl^1	58.2(9)	58.2(9)	51.9(8)	8.0(6)	26.1(6)	-0.2(7)
O^1	35.5(18)	51(2)	38.6(18)	-5.1(16)	8.8(14)	6.8(16)
O^2	31.3(18)	71(3)	45(2)	-9.8(19)	7.1(15)	-3.4(18)
C^{30}	39(2)	31(2)	34(2)	0.0(19)	3.1(19)	-0.4(19)
C^{25}	35(2)	33(2)	37(2)	2.1(19)	8.9(19)	1.4(19)
C^{10}	43(3)	39(3)	28(2)	-0.1(19)	3.5(19)	0(2)
C^{26}	39(2)	35(2)	37(2)	-1(2)	7.4(19)	2(2)
C^{28}	33(2)	38(3)	40(2)	0(2)	7.5(19)	1(2)
C^{11}	38(2)	37(3)	36(2)	2(2)	2.6(19)	4(2)
C^{27}	41(3)	33(2)	39(2)	-2(2)	6(2)	-2(2)
C^{16}	38(2)	35(3)	35(2)	-0.2(19)	2.4(19)	6(2)
C^{22}	34(2)	46(3)	41(3)	3(2)	8(2)	-2(2)
C^{20}	36(2)	34(3)	45(3)	2(2)	8(2)	-1(2)
C^{23}	38(3)	38(3)	45(3)	4(2)	6(2)	1(2)

C^{21}	36(2)	44(3)	41(3)	-4(2)	11(2)	2(2)
C^{29}	35(2)	35(2)	36(2)	-0.1(19)	10.7(19)	1.7(19)
C^{31}	43(3)	50(3)	38(3)	0(2)	11(2)	9(2)
C^{14}	54(3)	35(3)	50(3)	4(2)	-3(3)	-2(2)
C^{12}	42(3)	41(3)	38(2)	1(2)	7(2)	4(2)
C^{17}	37(2)	33(2)	39(2)	-3(2)	9(2)	3(2)
C^{15}	43(3)	38(3)	43(3)	-1(2)	4(2)	6(2)
C^{13}	47(3)	46(3)	44(3)	10(2)	10(2)	0(2)
C^{19}	31(2)	43(3)	37(2)	0(2)	4.2(19)	0(2)
C^{1}	34(3)	72(4)	43(3)	-1(3)	7(2)	-6(3)
C^{9}	51(3)	44(3)	34(2)	-2(2)	8(2)	0(2)
C^{24}	34(2)	43(3)	43(3)	-1(2)	4(2)	-2(2)
C^{6}	49(3)	46(3)	41(3)	7(2)	0(2)	-7(2)
C^{8}	72(4)	35(3)	38(3)	-2(2)	5(3)	0(3)
C^{18}	32(2)	39(3)	38(2)	-6(2)	6.3(19)	2(2)
C^{5}	43(3)	45(3)	33(2)	3(2)	1(2)	-1(2)
C^{3}	35(3)	53(3)	42(3)	-1(2)	5(2)	4(2)
C^{4}	36(3)	46(3)	46(3)	2(2)	1(2)	3(2)
C^{7}	64(4)	41(3)	39(3)	3(2)	0(2)	-11(3)
C^{32}	37(3)	87(5)	53(3)	-15(3)	2(2)	-8(3)
C^{2}	54(4)	65(4)	59(4)	-10(3)	-4(3)	19(3)

Table S33 Bond Lengths for **4ca**.

**Atom**	**Atom**	**Length/Å**	**Atom**	**Atom**	**Length/Å**
Cl^{1}	C^{22}	1.751(5)	C^{22}	C^{21}	1.378(8)
O^{1}	C^{29}	1.391(6)	C^{20}	C^{21}	1.401(7)
O^{1}	C^{31}	1.405(7)	C^{20}	C^{19}	1.393(7)
O^{2}	C^{28}	1.365(6)	C^{23}	C^{24}	1.397(8)
O^{2}	C^{32}	1.417(7)	C^{14}	C^{15}	1.376(8)
C^{30}	C^{25}	1.405(7)	C^{14}	C^{13}	1.386(8)
C^{30}	C^{29}	1.383(7)	C^{12}	C^{13}	1.392(8)
C^{25}	C^{26}	1.378(7)	C^{17}	C^{18}	1.347(7)
C^{25}	C^{18}	1.503(7)	C^{17}	C^{3}	1.555(7)
C^{10}	C^{11}	1.490(7)	C^{19}	C^{24}	1.404(8)
C^{10}	C^{9}	1.396(8)	C^{19}	C^{18}	1.508(7)
C^{10}	C^{5}	1.410(8)	C^{1}	C^{3}	1.539(9)
C^{26}	C^{27}	1.392(7)	C^{9}	C^{8}	1.392(8)
C^{28}	C^{27}	1.381(7)	C^{6}	C^{5}	1.384(8)
C^{28}	C^{29}	1.402(7)	C^{6}	C^{7}	1.399(9)
C^{11}	C^{16}	1.415(7)	C^{8}	C^{7}	1.384(9)
C^{11}	C^{12}	1.381(7)	C^{5}	C^{4}	1.512(8)
C^{16}	C^{17}	1.499(7)	C^{3}	C^{4}	1.554(8)
C^{16}	C^{15}	1.403(8)	C^{3}	C^{2}	1.533(9)
C^{22}	C^{23}	1.364(8)			

Table S34 Bond Angles for **4ca**.

**Atom**	**Atom**	**Atom**	**Angle/˚**	**Atom**	**Atom**	**Atom**	**Angle/˚**
C^{29}	O^{1}	C^{31}	116.8(4)	C^{15}	C^{14}	C^{13}	120.3(5)
C^{28}	O^{2}	C^{32}	115.7(4)	C^{11}	C^{12}	C^{13}	120.8(5)
C^{29}	C^{30}	C^{25}	119.9(4)	C^{16}	C^{17}	C^{3}	115.2(4)
C^{30}	C^{25}	C^{18}	121.4(4)	C^{18}	C^{17}	C^{16}	118.1(4)
C^{26}	C^{25}	C^{30}	118.3(4)	C^{18}	C^{17}	C^{3}	126.5(4)
C^{26}	C^{25}	C^{18}	120.2(4)	C^{14}	C^{15}	C^{16}	121.3(5)
C^{9}	C^{10}	C^{11}	121.4(5)	C^{14}	C^{13}	C^{12}	119.5(5)
C^{9}	C^{10}	C^{5}	119.3(5)	C^{20}	C^{19}	C^{24}	118.9(5)

C^{5}	C^{10}	C^{11}	119.1(5)	C^{20}	C^{19}	C^{18}	120.8(5)
C^{25}	C^{26}	C^{27}	122.0(5)	C^{24}	C^{19}	C^{18}	119.9(5)
O^{2}	C^{28}	C^{27}	125.1(5)	C^{8}	C^{9}	C^{10}	120.4(5)
O^{2}	C^{28}	C^{29}	116.0(4)	C^{23}	C^{24}	C^{19}	120.4(5)
C^{27}	C^{28}	C^{29}	118.9(4)	C^{5}	C^{6}	C^{7}	121.1(6)
C^{16}	C^{11}	C^{10}	119.7(5)	C^{7}	C^{8}	C^{9}	120.4(6)
C^{12}	C^{11}	C^{10}	120.2(5)	C^{25}	C^{18}	C^{19}	110.9(4)
C^{12}	C^{11}	C^{16}	120.1(5)	C^{17}	C^{18}	C^{25}	123.7(4)
C^{28}	C^{27}	C^{26}	119.7(5)	C^{17}	C^{18}	C^{19}	125.4(4)
C^{11}	C^{16}	C^{17}	121.7(5)	C^{10}	C^{5}	C^{4}	118.4(5)
C^{15}	C^{16}	C^{11}	118.0(5)	C^{6}	C^{5}	C^{10}	119.4(5)
C^{15}	C^{16}	C^{17}	120.2(5)	C^{6}	C^{5}	C^{4}	122.2(5)
C^{23}	C^{22}	Cl^{1}	119.9(4)	C^{1}	C^{3}	C^{17}	114.1(5)
C^{23}	C^{22}	C^{21}	122.0(5)	C^{1}	C^{3}	C^{4}	107.5(5)
C^{21}	C^{22}	Cl^{1}	118.1(4)	C^{4}	C^{3}	C^{17}	108.7(4)
C^{19}	C^{20}	C^{21}	120.2(5)	C^{2}	C^{3}	C^{17}	109.2(5)
C^{22}	C^{23}	C^{24}	119.3(5)	C^{2}	C^{3}	C^{1}	109.7(5)
C^{22}	C^{21}	C^{20}	119.2(5)	C^{2}	C^{3}	C^{4}	107.3(5)
O^{1}	C^{29}	C^{28}	115.1(4)	C^{5}	C^{4}	C^{3}	113.6(4)
C^{30}	C^{29}	O^{1}	123.8(4)	C^{8}	C^{7}	C^{6}	119.3(5)
C^{30}	C^{29}	C^{28}	121.1(4)				

Table S35 Torsion Angles for **4ca**.

A	B	C	D	Angle/˚	A	B	C	D	Angle/˚
Cl^{1}	C^{22}	C^{23}	C^{24}	179.9(4)	C^{21}	C^{20}	C^{19}	C^{18}	-174.9(4)
Cl^{1}	C^{22}	C^{21}	C^{20}	-179.5(4)	C^{29}	C^{30}	C^{25}	C^{26}	-3.1(7)
O^{2}	C^{28}	C^{27}	C^{26}	-179.0(5)	C^{29}	C^{30}	C^{25}	C^{18}	-179.9(5)
O^{2}	C^{28}	C^{29}	O^{1}	-0.8(7)	C^{29}	C^{28}	C^{27}	C^{26}	1.3(8)
O^{2}	C^{28}	C^{29}	C^{30}	177.3(5)	C^{31}	O^{1}	C^{29}	C^{30}	14.9(7)
C^{30}	C^{25}	C^{26}	C^{27}	1.6(7)	C^{31}	O^{1}	C^{29}	C^{28}	-167.0(5)
C^{30}	C^{25}	C^{18}	C^{17}	-47.7(7)	C^{12}	C^{11}	C^{16}	C^{17}	-173.6(5)
C^{30}	C^{25}	C^{18}	C^{19}	135.2(5)	C^{12}	C^{11}	C^{16}	C^{15}	1.7(7)
C^{25}	C^{30}	C^{29}	O^{1}	-178.1(5)	C^{17}	C^{16}	C^{15}	C^{14}	175.2(5)
C^{25}	C^{30}	C^{29}	C^{28}	3.9(7)	C^{17}	C^{3}	C^{4}	C^{5}	55.5(6)
C^{25}	C^{26}	C^{27}	C^{28}	-0.7(8)	C^{15}	C^{16}	C^{17}	C^{18}	-62.3(7)
C^{10}	C^{11}	C^{16}	C^{17}	6.3(7)	C^{15}	C^{16}	C^{17}	C^{3}	112.7(5)
C^{10}	C^{11}	C^{16}	C^{15}	-178.3(4)	C^{15}	C^{14}	C^{13}	C^{12}	1.4(8)
C^{10}	C^{11}	C^{12}	C^{13}	178.4(5)	C^{13}	C^{14}	C^{15}	C^{16}	-1.3(8)
C^{10}	C^{9}	C^{8}	C^{7}	0.9(8)	C^{19}	C^{20}	C^{21}	C^{22}	0.2(8)
C^{10}	C^{5}	C^{4}	C^{3}	-75.8(6)	C^{1}	C^{3}	C^{4}	C^{5}	-68.5(6)
C^{26}	C^{25}	C^{18}	C^{17}	135.6(5)	C^{9}	C^{10}	C^{11}	C^{16}	-134.9(5)
C^{26}	C^{25}	C^{18}	C^{19}	-41.5(6)	C^{9}	C^{10}	C^{11}	C^{12}	45.1(7)
C^{11}	C^{10}	C^{9}	C^{8}	-175.7(5)	C^{9}	C^{10}	C^{5}	C^{6}	-0.5(7)
C^{11}	C^{10}	C^{5}	C^{6}	175.7(5)	C^{9}	C^{10}	C^{5}	C^{4}	179.5(5)
C^{11}	C^{10}	C^{5}	C^{4}	-4.3(7)	C^{9}	C^{8}	C^{7}	C^{6}	-2.1(8)
C^{11}	C^{16}	C^{17}	C^{18}	112.9(6)	C^{24}	C^{19}	C^{18}	C^{25}	-72.6(6)
C^{11}	C^{16}	C^{17}	C^{3}	-72.0(6)	C^{24}	C^{19}	C^{18}	C^{17}	110.4(6)
C^{11}	C^{16}	C^{15}	C^{14}	-0.3(7)	C^{6}	C^{5}	C^{4}	C^{3}	104.2(6)
C^{11}	C^{12}	C^{13}	C^{14}	0.0(8)	C^{18}	C^{25}	C^{26}	C^{27}	178.3(5)
C^{27}	C^{28}	C^{29}	O^{1}	178.9(5)	C^{18}	C^{17}	C^{3}	C^{1}	-34.0(8)
C^{27}	C^{28}	C^{29}	C^{30}	-3.0(8)	C^{18}	C^{17}	C^{3}	C^{4}	-153.9(5)

C^{16}	C^{11}	C^{12}	C^{13}	-1.6(8)	C^{18}	C^{17}	C^{3}	C^{2}	89.3(7)
C^{16}	C^{17}	C^{18}	C^{25}	-6.1(7)	C^{18}	C^{19}	C^{24}	C^{23}	175.3(4)
C^{16}	C^{17}	C^{18}	C^{19}	170.6(5)	C^{5}	C^{10}	C^{11}	C^{16}	49.0(7)
C^{16}	C^{17}	C^{3}	C^{1}	151.5(5)	C^{5}	C^{10}	C^{11}	C^{12}	-131.1(5)
C^{16}	C^{17}	C^{3}	C^{4}	31.5(6)	C^{5}	C^{10}	C^{9}	C^{8}	0.4(8)
C^{16}	C^{17}	C^{3}	C^{2}	-85.3(6)	C^{5}	C^{6}	C^{7}	C^{8}	2.0(8)
C^{22}	C^{23}	C^{24}	C^{19}	-0.9(8)	C^{3}	C^{17}	C^{18}	C^{25}	179.5(5)
C^{20}	C^{19}	C^{24}	C^{23}	2.1(7)	C^{3}	C^{17}	C^{18}	C^{19}	-3.8(9)
C^{20}	C^{19}	C^{18}	C^{25}	100.5(5)	C^{7}	C^{6}	C^{5}	C^{10}	-0.7(8)
C^{20}	C^{19}	C^{18}	C^{17}	-76.5(7)	C^{7}	C^{6}	C^{5}	C^{4}	179.3(5)
C^{23}	C^{22}	C^{21}	C^{20}	1.1(8)	C^{32}	O^{2}	C^{28}	C^{27}	6.9(8)
C^{21}	C^{22}	C^{23}	C^{24}	-0.7(8)	C^{32}	O^{2}	C^{28}	C^{29}	-173.5(5)
C^{21}	C^{20}	C^{19}	C^{24}	-1.7(7)	C^{2}	C^{3}	C^{4}	C^{5}	173.5(5)

Table S36 Hydrogen Atom Coordinates (Å×10^4) and Isotropic Displacement Parameters (Å^2×10^3) for **JTSII140B**.

**Atom**	***x***	***y***	***z***	**U(eq)**
H^{30}	4802.64	5653.87	1643.47	42
H^{26}	4612.62	7585.65	3237.56	44
H^{27}	6650.71	7595.81	3406.31	45
H^{20}	2193.32	8166.67	3154.24	46
H^{23}	2397.03	3707.56	3829.84	48
H^{21}	1546	7577.39	4099.15	48
H^{31A}	7240.41	5075.57	834.42	65
H^{31B}	6176.54	6069.75	933.4	65
H^{31C}	6091.67	4517.72	1111.4	65
H^{14}	4904.5	10046.8	1006.87	56
H^{12}	4034.48	6792.54	-31.14	48
H^{15}	3788.84	9243.65	1750.34	50
H^{13}	5067.53	8808.34	121.19	54
H^{1A}	913.9	4997.93	1928.68	74
H^{1B}	-259.05	5878.56	1896.34	74
H^{1C}	703.3	6026.4	2463.8	74
H^{9}	3916.16	4408.85	360.5	51
H^{24}	3067.38	4272.98	2889.91	48
H^{6}	-64.63	4375.02	676	55
H^{8}	2791.49	2485.52	136.53	58
H^{4A}	2.05	6731.98	923	52
H^{4B}	1131.18	7576.86	779.1	52
H^{7}	788.32	2482.91	262.03	58
H^{32A}	9555.66	6865.73	3244.42	89
H^{32B}	8419.82	6440.3	3570.19	89
H^{32C}	8530.62	7932.1	3312.86	89
H^{2A}	849.51	8486.72	2300.76	90
H^{2B}	-126.1	8400.66	1736.42	90
H^{2C}	1120.88	9067.54	1653.77	90
